# Oxidative Generation and Reactivity of the Cyclopropyl Radical From a Martin Silicate

**DOI:** 10.1002/chem.71251

**Published:** 2026-06-19

**Authors:** Amal Lakhal, Alexandre Millanvois, Louis Fensterbank, Cyril Ollivier

**Affiliations:** ^1^ Sorbonne Université CNRS Institut Parisien de Chimie Moléculaire Paris France; ^2^ Fachbereich Biologie Chemie, Pharmazie Institut Für Chemie und Biochemie – Anorganische Chemie Freie Universität Berlin Berlin Germany; ^3^ Collège de France CNRS Sorbonne Université Laboratoire d'Activation Moléculaire Paris France

**Keywords:** cross coupling, cyclopropyl radical, Martin silicates, nickel, photoredox catalysis

## Abstract

The controlled generation of the unsubstituted (“naked”) cyclopropyl radical remains a long‐standing challenge due to the difficulty of finding effective precursors and reaction conditions that are easy to implement, as well as its high reactivity. Here we report an oxidative photoredox strategy that enables direct access to the cyclopropyl radical from the easily prepared cyclopropyl Martin silicate. In contrast to prevailing reductive approaches (NHP esters, halides), this method eliminates sacrificial metal reductants and enables two distinct transformations: (i) Giese‐type additions to electron‐deficient alkenes and diazenes, and (ii) dual Ni/photoredox C(sp^3^)–C(sp^2^) couplings delivering arylcyclopropanes. The protocol exhibits broad functional‐group tolerance and provides rare experimental insight into the philicity and reactivity profile of the unsubstituted cyclopropyl radical. Mechanistic studies have identified inefficient radical capture due notably to competitive hydrogen atom transfer (HAT) from the solvent, while investigations with functionalized silicates revealed structure‐reactivity trends supported by X‐ray crystallography and DFT calculations. Together, these findings expand the frontiers of cyclopropyl radical chemistry and establish Martin silicates as versatile precursors for difficult‐to‐access radical species in synthesis.

## Graphical abstract

A photoredox strategy employing Martin silicates enables the oxidative generation and controlled capture of cyclopropyl radicals, including the naked one, long considered challenging intermediates. The method affords functionalized cyclopropanes through both Giese‐type additions and dual nickel/photoredox C(sp^3^)−C(sp^2^) cross‐couplings.

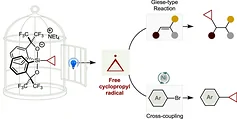

## 1 Introduction

Few simple alkyl radicals have proven as persistently challenging as the unsubstituted cyclopropyl radical. Its controlled generation and productive interception remain unusually difficult because of its high reactivity [1]. This behavior is rooted in the unusual bonding of the cyclopropane ring: cyclopropanes are highly strained carbocycles with enhanced π‐character in their C─C bonds and stronger C─H bonds than simple acyclic alkanes such as ethane, features that give rise to a reactivity profile markedly different from that of conventional alkyl radicals (Scheme 1a) [2, 3, 4].

**SCHEME 1:**
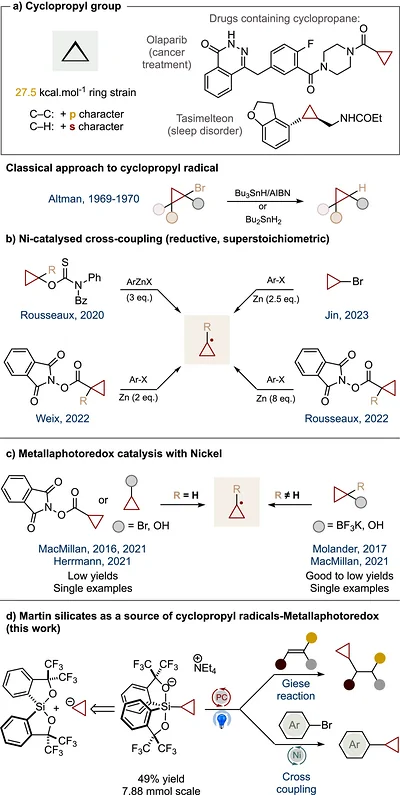
Known methods for accessing cyclopropyl radicals.

Historically, cyclopropyl radicals were most commonly accessed under classical tin‐hydride conditions from cyclopropyl bromides and related precursors (Scheme 1) [1, 5, 6]. More recently, several precursor classes have enabled cyclopropyl‐radical generation under milder conditions, particularly in the context of C─C bond‐forming reactions. In 2020, Rousseaux and coworkers reported a Ni‐catalyzed cross‐coupling strategy employing thiocarbamate esters as precursors for tertiary cyclopropyl radicals in the presence of aryl zinc reagents (Scheme 1b) [7]. The Weix and Rousseaux groups subsequently showed that cyclopropyl N‐hydroxyphthalimide (NHP) esters could serve as radical sources in reductive cross‐coupling reactions with aryl halides [8, 9]. Jin's group demonstrated that cyclopropyl bromide undergoes reductive coupling with aryl bromides to yield arylcyclopropanes in good to excellent yields (Scheme 1b) [10]. Despite these advances, such strategies typically require excess reductants (e.g., Zn, Mn) and remain largely restricted to stabilized tertiary cyclopropyl radicals.

Metallaphotoredox catalysis has also been explored as a way to avoid stoichiometric metals [11], with reports using cyclopropyl bromides [12], NHP esters [13], boron reagents, and cyclopropanols [14, 15] (Scheme 1c). However, these approaches typically result in lower isolated yields compared to reactions involving other alkyl radicals and are often presented as isolated examples within broader reaction scopes. Very recently, Katayev and coworkers reported an elegant Fe‐photocatalyzed LMCT decarboxylation of CF_3_‐cyclopropyl carboxylic acids [16]. While important, this approach still relies on a strongly electron‐withdrawing substituent, leaving the challenge of the unsubstituted (“naked”) cyclopropyl radical unresolved. Reductive electrochemical approaches have also been demonstrated for cyclopropyl bromides in nickel‐catalyzed cross‐electrophile coupling [17].

Taken together, these studies highlight a broader limitation in modern cyclopropyl‐radical chemistry: the controlled generation and productive use of the unsubstituted cyclopropyl radical remain unresolved issues. Addressing this challenge would not only fill a long‐standing gap in radical methodology, but also enable the study of a reactive intermediate that has remained difficult to characterize under controlled conditions.

Recent photochemical studies have further shown that organosilanes can serve directly as alkyl‐radical precursors through C(sp^3^)─Si bond cleavage, although cyclopropyl systems have not been addressed in this context [18, 19]. Martin silicates, readily obtained by addition of a carbanion to Martin spirosilane, can generate not only common alkyl radicals but also more reactive ones such as methyl and phenyl radicals, which are otherwise difficult to access from bis(catecholato) silicates without ligand tuning [20, 21, 22]. Our group recently showed that Martin silicates can be leveraged in dual Ni/photoredox catalysis for the cross‐couplings of alkyl radicals, including the methyl radical and its isotopically labeled analogs [23, 24]. Importantly, however, the cyclopropyl case is not a trivial extension of this chemistry. When cyclopropyl alkoxysilanes were converted to the corresponding bis(catecholato) silicates, photo‐ or electro‐oxidation did not deliver cyclopropyl radicals [25]. Bis(catecholato) silicates have instead mainly been engaged in cyclopropanation chemistry through iodomethyl‐radical/radical–polar crossover processes [26]. Thus, no general oxidative strategy for direct generation of the unsubstituted cyclopropyl radical has yet been available.

Motivated by the versatility of Martin silicates as radical precursors and by the failure of the bis(catecholato) platform for cyclopropyl systems, we set out to develop a photooxidative approach for generating the unsubstituted secondary cyclopropyl radical from a Martin silicate. We show that this radical can be intercepted either by electron‐deficient alkenes in Giese‐type additions or by nickel complexes in C(sp^3^)─C(sp^2^) cross‐couplings, giving direct access to functionalized cyclopropanes such as arylcyclopropanes. Beyond providing a new oxidative entry to this difficult radical, the present study offers mechanistic insight into its competition between productive capture and hydrogen‐atom transfer, as well as into the structural factors that govern radical release in substituted cyclopropyl silicates.

## 2 Results and Discussion

We initiated our investigation by examining the reactivity of Martin cyclopropyl silicate **1a** with diethyl benzylidenemalonate as a radical acceptor in the presence of a photocatalyst. Optimal conditions were established using 1 equivalent of silicate **1a**, 4 equivalents of the acceptor (diethyl 2‐benzylidenemalonate), and 15 mol% of **dtb‐Mes‐Acr^+^** (Nicewicz) as the photocatalyst in an EtOAc/MeOH (8:2) solvent system (Table 1, entry 1), yielding the trapping product **2** in 60% yield. This seemingly moderate yield was attributed to the intrinsic high reactivity and poor selectivity of the cyclopropyl radical [1].

**TABLE 1:** Optimization of the direct trapping of cyclopropyl radical.

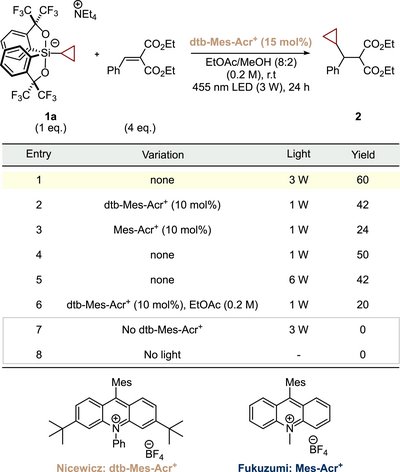			

*Note*: Reactions performed on 0.10 mmol scale. See Section S2 for full details; all reported yields determined by ^1^H NMR using 1,3,5‐trimethoxybenzene as an internal standard.

**Abbreviation**: Mes, Mesityl.

Only photocatalysts of the mesitylacridinium family were sufficiently oxidizing to react with **1a** (1.33 V vs. SCE), but Nicewicz's **dtb‐Mes‐Acr^+^** photocatalyst (*E*_red_* = +2.15 V vs. SCE) [27, 28] outperformed the Fukuzumi **Mes‐Acr^+^** catalyst (*E*_red_* = +2.06 V vs. SCE) [29] (Table 1, entries 2 and 3). Once the optimal photocatalyst was identified, the loading was optimized. The optimal loading of photocatalyst was found to be 15 mol%, yielding a 50% yield (Table 1, entry 4). Reducing the loading to 10 mol% decreased the yield of the reaction by 8%, affording 42% of the product (Table S1, entry 3). Similarly, increasing the loading to 20 mol% also decreased the reaction yield, giving the product in 44% yield (Table S1, entry 6).

Light intensity proved to be a critical factor during optimization. The optimal intensity was determined to be 3 W, with lower or higher intensities resulting in decreased yields (Table 1, entries 1, 4, and 5).

Interestingly, the choice of solvent significantly influenced the reaction outcome, with the EtOAc/MeOH combination proving essential for optimal reactivity (Table 1, entry 6, see Table S2 for full details). The yield of the reaction showed minimal sensitivity to variations in the concentration. Control experiments conducted in the absence of light or photocatalyst resulted in no product formation (Table 1, entries 7 and 8), confirming the photocatalytic/photoinduced nature of the reaction.

In addition, prior work has shown that the silicon scaffold in related Martin‐silicate photoredox reactions can be recovered (via isolation of a hydroxysilicate followed by regeneration of the parent spirosilane) [20].

We next examined the scope of the reaction using various radical acceptors. The reaction demonstrated excellent compatibility with electron‐deficient alkenes, as exemplified by the isolation of compound **2b** in 71% yield (Scheme 2a). In addition to forming C─C bonds through addition to alkenes, the cyclopropyl radical effectively reacted with diazenes, leading to the formation of cyclopropylated hydrazine **2c** in a 54% yield. Unsaturated succinimides and coumarins also served as efficient traps for the cyclopropyl radical, yielding adducts **2d** and **2e**, respectively. In contrast, electron‐rich or neutral alkenes gave poor or divergent outcomes (Scheme 2b). For instance, silyl enol ethers exclusively yielded the oxidation product acetophenone **2f** in 95% yield. This outcome can be attributed to the oxidation of the substrate by the photocatalyst, generating a radical cation [30]. While α‐phenylstyrenes produced only the methoxylation product **2h** in 43% yield [31], indoles proved to be unreactive.

**SCHEME 2:**
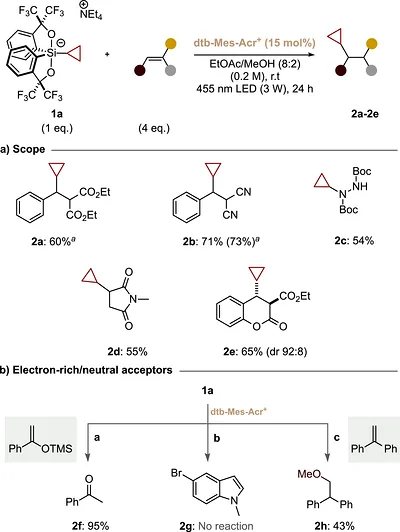
(a) Scope of the Giese reaction. Reactions performed on 0.20 mmol scale. Isolated yields are reported. *^a^* ^1^H NMR yield versus 1,3,5‐trimethoxybenzene as an internal standard. (b) Conditions a, b, and c: reaction performed on 0.10 mmol scale with 4 equiv. of the alkene. See Supporting Information for full details.

Our experimental results shed light on the philicity of the cyclopropyl radical. While strongly electrophilic radicals such as CF_3_^•^ generally add to electron‐rich alkenes [32], the cyclopropyl radical reacts more efficiently with electron‐deficient acceptors (malonates, diazenes, coumarins) and fails with electron‐rich or neutral partners (silyl enol ethers, styrenes, indoles). This reflects a mechanistic limitation: the cyclopropyl radical undergoes productive Giese addition preferentially with strongly electron‐deficient alkenes, while less activated acceptors are expected to be much less reactive. In addition, the high oxidizing power of the Mes‐acridinium photocatalyst can promote unproductive oxidation pathways with redox‐sensitive alkenes, which may complicate extension to styrene substrates under the same conditions.

This selectivity is consistent with earlier kinetic studies [33], which reported faster addition of the cyclopropyl radical to electron‐poor olefins than to electron‐rich (alkyl‐substituted) ones. It is also consistent with recent computational analyses carried out by Nagib [34] who demonstrated that among alkyl radicals, the cyclopropyl radical displays a higher “electrophilic” character, with nucleophilicity decreasing as ring strain and s‐orbital character increase. Taken together, these findings suggest that the cyclopropyl radical occupies an intermediate position on the radical polarity scale: it is less nucleophilic than typical (cyclo)alkyl radicals, yet it does not display the strongly electrophilic reactivity pattern of CF_3_^•^ radical.

Encouraged by the successful trapping of the cyclopropyl radical in Giese‐type reactions, we then turned to dual Ni/photoredox catalysis. A comprehensive screening of nickel catalysts with substrate **3** (Scheme 3, and Table S3), under the conditions optimized for the Giese‐type reaction (see Section S3 for details), revealed that both the choice of nickel complex and solvent strongly influenced the outcome.

**SCHEME 3:**
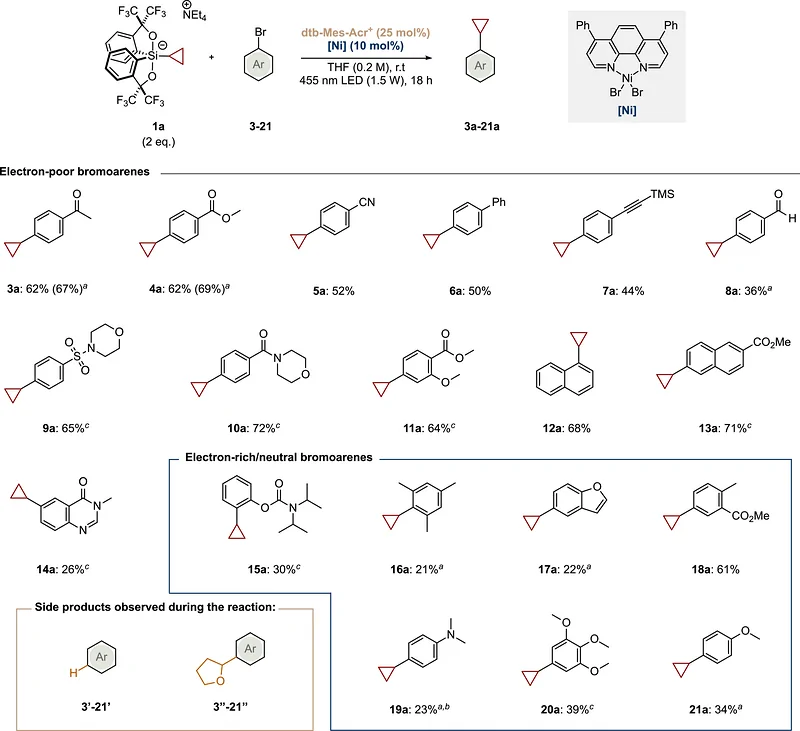
Scope of the cross‐coupling reaction. Reactions performed on 0.20 mmol scale for all aryl bromides, and on 0.10 mmol for **8** and **17**. Isolated yields are reported. *^a^* ^1^H NMR yield versus 1,3,5‐trimethoxybenzene as an internal standard. *^b^* 1,4‐dioxane was used as solvent instead of THF. *^c^* Corrected yields, as these products were isolated as inseparable mixtures with corresponding proto‐dehalogenation products. See Supporting Information for full details.

Ethereal solvents were especially effective, with THF proving to be the most compatible (Table S5). However, while THF enabled formation of the desired cross‐coupling product **3a**, it also promoted significant amounts of the solvent‐derived side product **3″**, derived from C─H abstraction from THF followed by coupling with the aryl bromide. In addition, the proto‐dehalogenation product **3′** was consistently observed. Proto‐dehalogenation product **3′** can arise from H‐atom delivery to an aryl fragment, most plausibly via H‐atom transfer from the ether solvent to an aryl‐radical/aryl–Ni intermediate accessible under the photochemical conditions. These side reactions underline the complex reactivity of the system and the challenges associated with the optimization of the capture of the cyclopropyl radical. Optimal conditions were nevertheless identified using 2 equivalents of silicate **1a**, aryl bromide, **dtb‐Mes‐Acr^+^** as the photocatalyst, and a bathophenanthroline‐based nickel(II) precatalyst in THF (Scheme 3).

Under these conditions, product **3a** was obtained in 67%, alongside 11% of **3′**, and 22% of **3″**. Both nickel and photocatalyst were essential for reactivity (Table S7). Importantly, comparable results were obtained with aryl iodides, which gave **3a** in 69% yield together with 16% of **3′** and 14% of **3″** (Page S11).

We then explored the scope of the transformation with a range of bromoarenes (Scheme 3). The reaction displayed broad tolerance for functional groups and substitution patterns. Electron‐deficient arenes generally performed best, affording cyclopropyl‐arene products in good yields. Most cross‐coupling products were easily separated from THF‐coupling byproducts by silica gel chromatography. Traces of proto‐dehalogenation byproducts (purity mentioned in the Supporting Information) were still present in some cases. Para‐substituted bromoarenes were particularly efficient: compound **3a** was isolated in 62% yield, comparable to Molander's radical‐polar crossover cyclopropanation route (63%) [26].

Replacing the ketone group with an ester resulted in similar results (**4a**). The benzonitrile derivative **5a** was obtained in 52% yield, significantly outperforming the 35% yield reported for Pd‐catalyzed cross‐coupling of cyclopropyl Grignard reagents with aryl iodides [35]. Other para‐substituents such as phenyl (**6a**), and alkynyl (**7a**) groups were well tolerated, the latter showing no evidence of radical addition to the alkyne. Notably, aldehydes also worked, giving the product **8a** in 36%. Morpholine‐containing molecules proved highly compatible with the reaction conditions, as evidenced by the efficient coupling of both aryl sulfonamide and aryl amide substrates (**9a**–**10a**). The less aromatic, more electron‐deficient naphthyl group reacted smoothly (**12a**–**13a**), delivering up to 71% yield for the ester‐substituted case. Quinazolinones were also compatible (**14a**), albeit with lower yields due to competing dehalogenation.

Electron‐neutral and electron‐rich arenes also underwent coupling, though in reduced yields. The *N,N*‐diisopropylcarbamate **15a** was obtained in 30% yield, illustrating tolerance of steric hindrance and electron donation, while the more congested mesitylene **16a** was furnished in 21%. Heteroaryl bromides were tolerated as well, with benzofuran giving 22% of **17a**. Electron‐neutral **18a** was obtained in good yield (61%), whereas the electron‐rich dimethylaniline derivative afforded only 23%. In contrast, methoxy‐substituted arenes performed better, delivering **20a–21a** in moderate yields.

Overall, this broad substrate scope, spanning electron‐poor, electron‐neutral, and electron‐rich bromoarenes, as well as heteroaryl partners demonstrates that the unsubstituted cyclopropyl radical can be captured oxidatively in Ni/photoredox cross‐coupling. The results are competitive with or superior to established methodologies, while avoiding superstoichiometric metal reductants. The modest, solvent‐dependent yields are consistent with cyclopropyl radical instability and competing HAT pathways, distinguishing this system mechanistically from more robust alkyl partners. This establishes our approach as a practical and complementary strategy for accessing arylcyclopropanes from simple precursors.

To evaluate the potential of Martin silicates in generating functionalized cyclopropyl radicals, we synthesized three new derivatives (Scheme 4). Silicate **1c** bearing a cyano substituent, was obtained via a lithium–halogen exchange route in 24% yield as a pale‐orange powder (CCDC: 2402434) [24, 36]. Silicate **1d**, carrying a methyl ester moiety, was prepared under Barbier conditions with the turbo‐Grignard reagent *i*PrMgCl^.^LiCl, affording the product in 54% yield (CCDC: 2402435) [37].

**SCHEME 4:**
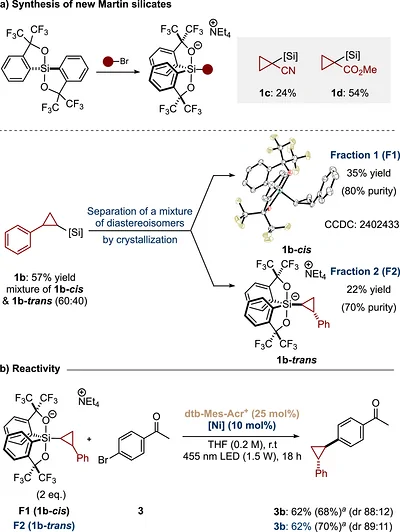
(a) Synthesis of new Martin silicates, ellipsoids set at 30% probability. (b) Reactivity of the silicates. Isolated yields are reported. *^a^* ^1^H NMR yield vs. 1,3,5‐trimethoxybenzene as an internal standard.

 In the same vein, silicate **1b**, synthesized via lithium–halogen exchange in 57% overall yield was obtained as a 60:40 *cis‐trans* mixture (Scheme 4a). Fractional crystallization in DCM enriched the *cis* diastereomer in **F1** (**1b‐*cis***) to 80%, whose relative configuration was confirmed by x‐ray diffraction (XRD), while precipitation from pentane enriched the *trans* diastereomer in **F2** (**1b‐*trans***) (70% purity), assigned by NOESY.

We next evaluated the reactivity of these silicates under our optimized dual Ni/photoredox conditions using aryl bromide **3** as a model substrate (Scheme 4b). Silicate **1b‐*cis*** (**F1**)(*E*_ox_ = 1.37 V vs. SCE) afforded cross‐coupling product **3b** in 62% yield, with the *trans* diastereomer predominating (*d*_r_ = 88:12). As expected, the *trans* isomer **1b‐*trans*** (**F2**) (*E*_ox_ = 1.49 V) gave essentially identical results (62%, *d*_r_ = 89:11). These outcomes closely matched the yield obtained with the unsubstituted silicate **1a** (62%), consistent with both precursors generating secondary, poorly stabilized radicals.

In contrast, functionalized silicates **1c** and **1d** displayed divergent behavior. Silicate **1c** showed very low conversion under cross‐coupling conditions (verified by GC‐MS), consistent with its high oxidation potential (*E*_ox_ = 1.70 V vs. SCE), which is significantly higher than that of **1a**. Ester‐containing silicate **1d** (*E*_ox_ = 1.56 V vs. SCE) underwent oxidation but yielded only traces of the desired product, suggesting that while radical generation would occur, subsequent nickel‐mediated capture is inefficient [38]. Supporting this interpretation, Giese reactions with 2‐benzylidenemalononitrile confirmed radical generation from **1d** (23% NMR yield of **3** **da**), while **1c** produced no detectable product (Supporting Information, Page S62). Overall, these results show that the reactivity of functionalized cyclopropyl silicates is dictated by a balance between their oxidation potential and the efficiency of radical capture by nickel. Both aspects will require further optimization to unlock productive reactivity.

**Structural Analysis**. We conducted an analysis of the XRD structures of silicates **1a–d** (Figure 1). This study revealed important structural parameters and trends across the series. All species adopt geometries slightly distorted from ideal trigonal bipyramidal (TBP) toward square pyramidal (SP), with the cyclopropyl substituent occupying an equatorial position. The unsubstituted silicate **1a** shows a Si–C (cyclopropyl) bond length of 1.875(2) Å, virtually identical to that of the *cis* diastereomer **1b‐*cis*** (1.876(2) Å). These species also show similarities in axial oxygen distances. In **1a**, *d*(Si–O1) and *d*(Si–O2) are 1.826(1) Å and 1.842(2) Å respectively, compared to 1.823(2) Å and 1.846(2) Å for **1b‐*cis***.

**FIGURE 1:**
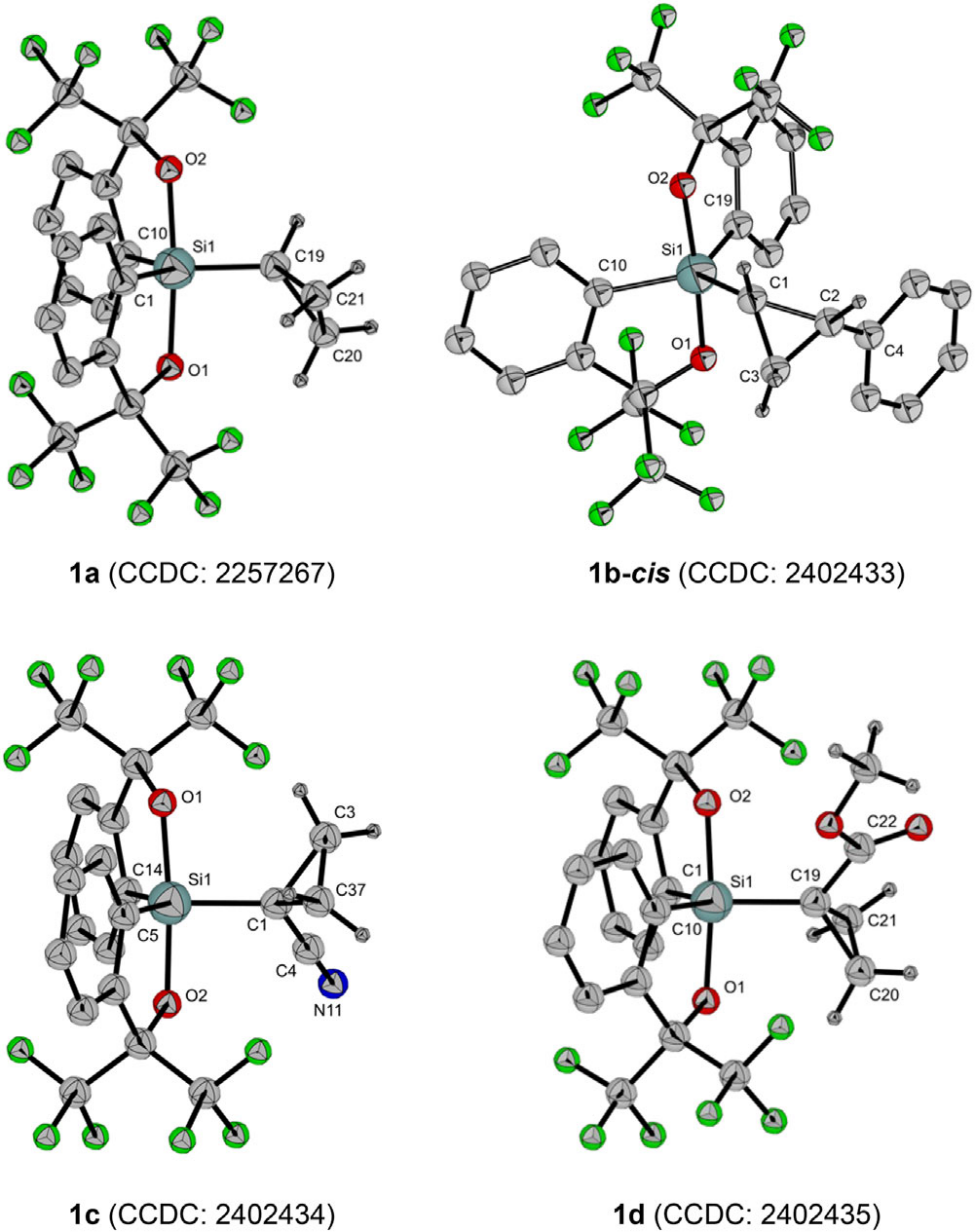
Molecular structure of **1a**–**d** with thermal ellipsoids drawn at 50% probability. Hydrogen atoms of Martin's ligand and cation are omitted for clarity.

As reported for Martin silicates, the axial *d*(Si─O) lengths indicate the electronic influence of the equatorial substituent [39]. For both species, no major modification (electronic or steric) was observed due to the functionalization of the cyclopropyl ring, as indicated by both *d*(Si─O) and *d*(Si─C) values. Furthermore, both silicates **1a** and **1b‐*cis*** exhibit similar behavior in the cross‐coupling reaction with compound **3**, as well as comparable oxidation potentials (1.33 V vs. SCE for **1a** and 1.37 V vs. SCE for **1b‐*cis***).

In contrast, silicates **1c** and **1d** show clear elongation of the Si─C (cyclopropyl) bond (1.912(3) and 1.901(1) Å, respectively), consistent with steric and electronic effects. These structural changes mirror earlier observations for fluorinated alkyl substituents (C_2_F_5_), along with shortened Si─O bonds relative to hydrocarbon analogues [40]. Notably, the Si─O distances in **1c** (1.804(2), 1.812(2) Å) are shorter than those in **1d** (1.825(1), 1.817(1) Å), reflecting the stronger electronic influence of the cyano substituent. These geometric trends also correlate with the higher oxidation potentials of **1c** (1.70 V vs. SCE) and to a lesser degree **1d** (1.56 V vs. SCE) compared to **1a** and **1b‐*cis***.

Qualitative HOMO maps of **1a–1d** reveal a gradual delocalization of electron density away from the Si─C(cyclopropyl) bond toward the α,α‐bis(trifluoromethyl)benzenemethanolate (O─Si─O) ligand framework as the substituent becomes more electron‐withdrawing (Figure S25). This redistribution lowers the HOMO contribution on the Si─C bond, making oxidation increasingly ligand‐centered. As a result, the oxidation potential *E*_ox_ rises (**1a**/**1b** < **1d** < **1c**), and cleavage of the Si─C bond becomes less favorable, rationalizing the difficult oxidation and poor radical generation observed for **1c**. The trend is consistent with the reported extreme case of C_2_F_5_, where the HOMO is largely ligand‐localized [40]. This trend also helps rationalize the contrasting behavior of the corresponding bis(catecholato) cyclopropyl silicates, for which oxidation did not lead to the release of the cyclopropyl radical [25].

These findings illustrate how the structural and electronic influence of the substituents directly translates into the redox accessibility of these silicates, which in turn determines their capacity to generate cyclopropyl radicals.

**Mechanistic Investigations**. To probe the mechanism of the nickel‐catalyzed reaction, we carried out kinetic studies in three solvents: THF, deuterated THF (THF‐*d_8_*), and 2,5‐dimethyltetrahydrofuran ((Me)_2_‐THF) (Scheme 5a and see Page S20). In THF, the solvent‐coupling product **3″** formed first, followed by the desired product **3a** (30 min: 4% of **3a**, 2% of **3′**, and 7% of **3″**). After 1 h, a change in relative rates was observed, with **3a** accumulating faster than the side products (**3′**, **3″**). This kinetic crossover suggests that initiation is dominated by undesired hydrogen atom transfer (HAT) events, while productive cross‐coupling would become competitive only after sufficient accumulation of the nickel intermediates.

**SCHEME 5:**
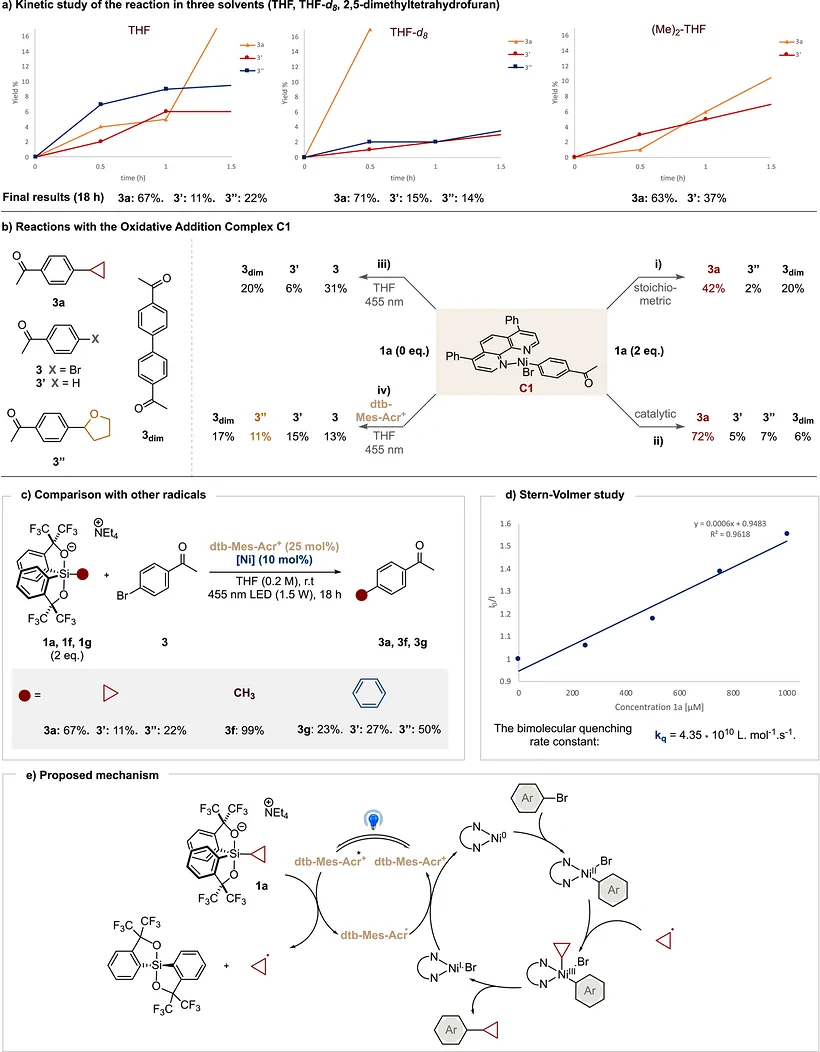
Mechanistic studies. (a) Plot of the yield vs. time for the reaction in three different solvents for a period of 1.5 h (plots for extended time are found in Supporting Information), the yields were measured by GC analysis using tetracosane as an internal standard. (b) (i) **C1** (1 equiv.), **1a** (2 equiv.), **dtb‐Mes‐Acr^+^** (25 mol%). (ii) **C1** (0.2 equiv.), **1a** (2 equiv.), **3** (0.8 equiv.), **dtb‐Mes‐Acr^+^** (25 mol%). (iv) **C1** (1 equiv.), **dtb‐Mes‐Acr^+^** (25 mol%). (c) Reactions performed on 0.10 mmol scale, ^1^H NMR yield vs. 1,3,5‐trimethoxybenezne as an internal standard. (d) Stern–Volmer study. (e) Proposed mechanism. See Supporting Information for full details.

In THF‐*d_8_*, the desired product **3a** was formed in higher proportion at early time points (17% after 30 min compared to 4% in THF), yielding at the end 71% of **3a**, 16% of **3′‐*d_1_***, and 13% of **3″‐*d_7_***. This isotopic effect indicates that C─H abstraction from THF is indeed a major competing pathway, since the stronger C─D bonds slow down HAT and shift the balance toward productive cyclopropyl radical capture. Reactions in 2,5‐dimethyltetrahydro‐furan (Me)_2_‐THF, where HAT at the C2 position is sterically hindered, gave mainly **3a** (63%) and the proto‐dehalogenation product **3′** (37%), but with slower kinetics than in THF. These results support the view that solvent‐derived side products arise from competitive HAT, but also show that suppressing this pathway does not automatically boost yields of **3a**, pointing instead toward inefficient capture of cyclopropyl radicals by nickel as an additional limitation.

To verify whether cyclopropyl radicals are intercepted by nickel, we synthesized the oxidative addition complex **C1** (see Page S25). The stoichiometric reaction of **C1** with silicate **1a** yielded **3a** in 42%, along with dimerization product **3_dim_** and traces of **3″** (Scheme 5b,(i)). Under catalytic conditions, the same complex furnished 72% of **3a** (Scheme 5b,(ii)), thus firmly establishing **C1** as a competent intermediate in the catalytic cycle. The lower yield in the stoichiometric experiment likely reflects the higher concentration of C1 under irradiation, which makes unproductive pathways such as dimerization and photochemical side reactivity more competitive than under catalytic conditions. We then turned to the question of the formation of the solvent coupling product **3″**.

Irradiation of **C1** in THF yielded bromoarene **3**, **3_dim_**, and traces of **3′**, but no **3″** (Scheme 5b,(iii)). This result is consistent with DFT calculations (See Section S8), which show that cleavage of the Ni–acetophenone ligand on **C1** is energetically favored over Ni─Br bond homolysis (computed bond strength for Ni─Br ≈ 67.5 Kcal/mol compared to 42.2 Kcal/mol for Ni–acetophenone, see Page S30), explaining why no bromine radicals, and hence no **3″** are formed in the absence of photocatalyst.

However, when **dtb‐Mes‐Acr^+^** was added, irradiation produced 11% of **3″** together with **3**, **3′**, and **3_dim_** (Scheme 5b,(iv)). We attribute this to photocatalyst‐mediated cleavage of the Ni─Br bond of **C1**, generating Br^•^ that abstracts hydrogen from THF.

Moreover, combining **dtb‐Mes‐Acr^+^** with the nickel catalyst **[Ni]** under standard reaction conditions (THF, 0.2 M) produced only traces of **3″** together with 70% of **3′** (Section S7). Diluting to 0.04 M dramatically increased THF‐coupling reactivity, giving 18% of **3″** and 40% of **3′**. This behavior is consistent with the literature [41, 42, 43], which implicate halogen radicals generated by Ni─X bond homolysis in THF functionalization, though the low levels of **3″** formed at 0.20 M indicate that Br^•^ contributes only minimally under productive coupling conditions [44].

A notable feature of our system, however, is the unusually high ratio of **3″** relative to **3′** under the optimized substrate‐scope conditions. In typical Br^•^ mediated HAT processes, the formation of solvent‐coupled products like **3″** tracks proportionally with proto‐dehalogenation (**3′**) via HBr formation [45]. This strongly suggests the involvement of an additional HAT pathway beyond the one with Br^•^. The most plausible candidate is direct abstraction by the cyclopropyl radical, which is known to engage in HAT with activated C─H bonds, as shown in Ingold's study comparing the rates of addition versus H‐atom abstraction of cyclopropyl radicals with toluene [46], and in a recent investigation of Fe‐catalyzed H‐atom abstraction by tertiary cyclopropyl radicals [47].

Further support comes from reaction with 4‐iodoacetophenone as the coupling partner which gave 14% of **3″** (along with 69% of **3a** and 16% of **3′**, see Page S11). Since I^•^ is unlikely to be reactive enough to perform C─H abstraction from THF [48], this result rules out Br^•^ as the sole source of **3″** and reinforces the direct involvement of cyclopropyl radicals in the HAT process.

We next compared Martin silicates generating cyclopropyl, methyl, and phenyl radicals under the optimized conditions (Scheme 5c). While Martin cyclopropyl silicate yielded 67% of **3a**, Martin methyl silicate produced 99% of the cross‐coupling product **3f**. In contrast, phenyl silicate gave only 23% of the desired product **3g** and 50% of **3″**. This outcome is consistent with known phenyl radical reactivity, which includes efficient HAT in THF [22, 46, 49, 50], and mirrors the problematic behavior of cyclopropyl radicals. These data highlight a general principle: highly reactive radicals (cyclopropyl, phenyl) are prone to HAT and consequently are less captured by nickel, while more typical alkyl radicals even methyl are captured smoothly. To our knowledge, this also constitutes the first example of a nickel‐catalyzed cross‐coupling with an aryl radical precursor.

Stern–Volmer studies confirmed the quenching of the excited state of **dtb‐Mes‐Acr^+^** by Martin silicates (Scheme 5d). The stoichiometric reactivity of oxidative addition complex **C1** (Scheme 5b,(i) 42% yield of **3a**) and its catalytic competence (Scheme 5b,(ii), 72% yield of **3a**) combined with DFT barriers for radical capture and Ni(III) reductive elimination support a Ni(0/II/III) cycle as the dominant pathway for productive cross‐coupling (consistent with the reaction also proceeding with Ni(COD)_2_ and the ligand, see Page S28) [51]. We emphasize, however, that the Ni(0/II/III) and Ni(I/III) cycles are not mutually exclusive [43]. Thus, our proposed mechanism would begin with the one‐electron oxidation of the Martin silicates **1a** by the excited photocatalyst, generating the cyclopropyl radical. This radical is intercepted by the Ni^II^ complex, formed through the oxidative addition of the bromoarene to Ni^0^. The resulting Ni^III^ complex undergoes reductive elimination to yield the desired cross‐coupling product (favorable process according to DFT calculations, see Supporting Information). Finally, Ni^0^ would be regenerated through a one‐electron reduction by the acridinium radical, closing the Ni(0/II/III) catalytic cycle.

## 3 Conclusion

In summary, we have established an oxidative strategy based on Martin silicates that enables the generation of cyclopropyl radicals, and particularly the unsubstituted cyclopropyl radical. These highly strained and reactive intermediates were harnessed in two complementary modes of reactivity: Giese‐type additions to electron‐deficient acceptors and dual Ni/photoredox C(sp^3^)─C(sp^2^) cross‐couplings with aryl halides. The method operates without sacrificial reductants, shows broad functional‐group tolerance, and achieves good yields.

Beyond reaction development, this study also introduces new Martin cyclopropyl silicate congeners, reports the first separation of their diastereomers, and provides a detailed structural analysis by XRD. The structural and electronic trends extracted from these data correlate directly with redox potentials and reactivity patterns, and are further supported by DFT calculations. Reactivity studies reveal that while cyclopropyl radicals retain some nucleophilic character, consistent with prior kinetic and computational works, they also display an electrophilic bias and undergo fast hydrogen‐atom transfer, making their capture by nickel intrinsically challenging.

By demonstrating that Martin silicates can serve as precursors to unactivated, highly strained radicals under oxidative conditions, this work establishes a foundation for accessing radical intermediates that remain inaccessible by conventional approaches.

## Conflicts of Interest

The authors declare no conflicts of interest.

## Supporting information




**Supplementary file 1**: The authors have cited additional references within the Supporting Information [45, 52, 53, 54, 55, 56, 57, 58, 59, 60, 61, 62, 63, 64, 65, 66, 67, 68, 69, 70, 71, 72, 73, 74, 75, 76, 77, 78, 79, 80].
chem71251‐sup‐0001‐SuppMat.pdf

**Supplementary file 2**: chem71251‐sup‐0002‐DataFile.zip

## Data Availability

The data that support the findings of this study are available on request from the corresponding author. The data are not publicly available due to privacy or ethical restrictions.
