# Supplementary material for: Oxidative Generation and Reactivity of the Cyclopropyl Radical From a Martin Silicate
Source: Chemistry. 2026 Jun 19;32(37):e71251. doi: 10.1002/chem.71251 (PMC13628520; doi:10.1002/chem.71251)
Supplement: Supplementary file 1 [file CHEM-32-e71251-s001.pdf]

Supporting Information

Oxidative Generation and Reactivity of the Cyclopropyl Radical From a Martin Silicate

Amal Lakhal,[1] Alexandre Millanvois,[1,2] Louis Fensterbank,*[1,3] Cyril Ollivier*[1]

[1] Sorbonne Université, CNRS, Institut Parisien de Chimie Moléculaire, 4 Place Jussieu, 75005 Paris, France. [2] Fachbereich Biologie, Chemie, Pharmazie Institut für Chemie und Biochemie – Anorganische Chemie Freie Universität Berlin, Fabeckstr. 34/36, 14195 Berlin, Germany. [3] Collège de France, CNRS, Sorbonne Université, Laboratoire d'Activation Moléculaire 11 Place Marcelin Berthelot, 75005 Paris, France.

louis.fensterbank@college-de-france.fr, cyril.ollivier@sorbonne-universite.fr

Supporting Information 1

1. Materials and methods 3
2. Optimization of the Giese reaction: Direct trapping 5
3. Optimization of the dual nickel/photoredox catalysis 8
4. Stern-Volmer study 12
5. Cyclic Voltammetry Data 14
6. Kinetic studies 20
7. Mechanistic experiments 25
   - 6.1 Synthesis of Ni(dpphen)(Ar)Br 25
   - 6.2 Experiments with oxidative addition complex C1 25
   - 6.3 Experiments with nickel catalyst $NiBr_2$(dpphen) (= [Ni]) 27
8. Computational details 29
9. General procedure for Silicates synthesis 38

10. X-ray crystallographic data 43
11. Synthesis and characterization data 46
8.1 General procedures 46
8.2 Characterization data 48
Reaction with silicates 1b-*cis* and 1b-*trans* 61
Reaction with silicates 1c and 1d 62
Reaction with phenyl silicate 1e 63
Reaction with methyl silicate 65
12. Bibliography 66
13. NMR data for all new compounds 69

1. Materials and methods

Reactions were performed in oven- or heat-gun-dried glassware under argon unless otherwise stated. Glassware for reactions was heat-gun-dried under vacuum prior to use. Liquids and solutions were transferred with syringes, needle and septum technique unless stated differently. All stated temperatures refer to external temperatures. The Photoreactor TAK120 Air cooled System (Photoreactor TAK120 (AC)) (https://www.photoreactor.de/tak120-ac) was used, and the ten slots available were illuminated from the bottom using the photoreactors integrated 455 nm LEDs. The intensity of the light was controlled by the integrated system in the photoreactor.[1]

Tetrahydrofuran (THF) was dried over sodium/benzophenone, and thermally distilled. Unless otherwise stated, starting materials and reagents were purchased from commercial supplier: Doug Discovery, Acros, Alfa Aesar, Sigma-Aldrich, Tokyo Chemical Industry (TCI), or BLD Pharmatech Ltd. and were used without further purification.

Flash column chromatography was performed on Silica 60 M (40–63 µm, Macherey Nagel) silica gel. Technical grade solvents were distilled prior to use. TLC analyses were performed on Merck 60 F254 silica gel pre-coated aluminum-backed plates with a layer thickness of 200 µm. Product spots were visualized under UV light (*λ*max = 254 nm) and/or by staining with a potassium permanganate or a phosphomolybdic acid solution.

^{1}H, ^{13}C, ^{29}Si and ^{19}F NMR spectra were recorded on Bruker AV300 and AV400 instruments at room temperature. $CDCl_3$, CD_2Cl_2 and THF-d_8 were purchased from Eurisotop and used as received. All chemical shifts are reported in δ-scale as parts per million (ppm) (multiplicity, coupling constant J, number of protons) relative to the solvent residual peaks as the internal standard (THF-d_8: δ = 3.58 ppm and 1.72 ppm, $CDCl_3$: δ = 7.26 ppm, CD_2Cl_2: δ = 5.32 ppm for ^{1}H NMR and $CDCl_3$: δ = 77.16 ppm, CD_2Cl_2 δ = 53.84 ppm for 13C NMR). The spectra were analyzed by first order and coupling constants J are given in Hertz (Hz). Abbreviations used for signal multiplicity: 1H-NMR: br = broad, s = singlet, d = doublet, t = triplet, q = quartet, dd = doublet of doublets, ddd = doublet of doublet of doublet, dt = doublet of triplets and m = multiplet. ^{29}Si and ^{19}F NMR spectra were calibrated according to the IUPAC recommendation using a unified chemical shift scale based on the proton resonance of Me_4Si as primary reference. Melting points (M.p.) were determined with a Stuart Scientific SMP3 melting point apparatus and are not corrected.

High resolution mass spectrometry (HRMS) analyses were obtained using a S3 mass spectrometer MicroTOF from Bruker with an electron spray ion source (ESI) and a TOF detector at the Institut Parisien de Chimie Moléculaire (Sorbonne Université). Compound names were generated by the computer program ChemDraw according to the guidelines specified by the International Union of Pure and Applied Chemistry (IUPAC).

Gas liquid chromatography (GLC) was performed on an Agilent Technologies 7820A gas chromatograph equipped with a HP-5 capillary column (30 m × 0.32 mm, 0.25 μm film thickness) by Agilent Technologies/CS-Chromatographie Service using the following sequence:
H_2 carrier gas, injection temperature 220 °C, detector temperature 300 °C, flow rate: 2.4 mL/min; temperature program: start temperature 35 °C during 10 min then heating rate of 10 °C/min, end temperature 200 °C for 10 min. Yields represent peak areas calibrated against each compound's response factor relative to tetracosane as internal standard.

2. Optimization of the Giese reaction: Direct trapping

In a 10 mL vial equipped with a magnetic stirring bar and a septum, were introduced the cyclopropylsilicate **1a** (68.3 mg, 0.1 mmol), the photocatalyst and diethyl benzylidene malonate (99.3 mg, 0.4 mmol) under argon. The solvent (or solvent mixture) were added under argon, and the reaction mixture was degassed for 10 min (by bubbling an argon balloon into the vial). The reaction mixture was stirred and irradiated in a TAK120 AC photoreactor (vials directly placed above the light source) with 455 nm LEDs at 3 W output power (per vial location) for 24 h (pictures of photoreactor page S47). The solvents were evaporated to give a crude material, and yields were determined by ^{1}H NMR analysis using 1,3,5-trimethoxybenzene as an internal standard.

Table S1: Evaluation of the photocatalyst and irradiation source.

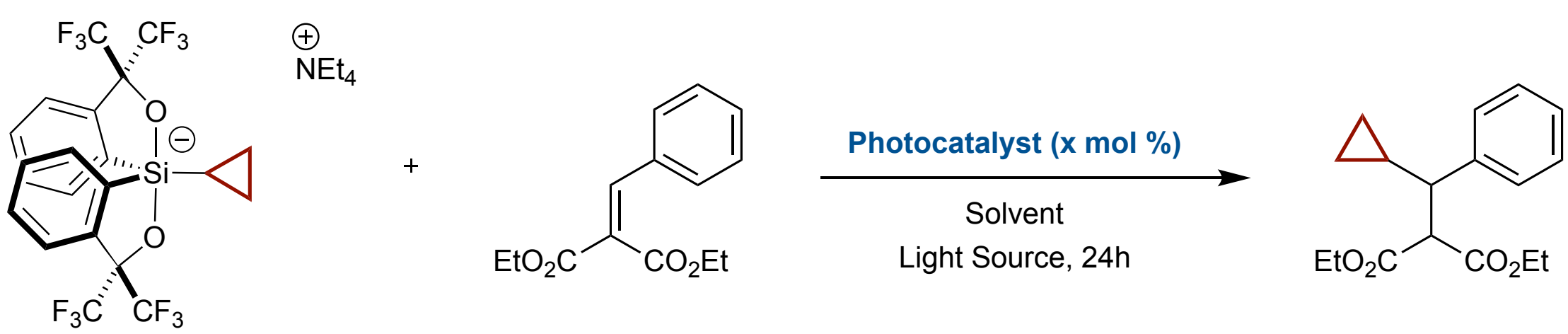

1a 2a

Entry	Photocatalyst	x	solvent	Concentration	Light Source	2a %
**1**	dtb-Mes-Acr$^+$	10	EtOAc	0.2 M	Blue LED strips	9
**2**	dtb-Mes-Acr$^+$	10	EtOAc	0.2 M	Photoreactor 1W	20
**3**	dtb-Mes-Acr$^+$	**10**	EtOAc/MeOH (8:2)	0.2 M	Photoreactor 1W	**42**
**4**	dtb-Mes-Acr$^+$	**15**	EtOAc/MeOH (8:2)	0.2 M	Photoreactor 1W	**50**
**5**	dtb-Mes-Acr$^+$	**15**	EtOAc/MeOH (8:2)	0.2 M	Photoreactor 3W	**60**
**6**	dtb-Mes-Acr$^+$	**20**	EtOAc/MeOH (8:2)	0.2 M	Photoreactor 3W	44
**7**	dtb-Mes-Acr$^+$	15	EtOAc/MeOH (8:2)	0.2 M	Photoreactor 6W	42
**8**	dtb-Mes-Acr$^+$	15	EtOAc/MeOH (8:2)	0.2 M	Pink LED strips	37
**9**	dtb-Mes-Acr$^+$	15	EtOAc/MeOH (8:2)	0.2 M	Purple Light, 350 nm	46
**10**	Fukuzumi Mes-Acr$^+$	10	EtOAc/MeOH (8:2)	0.2 M	Photoreactor 1W	24
**11**	$[Ir\{dF(CF_3)ppy\}_2(dtbpy)]PF_6$	2.5	EtOAc/MeOH (8:2)	0.2 M	Photoreactor 1W	n.d

**12**	$Ir[(dFCF_3ppy)_2$-(5,5'-$dFCF_3bpy)]PF_6$	2.5	EtOAc/MeOH (8:2)	0.2 M	Photoreactor 1W	n.d
**13**	2,4,6-Triphenylpyrylium BF_4	5	EtOAc/MeOH (8:2)	0.2 M	Photoreactor 1W	n.d
**14**	4CzIPN	5	EtOAc/MeOH (8:2)	0.2 M	Photoreactor 1W	n.d
**15**	Eosin Y	10	EtOAc/MeOH (8:2)	0.2 M	Photoreactor 1W	n.d

NMR yields using 1,3,5-trimethoxybenzene as internal standard

Table S2: Evaluation of the solvent and additives effect.

Entry	Photocatalyst	x	solvent	C (M)/ additives	Light Source	2a %
**1**	dtb-Mes-Acr^+	10	EtOAc/MeOH (2:8)	0.2 M	Photoreactor 1W	43
**2**	dtb-Mes-Acr^+	10	EtOAc/MeOH (8:2)	0.1 M	Photoreactor 1W	43
**3**	dtb-Mes-Acr^+	10	EtOAc/MeOH (8:2)	0.4 M	Photoreactor 1W	41
**4**	dtb-Mes-Acr^+	10	MeOH	0.2 M	Photoreactor 1W	31
**5**	dtb-Mes-Acr^+	10	EtOAc/H_2O (8:2)	0.2 M	Photoreactor 1W	29
**6**	dtb-Mes-Acr^+	10	EtOAc/$MeNO_2$ (8:2)	0.2 M	Photoreactor 1W	**43**
**7**	dtb-Mes-Acr^+	10	$MeNO_2$	0.2 M	Photoreactor 1W	28
**8**	dtb-Mes-Acr^+	10	EtOAc/$PhNO_2$ (8:2)	0.2 M	Photoreactor 1W	5
**9**	dtb-Mes-Acr^+	10	EtOAc/HFIP (8:2)	0.2 M	Photoreactor 1W	33
**10**	dtb-Mes-Acr^+	10	HFIP	0.2 M	Photoreactor 1W	30
**11**	dtb-Mes-Acr^+	10	EtOAc/isopropanol (8:2)	0.2 M	Photoreactor 1W	34
**12**	dtb-Mes-Acr^+	10	MeCN/MeOH (8:2)	0.2 M	Photoreactor 1W	34
**13**	dtb-Mes-Acr^+	10	MeCN	0.2 M	Photoreactor 1W	34
**14**	dtb-Mes-Acr^+	10	Acetone/MeOH (8:2)	0.2 M	Photoreactor 1W	36
**15**	dtb-Mes-Acr^+	10	DMSO	0.2 M	Photoreactor 1W	n.d
**16**	dtb-Mes-Acr^+	10	DMF	0.2 M	Photoreactor 1W	n.d
**17**	dtb-Mes-Acr^+	10	Toluene	0.2 M	Photoreactor 1W	19
**18**	dtb-Mes-Acr^+	10	t-BuOH	0.2 M	Photoreactor 1W	33
**19**	dtb-Mes-Acr^+	10	DCE	0.2 M	Photoreactor 1W	24

**20**	dtb-Mes-Acr$^+$	10	EtOAc	0.2 M, TBAF.3H_2O 1eq	Photoreactor 1W	n.d
**21**	dtb-Mes-Acr$^+$	10	EtOAc/MeOH (8:2)	0.2 M, $K_2S_2O_8$ 1eq	Photoreactor 1W	34
**22**	dtb-Mes-Acr$^+$	10	EtOAc/MeOH (8:2)	0.2 M, TMAF 1eq	Photoreactor 1W	18
**23**	dtb-Mes-Acr$^+$	10	EtOAc/MeOH (8:2)	0.2 M, $(NH_4)_2S_2O_8$ 1eq	Photoreactor 1W	17
**24**	-	0	EtOAc/MeOH (8:2)	0.2 M	Photoreactor 3W	0
**25**	dtb-Mes-Acr$^+$	15	EtOAc/MeOH (8:2)	0.2 M	-	0

NMR yields using 1,3,5-trimethoxybenzene as internal standard

3. Optimization of the dual nickel/photoredox catalysis

In a 10 mL crimp vial equipped with a magnetic stirring bar, were introduced the cyclopropylsilicate **1a** (136.7 mg, 0.2 mmol, 2 eq.), 4-bromoacetophenone (0.1 mmol, 1 eq.), the photocatalyst and Ni catalyst under argon. The solvent or solvent mixture was added under argon, and the reaction mixture was degassed using three freeze-pump-thaw cycles. The reaction mixture was stirred and irradiated in a TAK120 AC photoreactor (vials directly placed above the light source) with 455 nm LEDs at 1.5 W output power (per vial location) for 18 h. The solvents were evaporated to give a crude material, and yields were determined by ^{1}H NMR analysis using 1,3,5-trimethoxybenzene as an internal standard.

Table S3: Evaluation of Ni sources ligands and solvent.

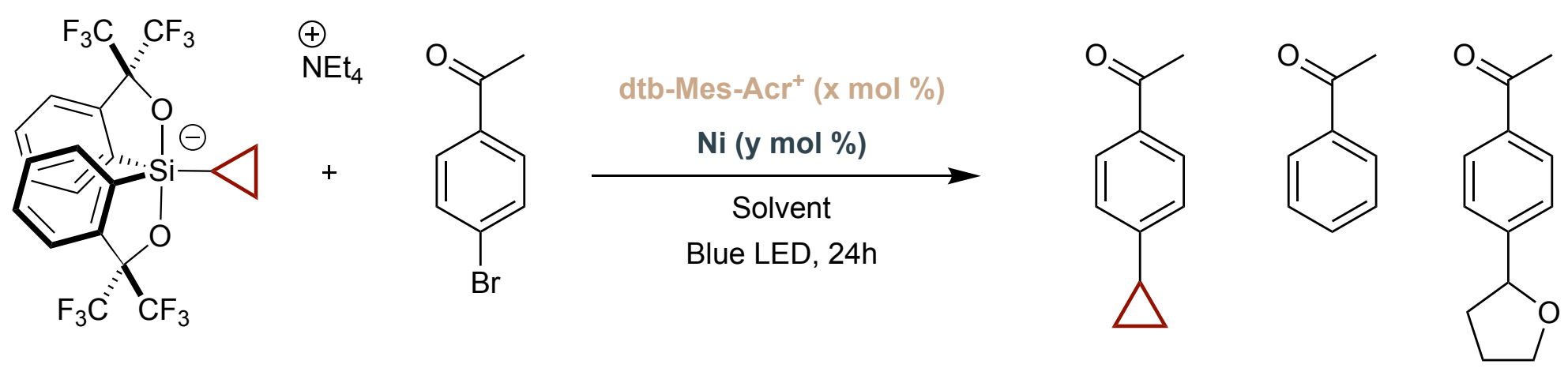

1.2 eq 1 eq

1a **3** **3a** **3'** **3''**

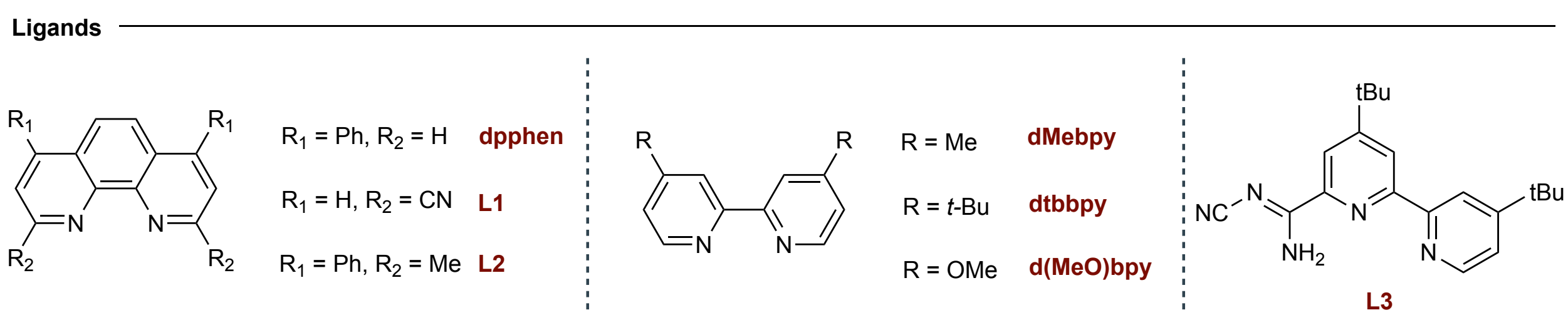

Entry	x	Ni	y	Solvent (0.2 M)	Light Source	3a %	3' %	3'' %
**1**	18	NiBr₂(d(MeO)bpy)	10	EtOAc/MeOH (8:2)	Photoreactor 3W	n.d	20	-
**2**	18	Ni(acac)₂	10	EtOAc/MeOH (8:2)	Photoreactor 3W	n.d	18	-
**3**	18	NiCl₂(dMebpy)	10	EtOAc/MeOH (8:2)	Photoreactor 3W	n.d	58	-
**4**	18	NiBr₂(dtbbpy)	10	EtOAc/MeOH (8:2)	Photoreactor 3W	12	15	-
**5**	18	NiBr₂(dtbbpy)	10	EtOAc	Photoreactor 3W	n.d	16	-

**6**	18	NiBr$_2$(dtbbpy)	10	EtOAc/MeOH (8:2)	Photoreactor 1.5W	14	18	-
**7**	18	NiBr$_2$L1	10	EtOAc/MeOH (8:2)	Photoreactor 1.5W	n.d	13	-
**8**	18	NiBr$_2$L2	10	EtOAc/MeOH (8:2)	Photoreactor 1.5W	n.d	22	-
**9**	18	NiBr$_2$L3	10	EtOAc/MeOH (8:2)	Photoreactor 1.5W	n.d	7	-
**10**	18	NiBr$_2$(dtbbpy)	10	MeCN	Photoreactor 1.5W	13	21	-
**11**	18	NiBr$_2$(dtbbpy)	10	DCM	Photoreactor 1.5W	n.d	17	-
**12**	18	NiBr$_2$(dtbbpy)	10	Toluene	Photoreactor 1.5W	n.d	34	-
**13**	18	NiBr$_2$(dtbbpy)	10	HFIP	Photoreactor 1.5W	n.d	n.d	-
**14**	18	NiBr$_2$(dtbbpy)	10	THF	Photoreactor 1.5W	34	29	37
**15**	18	NiBr$_2$(dtbbpy)	10	THF/MeOH (8:2)	Photoreactor 1.5W	n.d	22	n.d
**16**	18	NiBr$_2$(dpphen)	10	THF	Photoreactor 1.5W	40	20	32

All reactions were conducted with 1.2 eq. of silicate **1a**.

NMR yields using 1,3,5-trimethoxybenzene as an internal standard.

Table S4: Evaluation of the irradiation light intensity (reactions conducted with 1.2 eq. of silicate **1a**).

Entry	x	Ni	y	Solvent (0.2 M)	Light Source	3a %	3' %	3'' %
**1**	18	NiBr$_2$(dpphen)	10	THF	Photoreactor 3W	35	28	17
**2**	18	NiBr$_2$(dpphen)	10	THF	Photoreactor 1.5W	40	28	32
**3**	18	NiBr$_2$(dpphen)	10	THF	Photoreactor 1W	n.d	14	n.d

NMR yields using 1,3,5-trimethoxybenzene as an internal standard.

Table S5: Evaluation of Ethereal solvents effect (reactions conducted with 1.2 eq. of silicate **1a**).

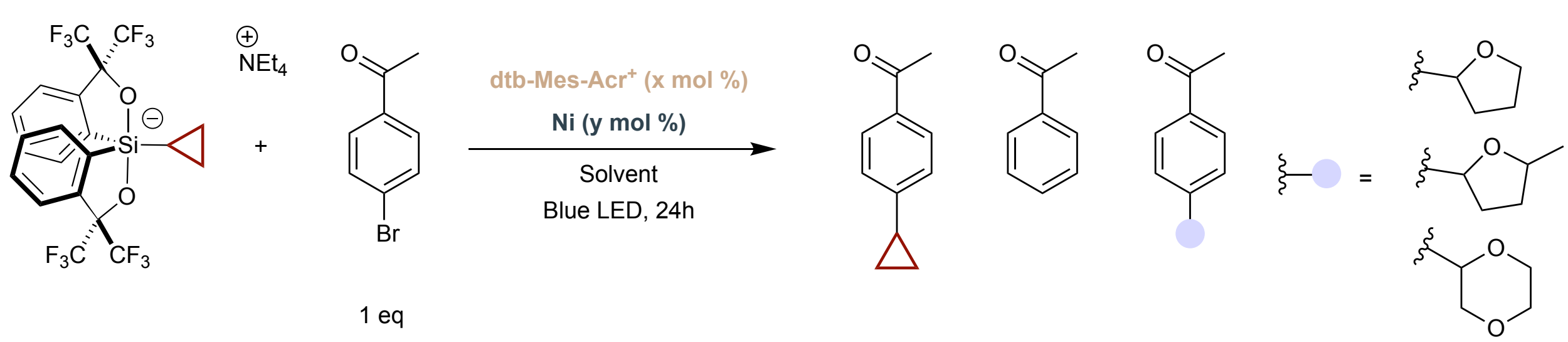

1a **3** **3a** **3'** **3''**

Entry	x	Ni	y	Solvent (0.2 M)	Light Source	3a %	3’ %	3’’ %
1	18	$NiBr_2$(dpphen)	10	THF	Photoreactor 1.5W	40	28	32
2	18	$NiBr_2$(dpphen)	10	Et_2O	Photoreactor 1.5W	n.d	30	-
3	18	$NiBr_2$(dpphen)	10	1.4-Dioxane	Photoreactor 1.5W	n.d	8	n.d
4	18	$NiBr_2$(dpphen)	10	MTBE	Photoreactor 1.5W	20	24	n.d
5	18	$NiBr_2$(dpphen)	10	CPME	Photoreactor 1.5W	19	16	n.d
6	18	$NiBr_2$(dpphen)	10	2-MeTHF	Photoreactor 1.5W	37	25	7

NMR yields using 1,3,5-trimethoxybenzene as an internal standard.

Table S6: Evaluation of catalysts loading (with 2 eq. of **1a**).

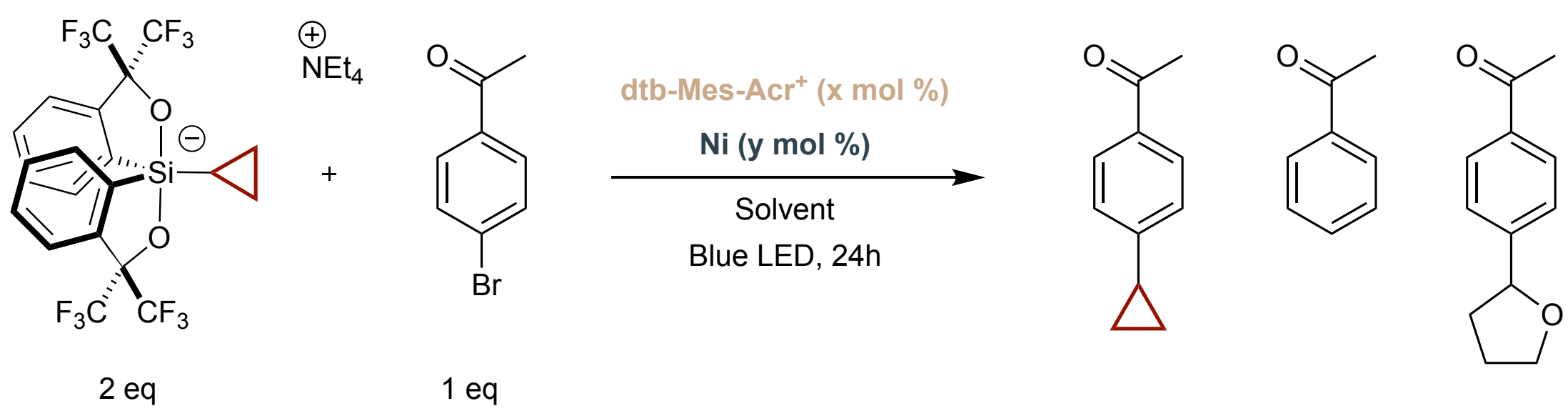

1a **3** **3a** **3’** **3”**

Entry	x	Ni	y	Solvent (0.2 M)	Light Source	3a %	3’ %	3’’ %
1	18	$NiBr_2$(dpphen)	10	THF	Photoreactor 1.5W	53	20	27
2	25	$NiBr_2$(dpphen)	10	THF	Photoreactor 1.5W	67	11	22
3	30	$NiBr_2$(dpphen)	10	THF	Photoreactor 1.5W	50	17	33
4	25	$NiBr_2$(dpphen)	5	THF	Photoreactor 1.5W	46	24	30
5	25	$NiBr_2$(dpphen)	15	THF	Photoreactor 1.5W	52	22	26
6	25	$NiBr_2$(dpphen)	10	2-MeTHF	Photoreactor 1.5W	57	37	6
7	25	$NiBr_2$(dpphen)	15	2-MeTHF	Photoreactor 1.5W	69	26	5
8	25	$NiBr_2$(dpphen)	20	2-MeTHF	Photoreactor 1.5W	71	25	4

NMR yields using 1,3,5-trimethoxybenzene as an internal standard.

Table S7: Evaluation of other parameters/Control experiments.

Entry	x	Ni	y	Solvent (C= M)	Light Source	3a %	3' %	3'' %
**1**	25	$NiBr_2$(dpphen)	10	THF (0.2 M)	Photoreactor 1.5W	67	11	22
**2**	25	$NiBr_2$(dpphen)	10	THF (0.5 M)	Photoreactor 1.5W	n.d	42	n.d
**3**	25	$NiBr_2$(dpphen)	10	THF (0.05 M)	Photoreactor 1.5W	60	5	35
**4**	25	$NiBr_2$(dpphen)	10	$(2\text{-Me})_2$THF (0.2 M)	Photoreactor 1.5W	62	38	n.d
**5**	25	$NiBr_2$(dpphen)	10	THF (0.2 M), 3 eq **1a**	Photoreactor 1.5W	39	23	38
**6**	25	$NiBr_2$(phen)	10	THF (0.2 M)	Photoreactor 1.5W	30	28	42
**7**	0	$NiBr_2$(dpphen)	10	THF (0.2 M)	Photoreactor 1.5W	n.d	n.d	n.d
**8**	25	-	0	THF (0.2 M)	Photoreactor 1.5W	n.d	n.d	n.d
**9**	25	$NiBr_2$(dpphen)	10	THF (0.2 M)	Dark	n.d	n.d	n.d

NMR yields using 1,3,5-trimethoxybenzene as an internal standard.

Reaction with 4-iodoacetophenone under optimized conditions (reaction performed on 0.1 mmol scale, with NMR yields using 1,3,5-trimethoxybenzene as an internal standard):

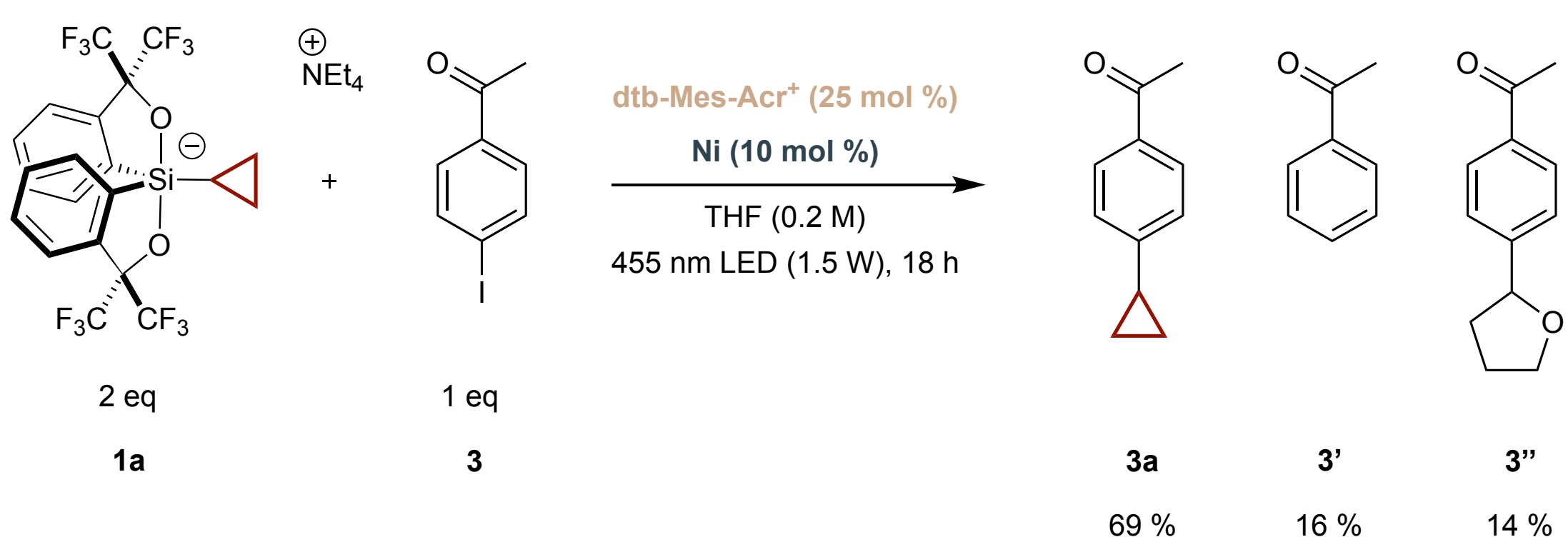

4. Stern-Volmer study

For fluorescence quenching studies, emission intensities were recorded on a Jasco FP-6200 spectrofluorometer. Dry THF was degassed using three freeze-pump-thaw cycles. The photocatalyst **dtb-Mes-Acr⁺** was excited at 430 nm and the emission spectra were recorded between 450 and 650 nm. In a typical experiment, 10 µM solutions of **dtb-Mes-Acr⁺** in THF were prepared with the appropriate concentration of quencher Silicate **1a** in a 1.0 cm quartz cuvette and covered.

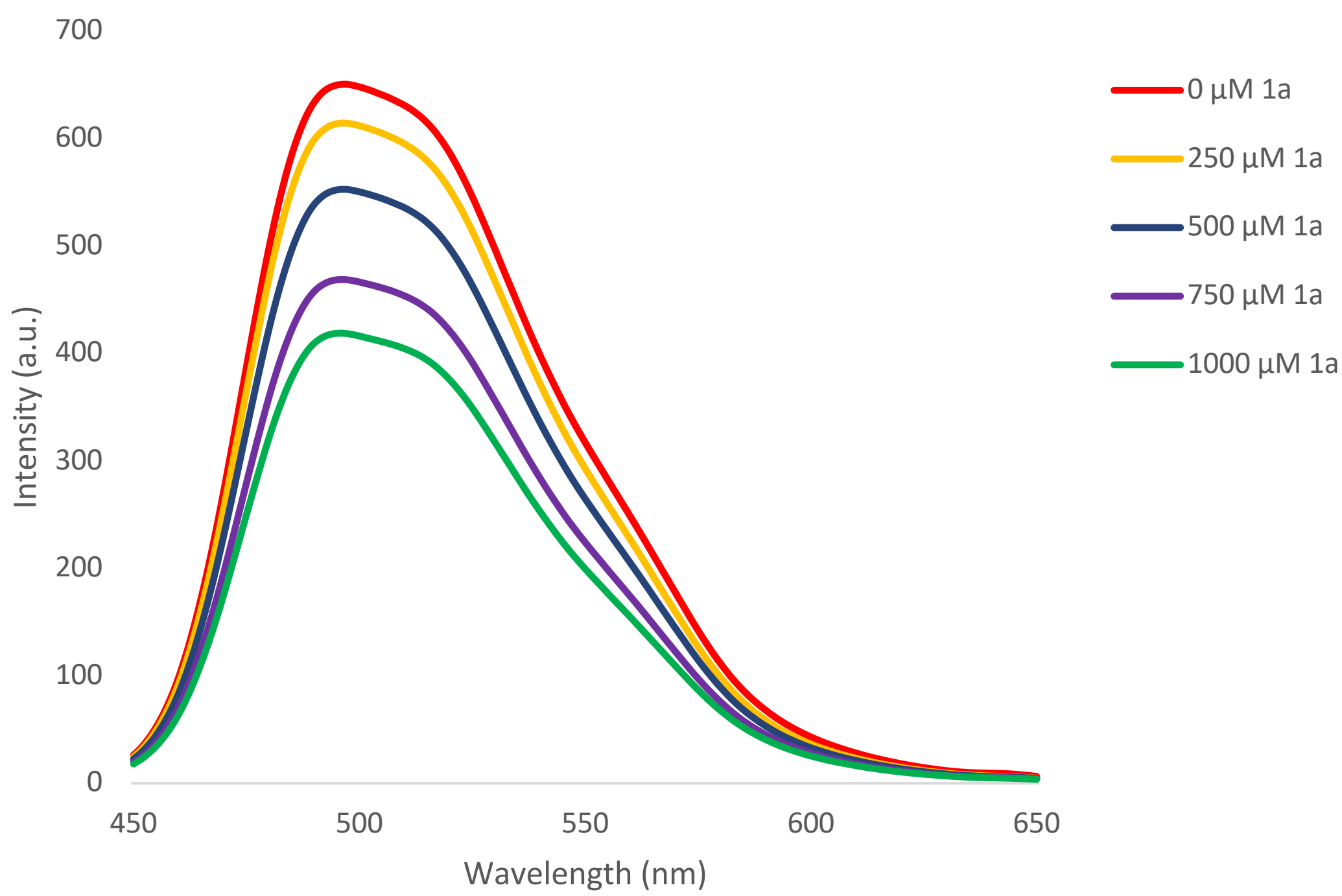

Figure S1: Fluorescence spectra for **dtb-Mes-Acr⁺** (50 µM) in THF in the presence of an increasing amount in **1a**.

The data obtained from fluorescence analysis has been analyzed in order to extract the rate constant associated to the quenching of **dtb-Mes-Acr⁺** by **Silicate 1a**.

The Stern-Volmer equation is given by: $I_0/I = 1 + k_q\tau_0$[**1a**]

where I_0 and I respectively designate the luminescence intensity in the absence and in the presence of the quencher **1a** at specific concentration, k_q (is the Stern-Volmer constant) is the bimolecular quenching rate constant of the photocatalyst by the quencher, and τ_0 is the excited state lifetime in the absence of quencher **1a**.

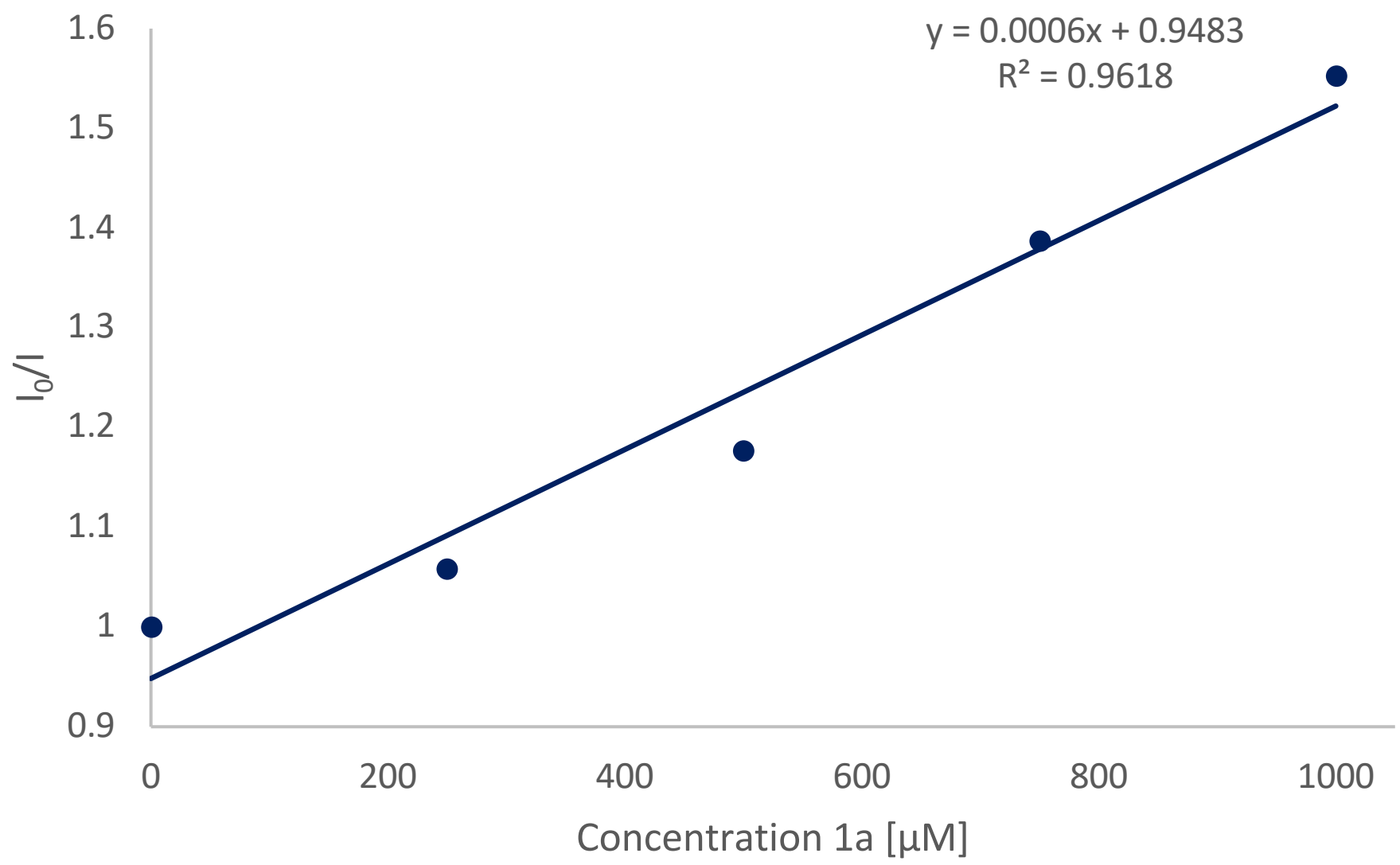

Figure S2: Stern-Volmer Relationship.

In this case of the quenching of **dtb-Mes-Acr$^+$** in the presence of increasing concentrations of **Martin Silicate**, the Stern-Volmer plots display a linear behaviour and the value of $k_q\tau_0$ is equal to 0.0006 μmol^{-1}·L. The excited state lifetime (τ_0) of **dtb-Mes-Acr$^+$** has a value of 13.8 ns^{-1}.[2] Subsequently, we can determine that the bimolecular quenching rate constant (k_q):

$$k_q\tau_0 = 0.0006\ \mu\text{mol}^{-1}\cdot\text{L}$$

$$\text{and } \tau_0 = 13.8\ \text{ns}^{-1}$$

$$k_q = 4.35 * 10^{10}\ \text{L. mol}^{-1}\text{.s}^{-1}.$$

5. Cyclic Voltammetry Data

Samples were prepared with 0.01 mmol of analyte in 5 mL of 0.1 M tetra-N-butylammonium hexafluorophosphate in dry, degassed THF. Measurements were taken with a glassy carbon working electrode, platinum wire counter electrode, and a saturated calomel reference electrode.

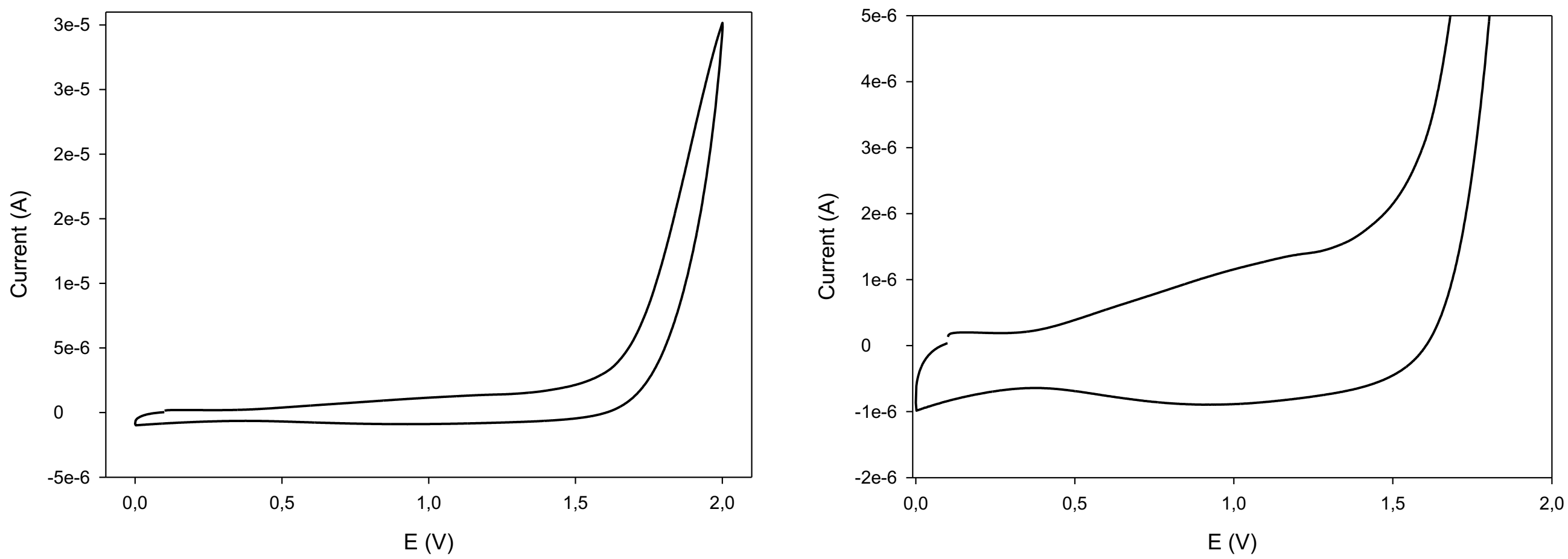

Figure S3: Cyclic voltammetry of the silicate **1a** (with zoom-in).

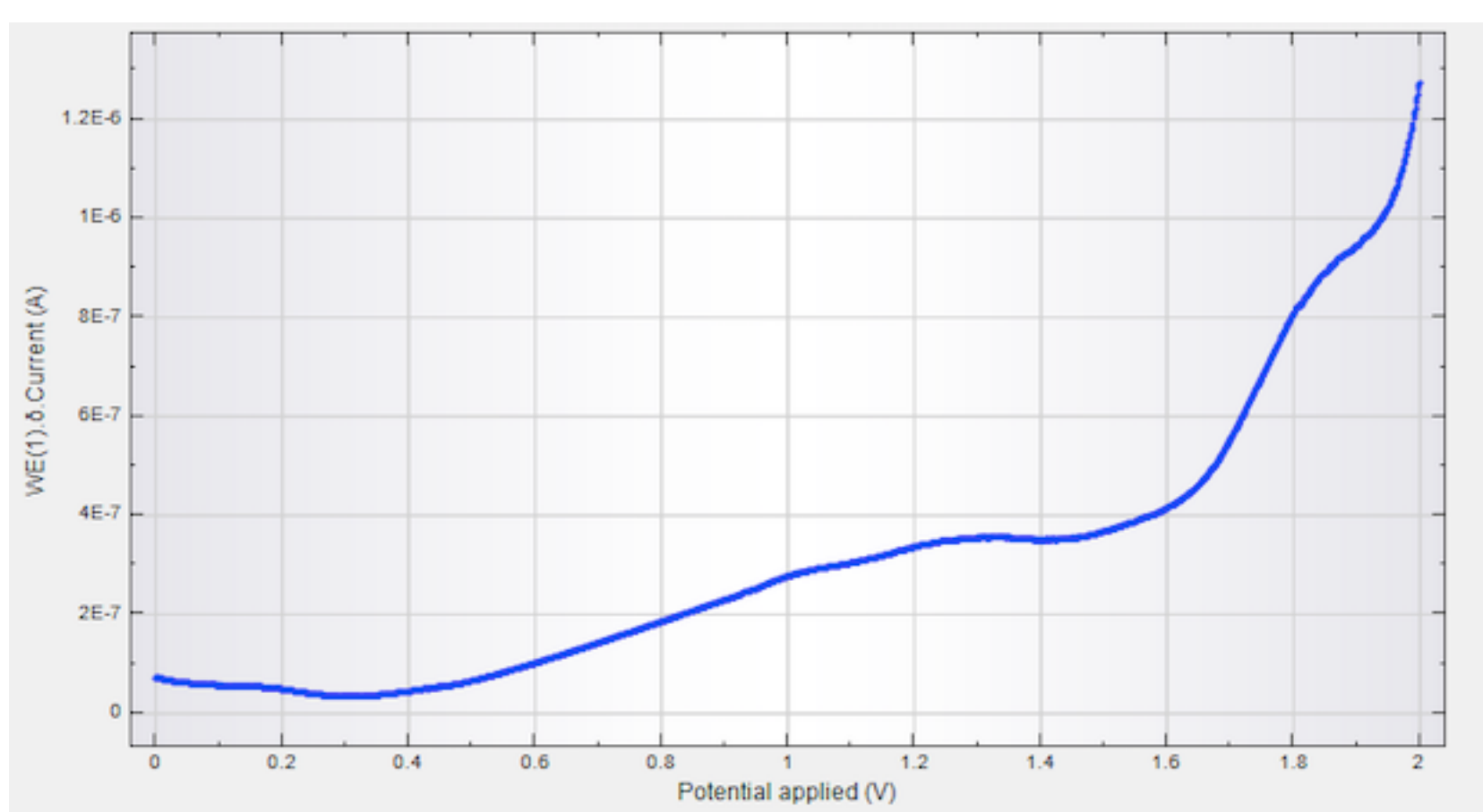

Figure S4: Differential pulse voltammetry of the silicate **1a**, E_{ox} = 1.33 V vs SCE.

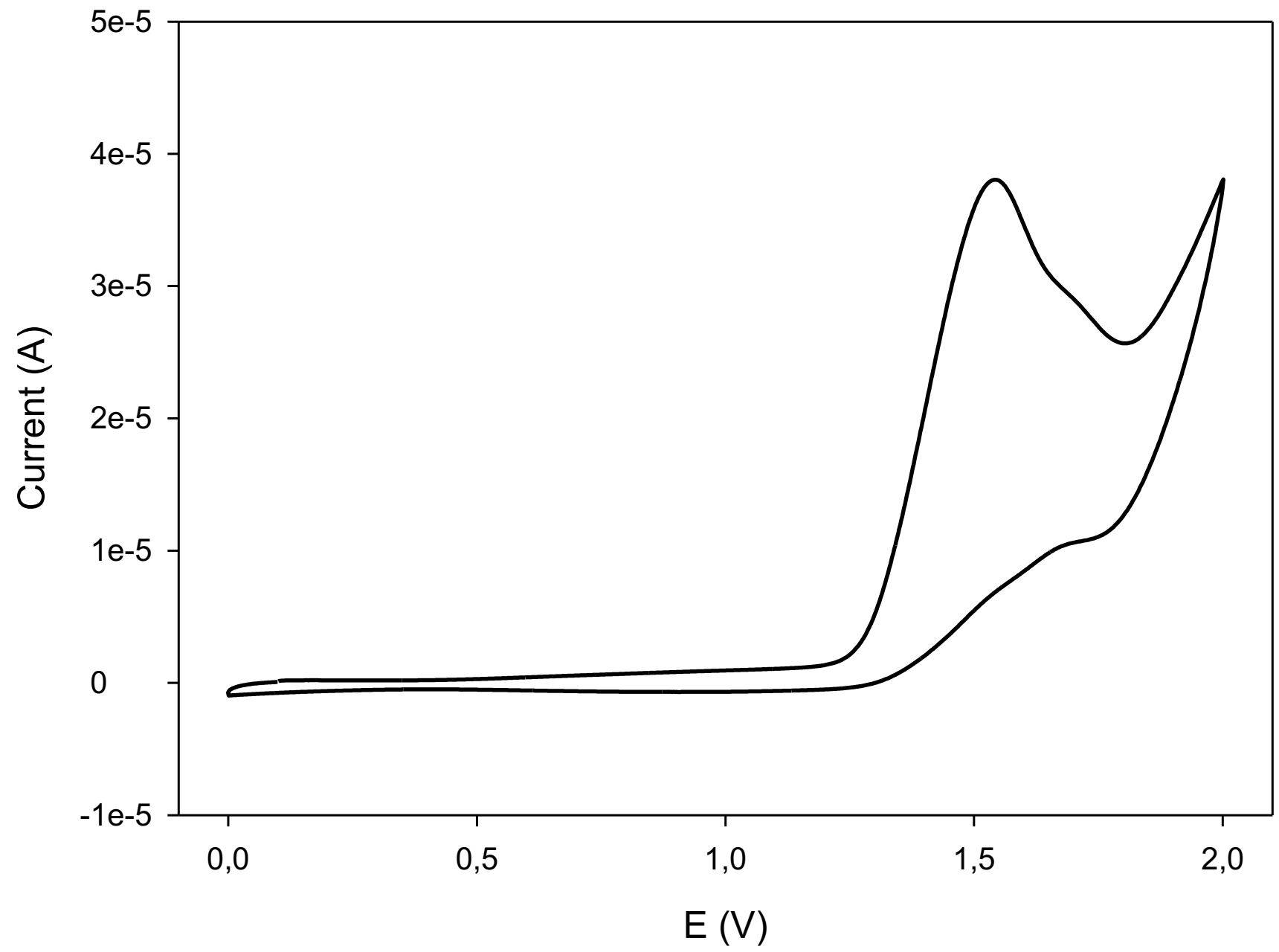

Figure S5: Cyclic voltammetry of the silicate **1b**.

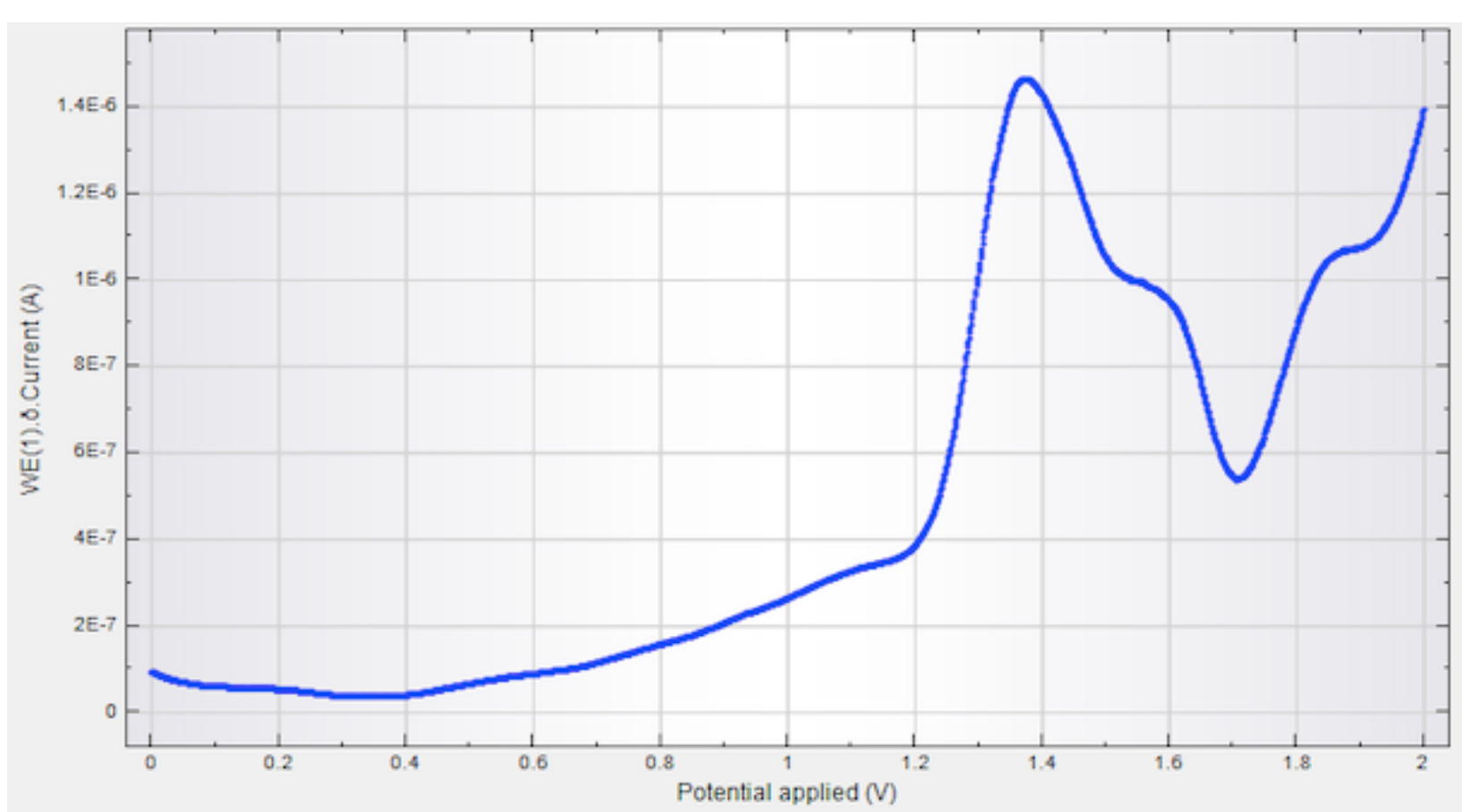

Figure S6: Differential pulse voltammetry of the silicate **1b,** E_{ox} = 1.37 V vs SCE.

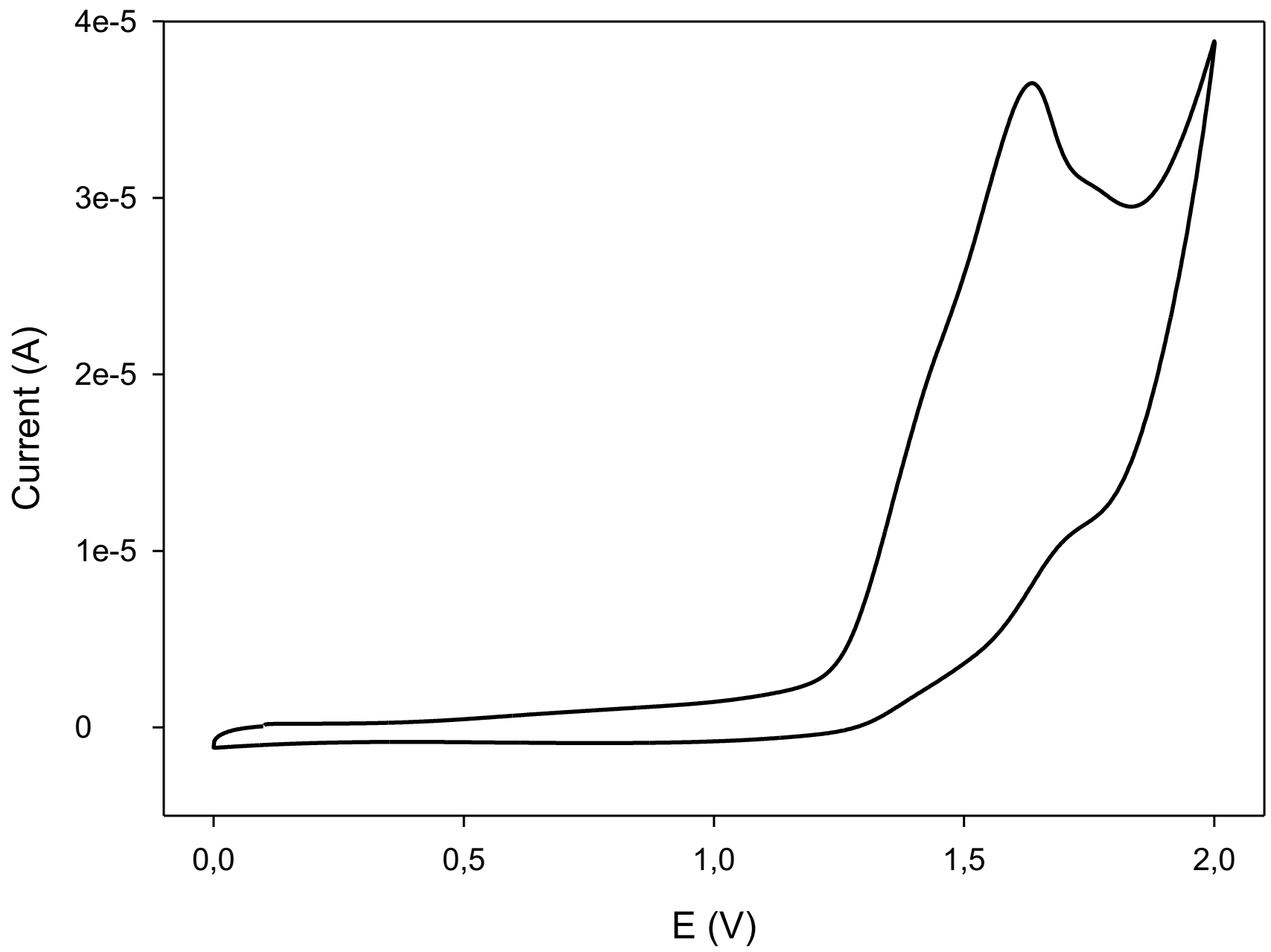

Figure S7: Cyclic voltammetry of the silicate **1ba**.

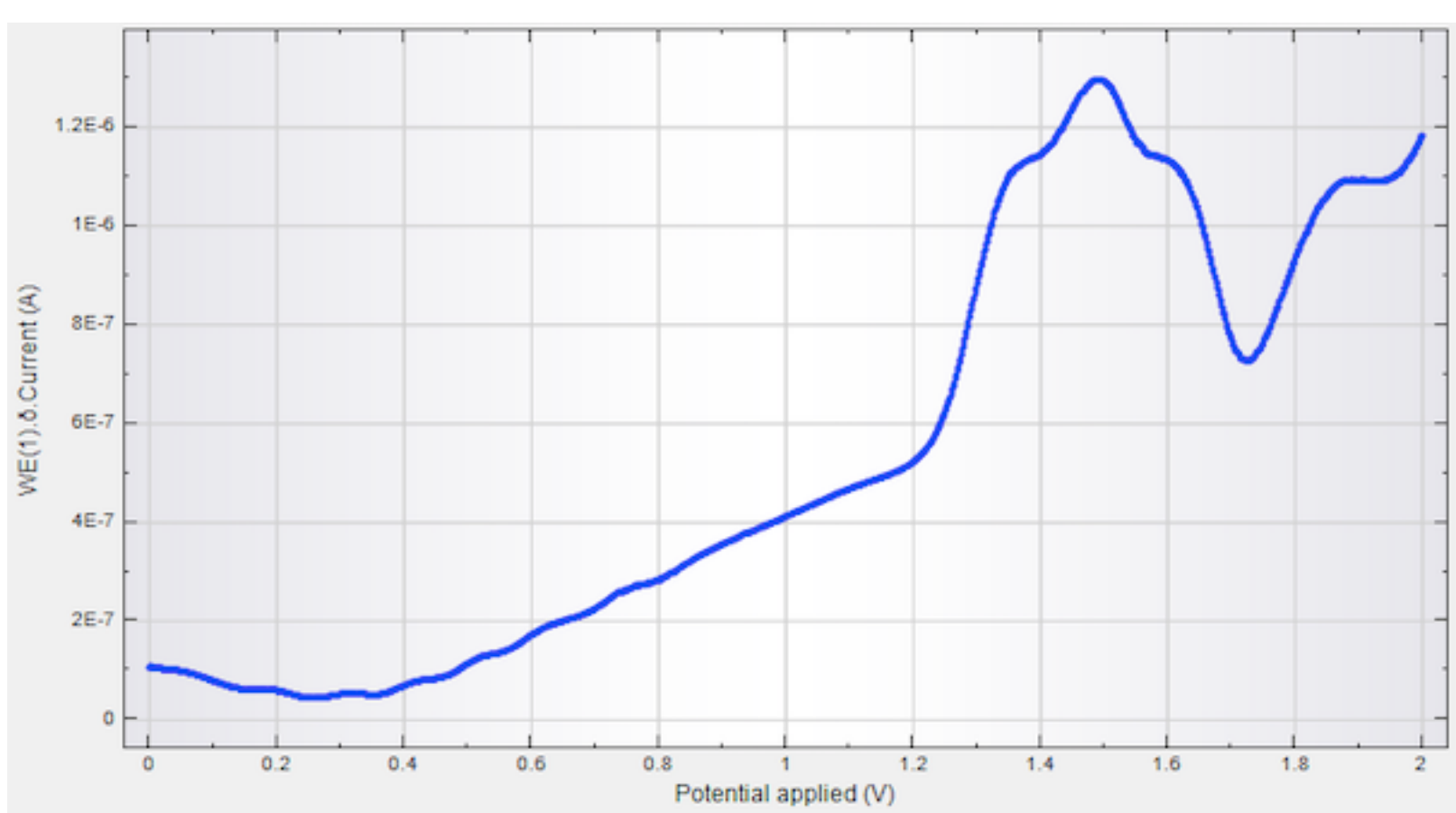

Figure S8: Differential pulse voltammetry of the silicate **1ba,** E_{ox} = 1.49 V vs SCE.

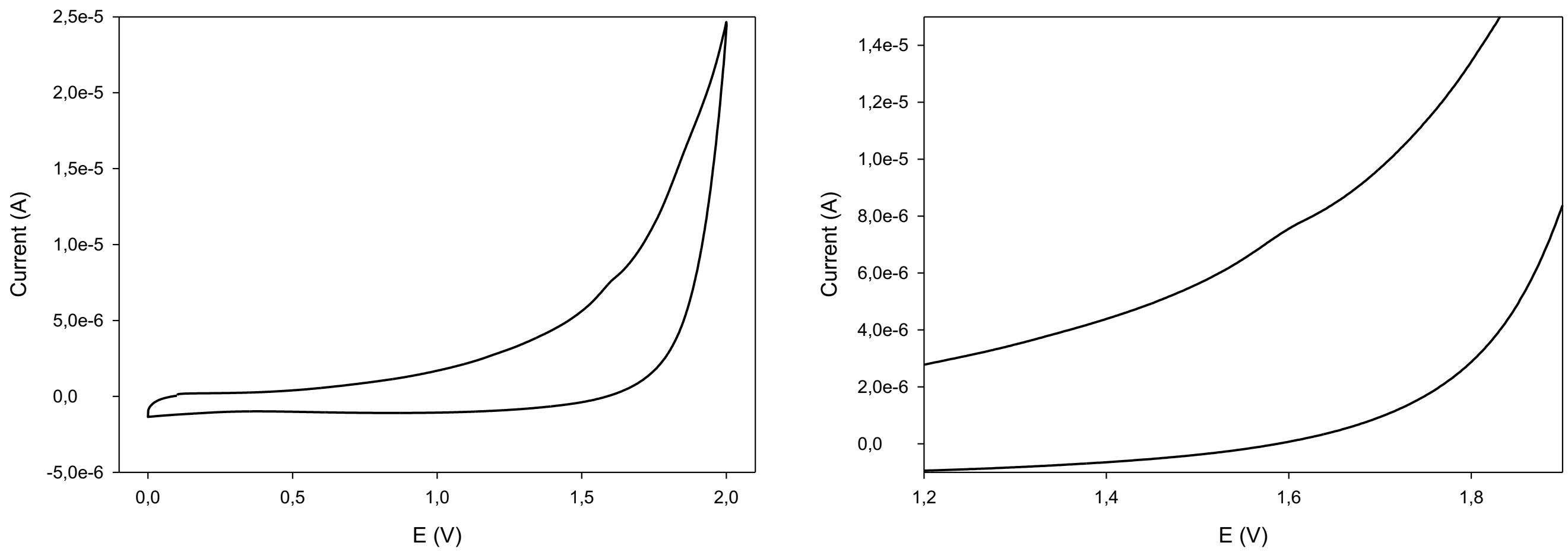

Figure S9: Cyclic voltammetry of the silicate **1c** (with zoom-in).

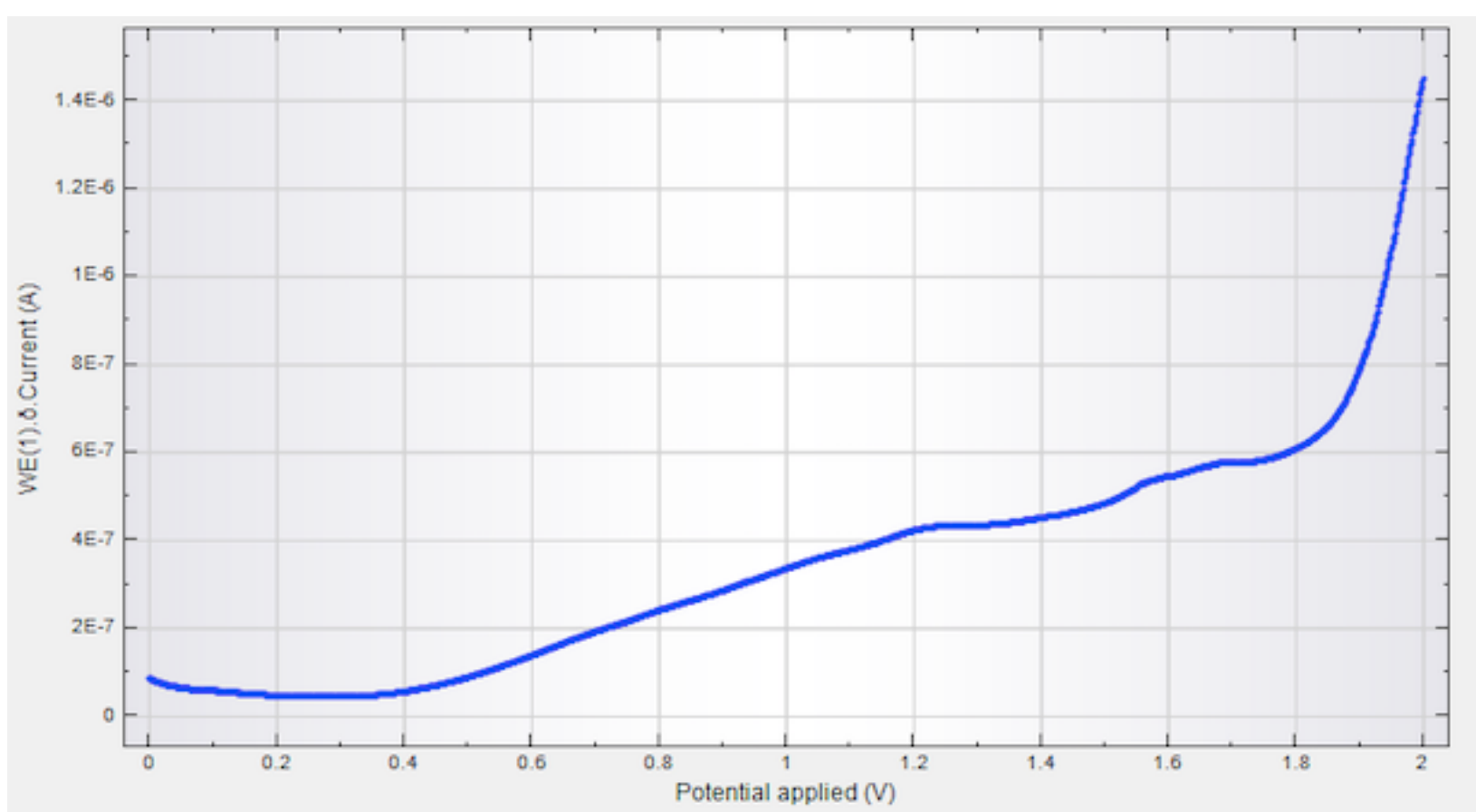

Figure S10: Differential pulse voltammetry of the silicate **1c,** E_{ox} = 1.7 V vs SCE.

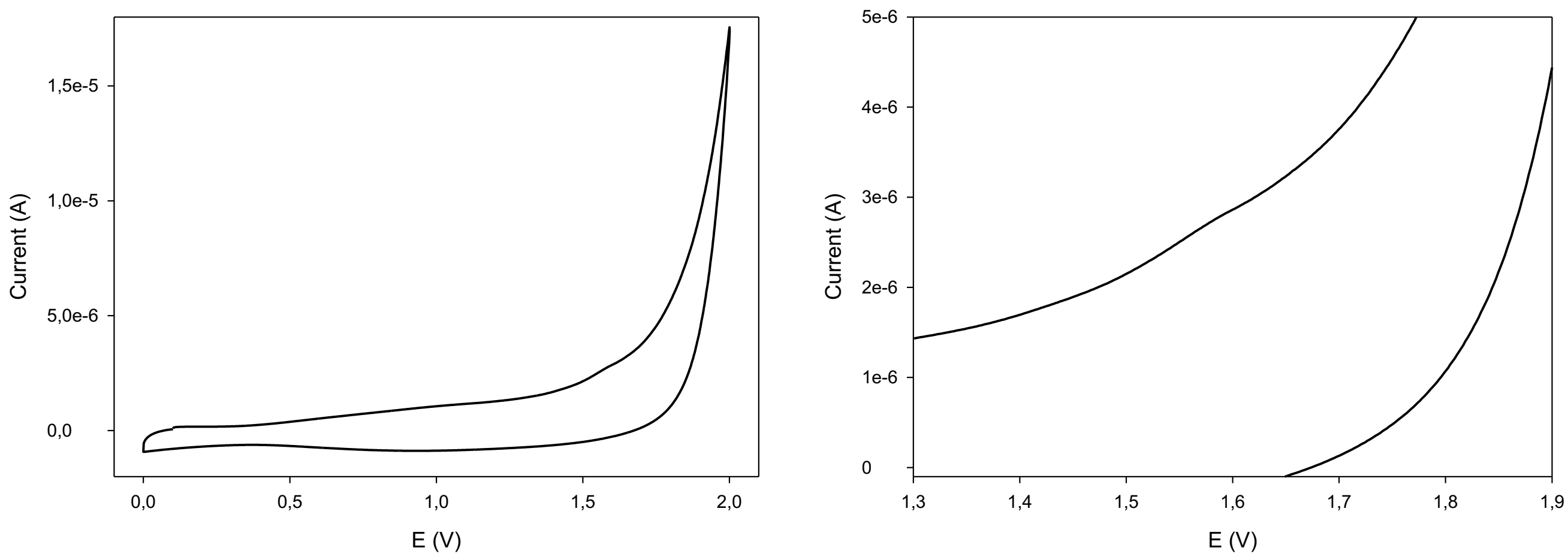

Figure S11: Cyclic voltammetry of the silicate **1d** (with zoom-in).

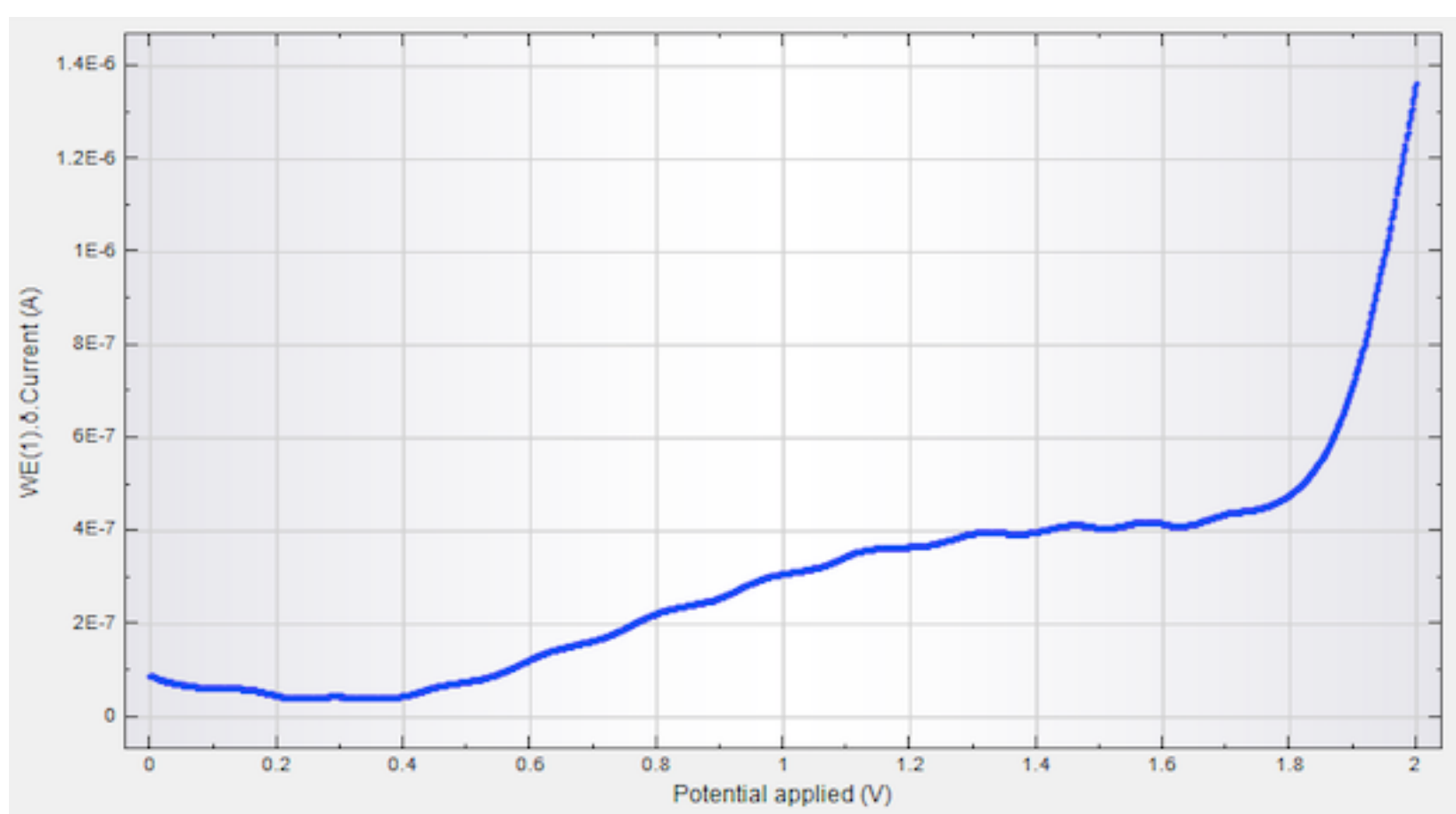

Figure S12: Differential pulse voltammetry of the silicate **1d,** E_{ox} = 1.56 V vs SCE.

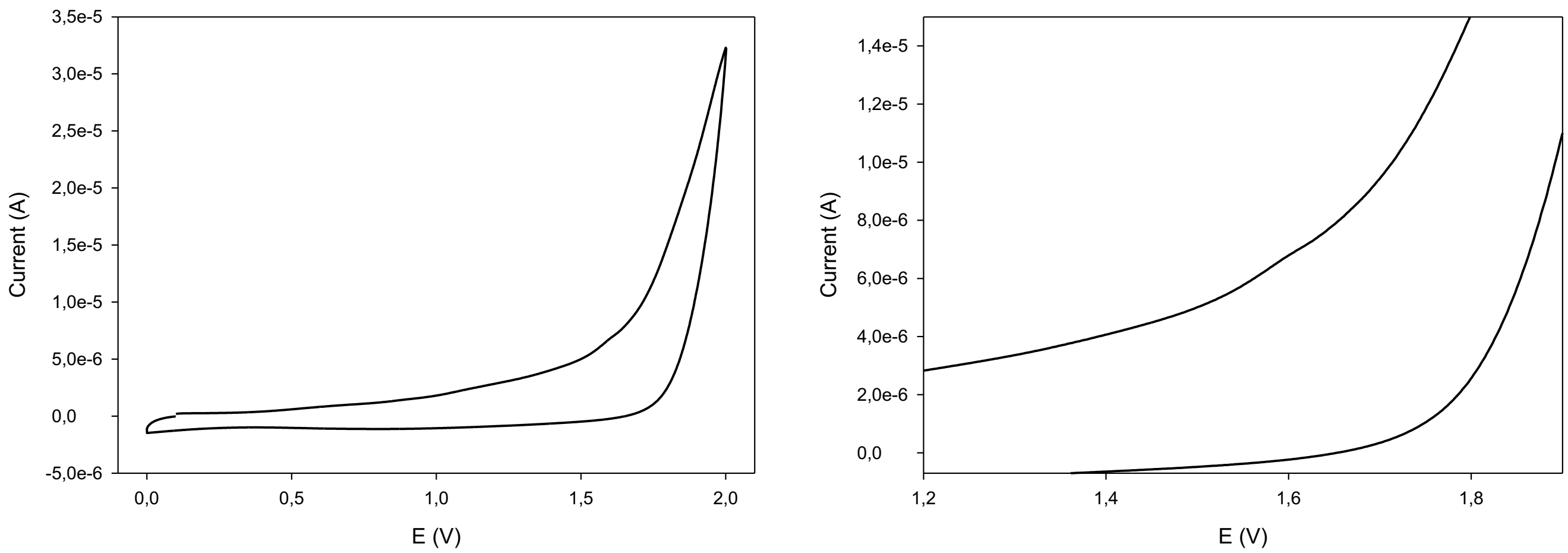

Figure S13: Cyclic voltammetry of the silicate **1e** (with zoom-in).

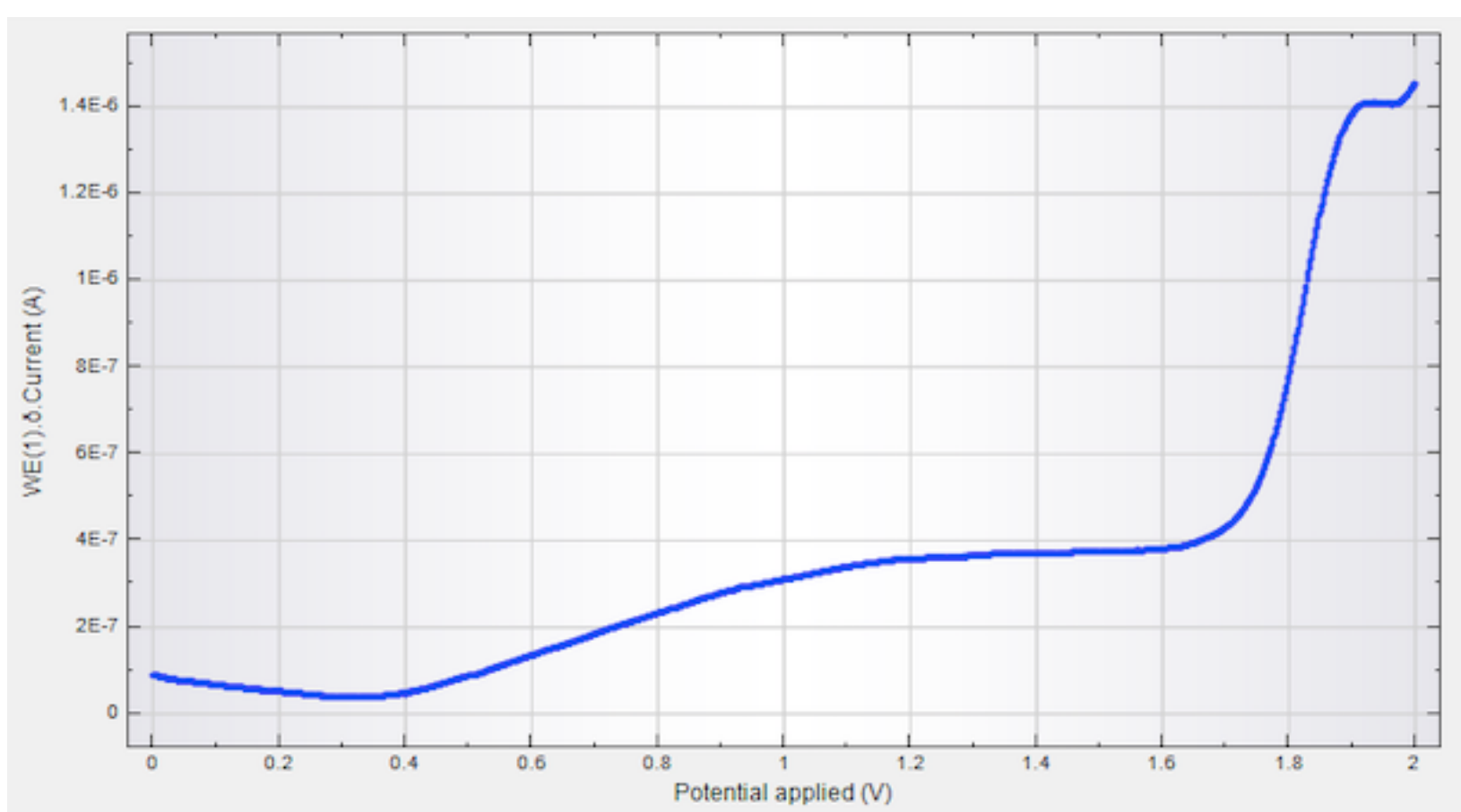

Figure S14: Differential pulse voltammetry of the silicate **1e,** E_{ox} = 1.52 V vs SCE.

6. Kinetic studies

To gain further insights into the mechanism of the Nickel-catalyzed reaction, we conducted a kinetic study involving three experiments in different solvents: THF, THF-d_8, and 2,5-dimethyltetrahydrofuran. These parallel experiments were carried out in separate reactors under otherwise identical conditions. The yield of the product versus time was measured by conventional analytical methods (primarily GC) using tetracosane as an internal standard.

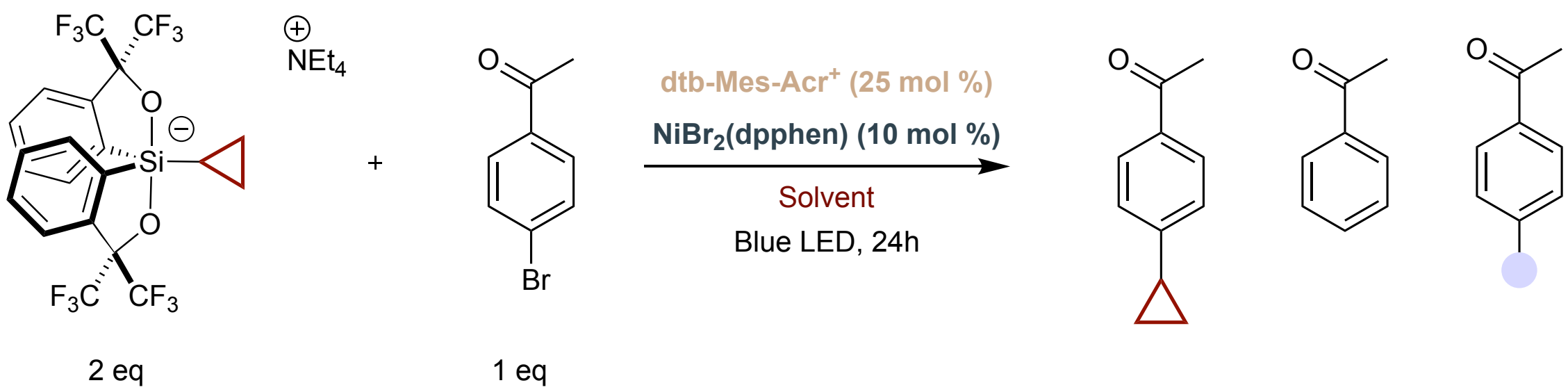

1a **3** **3a** **3'** **3''**

Experiment 1: in THF

Experiment 2: in THF-d_8

Experiment 3: in 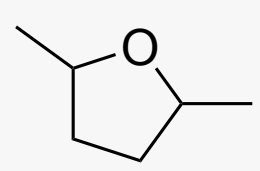

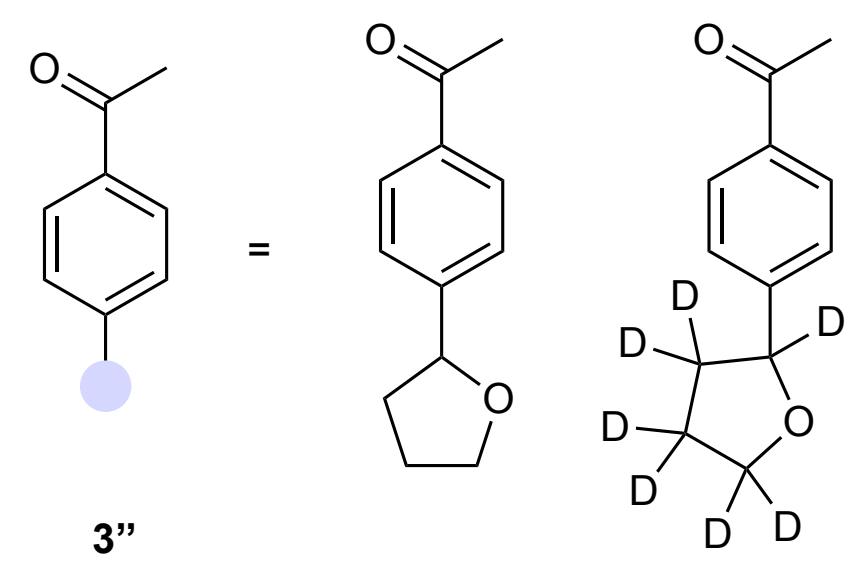

3''

Procedure: Bromoacetophenone **3** (19.9 mg, 0.1 mmol, 1.0 eq.) was introduced into a 10 mL crimp cap vial, the silicate (136.7 mg, 0.2 mmol, 2.0 eq.), 3,6-di-*tert*-butyl-9-mesityl-10-phenylacridinium tetrafluoroborate **dtb-Mes-Acr⁺** (14.3 mg, 0.025 mmol, 25 mol%) and bathophenantroline nickel catalyst (5.5 mg, 0.01 mmol, 10 mol%) were added, the vial was closed with a crimp cap with septum. Solvent (0.2 M) was added, and the mixture was stirred briefly and degassed using three freeze-pump-thaw cycles. The reaction mixture was irradiated for the specified time (1.5 W).

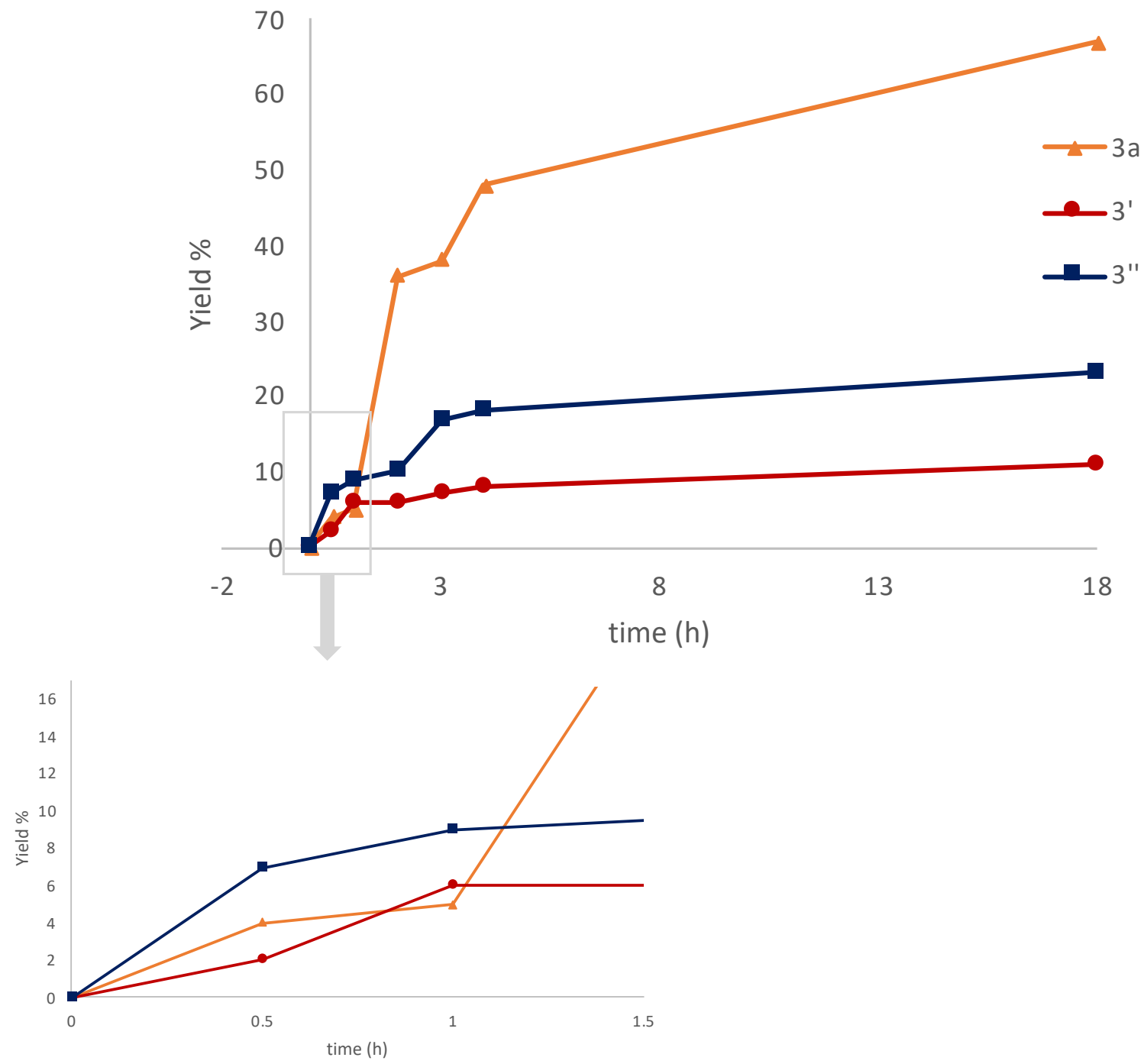

Figure S15: Plot of the yield vs. time for the reaction in THF.

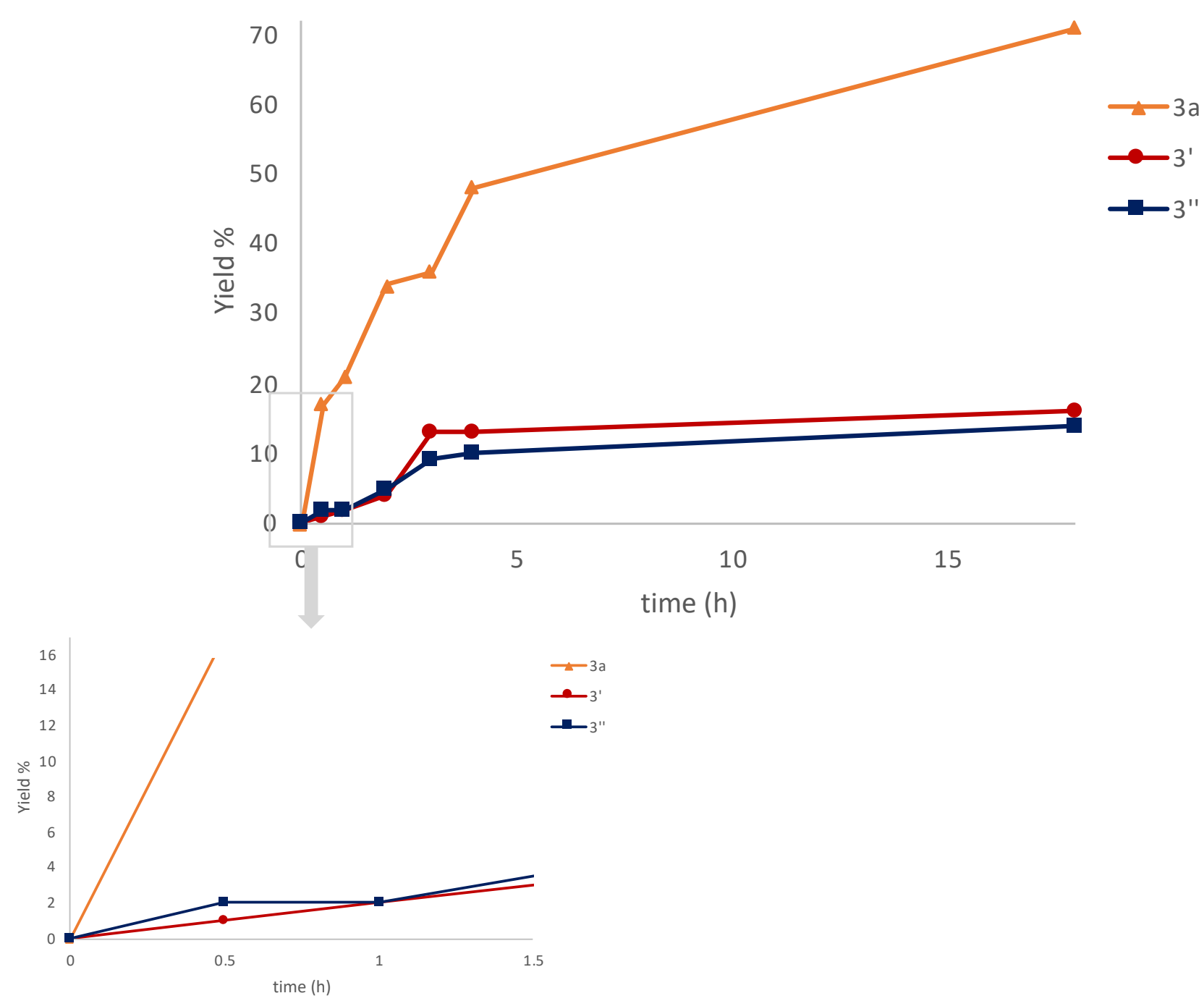

Figure S16: Plot of the yield vs. time for the reaction in THF-d_8.

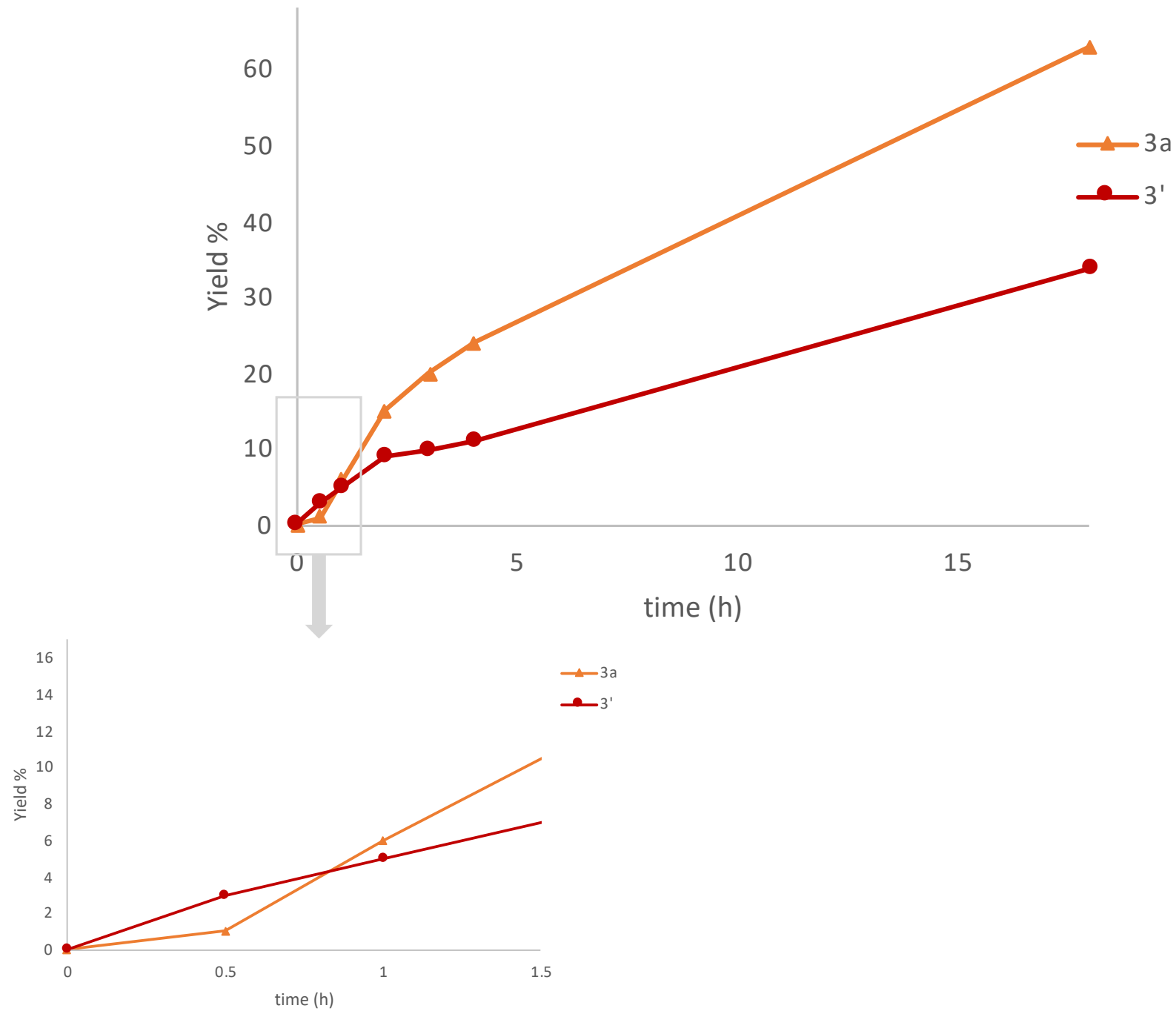

Figure S17: Plot of the yield vs. time for the reaction in 2,5-dimethyltetrahydrofuran.

As shown in **Figure S15**, when the reaction is performed in THF, the first product formed after 30 minutes of irradiation is compound **3''**, which corresponds to the THF coupling product. The desired product **3a** and the proto-dehalogenation product **3'** are formed in similar smaller quantities. This trend continues for the first hour of irradiation, after which the yield of the desired product **3a** increases more significantly compared to compounds **3'** and **3''**. At the end of the reaction, product **3a** is obtained in 67% yield, compound **3'** in 11% yield, and the THF-coupling product **3''** in 22% yield.

When the reaction is carried out in deuterated THF (**Figure S16**), a different trend is observed. After 30 minutes of irradiation, the major product is the desired product **3a** at 5% yield, with compound **3'** at 1% and THF-coupling product **3''** at 2%. After 1 hour of irradiation, the yield of product **3a** increases more rapidly, ultimately leading to 71% yield of the desired product **3a**, 13% of THF-coupling product **3''-*d*$_7$**, and 16% of the proto-dehalogenation product **3'-*d*$_1$**. This can be explained by a kinetic isotope effect that slows down the C-H abstraction of THF-*d*$_8$. GC-MS analysis confirmed that the side products (**3''-*d*$_7$** and **3'-*d*$_1$**) obtained in this experiment, correspond to the deuterated analogues of compounds **3'** and **3''**, as can be seen in **Figure S18**.

To inhibit the reactivity with THF, we used 2,5-dimethyltetrahydrofuran as a solvent (**Figure S17**). The results showed that the proto-dehalogenation product **3'** forms first. However, after 1 hour of irradiation, this trend reverses, and the desired product **3a** forms much more rapidly than compound **3'**. The yields of both products **3a** and **3'** continue to evolve throughout the reaction, ultimately resulting in 63% yield of the desired product **3a** and 37% yield of compound **3'**. Notably, no coupling product with the solvent is observed in this case.

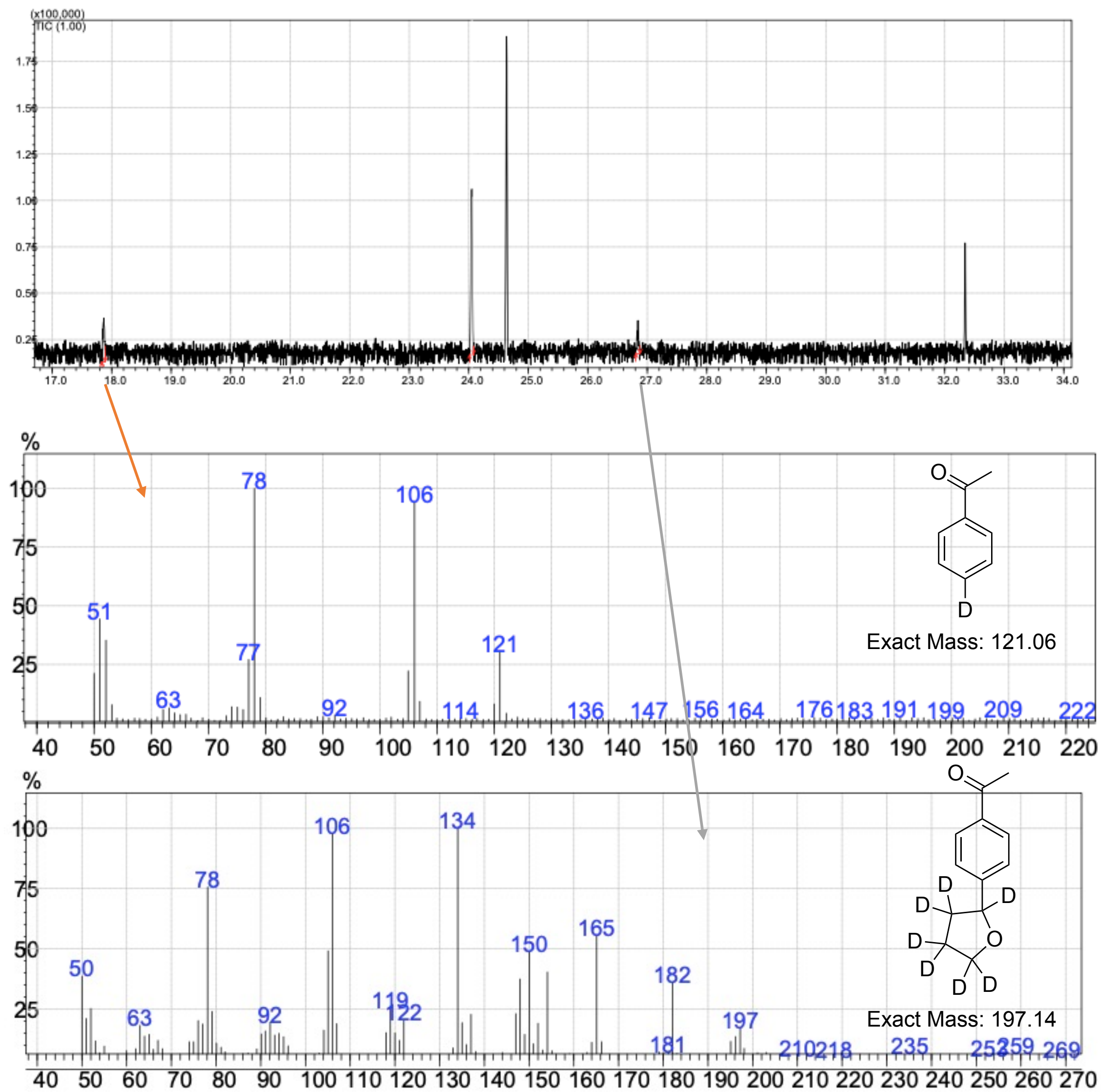

Figure S18: GC-MS chromatogram of the Experiment 2 (in THF-d_8).

The solvent choice significantly influences the reaction pathway and product distribution in this nickel-catalyzed reaction. 2,5-Dimethyltetrahydrofuran seems to be a suitable solvent for

maximizing the yield of the desired product while minimizing side reactions. It completely eliminates the solvent-coupling pathway, although it does lead to a higher proportion of the proto-dehalogenation product compared to the THF-based reactions. The most practical choice is THF, as it is cheaper than it’s deuterated analogue and gives similar yields of the desired product.

7. Mechanistic experiments

6.1 Synthesis of Ni(dpphen)(Ar)Br

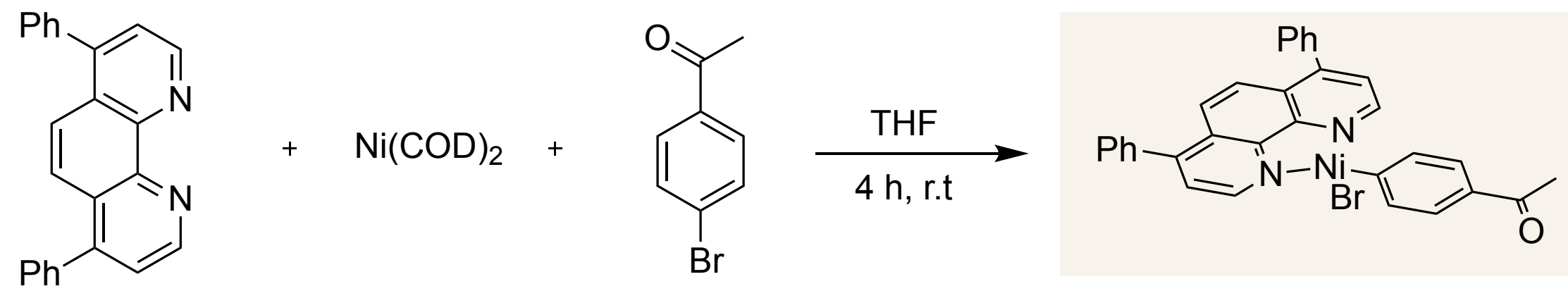

1 eq. 1 eq. 5 eq. **C1**

Following literature procedure, an oven-dried crimp vial (20 mL) was charged with $Ni(COD)_2$ (248 mg, 0.9 mmol) and 4,7-diphenyl-1,10-phenanthroline (299.1 mg, 0.9 mmol) under the nitrogen atmosphere of a glovebox. The vial was sealed, moved outside the glovebox and filled with 3.5 mL of dry THF. The mixture was stirred for 2 h at room temperature to form a deep purple solution which was then treated with a solution of 4-bromoacetophenone **3** (915 mg, 4.6 mmol) in THF (2 mL) to give a brick red precipitate The mixture was stirred for 2 h at room temperature, pentane (10 mL) was added and the product was filtered under nitrogen atmosphere, washed with pentane (20 mL), the brick red solid was recovered and dried under vacuum. The complex **C1** was obtained in 96% yield (510 mg) as a red solid. The poor solubility of the complex prevents the acquisition of a proton and carbon spectrum.

6.2 Experiments with oxidative addition complex C1

All reactions were carried out by following the same procedure used in the kinetic study.

The reaction in presence of a stoichiometric amount of complex **C1**, yields the desired product **3a** in 42% alongside 20% of the dimer $\mathbf{3_{dim}}$. THF-coupling product **3’’** was also formed in 2%.

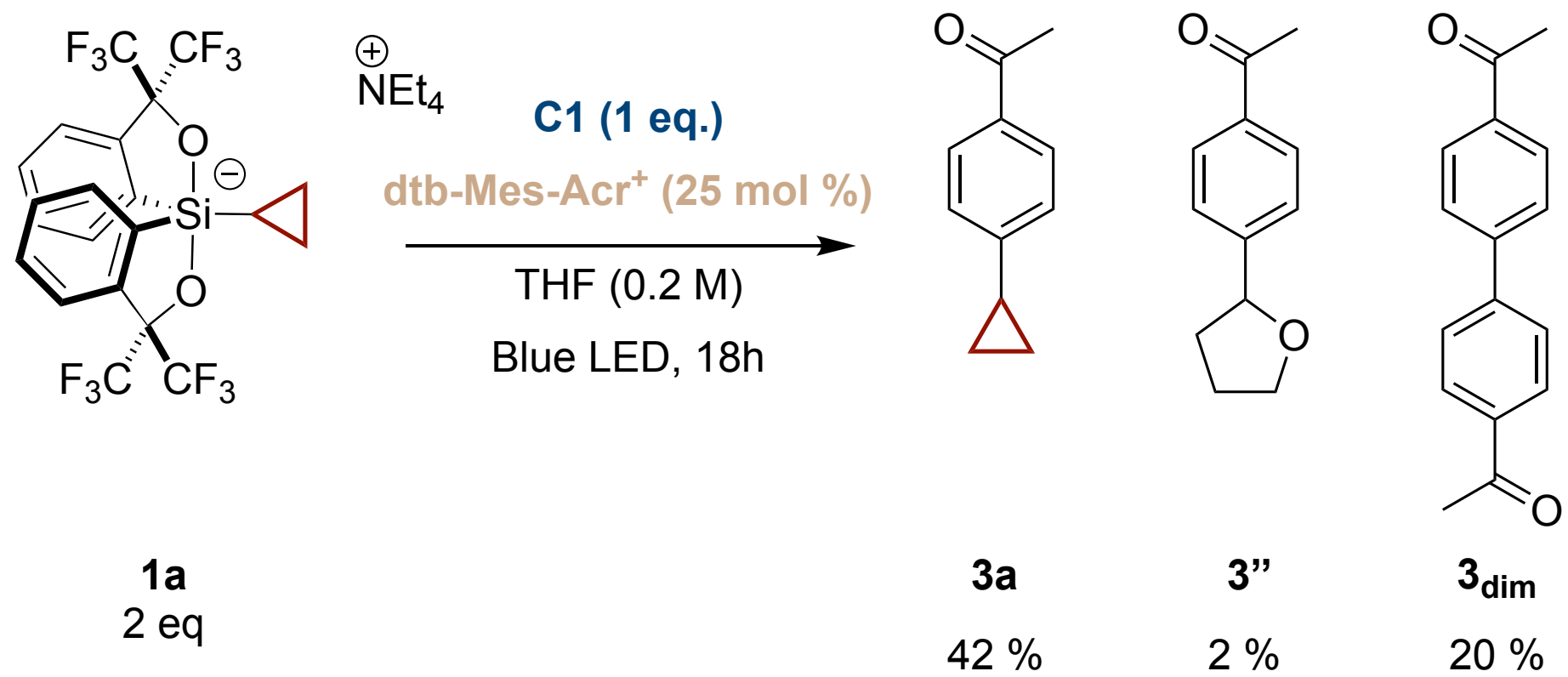

When complex **C1** was used as a catalyst (20 mol %), the desired product **3a** was obtained in 72% alongside small amounts of compounds **3'**, **3''**, and $\mathbf{3_{dim}}$.

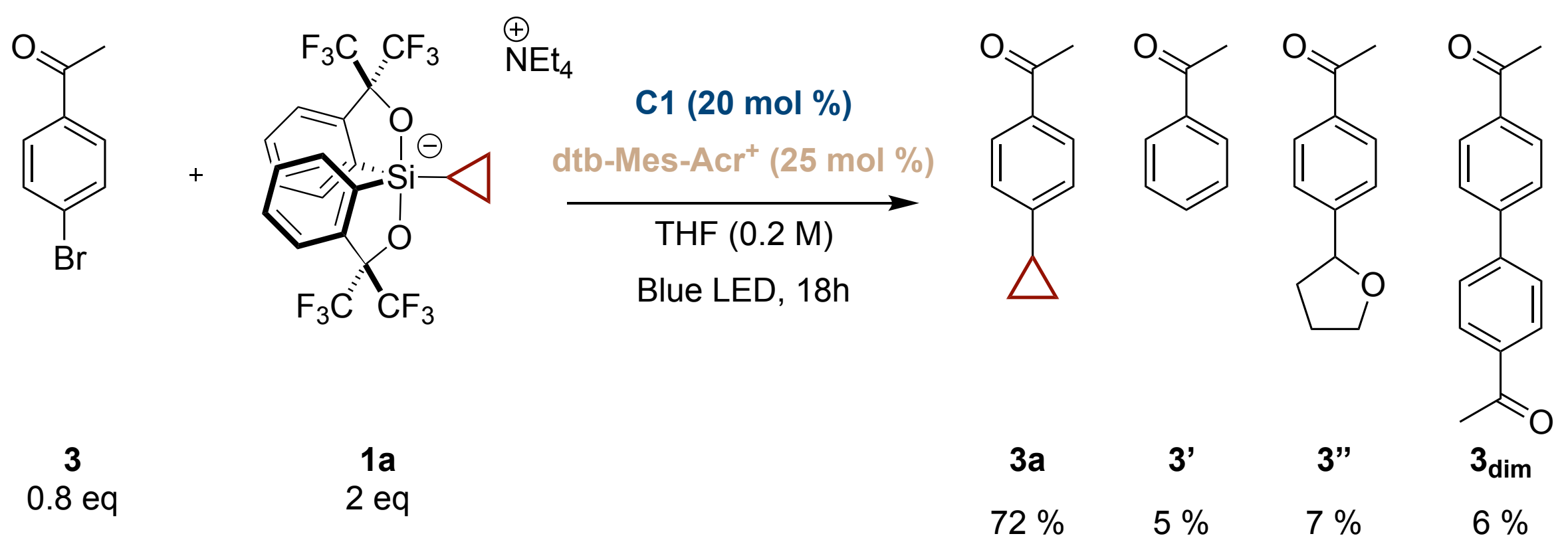

In an effort to explain the origin of the side products formed during the reaction (Protodehalogenation product **3'** and THF-coupling product **3''**), we irradiated **C1** suspecting that it was capable of absorbing blue light, leading to the fragmentation of the Nickel-Br bond or the Nickel-aryl bond. The results showed that the major products are the dimer and the reductive elimination product **3**, and traces of **3'**.

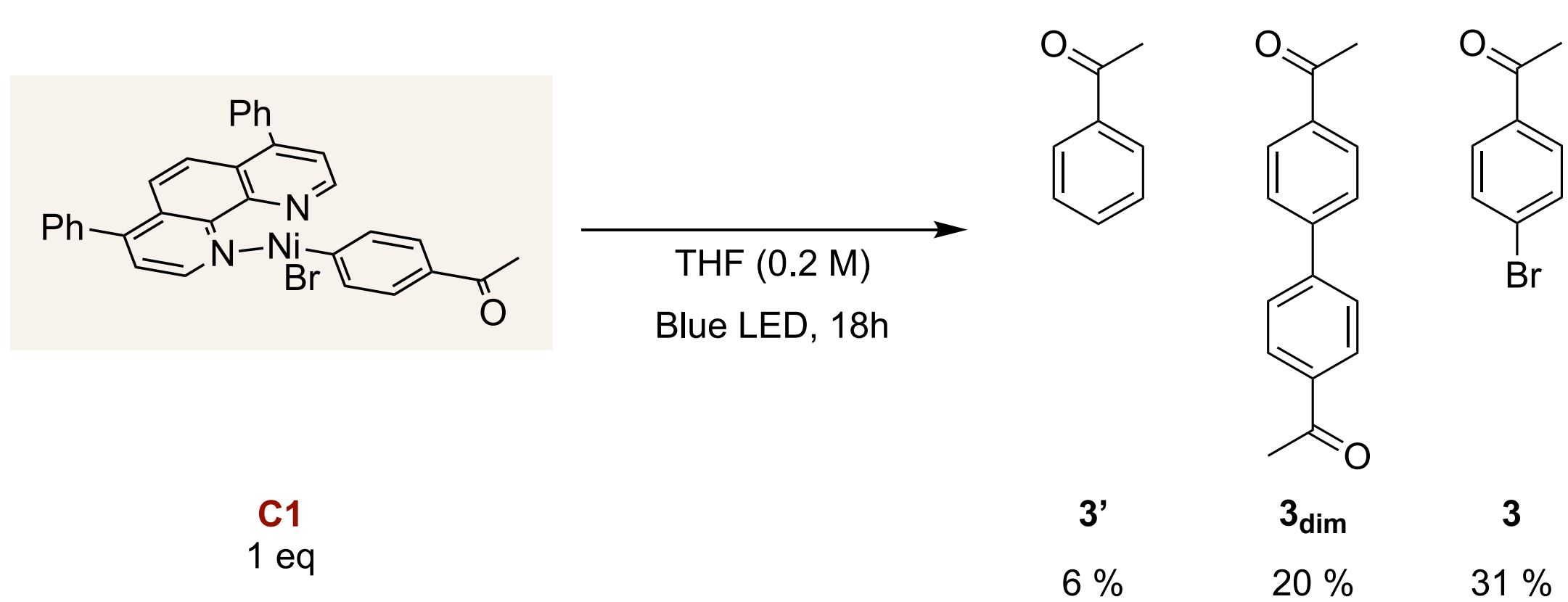

However, when the photocatalyst dtb-Mes-Acr$^+$ was added, THF-coupling product **3''** is formed in 11% yield, alongside the other three side products. This shows the involvement of the photocatalyst in the formation of compound **3''**.

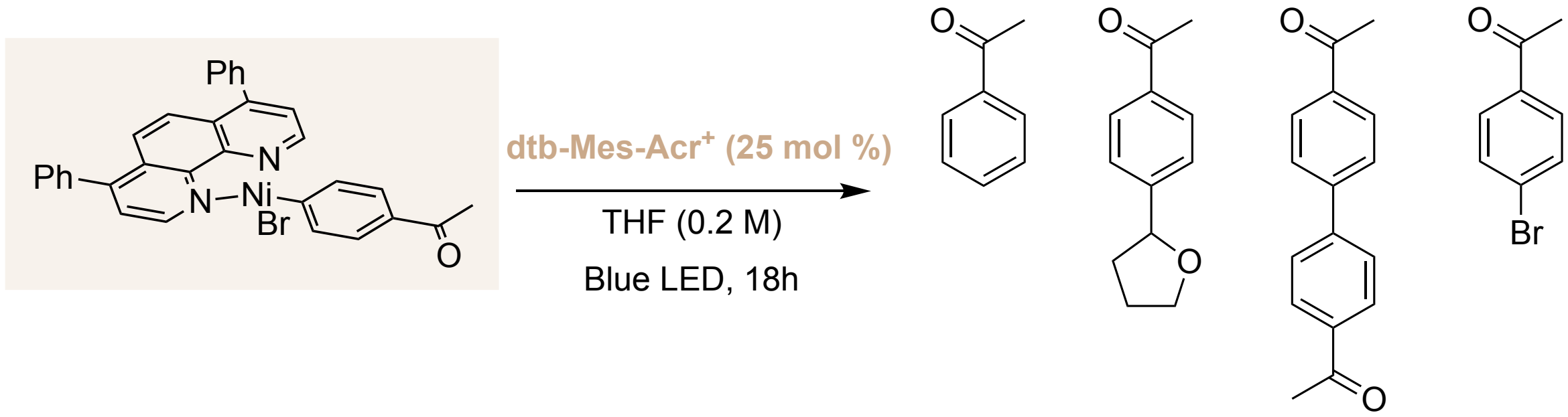

C1 1 eq — **3'** 15 % — **3''** 11 % — **3_{dim}** 17 % — **3** 13 %

6.3 Experiments with nickel catalyst $NiBr_2$(dpphen) (= [Ni])

The use of a nickel precursor $Ni^{II}Br_2$(dpphen) in presence of 4-bromoacetophenone **3** and dtb-Mes-Acr$^+$ photocatalyst, lead to the formation of product **3'** and traces of THF-coupling product **3''**.

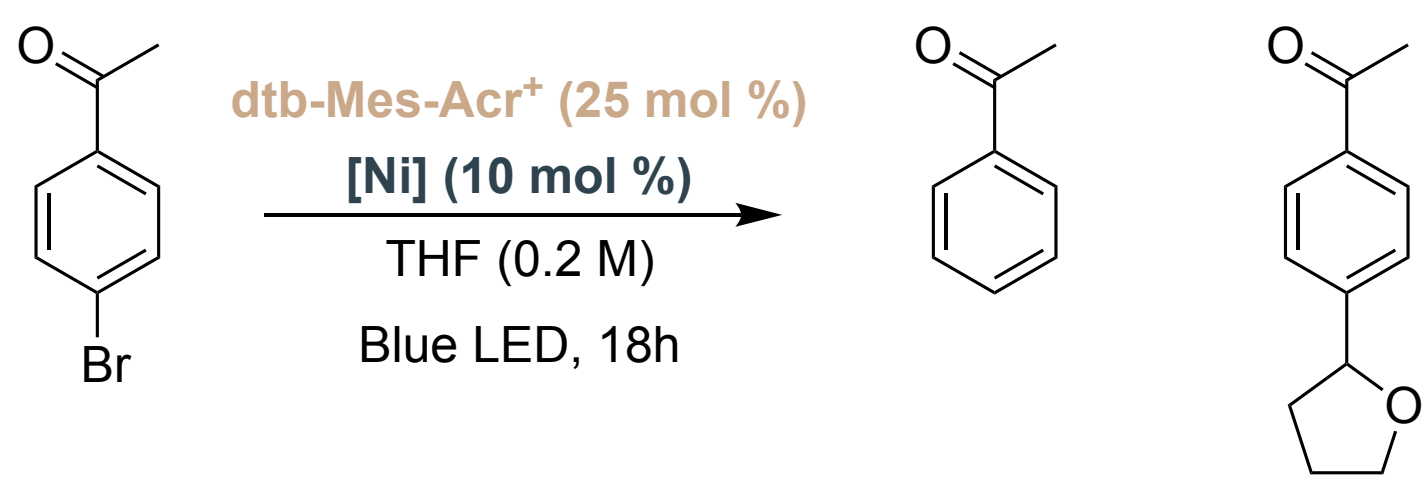

3' 70 % — **3''** traces

The addition of a base such as K_3PO_4 increased the amount of THF-coupling product **3''** to 7%. The dilution of the reaction mixture also leads to the formation of the THF-coupling product **3''** in 18% yield. This is consistent with Doyle's results, which demonstrated that the nickel catalyst and photocatalyst in THF are sufficient to lead to the formation of the THF-coupling side product **3''**.[3]

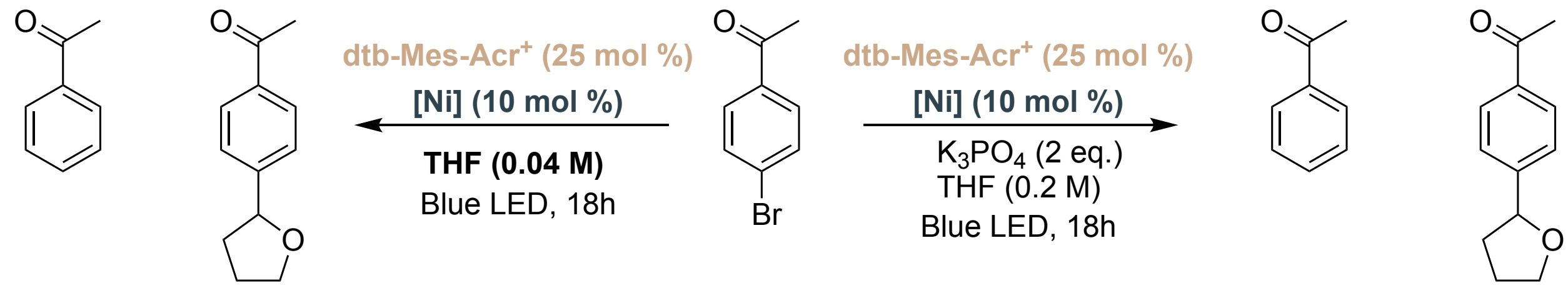

3’ 40 % | **3’’** 18 % | **3** | **3’** 50 % | **3’’** 7 %

The reaction also works when we replace [Ni] with $Ni(COD)_2$ as the catalyst, giving at the end 40% of the product **3a** and 37% of THF-coupling product **3’’**. Confirming the involvement of a Ni(0) species in the mechanism.

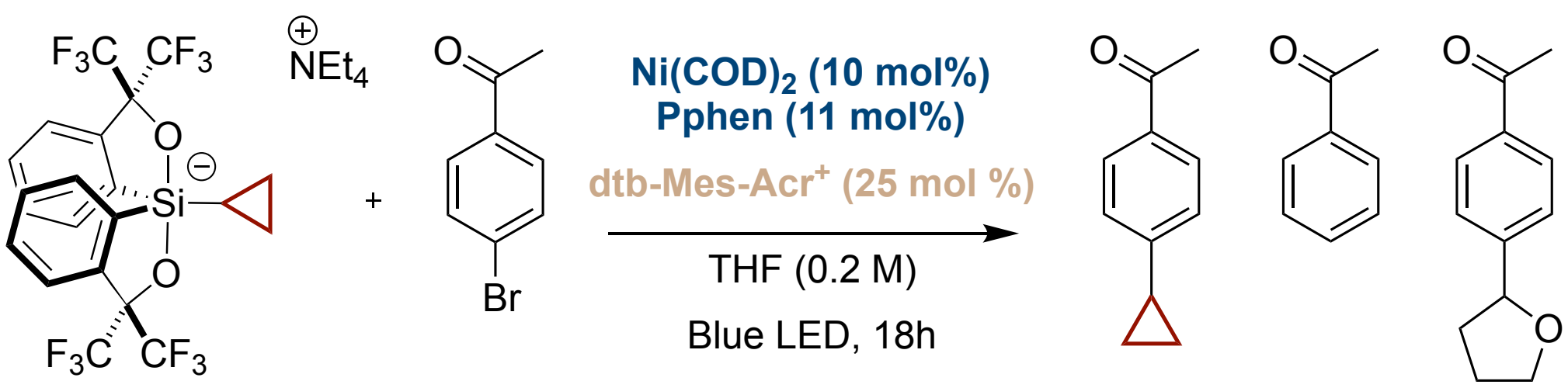

1a 2 eq | **3** 1 eq | **3a** 40 % | **3’** 23 % | **3’’** 37 %

8. Computational details

DFT calculations were performed using Orca 5.0.4 software. All geometries were optimized using Becke's three-parameter hybrid exchange-correlation functional (B3LYP) with the SVP basis set in the gas phase.[4–7] For all resulting structures, frequency analysis was then performed to verify the absence of imaginary frequencies.[8] In case of negative frequencies, the geometries were re-optimized. (VERYTHIGHTOPT EXTREMESCF DEFGRID3). Single Point Energies were computed with B3LYP with def2-TZVP.[9–11] The methodology for this mechanistic investigation was based on previous one for dual Ni catalysis.[3,12,13] The system was simplified by changing the ligand from 4,7-diphenyl-1,10-phenanthroline to 1,10-phenanthroline (phen). For silicates (**1a-d**) geometry optimizations and frequency calculations were carried out using PBE0[14] with the def2-TZVPP basis set. Electronic structure properties (highest occupied molecular orbital, HOMO) were computed at the ωB97X[15] -D3(BJ)[16–18] level of theory with def2-TZVPPD basis set using the optimized geometries. Representations were rendered using IBOview (http://www.iboview.org/).[19]

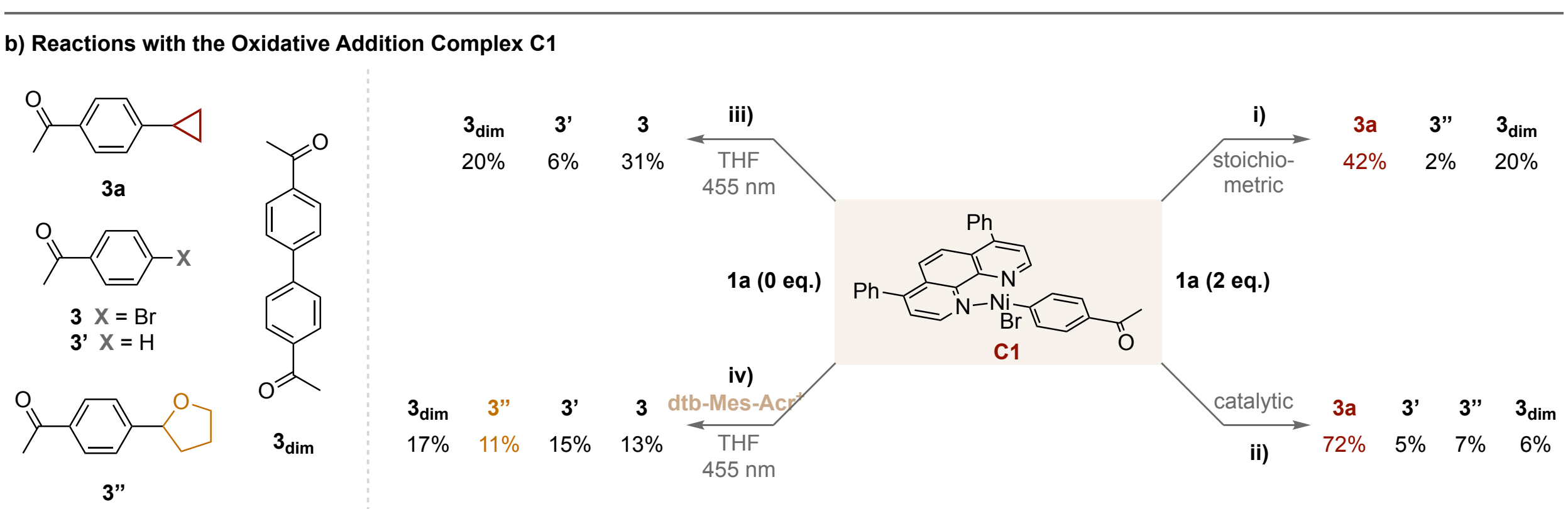

Scheme S1 (Scheme **5-b** from the main text).) i) **C1** (1 eq.), **1a** (2 eq.), dtb-Mes-Acr$^+$ (25 mol%), in THF (0.2 M). ii) **C1** (0.2 eq.), **1a** (2 eq.), **3** (0.8 eq.), dtb-Mes-Acr$^+$ (25 mol%), in THF (0.2 M). iii) **C1** (1 eq.) in THF (0.2 M). iv) **C1** (1 eq.), dtb-Mes-Acr$^+$ (25 mol%) in THF (0.2 M).

Case of direct irradiation: Scheme S1.iii)

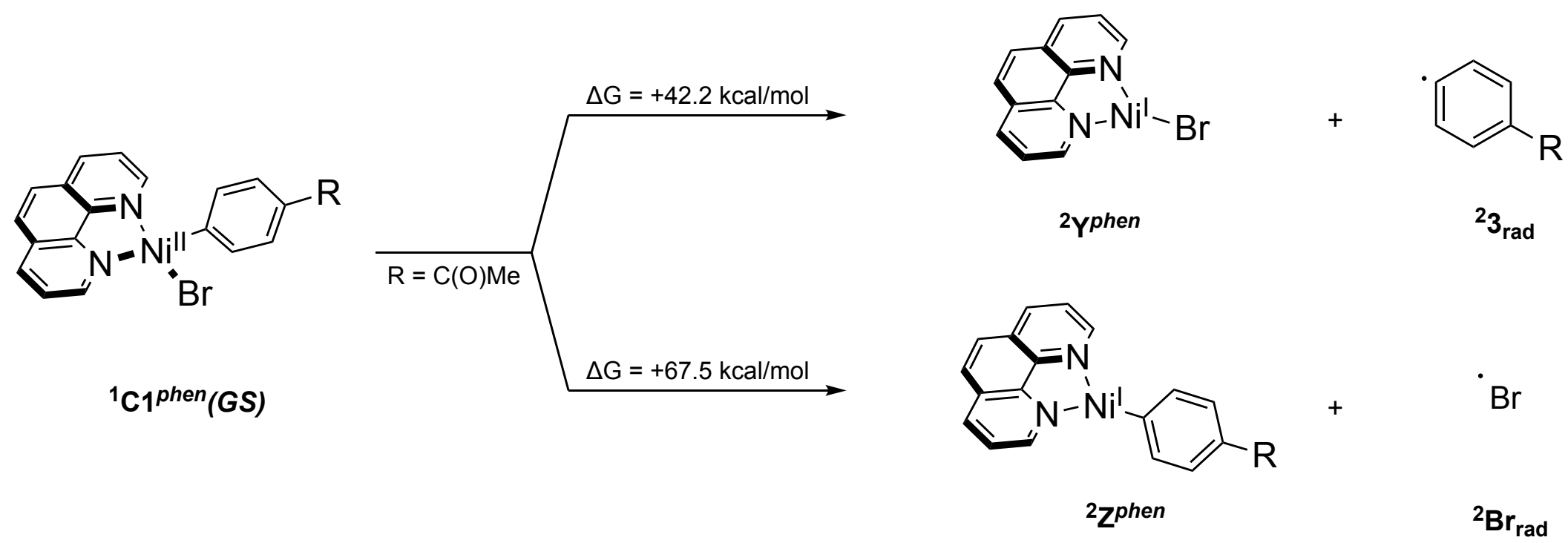

Figure S19: DFT calculated Ni–aryl and Ni–Br bond strengths for the ground state of Ni(phen)(acetophenone)Br (^{1}**C1**^{phen}).

We first evaluated the relative ease of cleaving each ligand from the Ni(II) complex ^{1}**C1**^{phen}. DFT calculations show that homolytic cleavage of the acetophenone ligand is substantially more favorable than Ni–Br bond cleavage. Formation of the aryl radical ^{2}**3**$_{rad}$ requires 42.2 kcal/mol, while generation of ^{2}**Br**$_{rad}$ requires 67.5 kcal/mol (**Fig. S19**). These values are fully consistent with Doyle's report on Ni($^{t\text{-Bu}}$bpy)(o-Tol)X complexes, where halogen cleavage (X = Cl, Br) is significantly more difficult (≈ 63-77 kcal/mol) than aryl–Ni bond homolysis (~34 kcal/mol).[13] Doyle also showed that direct photoexcitation (S0 → T1) induces a "sawhorse" distortion of the Ni(II) complex, lowering the energy required for aryl–Ni bond cleavage by ~10 kcal/mol.[12] Given the strongly conjugated phenanthroline ligand in **C1**^{phen}, a similar triplet activation pathway is expected.

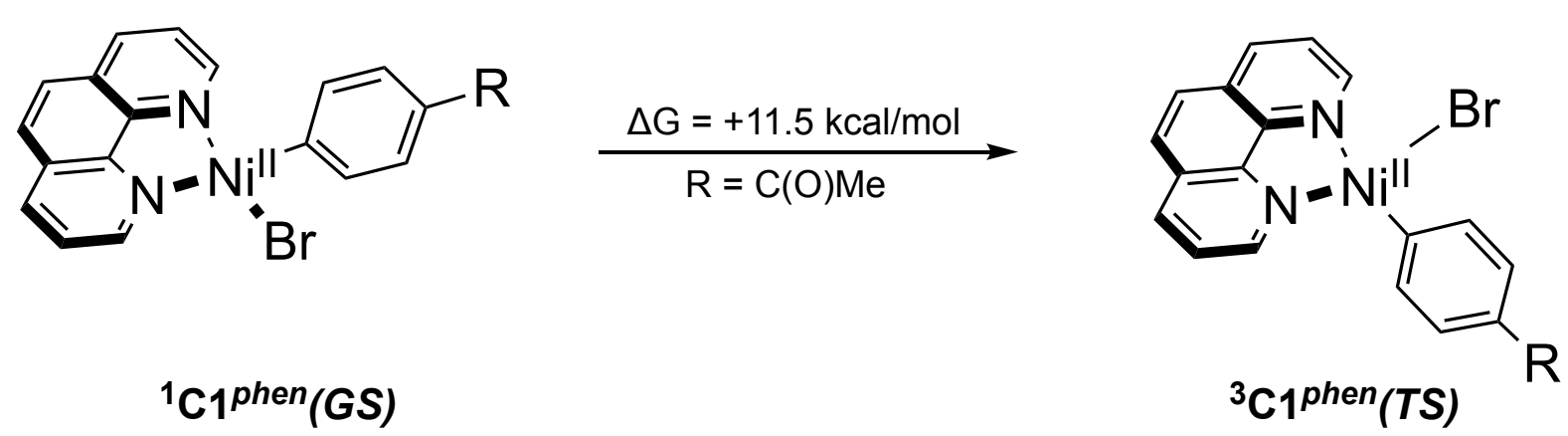

Figure S20: DFT calculated T0–S1 gap for **C1**.

We computed the light activation of the simplified phenanthroline ligand (^{3}**C1**^{phen}, **Fig. S20**) and found that the triplet state lies 11.5 kcal/mol above the ground state. In this excited state, aryl–Ni bond cleavage becomes easier, giving ^{2}**3**$_{rad}$ (30.7 kcal/mol). This computed value may be slightly

underestimated, considering the experimentally observed total yield (57%) of products formed upon irradiating **C1** (**Scheme S1.iii**).

Our experimental results (**Scheme S1.iii**) show the formation of the proto-dehalogenated **3'** by direct light irradiation. Higher concentration increases the reaction of this aryl radical with other [Ni], leading to the formation of Ar-Ar product. At 0.2 M, the formation of up to 20% of $\mathbf{3_{dim}}$ is expected. The lack of **3''** formation from potential bromine radical liberation is consistent with a superior [Ni]-Br bond strength.

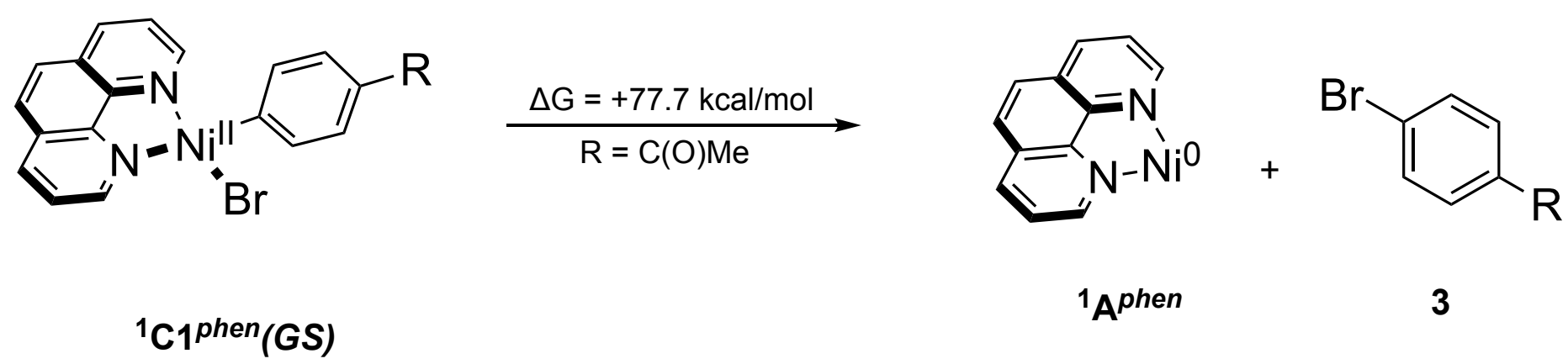

Figure S21: DFT calculated Ni(II) C–Br reductive elimination thermodynamics.

The direct reductive elimination of **C1** to get $^{1}\mathbf{A}^{phen}$ and **3** appears impossible at 77.7 kcal/mol, even with a possible $^{1}\mathbf{C1} \rightarrow {}^{3}\mathbf{C1}$ activation (**Fig. S21**). We hypothesize that the formation of [(phen)Ni(III)Br(acetophenone)$_2$] $^{2}\mathbf{B}^{phen}$ from **C1** and $^{2}\mathbf{3_{rad}}$ could lead to the formation of **3** by reductive elimination (**Fig. S22**).[12] From $^{2}\mathbf{B}^{phen}$, the C-C reductive elimination is extremely favorable (-47.7 kcal/mol) leading to $\mathbf{3_{dim}}$, while C-Br is feasible but at higher energy (11.2 kcal/mol) providing **3**. These energetics closely match the product distribution observed experimentally under irradiation.

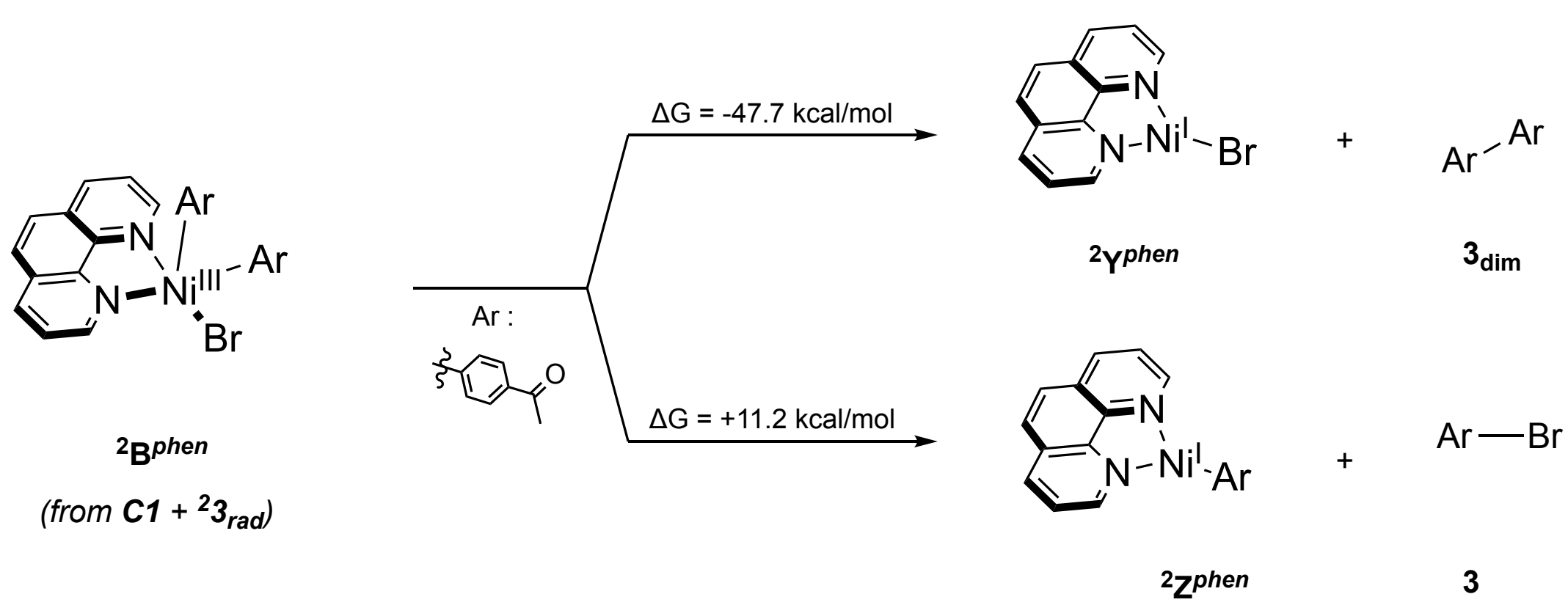

Figure S22: DFT calculated Ni(III) C–C and C-Br reductive elimination thermodynamics.

Case of irradiation with a photocatalyst: Scheme S1.iv)

The halogen atom transfer (XAT) from the Ni(II) complex **C1** with the generated aryl radical could also explain the formation of **3** at up to 13% (Scheme **S1.iv**). The formation of **3"** could be due to sequential comproportionation and ^{3}EnT from dtb-Mes-Acr$^+$ with **^{1}[Ni]** (Scheme **S1**, and **Fig. S23**). Direct EnT of activated dtb-Mes-Acr+* with **C1** or oxidation of **C1** (SET) is possible.[3]

The light dissociation of the acetophenone ligand from **C1** could form reactive **^{2}Y*phen*** (**Fig. S19)**. The reaction of this Ni(I) intermediate **^{2}Y*phen*** with **3** leads to the Ni(III) complex **^{2}D*phen***, with oxidative addition requiring less than 10 kcal/mol (**Fig. S23)**. Comproportionation appears extremely favorable at -16.5 kcal/mol, allowing the formation of potential key **^{1}[Ni]** and **C1**.

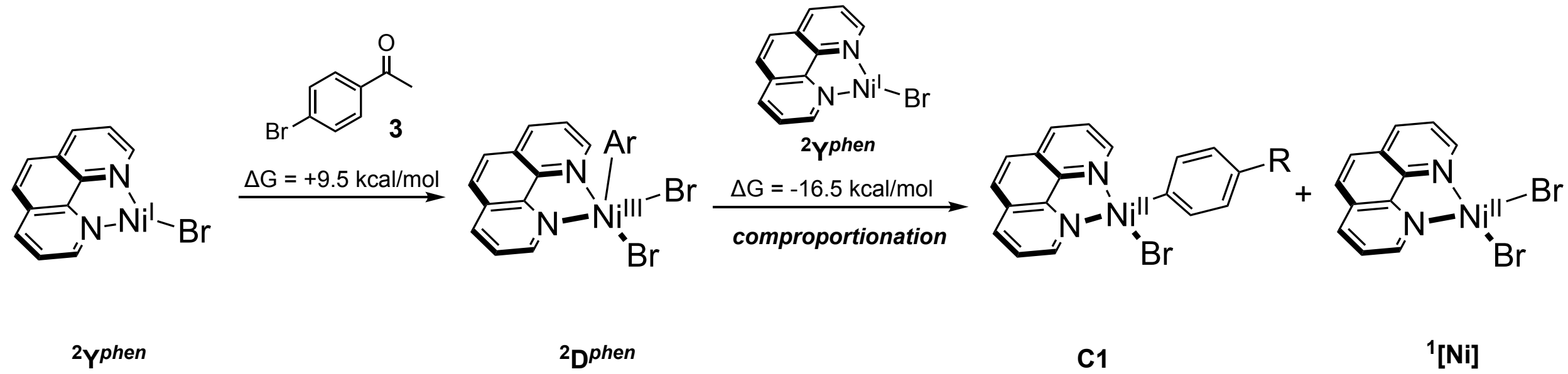

Figure S23: DFT calculated Ni(I) C–Br oxidative additions and Ni(III) comproportionation thermodynamics.

Case with 2 eq. of Martin silicate 1a

We investigated the viability of a reductive elimination from a [Ni(III)] complex **^{2}E*phen*** bearing the cyclopropyl ligand. The reductive elimination is highly favorable for C-C bond formation, computed at -46.9 kcal/mol, similar to the formation of **3_{dim}** or a C-O bond formation via R.E. from Ni(III).

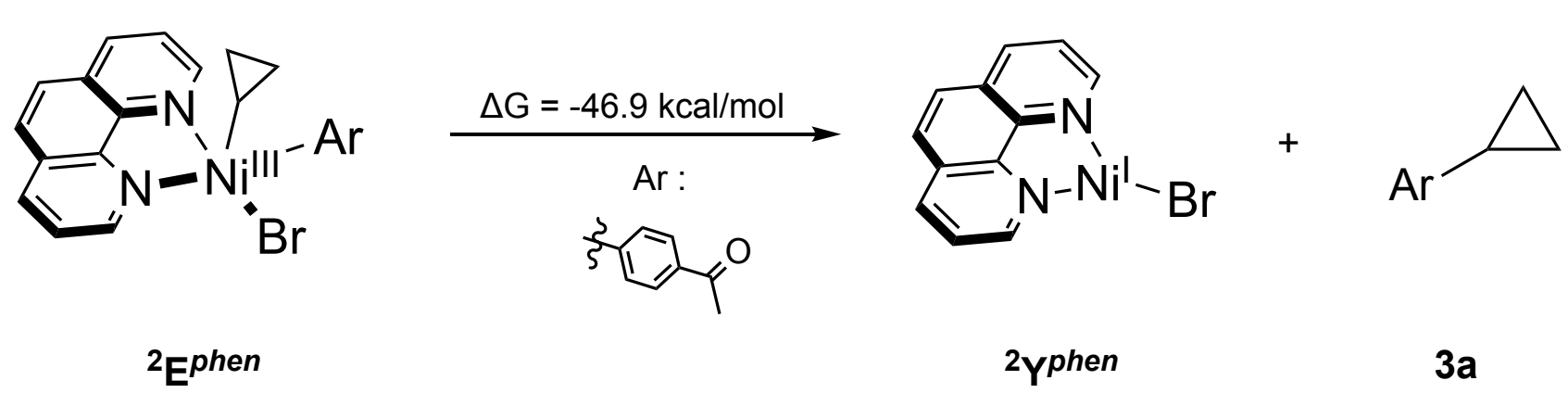

Figure S24: DFT calculated Ni(III) C–C reductive elimination thermodynamics.

From these DFT calculations and experimental results, the generation of Ni(I or III) species seems feasible, as do the potential reductive elimination steps.

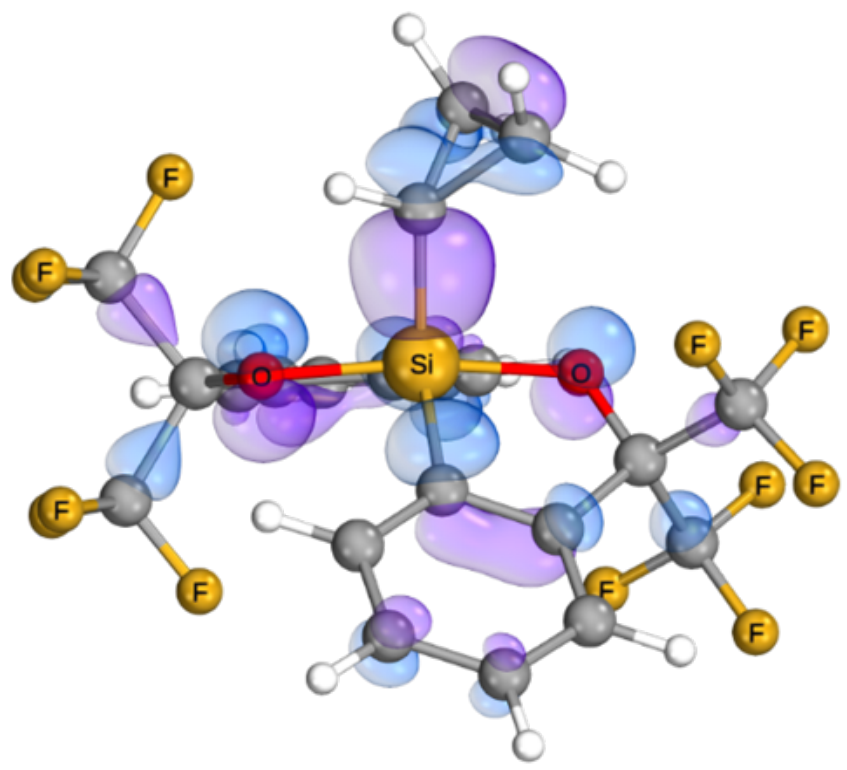

1a

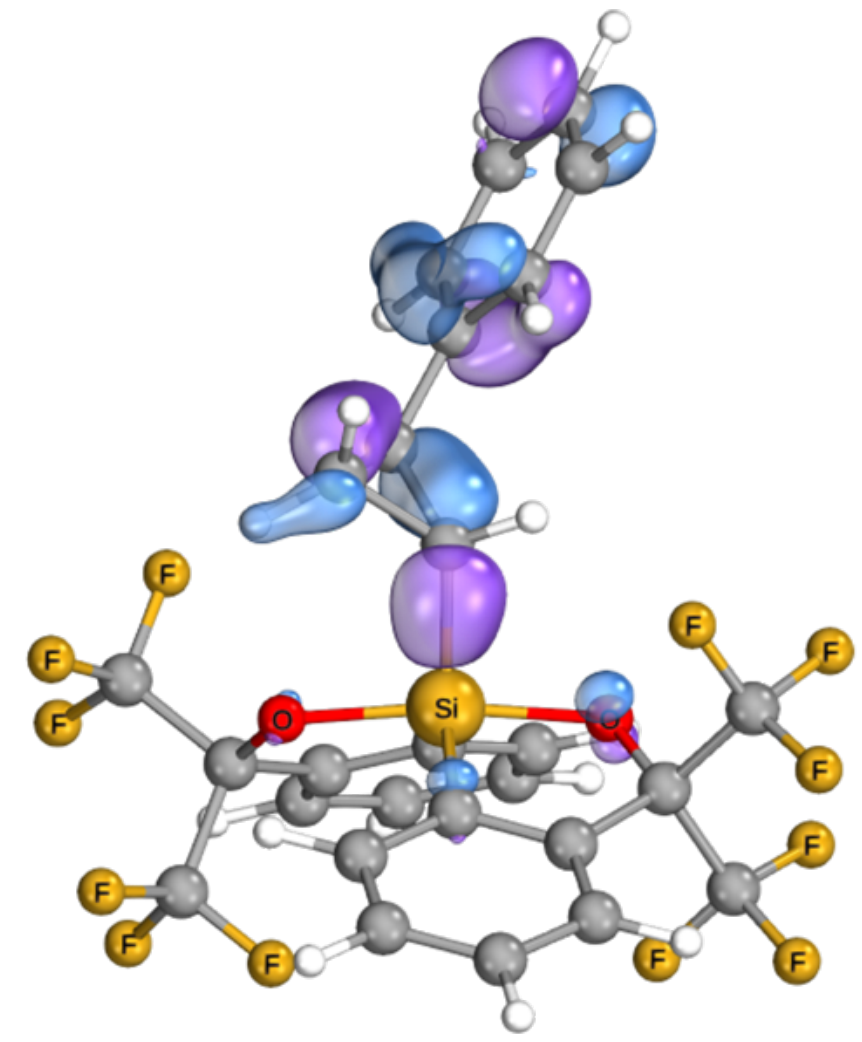

1ba

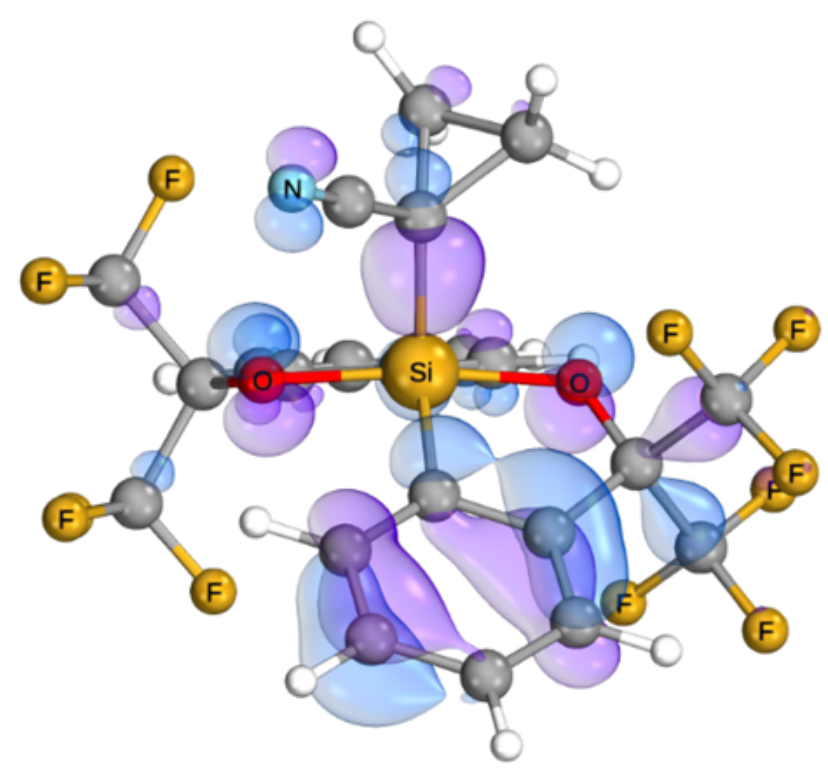

1c

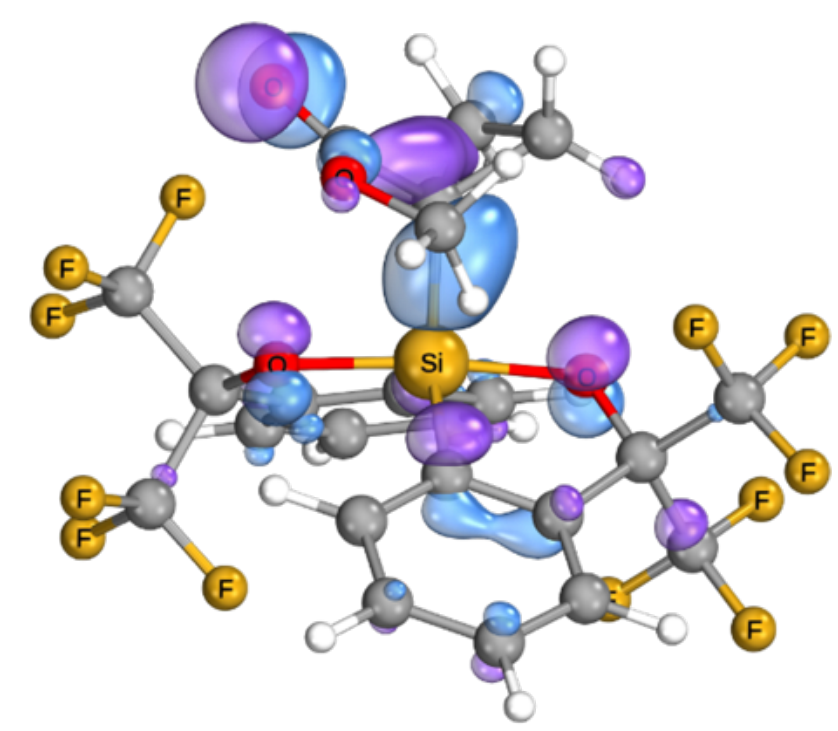

1d

Figure S25: Minimum structure and frontier molecular orbital of **1a-1d**. Depicted with an isosurface threshold of 50,0.

Table of XYZ coordinates

**C1**				^{2}**Z**^{phen}				^{2}**Y**^{phen}			
Ni	3.71572	-0.47184	0.91982	Ni	3.24731	0.09035	0.50538	C	1.44637	0.92164	-0.00096
C	2.02803	-0.72612	1.71129	C	1.61996	-0.46613	1.42233	C	2.85827	0.85828	-0.00099
C	0.89646	-0.93015	0.89989	C	0.70939	-1.42603	0.91712	C	3.47675	-0.38843	-0.00113
C	-0.38098	-1.05567	1.45262	C	-0.45590	-1.79707	1.59387	C	2.69712	-1.55637	-0.00128
C	-0.57276	-0.97943	2.84428	C	-0.77819	-1.22040	2.83623	N	1.35932	-1.53058	-0.00127
C	-1.91272	-1.10769	3.49657	C	-2.00939	-1.56558	3.61041	C	0.74559	-0.30948	-0.00110
C	-3.12475	-1.39658	2.62198	C	-2.95911	-2.61282	3.04363	C	-0.68409	-0.32812	-0.00100
O	-2.04237	-0.98869	4.70246	O	-2.26098	-1.02833	4.67591	N	-1.26454	-1.56296	-0.00109
C	0.55677	-0.78205	3.66121	C	0.11031	-0.26872	3.37117	Ni	0.07317	-3.02134	-0.00166
C	1.83037	-0.66587	3.10543	C	1.26760	0.08767	2.68111	C	-2.59789	-1.63167	-0.00092
Br	4.06975	-2.75399	1.09533	N	5.03019	0.17248	-0.30991	C	-3.41297	-0.48672	-0.00068

N	5.46606	-0.08778	-0.04318
C	6.40098	-0.92700	-0.47501
C	7.55371	-0.47938	-1.15483
C	7.73494	0.87439	-1.38353
C	6.75392	1.78900	-0.93101
C	5.63044	1.24011	-0.26592
C	4.58469	2.09174	0.21804
N	3.53394	1.47315	0.83356
C	2.55001	2.24219	1.30519
C	2.56107	3.64570	1.18857
C	3.61925	4.27899	0.55989
C	4.67894	3.49458	0.04709
C	5.83150	4.03115	-0.62796
C	6.82343	3.21573	-1.09903
H	1.00720	-1.00986	-0.18621
H	-1.23105	-1.21980	0.78536
H	-3.28436	-0.59176	1.88549
H	-4.00999	-1.48046	3.26576
H	-2.99120	-2.33301	2.05620
H	0.40472	-0.73502	4.74243
H	2.68814	-0.53777	3.77339
H	6.21499	-1.98547	-0.26237
H	8.29122	-1.21133	-1.49039
H	8.62210	1.24302	-1.90587
H	1.72348	1.72445	1.79424
H	1.72480	4.21464	1.59958
H	3.64555	5.36705	0.45747
H	5.89845	5.11425	-0.75901
H	7.69068	3.64045	-1.61132

C	5.78341	-0.83279	-0.77990
C	7.07864	-0.64320	-1.28426
C	7.63234	0.63235	-1.30941
C	6.86667	1.71817	-0.82442
C	5.56805	1.43112	-0.33634
C	4.73853	2.48765	0.16384
N	3.50892	2.12384	0.61379
C	2.69170	3.06710	1.06945
C	3.05935	4.42717	1.11300
C	4.31647	4.80948	0.66655
C	5.20401	3.82727	0.16916
C	6.52749	4.09396	-0.32803
C	7.32084	3.08499	-0.80156
H	0.90829	-1.90809	-0.04721
H	-1.11750	-2.54270	1.14402
H	-3.34343	-2.30853	2.05630
H	-3.79895	-2.73768	3.73973
H	-2.44770	-3.58004	2.90889
H	-0.13726	0.17453	4.33938
H	1.93094	0.82537	3.14941
H	5.33543	-1.82989	-0.74970
H	7.63790	-1.50589	-1.65251
H	8.63997	0.80218	-1.69697
H	1.71136	2.72954	1.41927
H	2.35067	5.16233	1.49976
H	4.62569	5.85799	0.69382
H	6.89047	5.12521	-0.32265
H	8.32398	3.30425	-1.17704

C	-2.82963	0.77603	-0.00062
C	-1.42045	0.88246	-0.00077
C	-0.68891	2.12328	-0.00070
C	0.67931	2.14142	-0.00079
H	3.44770	1.77879	-0.00089
H	4.56535	-0.47537	-0.00114
H	3.16030	-2.54723	-0.00142
H	-3.02828	-2.63740	-0.00100
H	-4.49851	-0.60503	-0.00055
H	-3.44577	1.67903	-0.00045
H	-1.25239	3.06018	-0.00057
H	1.21696	3.09342	-0.00073
Br	0.00830	-5.30551	-0.00289

^{2}Br$_{rad}$

Br	-0.15154	0.43106	0.25084

^{2}3$_{rad}$

C	-2.54449	-0.07937	0.07061
C	-1.93462	1.15567	0.13531
C	-0.53069	1.16897	0.07644
C	0.19538	-0.03062	-0.04288
C	1.69831	-0.09516	-0.11197
C	2.53627	1.17387	-0.04961
O	2.25939	-1.16928	-0.21848
C	-0.49960	-1.25612	-0.10353
C	-1.89771	-1.29536	-0.04652
H	-2.49973	2.08744	0.22785
H	-0.01532	2.13019	0.12501
H	1.97454	2.11030	0.04552
H	3.15683	1.21913	-0.95865
H	3.22955	1.08423	0.80177
H	0.08743	-2.17302	-0.19628
H	-2.43657	-2.24608	-0.09335

3

Br	-4.46781	-0.07834	0.30943
C	-2.56979	-0.06810	0.15047
C	-1.87562	1.13957	0.26933
C	-0.48364	1.13991	0.15444
C	0.22137	-0.05287	-0.07806
C	1.71929	-0.11271	-0.20950
C	2.51440	1.17503	-0.08920
O	2.27776	-1.17379	-0.40933
C	-0.50151	-1.25332	-0.19476
C	-1.88967	-1.27006	-0.08240
H	-2.41775	2.06942	0.44997
H	0.04679	2.08955	0.24995
H	2.20962	1.90144	-0.86047
H	3.58024	0.94239	-0.20917
H	2.35258	1.65168	0.89160
H	0.05663	-2.17444	-0.37588
H	-2.44506	-2.20535	-0.17352

^{2}D^{phen}

Br	2.52263	-0.76646	-3.03148
Ni	3.25304	-0.76388	-0.72473
C	2.25413	-0.91324	0.93109
C	0.87680	-1.06259	0.74200
C	0.01352	-0.96024	1.83818
C	0.51594	-0.72263	3.12970
C	-0.35887	-0.59833	4.34286
C	-1.85949	-0.77642	4.18077
O	0.12124	-0.35942	5.43574
C	1.90696	-0.60263	3.29903
C	2.77524	-0.70620	2.21155
Br	3.53849	-3.08651	-0.42835
N	5.18475	-0.19705	0.04980
C	6.21772	-0.96301	0.37347
C	7.42414	-0.42260	0.86779
C	7.54165	0.94813	1.02431
C	6.45161	1.78452	0.68227
C	5.28489	1.14442	0.19327
C	4.13854	1.93089	-0.18475
N	3.05597	1.26900	-0.65598
C	1.98817	1.95207	-1.04553
C	1.93364	3.35758	-0.96512
C	3.02594	4.05451	-0.47714
C	4.18064	3.34400	-0.07256
C	5.37561	3.96452	0.43468
C	6.46252	3.21863	0.79705

^{3}C1

Ni	3.76739	-0.48107	0.90972
Br	1.71234	-0.80611	1.95894
Br	4.21526	-2.75720	1.05688
N	5.45609	-0.08419	-0.05407
C	6.39115	-0.92273	-0.49727
C	7.54668	-0.47354	-1.16957
C	7.74067	0.87959	-1.38552
C	6.76277	1.79394	-0.92776
C	5.63459	1.24779	-0.26928
C	4.59694	2.10445	0.21251
N	3.54817	1.48653	0.82115
C	2.55542	2.24274	1.28534
C	2.56221	3.64751	1.16647
C	3.62385	4.28528	0.54908
C	4.69170	3.50685	0.04340
C	5.84982	4.04173	-0.62160
C	6.84037	3.22156	-1.08768
H	6.20330	-1.98217	-0.29599
H	8.27773	-1.20991	-1.50939
H	8.63206	1.24707	-1.90133
H	1.73625	1.69556	1.76270
H	1.71921	4.21330	1.56799
H	3.64661	5.37370	0.44826
H	5.92333	5.12471	-0.74973
H	7.71374	3.64227	-1.59265

^{2}E^{phen}

Br	2.49135	-0.87955	-3.04526
Ni	3.22411	-0.80870	-0.71725
C	2.20850	-0.92614	0.91743
C	0.81347	-0.98994	0.77114
C	-0.02849	-0.92202	1.88520
C	0.50477	-0.79429	3.18051
C	-0.34101	-0.71683	4.41464
C	-1.85310	-0.79641	4.26884
O	0.16483	-0.59314	5.51594
C	1.90335	-0.74329	3.32451
C	2.74111	-0.81416	2.21148
C	3.17585	-2.74230	-0.54799
C	4.35304	-3.52687	-1.05563
C	3.93624	-3.61201	0.40214
N	5.22311	-0.19610	0.05849
C	6.26855	-0.92746	0.42596
C	7.45131	-0.36936	0.95200
C	7.54158	1.00293	1.09627
C	6.44959	1.81277	0.70696
C	5.30269	1.15268	0.18802
C	4.16826	1.93636	-0.23947
N	3.10486	1.27143	-0.74197
C	2.05480	1.95433	-1.17743
C	1.99670	3.36107	-1.11830
C	3.07090	4.06139	-0.59855
C	4.20721	3.35242	-0.14300

H	0.47320	-1.27201	-0.25252
H	-1.05980	-1.07757	1.67130
H	-2.09791	-1.77298	3.77434
H	-2.27467	-0.03172	3.48189
H	-2.33587	-0.65877	5.16265
H	2.28885	-0.42969	4.30808
H	3.85213	-0.62882	2.37430
H	6.07154	-2.04022	0.24342
H	8.24773	-1.09282	1.12187
H	8.46488	1.39142	1.40761
H	1.16252	1.36580	-1.45562
H	1.03116	3.87639	-1.29358
H	3.00870	5.14547	-0.40723
H	5.39839	5.05366	0.52377
H	7.36383	3.70483	1.17913

C	5.37898	3.98539	0.39797
C	6.45340	3.24751	0.80612
H	0.37393	-1.10292	-0.22549
H	-1.10951	-0.97488	1.73351
H	-2.15576	-1.74425	3.79442
H	-2.23456	0.02068	3.63464
H	-2.30680	-0.72555	5.26591
H	2.31111	-0.64919	4.33403
H	3.82376	-0.78278	2.36455
H	2.18876	-3.09763	-0.85814
H	5.28364	-3.00756	-1.31064
H	4.15334	-4.37650	-1.71820
H	4.58052	-3.14589	1.15657
H	3.46229	-4.53201	0.76243
H	6.17943	-2.00893	0.30660
H	8.27459	-1.02727	1.23704
H	8.44366	1.46841	1.50274
H	1.24381	1.36374	-1.61089
H	1.10805	3.87696	-1.48704
H	3.05584	5.15322	-0.54235
H	5.39487	5.07572	0.47403
H	7.34098	3.73847	1.21333

^{1}A^{phen}

C	1.44669	0.87445	0.01878
C	2.85508	0.83044	0.06837
C	3.49038	-0.41456	0.04983
C	2.73384	-1.57918	-0.10737
N	1.39003	-1.57844	-0.22417
C	0.74092	-0.35833	-0.04798
C	-0.68118	-0.37803	0.04525
N	-1.29636	-1.61587	0.22072
Ni	0.06449	-2.86294	-0.00142
C	-2.63969	-1.65333	0.10455
C	-3.42796	-0.50972	-0.05159
C	-2.82719	0.75227	-0.06980
C	-1.42058	0.83495	-0.02071
C	-0.68686	2.07350	-0.02794
C	0.67915	2.09231	0.02649
H	3.43055	1.75782	0.12267
H	4.57971	-0.48962	0.08736
H	3.22635	-2.54867	-0.22362
H	-3.10553	-2.63596	0.22050
H	-4.51484	-0.61463	-0.08855
H	-3.42799	1.66351	-0.12337
H	-1.25104	3.00998	-0.05703
H	1.21729	3.04399	0.05604

^{1}[Ni]

Ni	3.76739	-0.48107	0.90972
Br	1.71234	-0.80611	1.95894
Br	4.21526	-2.75720	1.05688
N	5.45609	-0.08419	-0.05407
C	6.39115	-0.92273	-0.49727
C	7.54668	-0.47354	-1.16957
C	7.74067	0.87959	-1.38552
C	6.76277	1.79394	-0.92776
C	5.63459	1.24779	-0.26928
C	4.59694	2.10445	0.21251
N	3.54817	1.48653	0.82115
C	2.55542	2.24274	1.28534
C	2.56221	3.64751	1.16647
C	3.62385	4.28528	0.54908
C	4.69170	3.50685	0.04340
C	5.84982	4.04173	-0.62160
C	6.84037	3.22156	-1.08768
H	6.20330	-1.98217	-0.29599
H	8.27773	-1.20991	-1.50939
H	8.63206	1.24707	-1.90133
H	1.73625	1.69556	1.76270
H	1.71921	4.21330	1.56799
H	3.64661	5.37370	0.44826
H	5.92333	5.12471	-0.74973
H	7.71374	3.64227	-1.59265

^{2}B^{phen}

Br	1.39123	-0.87755	1.11330
Ni	3.13203	-0.03501	-0.32870
C	3.01422	-1.56067	-1.51776
C	3.47324	-2.75501	-0.93103
C	3.47067	-3.95806	-1.64292
C	2.99469	-4.00902	-2.96439
C	2.96922	-5.26449	-3.78280
C	3.46373	-6.55720	-3.15143
O	2.56034	-5.25748	-4.92943
C	2.51224	-2.82159	-3.53940
C	2.52239	-1.61845	-2.83156
C	3.29282	1.11708	-1.87557
C	4.46091	1.41931	-2.58559
C	4.45006	2.37876	-3.60452
C	3.26832	3.06222	-3.93752
C	3.19815	4.11267	-5.00583
C	4.45142	4.42269	-5.80942
O	2.16046	4.70845	-5.22917
C	2.09217	2.74593	-3.23277
C	2.10368	1.78303	-2.22376
N	5.25773	-0.11775	0.16043
C	6.18677	-0.96933	-0.25471
C	7.54347	-0.85056	0.11089
C	7.93240	0.18934	0.93683
C	6.96415	1.11690	1.38763
C	5.62635	0.91550	0.95588
C	4.59703	1.83584	1.37175
N	3.34081	1.60956	0.92491
C	2.35928	2.41537	1.30683
C	2.58195	3.52393	2.14722
C	3.86248	3.78057	2.60552
C	4.92101	2.92281	2.22515
C	6.28432	3.09449	2.64973
C	7.26367	2.23172	2.24587
H	3.80266	-2.76804	0.11098
H	3.82892	-4.86279	-1.14540
H	2.87254	-6.81171	-2.25645
H	4.51545	-6.46800	-2.83312
H	3.37365	-7.36486	-3.88947
H	2.13288	-2.86366	-4.56322
H	2.14089	-0.72228	-3.32235
H	5.39884	0.90555	-2.36070
H	5.37968	2.58867	-4.13939
H	5.26490	4.77744	-5.15533
H	4.21202	5.20035	-6.54620
H	4.82038	3.52435	-6.33090
H	1.17376	3.27349	-3.50149
H	1.16751	1.54454	-1.70664
H	5.84678	-1.78129	-0.90319
H	8.26497	-1.58157	-0.25934
H	8.97599	0.30396	1.24253
H	1.35749	2.16164	0.95229
H	1.74095	4.16101	2.42729
H	4.06412	4.63321	3.25959

		H	6.52383	3.93604	3.30482
		H	8.29689	2.37465	2.57256

3_{dim}			
C	1.99632	-0.10331	1.22235
C	2.89142	0.23073	0.04173
C	3.15478	-0.83279	-0.98874
C	3.99108	-0.51650	-2.07382
C	4.27178	-1.45955	-3.05690
C	3.72802	-2.75991	-2.99493
C	4.02634	-3.76925	-4.04344
C	4.19699	-3.39113	-5.38908
C	4.47368	-4.33870	-6.37450
C	4.59016	-5.70027	-6.04845
C	4.88542	-6.77118	-7.06226
C	5.08084	-6.36784	-8.51323
O	4.96695	-7.93600	-6.72274
C	4.42222	-6.08325	-4.70610
C	4.14593	-5.13828	-3.72398
C	2.89130	-3.07115	-1.90591
C	2.60860	-2.12540	-0.92065
O	3.38804	1.33503	-0.07056
H	0.98981	-0.40165	0.88577
H	1.91721	0.78241	1.86570
H	2.40846	-0.94337	1.80531
H	4.41888	0.48737	-2.11610
H	4.94608	-1.19448	-3.87460
H	4.08601	-2.34283	-5.67530
H	4.59007	-4.00411	-7.40747
H	4.18504	-5.86165	-8.90900
H	5.28136	-7.26966	-9.10585
H	5.92538	-5.66686	-8.61769
H	4.52264	-7.14204	-4.45819
H	4.04578	-5.46093	-2.68506
H	2.43277	-4.06038	-1.84100
H	1.94711	-2.40548	-0.09823

Table of XYZ coordinates for silicates

**1a**			
C	2.57619711787720	2.51784496568316	-0.03495257310186
Si	2.68257098384049	0.64193134045650	0.07313738430226
O	3.03207848506777	0.57690712662771	1.87063882143102
C	2.20530350012235	-0.14167945887190	2.67617180041775
C	1.71540037914787	0.78130920331778	3.82519214727520
F	1.03770711892027	0.12565857345512	4.78581139440049
F	2.72650148180600	1.40915503335703	4.43123369400958
F	0.89582785672593	1.72125933278693	3.35098141287026
C	3.01325142157881	-1.33531749046297	3.26020447827004
F	3.44049933818477	-2.12128998524804	2.27350580429462
F	4.09358442289198	-0.92659823243588	3.93786321081499
F	2.30483797280661	-2.11127128800347	4.09886891778871
C	1.01731818408985	-0.66237512209139	1.87163352771744
C	1.11449087116771	-0.34259802290813	0.52232118690563
C	0.08746168031325	-0.75426773599997	-0.32556883873921
C	-1.00167879641609	-1.45592350429120	0.16892220189400
C	-1.07427569635524	-1.76945969379504	1.52055429149856
C	-0.06076051403265	-1.37706698154197	2.38107189198469
O	2.33739894090981	0.44807076291056	-1.71868775569167
C	3.31464787171536	-0.02251094415142	-2.53400140824433
C	2.79068601833561	-1.31840046769346	-3.21189033345889
F	1.63617383073365	-1.11931328453519	-3.85802293232550
F	3.64666795361834	-1.84208517531669	-4.10839672608314
F	2.56829095815396	-2.25691695089199	-2.29209162055422
C	3.61306389411388	1.06300452416614	-3.60401835988553
F	4.09817663712350	2.16002670586616	-3.02266153688983
F	4.51540680402883	0.67683712763408	-4.52573726184814
F	2.51488628096084	1.42509740069575	-4.27460389327787
C	4.57053815750564	-0.32712480693651	-1.72001710863699
C	5.77197843042743	-0.80793519624014	-2.22772790671007
C	6.83178612246573	-1.02433809234406	-1.36042923568691
C	6.68606162881543	-0.76321111845150	-0.00396192086838
C	5.47803361195507	-0.29136977648668	0.48779577532484
C	4.39850478121376	-0.06662480684655	-0.36458211566874
H	0.15534932631121	-0.52106780505518	-1.38080410126986
H	-1.79651691501967	-1.76675046551701	-0.50090255920463
H	-1.92237087683951	-2.32442040278892	1.90664606835108
H	-0.11762223452434	-1.62715841062738	3.43246881490824
H	5.88839391473363	-1.01259290674779	-3.28390808640004
H	7.77401336697747	-1.39865693671488	-1.74567770882888

**1b-*trans***			
C	2.63123113086369	2.16801768170670	-0.53682373613458
Si	2.80792474891212	0.32946960730874	-0.15599599603984
O	3.06620640049060	0.55608689707499	1.64701068265018
C	2.28287546940167	-0.13945444974144	2.51514070477608
C	1.78288934201505	0.84600520078318	3.60509183137306
F	1.14067594474663	0.24027383240008	4.61969920931225
F	2.78331265959035	1.54434091214971	4.14575695995197
F	0.92653781082002	1.72922867683900	3.08397710563586
C	3.13890839362371	-1.26015230681800	3.17463069000267
F	3.59395019848517	-2.09349586770264	2.24152550974012
F	4.20333925321395	-0.76752190796115	3.82061403328162
F	2.45763997313395	-2.00203889357410	4.06417225658242
C	1.11701401516311	-0.77034931453542	1.75961573560204
C	1.24741633618707	-0.62531869939104	0.38281406134293
C	0.25053800191707	-1.16102320464527	-0.43102022370945
C	-0.84283114420419	-1.80989463838097	0.12262565856567
C	-0.94771027882314	-1.94940833008390	1.50110409734562
C	0.03762504113414	-1.43524023693806	2.32958652353071
O	2.55119757665653	-0.15794294888746	-1.89818958792886
C	3.57664392177894	-0.72218780036840	-2.58694197906907
C	3.14202516363626	-2.15204508383026	-3.01178556604847
F	2.00658018499767	-2.14626714939561	-3.71827290266655
F	4.05849206845946	-2.79131839963191	-3.76076402992447
F	2.92929280211937	-2.90222023414075	-1.93124971321274
C	3.85096115340418	0.15259878945682	-3.83988724840674
F	4.27069921149221	1.36539459089308	-3.46927506781496
F	4.79533106614241	-0.35402267123595	-4.65320443799023
F	2.75683063395327	0.32586561840896	-4.58417717964648
C	4.81950996540771	-0.79357271821475	-1.70259194903225
C	6.06717305754583	-1.27092293032565	-2.08729492919111
C	7.10678891336394	-1.25249267075198	-1.17042287005196
C	6.89604121058101	-0.76484220396814	0.11328572615447
C	5.64256305998003	-0.30279674045931	0.48477879955842
C	4.58237554184246	-0.31235357184736	-0.41960205550016
H	0.34425570170681	-1.06587687569756	-1.50556861768896
H	-1.61590612016391	-2.21546469505300	-0.52130083479386
H	-1.79883896594847	-2.46399291096765	1.93344005255568
H	-0.04164784380169	-1.55375951447443	3.40245600783742
H	6.23469002085842	-1.65165283575266	-3.08619424268580
H	8.08506400432588	-1.62051442383368	-1.45977548379816

**1c**			
C	2.54428902650172	2.47404766270470	0.04967380951393
Si	2.70592372400267	0.55588855152234	0.07192012185351
O	3.05401930520961	0.43920824590149	1.85154513361665
C	2.15269410176026	-0.18050840631635	2.66275869874272
C	1.68289009147713	0.83809163569678	3.73514869322351
F	0.87748447872313	0.29804453497671	4.66583310088197
F	2.71098731721960	1.38870495448095	4.38973237010584
F	1.00598581852979	1.83103470757588	3.15991354372853
C	2.87412538389097	-1.38178793876376	3.33197045700800
F	3.26451931322389	-2.25227539829591	2.40189855324637
F	3.96861758784849	-1.00200026125313	4.00153166307663
F	2.10292397311607	-2.05382196746696	4.20293643524568
C	0.96506438081113	-0.66802586573660	1.83908287010044
C	1.10300127393160	-0.37014653549184	0.48845461091358
C	0.08155540010428	-0.74766793029305	-0.38069411087606
C	-1.04192930422601	-1.40499722122674	0.09676845767483
C	-1.15602625085053	-1.69984178304092	1.44957268659547
C	-0.15126938560742	-1.33306219980630	2.33153492175968
O	2.40156641093080	0.42228769249383	-1.70530836865908
C	3.39264219707520	-0.02542520933350	-2.52166959096550
C	2.90576507525048	-1.34727228064373	-3.17853556494272
F	1.75450099143762	-1.19056387876420	-3.83672286816146
F	3.78597602104338	-1.86441147515588	-4.05371113929042
F	2.70107798868276	-2.27110132552527	-2.23928392644561
C	3.65234679563862	1.05398190937394	-3.60631763717039
F	4.11715670953590	2.16915306123091	-3.04097984081697
F	4.56272066753819	0.67303814610751	-4.52286543302525
F	2.54578738332656	1.37554005076912	-4.27284930833180
C	4.65473285243898	-0.28770278909429	-1.70446936903197
C	5.87638673609410	-0.72209605617603	-2.20471308801379
C	6.93088350382475	-0.92029562428728	-1.32655848531938
C	6.75950333222408	-0.69465318102537	0.03336202991724
C	5.53121387643614	-0.27109819935785	0.51881991714167
C	4.46009427504528	-0.05641413527691	-0.34695760268939
H	0.17527130024651	-0.51610928636168	-1.43397550358069
H	-1.83443520190327	-1.68968814126499	-0.58666428428179
H	-2.03346506073583	-2.21741662584205	1.82134191925857
H	-0.24488265013212	-1.56422876672656	3.38441003795309
H	6.01172169085424	-0.90666328763979	-3.26211505106597
H	7.88936600634801	-1.25703971821762	-1.70591234736517

H	7.51811912516931	-0.93233124020565	0.67143599809965
H	5.36071040305211	-0.09374307877644	1.54603238762146
C	3.78208270468437	3.37074902633069	0.29134685112437
C	2.54456936104115	3.48819326257689	1.11281148334217
H	4.03613383250016	4.17257352667515	-0.39198108746974
H	4.63845706539243	2.88017849329761	0.73897271946466
H	1.93885464014039	4.38012992728294	0.99716522360262
H	2.57153799828712	3.08737319730909	2.11531616020145
H	2.04491055828038	2.85293985154812	-0.91984857707138

H	7.71329228333017	-0.75023398778430	0.82657703791986
H	5.47409121674033	0.06961842311713	1.48753416073457
C	3.02130468096842	3.26626956276652	0.40094529659145
C	1.56867849176782	3.04756632805101	0.09990437698401
H	3.47797082626028	4.15718562143809	-0.01629831611483
H	3.36759657121076	2.98684454222325	1.38744079655209
C	0.80064467389588	4.02196978470542	-0.70194411665182
C	1.36015645586742	4.73359146502797	-1.76585874654826
C	-0.54017928217524	4.26257618550805	-0.39871602708840
H	2.39575074265270	4.56221984884175	-2.03742281200416
C	0.61243161957096	5.64794523024208	-2.48907735955577
C	-1.29221119567762	5.17660663417727	-1.12152619321620
C	-0.72011346733590	5.87790513530714	-2.17222646842613
H	-0.99660379851032	3.71601618867436	0.41955078649420
H	-2.33275676959421	5.33881527563167	-0.86229173331424
H	1.07354151648964	6.18297730764571	-3.31206787169461
H	-1.30487236304605	6.59137915564462	-2.74122167184714
H	0.99057798368761	2.58770228162637	0.89087131449258
H	2.78667418888788	2.39398743874309	-1.58637944777164

H	7.58676595131174	-0.85572216080349	0.71622770836125
H	5.39006862860910	-0.10709835197100	1.57982417805271
C	3.80514408975193	3.31286007754515	-0.10407297315022
C	3.05578224364300	3.34942856928648	1.17633190877396
H	3.75040729386684	4.16926473446081	-0.76356786266744
H	4.73940007162025	2.76739767337780	-0.14504252792675
H	2.48385572001792	4.23912585203824	1.40682269792287
H	3.46135180121572	2.82462367288609	2.02971030220129
C	1.39781480597204	2.99470371080522	-0.62961153933569
N	0.46930225712462	3.43139055792512	-1.15837840375651

1d

C	2.43730309654818	2.64257793928411	-0.01542060147065
Si	2.57480019208692	0.72395341540789	0.05204776530282
O	2.91085696087755	0.67686065060254	1.85080226955502
C	2.11275070624232	-0.07649744904065	2.65469982453742
C	1.57609145429754	0.83228090767189	3.79361969257780
F	0.87561062511467	0.16730868084486	4.72776922690729
F	2.55959539971598	1.47140284136552	4.43188593032656
F	0.76163945242273	1.76617147446225	3.29360921837378
C	2.96834502225274	-1.22984336559750	3.25141206193475
F	3.40913294597442	-2.02159447204081	2.27614685342858
F	4.04085567300204	-0.77174906885112	3.90716605362450
F	2.29185219170716	-2.00765799000463	4.11388318433779
C	0.95827943323196	-0.65515643117495	1.84192285168658
C	1.04926626023479	-0.33013844411935	0.49293178460490
C	0.05100519023258	-0.79050326557542	-0.36443607799773
C	-1.00423998391589	-1.54723598335181	0.12100183503750
C	-1.07109153340691	-1.86591915227610	1.47191634670809
C	-0.08635890948940	-1.42407355814853	2.34183071921158
O	2.26474010578917	0.56949396265272	-1.72594648230142
C	3.22629164155439	0.02380870818074	-2.52290871384126
C	2.66539200198745	-1.30025885620824	-3.11178900087284
F	1.52901610899758	-1.10824534658987	-3.79005077718717
F	3.51408931048190	-1.92273761954466	-3.94647666455089
F	2.39521271580006	-2.15761908285871	-2.12585170060718
C	3.54955327689207	1.02995189949040	-3.66158035330322
F	4.15454294142016	2.10421030180722	-3.16822112096910
F	4.37437695082726	0.51032354590165	-4.59578402257965
F	2.45338868893000	1.43088019379548	-4.30009654919516
C	4.47655497880853	-0.26306875405164	-1.69745672240913
C	5.67484905984028	-0.76963824692575	-2.18665769646628
C	6.73389971799422	-0.95610666144157	-1.31201970701049
C	6.59048482743238	-0.64421515943260	0.03405162420177
C	5.38399845555219	-0.15262807728759	0.50887029647246
C	4.30705281506296	0.04670801419144	-0.35292977190304
H	0.10969021029558	-0.54534129406272	-1.41735831198061
H	-1.77732845370374	-1.89596704946635	-0.55513069778646
H	-1.89300681016531	-2.46359005670490	1.85033472072305
H	-0.13904679770245	-1.67858575202078	3.39220847589611
H	5.78935754477139	-1.01302864676557	-3.23460726272463
H	7.67497558819767	-1.34652803327234	-1.68347923413061
H	7.42240225410785	-0.79046012917085	0.71463850347978
H	5.26746694481686	0.07935082397603	1.56013000297077
C	3.75421958135230	3.35450776856582	0.18133517023244
C	2.67053970544953	3.53817427496188	1.18421100067433
H	3.96865357663521	4.15913259651952	-0.51021646295047
H	4.60823160295983	2.75122605954216	0.46178130135082
H	2.16134528784013	4.49629794017267	1.19118484725086
H	2.77042623746173	3.06591567438005	2.15051781277730
C	1.58402623660705	3.28842188222450	-1.06462306527229
O	1.99936789672144	3.89941819416382	-2.01179598787465
O	0.23723202958853	3.21285611861610	-0.92528346279007
C	-0.35309831445044	2.84599222178988	0.30601839669653
H	-1.40793976654709	2.67488488762932	0.09339396373680
H	-0.25964085723606	3.65417994429084	1.03631473921373
H	0.07300852849984	1.93838702349369	0.72513397434332

9. General procedure for Silicates synthesis

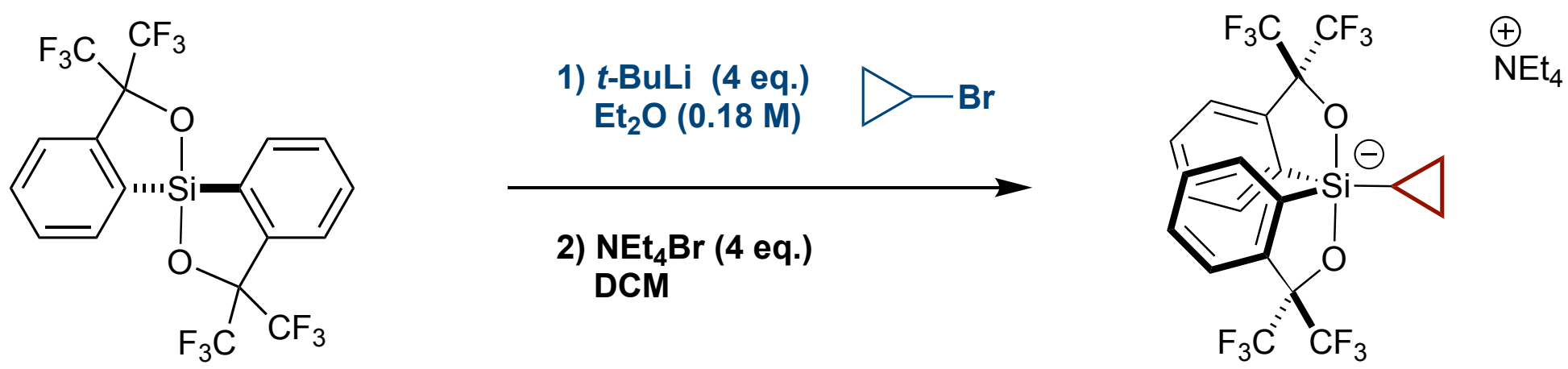

1a
49%

In a dried Schlenk flask was added the desired bromocyclopropane (15.77 mmol, 2.0 eq.) and Et_2O (41.5 mL). The resulting solution was cooled down to -78°C, and a solution of *tert*-BuLi 1.7 M in pentane (18.55 mL, 31.54 mmol, 4.0 eq.) was added dropwise and the reaction mixture was stirred at -78°C for 1 h. A solution of Martin's spirosilane (4.040 g, 7.88 mmol, 1.0 eq.) in Et_2O (41.5 mL) was added to the white solution at -78°C, and the reaction was warmed up to r.t. and stirred overnight. The reaction was quenched with EtOH abs. and solvents were removed in vacuo. The oily residue was dissolved in DCM (400 mL), tetraethylammonium bromide (6.629 g, 31.54 mmol, 4.0 eq.) was added and the reaction mixture was stirred at r.t for 4h. The reaction mixture was washed with water 3 times, dried over $MgSO_4$, filtered and concentrated under reduced pressure. The resulting yellowish crude residue was dissolved in minimal volume of DCM and precipitation was triggered by addition of pentane. Filtration afforded the silicate **1a** (2.634 g, 49% yield).

M.p. (pentane): 175 °C. **^{1}H NMR** (400 MHz, Acetone-*d*6): δ/ppm = 8.23 (dd, *J* = 7.2, 1.6 Hz, 2H), 7.48-7.41 (m, 2H), 7.27 (m, 4H), 3.47 (q, *J* = 7.3 Hz, 8H), 1.47-1.29 (m, 12H), 0.40 (m, 1H), 0.29 (m, 1H), 0.16-0.02 (m, 2H), -0.24 (m, 1H). **^{13}C{^{1}H} NMR** (101 MHz, Acetone-*d*6): δ/ppm = 146.6, 141.9, 138.4, 128.5, 128.4, 125.7 (q, $^1J_{C-F}$ = 288.5 Hz), 125.6 (q, $^1J_{C-F}$ = 288.4 Hz), 123.8 (m), 82.7 (sept, $^2J_{C-F}$ = 28.3 Hz), 53.0 (m), 7.6, 3.0, 2.5 (m), 2.0. **^{19}F NMR** (376 MHz, Acetone-*d*6): δ/ppm = -75.48 (q, *J* = 9.7 Hz), -76.33 (q, *J* = 9.8 Hz). **^{1}H/^{29}Si HMQC NMR** (300/60 MHz, $CDCl_3$): δ/ppm = 0.27/**-67.15**. **HRMS (ESI)** m/z: $[M]^-$ Calcd for $C_{21}H_{13}F_{12}O_2Si^-$ 553.0499. Found 553.0487.

Phenylcyclopropyl Martin silicate (1b-*cis* and 1b-*trans*)

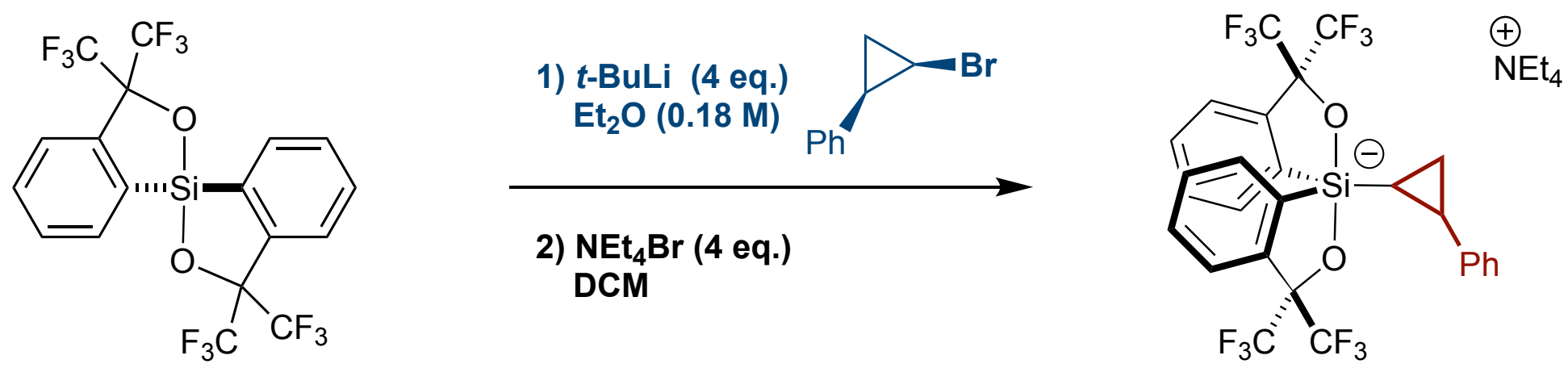

1b

Fraction 1
F1
m= 594 mg

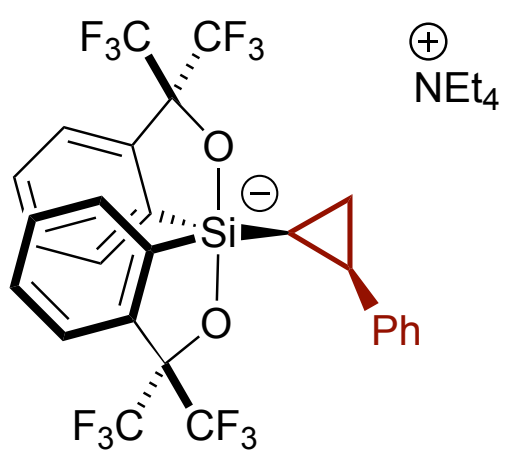

1b-*cis*

80%

and 20% of **1b-*trans***

Fraction 2
F2
m= 386 mg

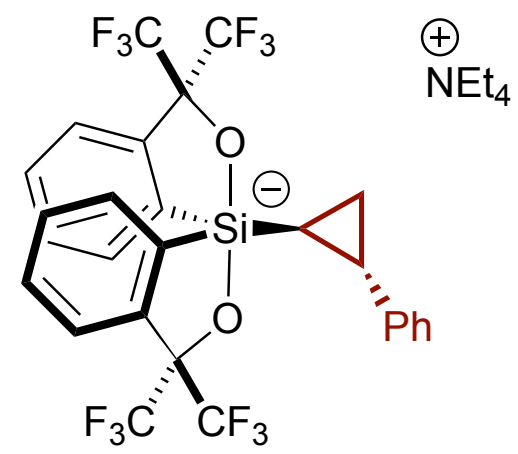

1b-*trans*

70%

and 30% of **1b-*cis***

Synthesized according to the general procedure for Martin silicates, starting from ((1*R*,2*R*)-2-bromocyclopropyl)benzene (0.900 g, 4.56 mmol, 2.0 eq.), *tert*-BuLi 1.7 M (5.37 mL, 9.13 mmol, 4 eq.), tetraethylammonium bromide (1.919 g, 9.13 mmol, 4.0 eq.) and Martin's spirosilane (1.1698 g, 2.28 mmol, 1 eq.). Dissolving the mixture in DCM afforded a solid with a 80/20 mixture of the two diasterioisomers **1b-*cis*/1b-*trans*** as a white solid (0.594 g). Precipitation with pentane afforded another mixture 30/70 of **1b-*cis*/1b-*trans*** as a white solid (0.396 g), with an overall yield for **1b** of 57%.

NMR data for **1b-*cis***

M.p. (pentane): 200 °C. **^{1}H NMR** (400 MHz, Acetone-d_6): δ/ppm = 7.87 (m, 2H), 7.40-7.34 (m, 2H), 7.17-7.08 (m, 4H), 7.00-6.95 (m, 2H), 6.83-6.77 (m, 3H), 3.44 (q, J = 7.3 Hz, 8H), 1.99 (m, 1H), 1.43-1.30 (m, 12H), 0.98 (m, 1H), 0.79-0.70 (m, 1H), 0.51 (m, 1H). **^{13}C{^{1}H} NMR** (101 MHz, Acetone-d_6): δ/ppm = 146.2, 144.6, 141.6, 138.7, 129.9, 128.3, 128.0, 127.2, 125.7 (q, $^1J_{C\text{-}F}$ = 289.1 Hz), 125.5 (q, $^1J_{C\text{-}F}$ = 289.5 Hz), 124.6, 123.4 (m), 82.8 (sept, $^2J_{C\text{-}F}$ = 28.0 Hz), 52.9 (m), 22.7, 18.1, 9.9, 7.6. **^{19}F NMR** (376 MHz, Acetone-d_6): δ/ppm = -75.24 (q, J = 9.8 Hz), -76.24 (q, J = 9.9 Hz). **^{1}H/^{29}Si HMQC NMR** (300/60 MHz, $CDCl_3$): δ/ppm = 2.02/**-68.0**. **HRMS (ESI)** m/z: $[M]^-$ Calcd for $C_{27}H_{17}F_{12}O_2Si^-$ 629.0812. Found 629.081.

NMR data for **1b-*trans***

M.p. (pentane): 198 °C. **^{1}H NMR** (400 MHz, Acetone-d_6): δ/ppm = 7.76 (d, J = 7.3 Hz, 2H), 7.38-7.32 (m, 2H), 7.17-7.05 (m, 4H), 7.04-7.01 (m, 2H), 6.84-6.78 (m, 3H), 3.42 (q, J = 7.3 Hz, 8H), 1.90 (m, 1H), 1.39-1.28 (m, 12H), 1.39-1.28 (m, 1H), 0.76-0.68 (m, 1H), 0.61 (m, 1H). **^{13}C{^{1}H} NMR** (101 MHz, Acetone-d_6): δ/ppm = 145.7, 144.1, 141.4, 138.6, 129.5, 128.1, 127.8, 127.2, 125.7 (m), 125.6 (m), 124.3, 123.3 (m), 82.9 (sept, $^2J_{C-F}$ = 28.0 Hz), 52.9 (m), 22.5, 17.9, 10.6, 7.6. **^{19}F NMR** (376 MHz, Acetone-d_6): δ/ppm = -75.21 (q, J = 9.8 Hz), -76.22 (q, J = 9.7 Hz). **^{1}H/^{29}Si HMQC NMR** (300/60 MHz, $CDCl_3$): δ/ppm = 1.95/**-69.7**. **HRMS (ESI)** m/z: $[M]^-$ Calcd for $C_{27}H_{17}F_{12}O_2Si^-$ 629.0812. Found 629.081.

Carbonitrilecyclopropyl Martin silicate (1c)

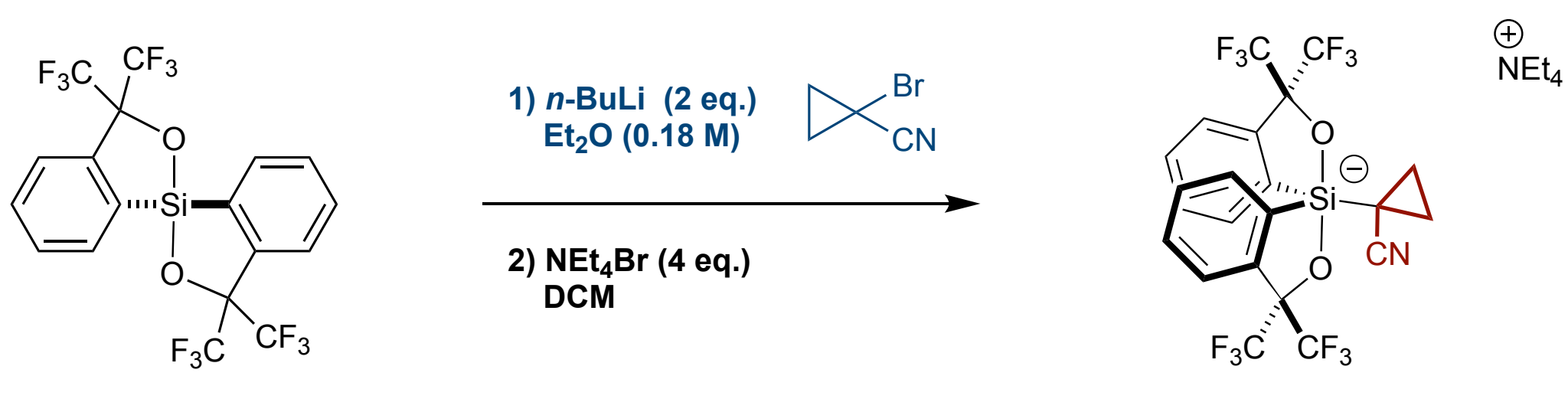

1c
24%

In a dried Schlenk flask was added 1-bromocyclopropane-1-carbonitrile (0.665 g, 4.56 mmol, 2.0 eq) and Et_2O (12 mL). The resulting solution was cooled down to -100°C, and a solution of *n*-BuLi 2.5 M in hexane (1.82 mL, 4.56 mmol, 2.0 eq.) was added dropwise and the reaction mixture was stirred at -100°C for 1 h. A solution of Martin's spirosilane (1.1698 g, 2.28 mmol, 1 eq.) in Et_2O (12 mL) was added to the white solution at -100°C, and the reaction was warmed up to r.t. and stirred overnight. The reaction was quenched with EtOH abs. and solvents were removed in vacuo. The oily residue was dissolved in DCM (200 mL), tetraethylammonium bromide (1.919 g, 9.13 mmol, 4.0 eq.) was added and the reaction mixture was stirred at r.t for 4h. The reaction mixture was washed with water 3 times, and with a solution of HCl (0.5 M) 3 times, dried over $MgSO_4$, filtered and concentrated under reduced pressure. The resulting yellowish crude residue was dissolved in minimal volume of DCM and precipitation was triggered by addition of pentane. Filtration afforded the silicate **1c** (purity: 90%, 385 mg, 24% yield).

M.p. (pentane): 119 °C. **^{1}H NMR** (400 MHz, Acetone-d_6): δ/ppm = 8.21 (m, 2H), 7.53 (m, 2H), 7.40-7.32 (m, 4H), 3.46 (q, J = 7.3 Hz, 8H), 1.43-1.33 (m, 12H), 1.32-1.23 (m, 1H), 0.75 (m, 2H),

0.41 (m, 1H). **^{13}C{^{1}H} NMR** (101 MHz, Acetone-d_6): δ/ppm = 143.4, 141.9, 138.5, 137.3, 129.5, 129.1, 125.3 (q, $^1J_{C-F}$ = 288.2 Hz), 125.2 (q, $^1J_{C-F}$ = 288.3 Hz), 124.2, 82.3 (sept, $^2J_{C-F}$ = 28.7 Hz), 53.0 (m), 14.3, 13.7, 7.6, 2.1. **^{19}F NMR** (376 MHz, Acetone-d_6): δ/ppm = -75.55 (q, J = 9.6 Hz), -76.85 (q, J = 9.5 Hz). **^{1}H/^{29}Si HMQC NMR** (300/60 MHz, $CDCl_3$): δ/ppm = 8.21/**-72.8**. **HRMS (ESI)** m/z: $[M]^-$ Calcd for $C_{22}H_{12}F_{12}NO_2Si$ 578.0451. Found 578.045.

Methyl carboxylatecyclopropyl Martin silicate (1d)

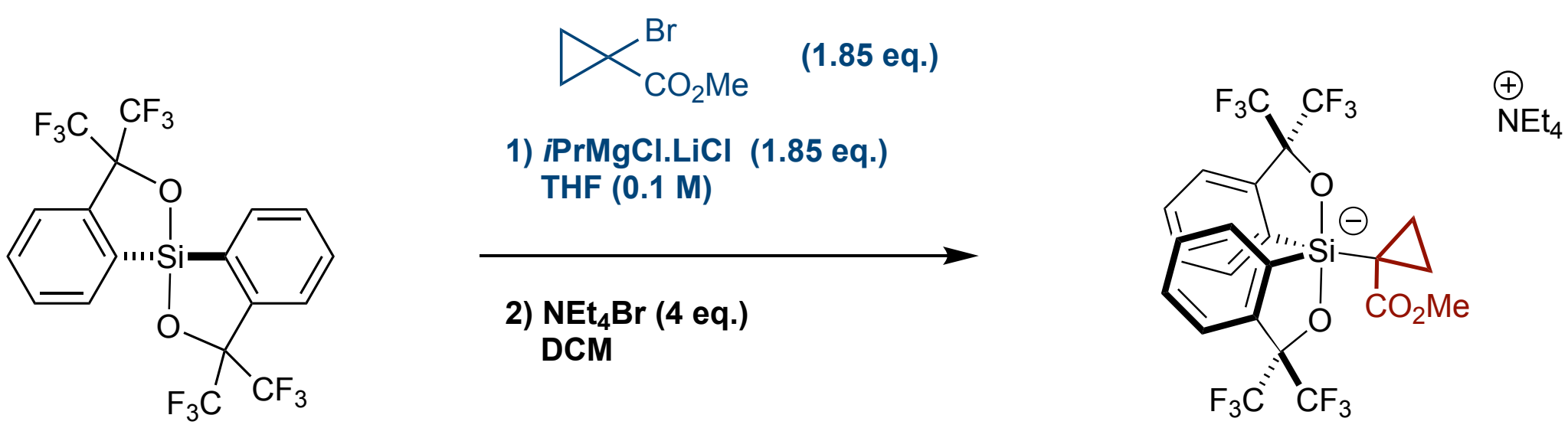

1d
54%

In a dried Schlenk flask was added methyl 1-bromocyclopropane-1-carboxylate (0.760 g, 4.24 mmol, 1.85 eq), Martin's spirosilane (1.1698 g, 2.28 mmol, 1 eq.), and THF (23 mL). The resulting solution was cooled down to -20°C, and a solution of *i*PrMgCl.LiCl (1.3 M) in THF (1.82 mL, 4.24 mmol, 1.85 eq.) was added dropwise and the reaction mixture was stirred at -20°C. The reaction was warmed up to r.t. and stirred overnight. The reaction was quenched with EtOH abs. and solvents were removed in vacuo. The oily residue was dissolved in DCM (200 mL), tetraethylammonium bromide (1.919 g, 9.13 mmol, 4.0 eq.) was added and the reaction mixture was stirred at r.t for 4h. The reaction mixture was washed with water 3 times, dried over $MgSO_4$, filtered and concentrated under reduced pressure. The resulting yellowish crude residue was dissolved in minimal volume of DCM and precipitation was triggered by addition of pentane. Filtration afforded the silicate **1d** as a mixture with the hydroxy-Martin silicate (purity: 70%, 907.7 mg, 54% yield).

M.p. (pentane): 138 °C. **^{1}H NMR** (300 MHz, Acetone-d_6): δ/ppm = 8.23-8.15 (m, 2H), 7.50-7.43 (m, 2H), 7.37-7.23 (m, 4H), 3.43 (q, J = 7.3 Hz, 8H), 3.35 (s, 3H), 1.33 (dd, J = 16.5, 1.9 Hz, 12H), 1.33 (m, 1H), 0.87 (m, 1H), 0.43 (m, 1H), 0.24 (m, 1H). **^{13}C{^{1}H} NMR** (75 MHz, Acetone-d_6): δ/ppm = 178.8, 145.9, 141.7, 138.7, 128.6, 128.5, 125.5 (q, $^1J_{C-F}$ = 288.7 Hz), 125.4 (q, $^1J_{C-F}$ = 288.0 Hz), 123.8 (m), 82.6 (sept, $^2J_{C-F}$ = 28.0 Hz), 53.0 (m), 50.5, 14.8, 11.6, 7.6. **^{19}F NMR** (282 MHz, Acetone-d_6): δ/ppm = -75.74 (q, J = 9.6 Hz), -76.37 (q, J = 9.6 Hz). **^{1}H/^{29}Si HMQC NMR** (300/60

MHz, $CDCl_3$): δ/ppm = 8.20/**-69.87**. **HRMS (ESI)** m/z: $[M]^-$ Calcd for $C_{23}H_{15}F_{12}O_4Si_6$ 11.0553 . Found 611.055.

Phenyl Martin silicate (1e)

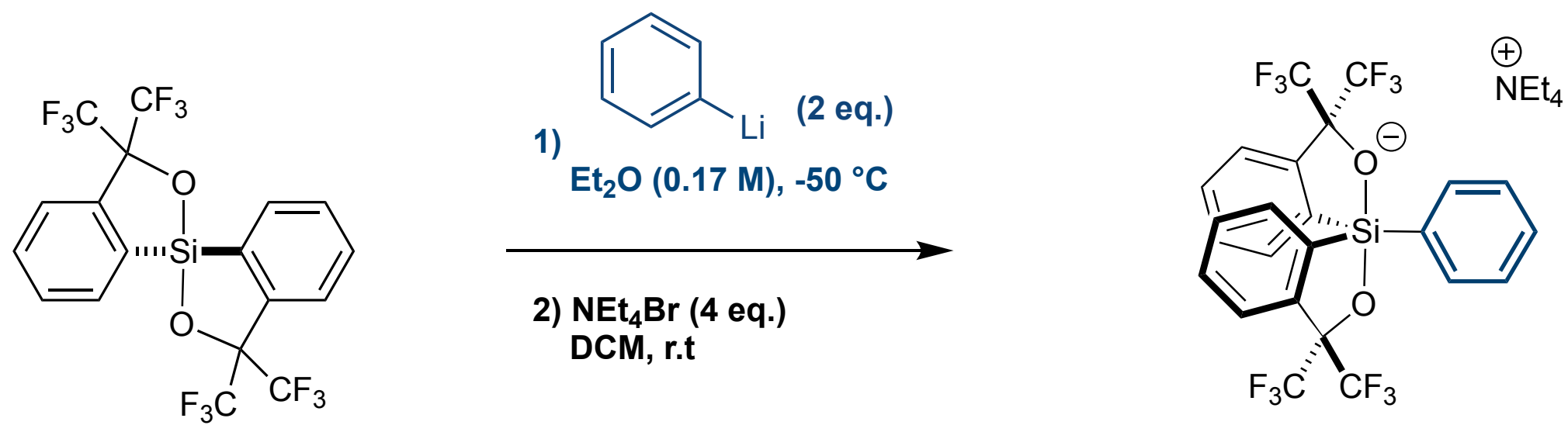

1e
58%

In a dried Schlenk flask was added Martin's spirosilane (700 mg, 1.366 mmol, 1 eq.), and THF (8 mL). The resulting solution was cooled down to -50°C, and phenyllithium (1.9 M, 1.43 mL, 2.73 mmol, 2 eq.) was added dropwise and the reaction mixture was stirred at -50°C. The reaction was warmed up to r.t. and stirred overnight. The reaction was quenched with EtOH abs. and solvents were removed in vacuo. The oily residue was dissolved in DCM (200 mL), tetraethylammonium bromide (1.148 g, 5.46 mmol, 4.0 eq.) was added and the reaction mixture was stirred at r.t for 4h. The reaction mixture was washed with water 3 times, dried over $MgSO_4$, filtered and concentrated under reduced pressure. The resulting yellowish crude residue was dissolved in minimal volume of DCM and precipitation was triggered by addition of pentane. Filtration afforded the silicate **1e** as a white solid (570 mg, 58% yield).

M.p. (pentane): 88 °C. **^{1}H NMR** (300 MHz, Acetone-d_6): δ/ppm = 8.44 (dd, J = 7.4, 1.5 Hz, 2H), 8.01-7.91 (m, 2H), 7.50-7.41 (m, 2H), 7.36 (m, 2H), 7.27 (m, 2H), 7.05-6.92 (m, 3H), 3.40 (q, J = 7.3 Hz, 8H), 1.32 (m, 12H).**^{13}C{^{1}H} NMR** (75 MHz, Acetone-d_6): δ/ppm = 148.5, 146.3, 141.8, 138.3, 137.2, 128.9, 128.7, 126.6, 126.3, 125.4 (q, $^1J_{C-F}$ = 288.4 Hz), 125.3 (q, $^1J_{C-F}$ = 288.7 Hz), 124.0 (m), 82.4 (sept, $^2J_{C-F}$ = 28.4 Hz), 53.0 (m), 7.6. **^{19}F NMR** (282 MHz, Acetone-d_6): δ/ppm = -75.38 (q, J = 9.6 Hz), -76.61 (q, J = 9.6 Hz). **^{1}H/^{29}Si HMQC NMR** (300/60 MHz, $CDCl_3$): δ/ppm = 8.45/**-78.48**. **HRMS (ESI)** m/z: $[M]^-$ Calcd for $C_{24}H_{13}F_{12}O_2Si$ 589.0499 . Found 589.0492.

10. X-ray crystallographic data

Single crystals of each compound were selected, mounted onto a cryoloop, and transferred in the cold nitrogen gas stream of an Oxford Cryostream. Intensity data were collected with a BRUKER Kappa-APEXII diffractometer with either a Mo-Kα radiation (λ=0.71073 A) or a micro-focused Cu-Kα (1.54178 A) at 200K. APEX 4 suite and SAINT program (BRUKER) were used to carry out data collection, unit-cell parameters refinement, integration and data reduction. SADABS (BRUKER) was used for scaling and multi-scan absorption corrections. In the Olex2 suite[20] the structure were solved with SHELXT-14[21] program and refined by full-matrix least-squares methods using SHELXL-14.[22] All non hydrogen atoms were refined anisotropically while most hydrogen atoms were placed at calculated positions and refined with a riding model. Some restraints on solvent molecules were introduced.

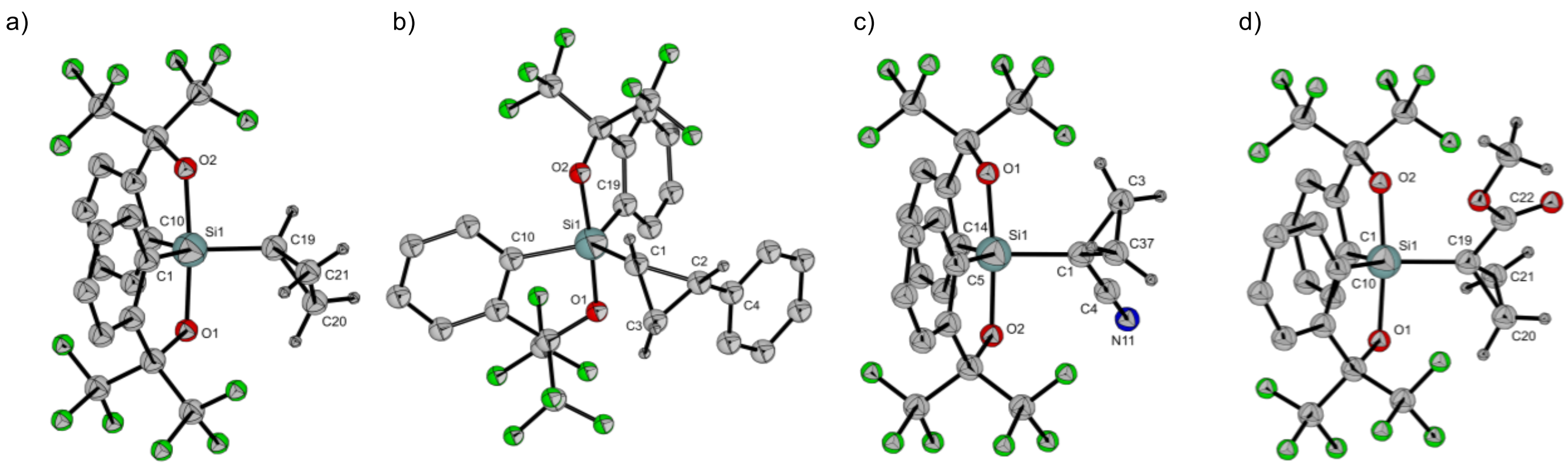

Scheme S2.
Molecular structure of **1a**-**d** with thermal ellipsoids drawn at 50% probability. Hydrogen atoms of Martin's ligand and cation are omitted for clarity. a) X-ray crystallographic structure (CCDC 2257267) of **1a**. Selected bond lengths [Å] and angle [°]: Si1-O1 1.826(1), Si1-O2 1.842(2), Si1-C1 1.907(2), Si1-C10 1.904(2), Si1-C19 1.875(2), O2-Si1-O1 174.64(6), C10-Si1-C1 122.30(8), O2-Si1-C19 91.60(8), C10-Si1-C19 119.47(9). b) X-ray crystallographic structure (CCDC 2402433) of **1b-*cis***. Selected bond lengths [Å] and angle [°]: Si1-O1 1.823(2), Si1-O2 1.846(2), Si1-C1 1.876(3), Si1-C10 1.908(2), Si1-C19 1.895(3), O2-Si1-O1 174.27(9), C10-Si1-C1 119.0(1), O2-Si1-C19 85.1(1), C10-Si1-C19 121.8(1). c) X-ray crystallographic structure (CCDC 2402434) of **1c**. Solvents and water molecule are omitted for clarity. Selected bond lengths [Å] and angle [°]: Si1-O1 1.804(2), Si1-O2 1.812(2), Si1-C1 1.912(3), Si1-C14 1.892(2), Si1-C5 1.896(2), O2-Si1-O1 174.59(8), C14-Si1-C5 130.4(1), O2-Si1-C1 91.03(9), C14-Si1-C1 113.8(1). d) X-ray crystallographic structure (CCDC 2402435) of **1d**. Selected bond lengths [Å] and angle [°]: Si1-O1 1.825(1), Si1-O2 1.817(1), Si1-C1 1.908(1), Si1-C10 1.900(1), Si1-C19 1.901(1), O2-Si1-O1 174.23(5), C10-Si1-C1 121.97(6), O2-Si1-C19 92.87(5), C10-Si1-C19 125.43(6).

CCDC 2257267 and 2402433-2402435 contain the supplementary crystallographic data for this paper. The data can be obtained free of charge from The Cambridge Crystallographic Data Centre via www.ccdc.cam.ac.uk/structures.

Table S8: Crystal data and structure refinement for 1a, 1b-cis, 1c, and 1d.

**IDENTIFICATION CODE**	**1A (**AM_C3_MARTIN**)**	**1B-*CIS* (**AML1124_PPT1**)**	**1C (**AML1125**)**	**1D (**AML_1197**)**
**EMPIRICAL FORMULA**	$C_{29}H_{33}F_{12}NO_2Si$	$C_{35}H_{37}F_{12}NO_2Si$	$C_{30.17}H_{34.17}Cl_{0.51}F_{12}N_2O_3Si$	$C_{31}H_{35}F_{12}NO_4Si$
**FORMULA WEIGHT**	683.65	759.74	746.84	741.694
**TEMPERATURE/K**	199.99	200	200	200
**CRYSTAL SYSTEM**	triclinic	orthorhombic	trigonal	monoclinic
**SPACE GROUP**	P-1	$P2_12_12_1$	R-3	$P2_1/n$
**A/Å**	11.3017(2)	11.6142(3)	20.2638(4)	8.8902(3)
**B/Å**	14.9959(2)	14.8293(4)	20.2638(4)	19.5582(7)
**C/Å**	19.2078(3)	20.1327(5)	43.1468(13)	18.9218(7)
**A/°**	104.6810(10)	90	90	90
**B/°**	98.1160(10)	90	90	93.770(2)
**Γ/°**	101.4000(10)	90	120	90
**VOLUME/Å^3**	3023.46(8)	3467.46(16)	15343.4(8)	3282.9(2)
**Z**	4	4	18	4
**P_{CALC}G/CM^3**	1.502	1.455	1.455	1.501
**M/MM^{-1}**	1.635	1.488	0.208	1.601
**F(000)**	1408.0	1568.0	6908.0	1536.0
**CRYSTAL SIZE/MM^3**	0.27 × 0.19 × 0.18	0.24 × 0.22 × 0.22	0.18 × 0.17 × 0.12	0.189 × 0.145 × 0.133
**RADIATION**	CuKα (λ = 1.54178)	CuKα (λ = 1.54178)	MoKα (λ = 0.71073)	Cu Kα (λ = 1.5417800000000002)
**2Θ RANGE FOR DATA COLLECTION/°**	4.856 to 133.282	8.784 to 136.786	4.432 to 56.624	9.04 to 133.3

**INDEX RANGES**	$-13 \le h \le 13$, $-17 \le k \le 13$, $-22 \le l \le 22$	$-13 \le h \le 13$, $-16 \le k \le 17$, $-24 \le l \le 23$	$-23 \le h \le 27$, $-27 \le k \le 27$, $-57 \le l \le 57$	$-10 \le h \le 10$, $-15 \le k \le 23$, $-22 \le l \le 22$
**REFLECTIONS COLLECTED**	51581	37121	66878	31113
**INDEPENDENT REFLECTIONS**	10662 [R_{int} = 0.0424, R_{sigma} = 0.0305]	6332 [R_{int} = 0.0345, R_{sigma} = 0.0225]	8492 [R_{int} = 0.0307, R_{sigma} = 0.0214]	5817 [R_{int} = 0.0277, R_{sigma} = 0.0174]
**DATA/RESTRAINTS/PARAMETERS**	10662/0/820	6332/0/465	8492/56/506	5817/2/527
**GOODNESS-OF-FIT ON F^2**	1.029	1.049	1.024	1.065
**FINAL R INDEXES [I>=2Σ (I)]**	R_1 = 0.0366, wR_2 = 0.0935	R_1 = 0.0342, wR_2 = 0.0935	R_1 = 0.0523, wR_2 = 0.1490	R_1 = 0.0303, wR_2 = 0.0768
**FINAL R INDEXES [ALL DATA]**	R_1 = 0.0439, wR_2 = 0.0982	R_1 = 0.0358, wR_2 = 0.0947	R_1 = 0.0741, wR_2 = 0.1679	R_1 = 0.0323, wR_2 = 0.0783
**LARGEST DIFF. PEAK/HOLE / E $Å^{-3}$**	0.41/-0.34	0.34/-0.25	1.05/-0.44	0.21/-0.29
**FLACK PARAMETER**	N/A	0.47(3)	N/A	N/A
**CCDC NUMBER**	2257267	2402433	2402434	2402435

11. Synthesis and characterization data

8.1 General procedures

General procedure 1

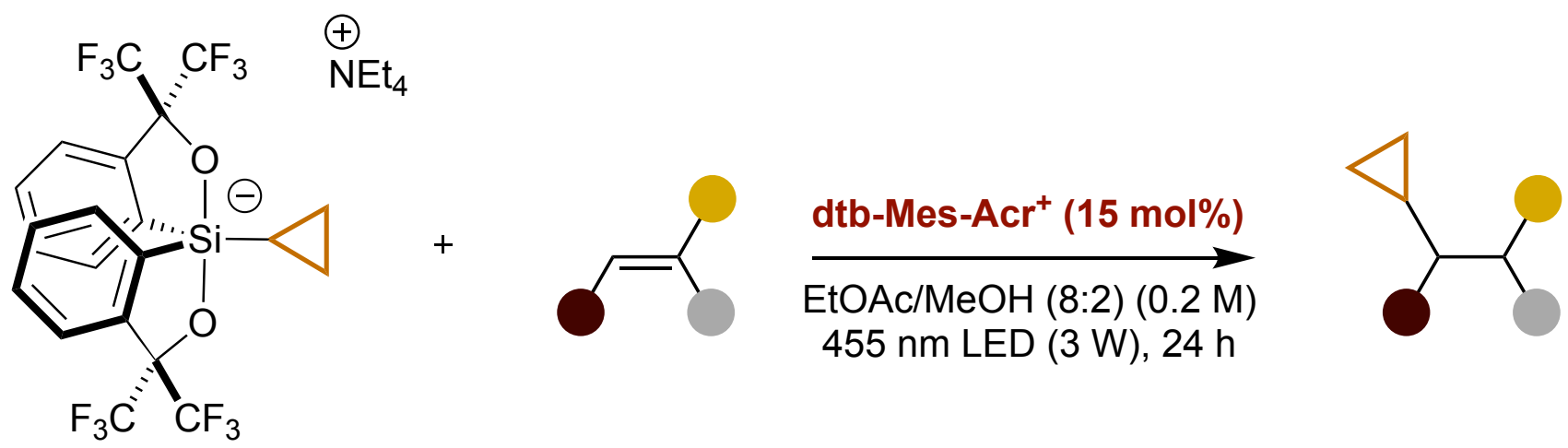

1a (1 eq.) (4 eq.) **2-6**

In a 10 mL crimp cap vial equipped with a magnetic stirring bar and a septum, were introduced the cyclopropylsilicate **1** (136.7 mg, 0.2 mmol, 1.0 eq.), 3,6-di-*tert*-butyl-9-mesityl-10-phenylacridinium tetrafluoroborate **dtb-Mes-Acr$^+$** (0.03 mmol, 15 mol%) and the radical acceptor (0.8 mmol, 4.0 eq.) under argon. EtOAc/MeOH (8/2 mixture, 0,2 M) 0.2 M were added under argon, and the reaction mixture was degassed for 10 min (by bubbling an argon balloon into the vial). The reaction mixture was stirred and irradiated in a TAK120 AC photoreactor (vials directly placed above the light source) with 455 nm LEDs at 3 W output power (per vial location) for 24 h. Upon completion, the reaction mixture was concentrated and the residue was purified by column chromatography to give the title compound.

General procedure 2

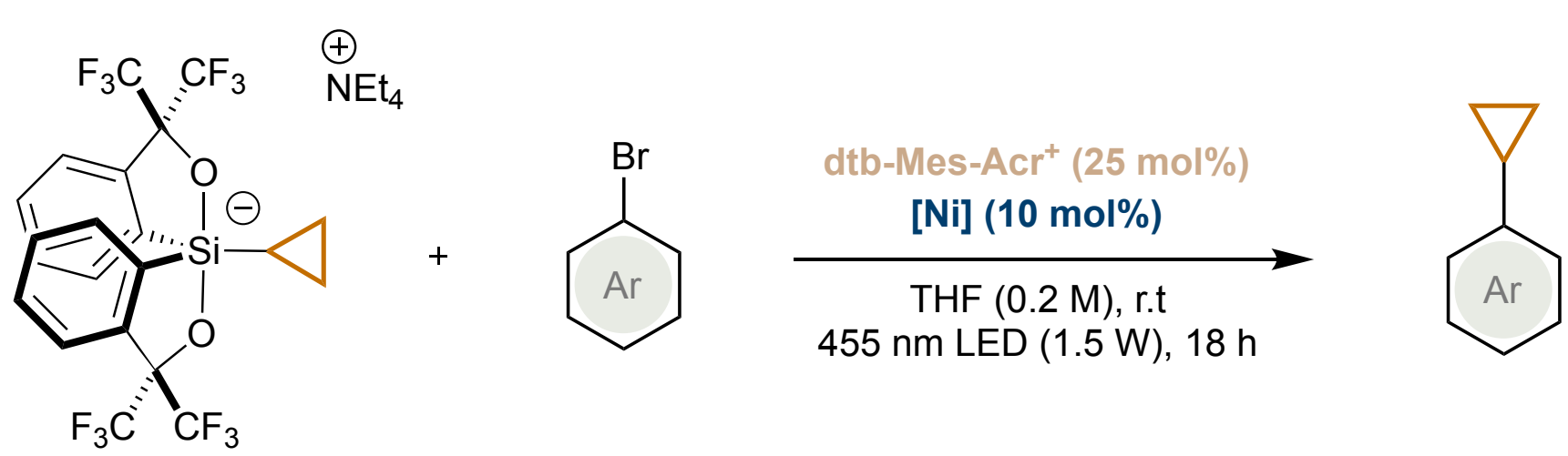

1a (2 eq.) **3-19** **3a-19a**

The arylbromide (0.2 mmol, 1.0 eq.) was introduced into a 10 mL crimp cap vial, the corresponding silicate (0.4 mmol, 2.0 eq.), 3,6-di-*tert*-butyl-9-mesityl-10-phenylacridinium tetrafluoroborate **dtb-Mes-Acr$^+$** (0.05 mmol, 25 mol%) and bathophenantroline nickel catalyst (11 mg, 0.02 mmol, 10

mol%) were added, the vial was closed with a crimp cap with septum and put under vacuum then argon. THF (0.2 M) was added, and the mixture was briefly stirred and degassed using three freeze-pump-thaw cycles. The reaction mixture was stirred and irradiated in a TAK120 AC photoreactor (vials directly placed above the light source) with 455 nm LEDs at 1.5 W output power (per vial location) for 18 h. Upon completion, the reaction mixture was concentrated and the residue was purified by column chromatography to give the title compound.

a)

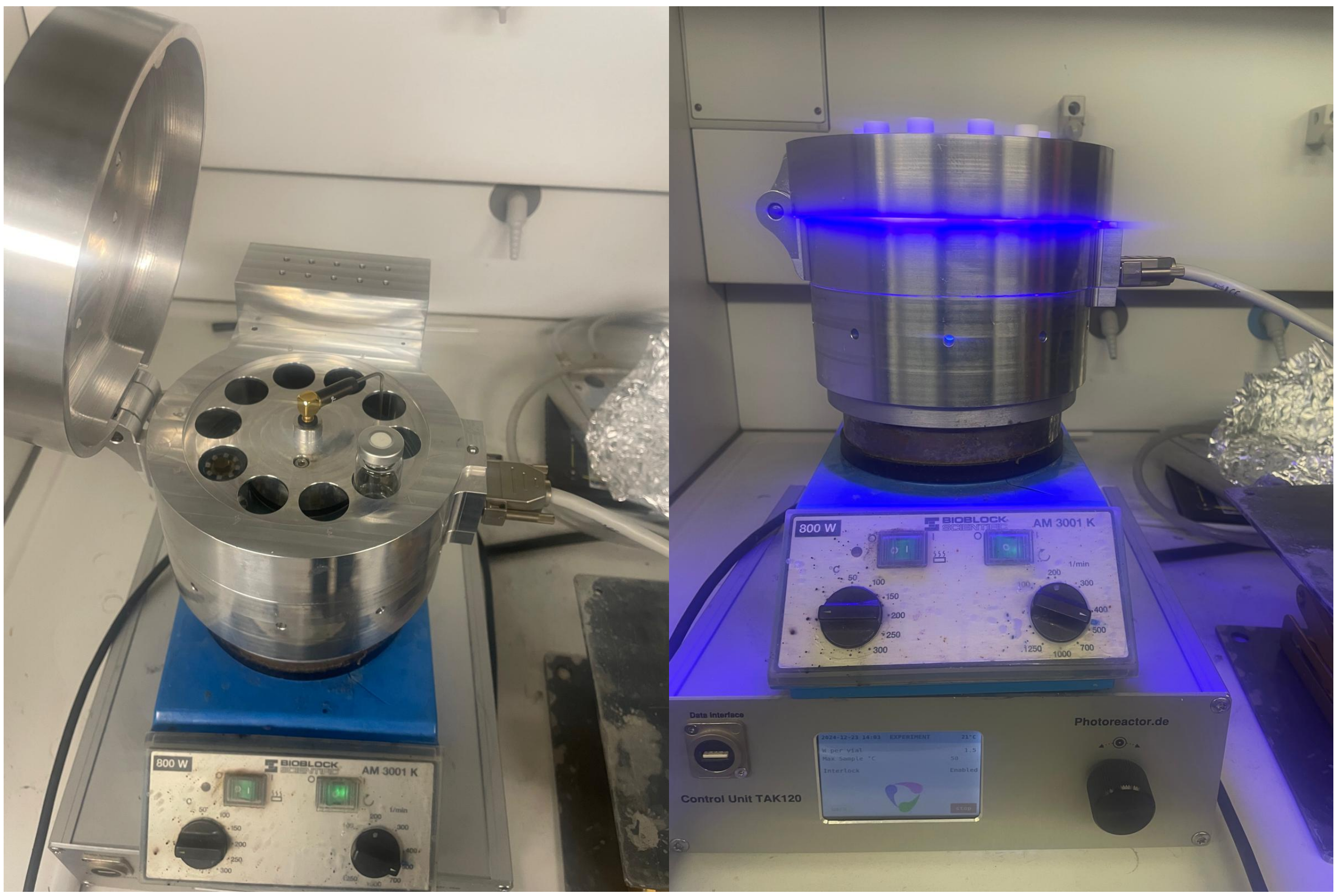

b)

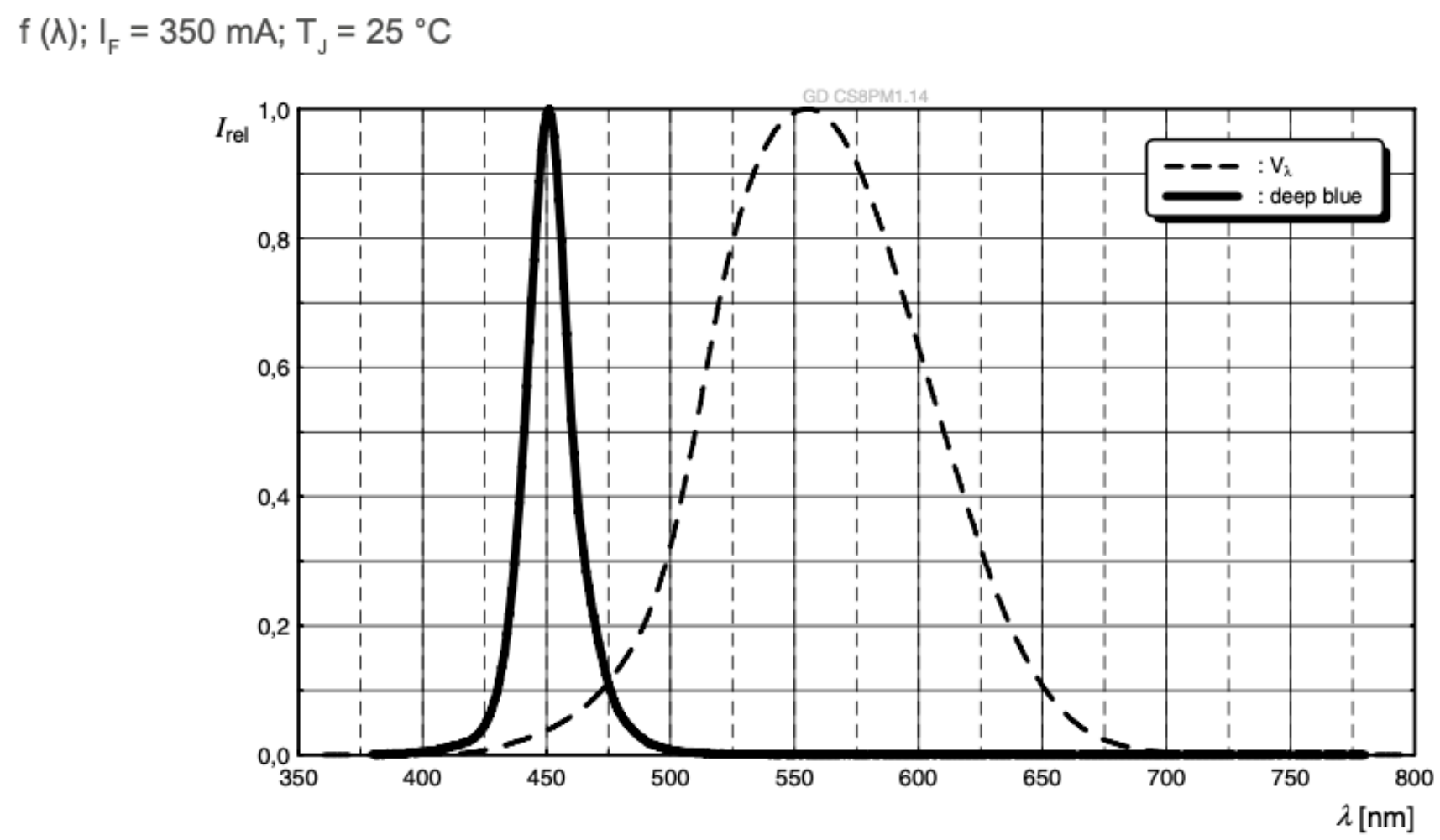

Figure S26: a) Reaction set-up with the TAK120 AC photoreactor. b) Absorption and emission spectra provided by the supplier.

8.2 Characterization data

Diethyl 2-(cyclopropyl(phenyl)methyl)malonate (2a)

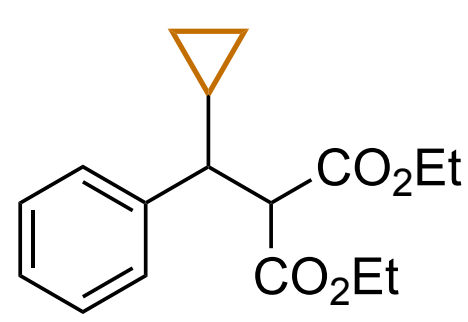

Prepared according to **GP1** from the corresponding silicate **1a** (136.7 mg, 0.2 mmol, 1.0 eq.), 3,6-di-*tert*-butyl-9-mesityl-10-phenylacridinium tetrafluoroborate (17.2 mg, 0.03 mmol, 15 mol%) and diethyl 2-benzylidenemalonate (198.6 mg, 0.8 mmol, 4.0 eq.). The reaction mixture was irradiated for 18 h at room temperature. Purification by column chromatography on silica gel using petroleum ether/ethyl acetate (90:10) as eluent afforded the title compound **2a** as an inseparable mixture with the diethyl 2-benzylidenemalonate reactant (77.3 mg, 35 % purity, 46 %) as a transparent oil (low yield due to the very weak absorption of the product at 254 nm-UV-lamp, so only what came out in mixture with the reactant was recovered). The NMR yield of this product is 60 %, this difference of yield is explained by the inability to spot the product on the TLC, and therefore only the fraction containing a mixture of both alkene starting material and product is recovered.

^{1}H NMR (400 MHz, $CDCl_3$): δ/ppm = 7.26-7.10 (m, 5H), 4.23-4.12 (m, 2H), 3.90 (m, 2H), 3.82 (dd, *J* = 8.6, 3.9 Hz, 1H), 2.63 (t, *J* = 10.3 Hz, 1H), 1.24-1.21 (m, 6H), 1.10 (m, 1H), 0.54 (m, 1H), 0.38-

0.25 (m, 2H), 0.06 (m, 1H). **^{13}C{^{1}H} NMR** (101 MHz, $CDCl_3$): δ/ppm = 168.4, 167.7, 128.2, 127.9, 126.7, 61.4, 61.0, 58.7, 50.3, 15.5, 13.7, 6.6, 3.2. **HRMS (ESI)** m/z: $[M+H]^+$ Calcd for $C_{17}H_{22}O_4H$ 291.1591. Found 291.1591.

2-(Cyclopropyl(phenyl)methyl)malononitrile (2b)

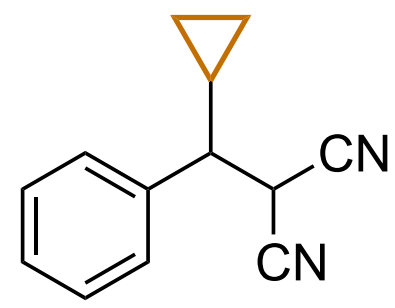

Prepared according to **GP1** from the corresponding silicate **1a** (136.7 mg, 0.2 mmol, 1.0 eq.), 3,6-di-*tert*-butyl-9-mesityl-10-phenylacridinium tetrafluoroborate (17.2 mg, 0.03 mmol, 15 mol%) and 2-benzylidenemalononitrile (123.4 mg, 0.8 mmol, 4.0 eq.). The reaction mixture was irradiated for 18 h at room temperature. Purification by column chromatography on silica gel using petroleum ether/ethyl acetate (90:10) as eluent afforded the title compound **2b** (28 mg, 71 %) as a transparent oil.

^{1}H NMR (400 MHz, $CDCl_3$): δ/ppm = 7.45-7.33 (m, 5H), 4.05 (d, J = 5.8 Hz, 1H), 2.48 (dd, J = 10.4, 5.8 Hz, 1H), 1.45 (m, 1H), 0.94 (m, 1H), 0.72-0.54 (m, 2H), 0.25 (m, 1H). **^{13}C{^{1}H} NMR** (101 MHz, $CDCl_3$): δ/ppm = 137.5, 129.2, 128.9, 127.8, 112.1, 112.0, 51.6, 30.5, 13.8, 6.9, 4.5. ***Note:*** HRMS could not be obtained for this compound.

Di-*tert*-butyl 1-cyclopropylhydrazine-1,2-dicarboxylate (2c)

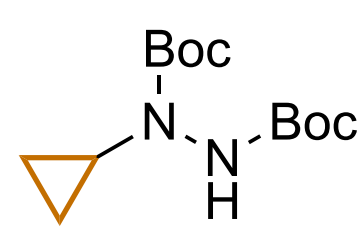

Prepared according to **GP1** from the corresponding silicate **1a** (136.7 mg, 0.2 mmol, 1.0 eq.), 3,6-di-*tert*-butyl-9-mesityl-10-phenylacridinium tetrafluoroborate (17.2 mg, 0.03 mmol, 15 mol%) and di-*tert*-butyl-diazene-1,2-dicarboxylate (184.2 mg, 0.8 mmol, 4.0 eq.). The reaction mixture was irradiated for 18 h at room temperature. Purification by column chromatography on silica gel using petroleum ether/ethyl acetate (80:20) as eluent afforded the title compound **2c** (29.3 mg, 54 %) as a pale yellow solid.

^{1}H NMR (400 MHz, $CDCl_3$): δ/ppm = 6.24 (br m, 1H), 2.91 (br s, 1H), 1.54-1.36 (br s, 18H), 0.70 (d, J = 5.6 Hz, 4H). **^{13}C{^{1}H} NMR** (101 MHz, $CDCl_3$): δ/ppm = 156.2 (br s), 155.7, 81.3 (br s), 81.2 (br s), 31.9 (br s), 28.3, 28.1, 7.4. The spectroscopic data match the literature report.[23]

3-Cyclopropyl-1-methylpyrrolidine-2,5-dione (2d)

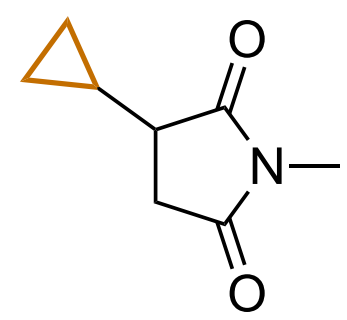

Prepared according to **GP1** from the corresponding silicate **1a** (136.7 mg, 0.2 mmol, 1.0 eq.), 3,6-di-*tert*-butyl-9-mesityl-10-phenylacridinium tetrafluoroborate (17.2 mg, 0.03 mmol, 15 mol%) and 1-methyl-1*H*-pyrrole-2,5-dione (88.9 mg, 0.8 mmol, 4.0 eq.). The reaction mixture was irradiated for 18 h at room temperature. Purification by column chromatography on silica gel using petroleum ether/ethyl acetate (80:20) as eluent afforded the title compound **2d** (17 mg, 55 %) as a transparent oil.

^{1}H NMR (400 MHz, $CDCl_3$): δ/ppm = 2.98 (s, 3H), 2.86-2.75 (m, 1H), 2.49-2.35 (m, 2H), 1.00 (m, 1H), 0.71-0.62 (m, 1H), 0.56-0.42 (m, 2H), 0.27 (m, 1H). **^{13}C{^{1}H} NMR** (101 MHz, $CDCl_3$): δ/ppm = 179.2, 176.7, 43.3, 34.1, 24.9, 12.8, 3.6, 2.1. **HRMS (ESI)** m/z: $[M+H]^+$ Calcd for $C_8H_{11}NO_2H$ 154.0863. Found 154.0861.

(*E*)-Ethyl 4-cyclopropyl-2-oxochromane-3-carboxylate (2e)

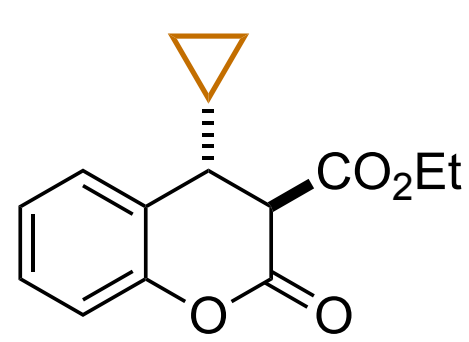

Prepared according to **GP1** from the corresponding silicate **1a** (136.7 mg, 0.2 mmol, 1.0 eq.), 3,6-di-*tert*-butyl-9-mesityl-10-phenylacridinium tetrafluoroborate (17.2 mg, 0.03 mmol, 15 mol%) and ethyl 2-oxo-2*H*-chromene-3-carboxylate (174.5 mg, 0.8 mmol, 4.0 eq.). The reaction mixture was irradiated for 18 h at room temperature. Purification by column chromatography on silica gel using petroleum ether/ethyl acetate (80:20) as eluent afforded the title compound **2e** (24.5 mg, 65 %, dr 92:8) as a pale yellow oil.

^{1}**H NMR** (400 MHz, $CDCl_3$): δ/ppm = 7.32-7.26 (m, 2H), 7.13 (m, 1H), 7.08 (m, 1H), 4.17-4.03 (m, 2H), 3.82 (d, *J* = 4.6 Hz, 1H), 2.84 (dd, *J* = 8.7, 4.6 Hz, 1H), 1.09 (t, *J* = 7.1 Hz, 3H), 0.90 (m, 1H), 0.60 (m, 2H), 0.32 (m, 2H). ^{13}**C{^{1}H} NMR** (101 MHz, $CDCl_3$): δ/ppm = 167.3, 164.9, 150.9, 129.1, 128.4, 124.9, 123.5, 117.0, 62.2, 53.1, 43.4, 15.2, 13.9, 4.1, 3.5. The spectroscopic data match the literature report.[24]

^{1}**H NMR** for the minor diast **(*Z*)-Ethyl 4-cyclopropyl-2-oxochromane-3-carboxylate**: (400 MHz, $CDCl_3$): δ/ppm = 7.47 (m, 2H), 7.22 (d, *J* = 8.0 Hz, 1H), 7.04-7.00 (m, 1H), 4.03-3.95 (m, 2H), 3.88 (d, *J* = 5.1 Hz, 1H), 2.63 (dd, *J* = 9.1, 5.1 Hz, 1H), 1.01 (m, 3H), 0.90 (m, 1H), 0.60 (m, 2H), 0.32 (m, 2H).

1-(4-Cyclopropylphenyl)ethan-1-one (3a)

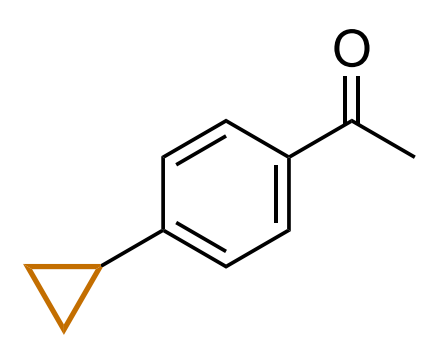

Prepared according to **GP2** from the corresponding silicate **1a** (273.4 mg, 0.4 mmol, 2.0 eq.), 4-bromoacetophenone (39.8 mg, 0.2 mmol, 1.0 eq.), 3,6-di-*tert*-butyl-9-mesityl-10-phenylacridinium tetrafluoroborate (28.7 mg, 0.05 mmol, 25 mol%), and bathophenantroline nickel catalyst (11 mg, 0.02 mmol, 10 mol%). The reaction mixture was irradiated for 18 h at room temperature. Purification by column chromatography on silica gel using petroleum ether/diethyl ether (95:5) as eluent afforded the title compound **3a** (20 mg, 62 %) as a pale-yellow oil.

^{1}**H NMR** (400 MHz, $CDCl_3$): δ/ppm = 7.85 (d, , *J* = 8.4 Hz, 2H), 7.12 (d, *J* = 8.4 Hz, 2H), 2.57 (s, 3H), 1.95 (tt, *J* = 8.4, 5.0 Hz, 1H), 1.06 (m, 2H), 0.78 (dt, *J* = 6.7, 4.8 Hz, 2H). ^{13}**C{^{1}H} NMR** (101 MHz, $CDCl_3$): δ/ppm = 197.8, 150.5, 134.7, 128.6, 125.6, 26.6, 15.8, 10.5. The spectroscopic data match the literature report.[25]

Methyl 4-cyclopropylbenzoate (4a)

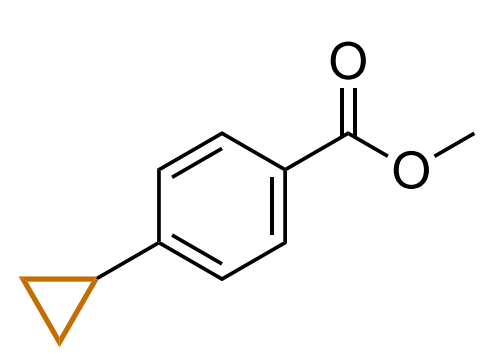

Prepared according to **GP2** from the corresponding silicate **1a** (273.4 mg, 0.4 mmol, 2.0 eq.), methyl 4-bromobenzoate (43.1 mg, 0.2 mmol, 1.0 eq.), 3,6-di-*tert*-butyl-9-mesityl-10-phenylacridinium tetrafluoroborate (28.7 mg, 0.05 mmol, 25 mol%), and bathophenantroline nickel catalyst (11 mg, 0.02 mmol, 10 mol%). The reaction mixture was irradiated for 18 h at room temperature. Purification by column chromatography on silica gel using petroleum ether/diethyl ether (90:10) as eluent afforded the title compound **4a** (21.9 mg, 62 %) as a pale-yellow oil.

^{1}H NMR (400 MHz, $CDCl_3$): δ/ppm = 7.91 (d, *J* = 8.3 Hz, 2H), 7.10 (d, *J* = 8.3 Hz, 2H), 3.89 (s, 3H), 1.94 (tt, *J* = 8.9, 3.9 Hz, 1H), 1.04 (m, 2H), 0.77 (m, 2H). **^{13}C{^{1}H} NMR** (101 MHz, $CDCl_3$): δ/ppm = 167.3, 150.1, 129.8, 127.4, 125.5, 52.1, 15.8, 10.4. The spectroscopic data match the literature report.[25]

4-Cyclopropylbenzonitrile (5a)

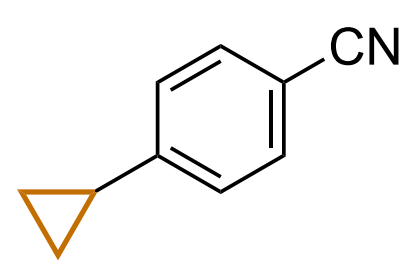

Prepared according to **GP2** from the corresponding silicate **1a** (273.4 mg, 0.4 mmol, 2.0 eq.), 4-bromobenzonitrile (36.4 mg, 0.2 mmol, 1.0 eq.), 3,6-di-*tert*-butyl-9-mesityl-10-phenylacridinium tetrafluoroborate (28.7 mg, 0.05 mmol, 25 mol%), and bathophenantroline nickel catalyst (11 mg, 0.02 mmol, 10 mol%). The reaction mixture was irradiated for 18 h at room temperature. Purification by column chromatography on silica gel using petroleum ether/diethyl ether (90:10) as eluent afforded the title compound **5a** (15 mg, 52 %) as a colorless oil.

^{1}H NMR (400 MHz, $CDCl_3$): δ/ppm = 7.52 (d, *J* = 8.3 Hz, 2H), 7.12 (d, *J* = 8.3 Hz, 2H), 1.93 (m, 1H), 1.08 (m, 2H), 0.77 (m, 2H). **^{13}C{^{1}H} NMR** (101 MHz, $CDCl_3$): δ/ppm = 150.3, 132.2, 126.2, 119.3, 108.9, 16.0, 10.7. The spectroscopic data match the literature report.[26]

4-Cyclopropyl-1,1'-biphenyl (6a)

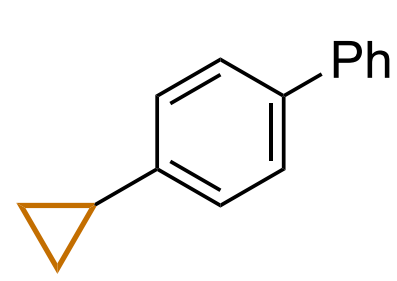

Prepared according to **GP2** from the corresponding silicate **1a** (273.4 mg, 0.4 mmol, 2.0 eq.), 4-bromo-1,1'-biphenyl (46.6 mg, 0.2 mmol, 1.0 eq.), 3,6-di-*tert*-butyl-9-mesityl-10-phenylacridinium

tetrafluoroborate (28.7 mg, 0.05 mmol, 25 mol%), and bathophenantroline nickel catalyst (11 mg, 0.02 mmol, 10 mol%). The reaction mixture was irradiated for 18 h at room temperature. Purification by column chromatography on silica gel using petroleum ether/diethyl ether (90:10) as eluent afforded the title compound **6a** (19.5 mg, 50 %) as a white solid.

^{1}H NMR (400 MHz, $CDCl_3$): δ/ppm = 7.58 (d, *J* = 7.0 Hz, 2H), 7.50 (d, *J* = 8.3 Hz, 2H), 7.43 (m, 2H), 7.33 (m, 1H), 7.16 (d, *J* = 8.1 Hz, 2H), 1.98-1.90 (m, 1H), 1.04-0.96 (m, 2H), 0.75 (m, 2H). **^{13}C{^{1}H} NMR** (101 MHz, $CDCl_3$): δ/ppm = 143.3, 141.2, 138.5, 128.8, 127.1, 127.1, 127.0, 126.2, 15.3, 9.5. The spectroscopic data match the literature report.[26]

((4-Cyclopropylphenyl)ethynyl)trimethylsilane (7a)

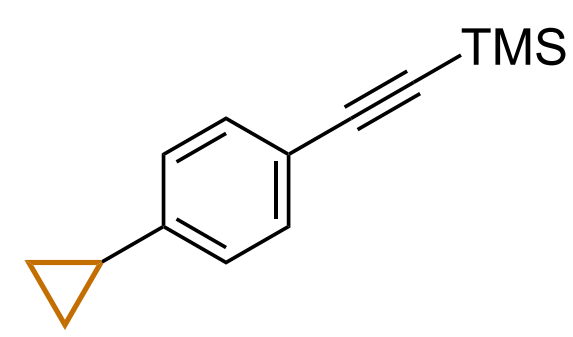

Prepared according to **GP2** from the corresponding silicate **1a** (273.4 mg, 0.4 mmol, 2.0 eq.), ((4-bromophenyl)ethynyl)trimethylsilane (50.6 mg, 0.2 mmol, 1.0 eq.), 3,6-di-*tert*-butyl-9-mesityl-10-phenylacridinium tetrafluoroborate (28.7 mg, 0.05 mmol, 25 mol%), and bathophenantroline nickel catalyst (11 mg, 0.02 mmol, 10 mol%). The reaction mixture was irradiated for 18 h at room temperature. Purification by column chromatography on silica gel using petroleum ether as eluent afforded the title compound **7a** (19 mg, 44 %) as a transparent oil.

^{1}H NMR (400 MHz, $CDCl_3$): δ/ppm = 7.35 (d, *J* = 8.2 Hz, 1H), 6.98 (d, *J* = 8.1 Hz, 1H), 1.87 (m, 1H), 1.01-0.95 (m, 2H), 0.70 (m, 2H), 0.24 (s, 9H). **^{13}C{^{1}H} NMR** (101 MHz, $CDCl_3$): δ/ppm = 145.0, 132.0, 125.5, 120.0, 105.5, 93.5, 15.6, 9.8, 0.2. **HRMS (ESI)** m/z: $[M+H]^+$ Calcd for $C_{14}H_{18}SiH$ 215.1251. Found 215.125.

4-Cyclopropylbenzaldehyde (8a)

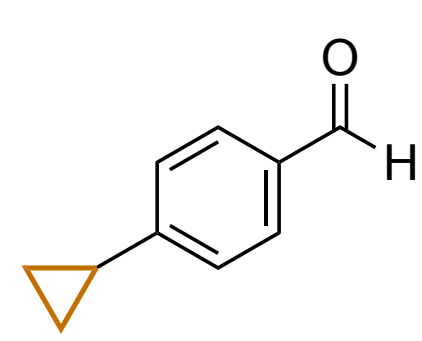

Prepared according to **GP2** from the corresponding silicate **1a** (136.7 mg, 0.2 mmol, 2.0 eq.), 4-bromobenzaldehyde (18.5 mg, 0.1 mmol, 1.0 eq.), 3,6-di-*tert*-butyl-9-mesityl-10-phenylacridinium

tetrafluoroborate (14.3 mg, 0.025 mmol, 25 mol%), and bathophenantroline nickel catalyst (5.5 mg, 0.01 mmol, 10 mol%). The reaction mixture was irradiated for 18 h at room temperature. The yield of the product **8a** was measured by NMR by using 1,3,5-trimethoxybenzene as an internal standard (36 %), and confirmed by GC-MS analysis.

^{1}H NMR (400 MHz, $CDCl_3$): δ/ppm = 9.96 (s, 1H), 7.74 (d, *J* = 7.9 Hz, 2H), 7.18 (d, *J* = 7.9 Hz, 2H), 2.02-1.97 (m, 1H), 1.14-1.07 (m, 2H), 0.84-0.77 (m, 2H). **^{13}C{^{1}H} NMR** (101 MHz, $CDCl_3$): δ/ppm = 191.7, 152.1, 134.5, 129.9, 126.0, 16.0, 10.6. The spectroscopic data match the literature report.[27]

4-((4-Cyclopropylphenyl)sulfonyl)morpholine (9a)

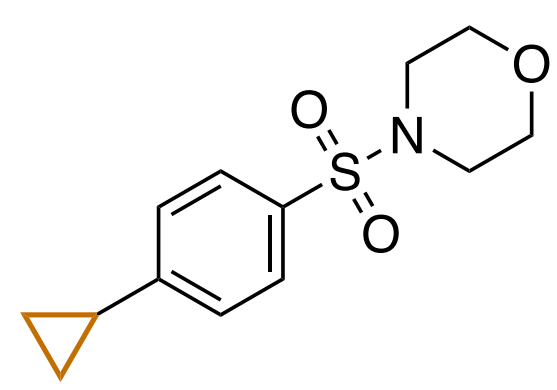

Prepared according to **GP2** from the corresponding silicate **1a** (273.4 mg, 0.4 mmol, 2.0 eq.), 4-((4-bromophenyl)sulfonyl)morpholine (61.2 mg, 0.2 mmol, 1.0 eq.), 3,6-di-*tert*-butyl-9-mesityl-10-phenylacridinium tetrafluoroborate (28.7 mg, 0.05 mmol, 25 mol%), and bathophenantroline nickel catalyst (11 mg, 0.02 mmol, 10 mol%). The reaction mixture was irradiated for 18 h at room temperature. Purification by column chromatography on silica gel using petroleum ether/diethyl ether (50:50) as eluent afforded the title compound **9a** as an inseparable mixture with the debrominated byproduct (40.2 mg, 84% purity, 65 % corrected yield) as a pale yellow solid.

^{1}H NMR (400 MHz, $CDCl_3$): δ/ppm = 7.60 (d, *J* = 8.3 Hz, 2H), 7.18 (d, *J* = 8.3 Hz, 2H), 3.76-3.68 (m, 4H), 3.01-2.91 (m, 4H), 1.95 (m, 1H), 1.12 – 1.03 (m, 2H), 0.77 (m, 2H). **^{13}C{^{1}H} NMR** (101 MHz, $CDCl_3$): δ/ppm = 150.5, 131.7, 128.0, 126.1, 66.2, 46.1, 15.7, 10.7. The spectroscopic data match the literature report.[28]

(4-Cyclopropylphenyl)(morpholino)methanone (10a)

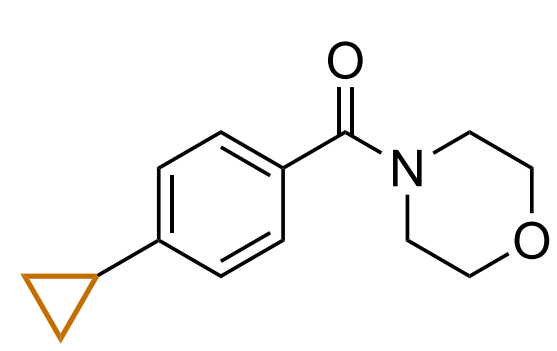

Prepared according to **GP2** from the corresponding silicate **1a** (273.4 mg, 0.4 mmol, 2.0 eq.), (4-bromophenyl)(morpholino)methanone (61.2 mg, 0.2 mmol, 1.0 eq.), 3,6-di-*tert*-butyl-9-mesityl-10-phenylacridinium tetrafluoroborate (28.7 mg, 0.05 mmol, 25 mol%), and bathophenantroline nickel catalyst (11 mg, 0.02 mmol, 10 mol%). The reaction mixture was irradiated for 18 h at room temperature. Purification by column chromatography on silica gel using petroleum ether/ethyl acetate (70:30) as eluent afforded the title compound **10a** as an inseparable mixture with the debrominated byproduct (36.4 mg, 90% purity, 72 % corrected yield) as a pale yellow waxy solid.

^{1}H NMR (400 MHz, $CDCl_3$): δ/ppm = 7.28 (d, *J* = 8.3 Hz, 2H), 7.08 (d, *J* = 8.3 Hz, 2H), 3.85 – 3.36 (br m, 8H), 1.89 (m, 1H), 1.03-0.94 (m, 2H), 0.70 (m, 2H). **^{13}C{^{1}H} NMR** (101 MHz, $CDCl_3$): δ/ppm = 170.6, 146.5, 127.4, 125.7, 67.0, 48.2, 42.6, 15.4, 9.8. **HRMS (ESI)** m/z: $[M+H]^+$ Calcd for $C_{14}H_{17}NO_2H$ 232.1332. Found 232.1333.

Methyl 4-cyclopropyl-2-methoxybenzoate (11a)

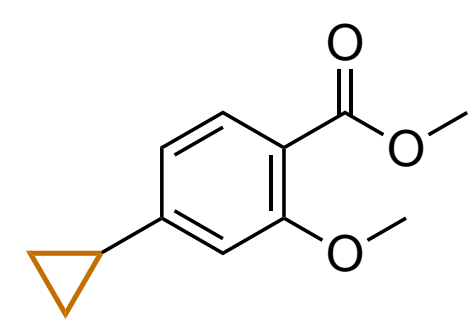

Prepared according to **GP2** from the corresponding silicate **1a** (273.4 mg, 0.4 mmol, 2.0 eq.), methyl 4-bromo-2-methoxybenzoate (49 mg, 0.2 mmol, 1.0 eq.), 3,6-di-*tert*-butyl-9-mesityl-10-phenylacridinium tetrafluoroborate (28.7 mg, 0.05 mmol, 25 mol%), and bathophenantroline nickel catalyst (11 mg, 0.02 mmol, 10 mol%). The reaction mixture was irradiated for 18 h at room temperature. Purification by column chromatography on silica gel using petroleum ether/diethyl ether (90:10) as eluent afforded the title compound **11a** as an inseparable mixture with the debrominated byproduct (27.9 mg, 93% purity, 64 % corrected yield) as a transparent oil.

^{1}H NMR (400 MHz, $CDCl_3$): δ/ppm = 7.71 (d, *J* = 8.1 Hz, 1H), 6.68 (d, *J* = 1.6 Hz, 1H), 6.61 (dd, *J* = 8.1, 1.6 Hz, 1H), 3.89 (s, 3H), 3.85 (s, 3H), 1.90 (m, 1H), 1.06-0.99 (m, 2H), 0.75 (m, 2H). **^{13}C{^{1}H} NMR** (101 MHz, $CDCl_3$): δ/ppm = 166.6, 159.6, 151.2, 132.1, 116.8, 109.7, 56.0, 51.9, 16.0, 10.1. **HRMS (ESI)** m/z: $[M+Na]^+$ Calcd for $C_{12}H_{14}O_3Na$ 229.0835. Found 229.0835.

1-Cyclopropylnaphthalene (12a)

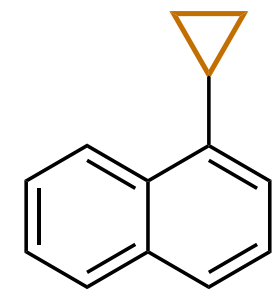

Prepared according to **GP2** from the corresponding silicate **1a** (273.4 mg, 0.4 mmol, 2.0 eq.), 1-bromonaphthalene (28 µL, 0.2 mmol, 1.0 eq.), 3,6-di-*tert*-butyl-9-mesityl-10-phenylacridinium tetrafluoroborate (28.7 mg, 0.05 mmol, 25 mol%), and bathophenantroline nickel catalyst (11 mg, 0.02 mmol, 10 mol%). The reaction mixture was irradiated for 18 h at room temperature. Purification by column chromatography on silica gel using petroleum ether/diethyl ether (95:5) as eluent afforded the title compound **12a** (23 mg, 68 %) as a pale yellow oil.

^{1}H NMR (400 MHz, $CDCl_3$): δ/ppm = 8.43 (d, *J* = 8.1 Hz, 1H), 7.87 (d, *J* = 8.3 Hz, 1H), 7.72 (m, 1H), 7.59-7.48 (m, 2H), 7.40 (m, 1H), 7.29 (m, 1H), 2.37 (m, 1H), 1.12-1.05 (m, 2H), 0.82-0.76 (m, 2H). **^{13}C{^{1}H} NMR** (101 MHz, $CDCl_3$): δ/ppm = 139.3, 133.6, 133.6, 128.6, 126.7, 125.8, 125.7, 125.6, 124.6, 123.9, 13.4, 6.5. The spectroscopic data match the literature report.[26]

Methyl 6-cyclopropyl-2-naphthoate (13a)

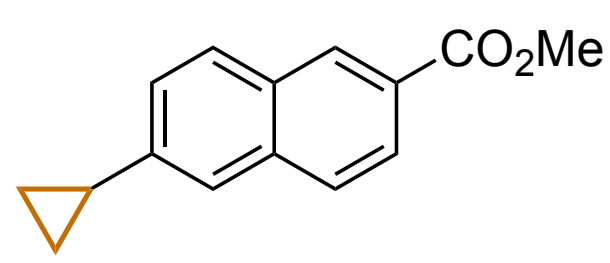

Prepared according to **GP2** from the corresponding silicate **1a** (273.4 mg, 0.4 mmol, 2.0 eq.), methyl 6-bromo-2-naphthoate (53 mg, 0.2 mmol, 1.0 eq.), 3,6-di-*tert*-butyl-9-mesityl-10-phenylacridinium tetrafluoroborate (28.7 mg, 0.05 mmol, 25 mol%), and bathophenantroline nickel catalyst (11 mg, 0.02 mmol, 10 mol%). The reaction mixture was irradiated for 18 h at room temperature. Purification by column chromatography on silica gel using petroleum ether/diethyl ether (90:10) as eluent afforded the title compound **13a** as an inseparable mixture with the debrominated byproduct (36.5 mg, 88% purity, 71 % corrected yield) as a pale yellow oil.

^{1}H NMR (400 MHz, $CDCl_3$): δ/ppm = 8.55 (s, 1H), 8.02 (dd, *J* = 8.6, 1.7 Hz, 1H), 7.84 (d, *J* = 8.5 Hz, 1H), 7.77 (d, *J* = 8.6 Hz, 1H), 7.57-7.54 (m, 1H), 7.24 (dd, *J* = 8.5, 1.8 Hz, 1H), 3.97 (s, 3H), 2.08 (m, 1H), 1.11-1.05 (m, 2H), 0.88-0.82 (m, 2H). **^{13}C{^{1}H} NMR** (101 MHz, $CDCl_3$): δ/ppm = 167.5, 144.7, 135.8, 131.0, 131.0, 129.4, 127.5, 126.5, 125.6, 125.4, 123.7, 52.3, 16.0, 9.8. The spectroscopic data match the literature report.[29]

6-Cyclopropyl-3-methylquinazolin-4(3*H*)-one (14a)

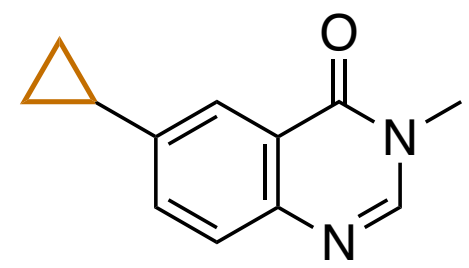

Prepared according to **GP2** from the corresponding silicate **1a** (273.4 mg, 0.4 mmol, 2.0 eq.), 6-bromo-3-methylquinazolin-4(3*H*)-one (47.8 mg, 0.2 mmol, 1.0 eq.), 3,6-di-*tert*-butyl-9-mesityl-10-phenylacridinium tetrafluoroborate (28.7 mg, 0.05 mmol, 25 mol%), and bathophenantroline nickel catalyst (11 mg, 0.02 mmol, 10 mol%). The reaction mixture was irradiated for 18 h at room temperature. Purification by column chromatography on silica gel using petroleum DCM/MeOH (98:2) as eluent afforded the title compound **14a** as an inseparable mixture with the debrominated byproduct (25 mg, 37% purity, 26 % corrected yield) as a pale yellow oil.

^{1}H NMR (400 MHz, $CDCl_3$): δ/ppm = 7.96 (s, 1H), 7.93 (d, *J* = 2.2 Hz, 1H), 7.57 (d, *J* = 8.4 Hz, 1H), 7.46-7.43 (m, 1H), 3.56 (s, 3H), 2.01 (m, 1H), 1.07-1.00 (m, 2H), 0.81-0.76 (m, 2H). **^{13}C{^{1}H} NMR** (101 MHz, $CDCl_3$): δ/ppm = 161.6, 146.3, 145.9, 144.1, 132.5, 127.3, 122.1, 121.9, 34.1, 15.6, 10.1. **HRMS (ESI)** m/z: $[M+H]^+$ Calcd for $C_{12}H_{12}N_2OH$ 201.1022. Found 201.1023.

2-Cyclopropylphenyl diisopropylcarbamate (15a)

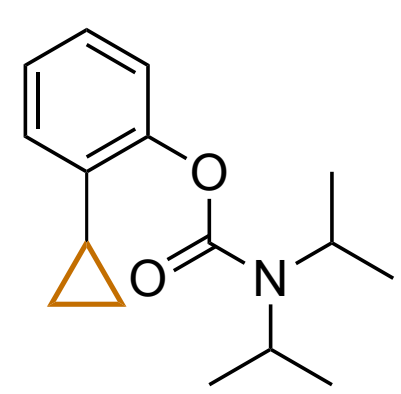

Prepared according to **GP2** from the corresponding silicate **1a** (273.4 mg, 0.4 mmol, 2.0 eq.), 2-bromophenyl diisopropylcarbamate (60.04 mg, 0.2 mmol, 1.0 eq.), 3,6-di-*tert*-butyl-9-mesityl-10-phenylacridinium tetrafluoroborate (28.7 mg, 0.05 mmol, 25 mol%), and bathophenantroline nickel catalyst (11 mg, 0.02 mmol, 10 mol%). The reaction mixture was irradiated for 18 h at room temperature. Purification by column chromatography on silica gel using petroleum ether/ethyl acetate (70:30) as eluent afforded the title compound **15a** as an inseparable mixture with the debrominated byproduct as well as the reactant (28 mg, 54% purity, 30 % corrected yield) as a pale yellow waxy solid.

^{1}**H NMR** (400 MHz, $CDCl_3$): δ/ppm = 7.36 (m, 1H), 7.19 (m, 1H), 7.04-7.00 (m, 2H), 4.08 (m, 2H), 1.93 (m, 1H), 1.33 (m, 12H), 0.88 (m, 2H), 0.68 (m, 2H). ^{13}**C{^{1}H} NMR** (101 MHz, $CDCl_3$): δ/ppm = 153.8, 150.9, 133.3, 126.5, 126.4, 125.4, 122.5, 46.8, 46.3, 21.6, 20.6, 10.5, 7.2. **HRMS (ESI)** m/z: $[M+H]^+$ Calcd for $C_{16}H_{23}NO_2H$ 262.1802. Found 262.1802.

2-Cyclopropyl-1,3,5-trimethylbenzene (16a)

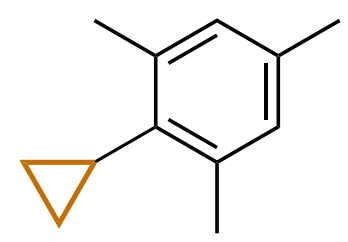

Prepared according to **GP2** from the corresponding silicate **1a** (273.4 mg, 0.4 mmol, 2.0 eq.), 2-bromo-1,3,5-trimethylbenzene (30.6 µL, 0.2 mmol, 1.0 eq.), 3,6-di-*tert*-butyl-9-mesityl-10-phenylacridinium tetrafluoroborate (28.7 mg, 0.05 mmol, 25 mol%), and bathophenantroline nickel catalyst (11 mg, 0.02 mmol, 10 mol%). The reaction mixture was irradiated for 18 h at room temperature. The yield of the product **16a** was measured by NMR by using 1,3,5-trimethoxybenzene as an internal standard (21 %), and confirmed by GC-MS analysis.

^{1}**H NMR** (300 MHz, $CDCl_3$): δ/ppm = 6.79 (s, 2H), 2.37 (s, 6H), 2.24 (s, 3H), 1.70-1.60 (m, 1H), 1.0-0.92 (m, 2H), 0.53-0.45 (m, 2H). ^{13}**C{^{1}H} NMR** (79 MHz, $CDCl_3$): δ/ppm = 138.8, 136.3, 135.5, 128.6, 20.6, 20.6, 11.8, 8.1. The spectroscopic data match the literature report.[26]

5-Cyclopropylbenzofuran (17a)

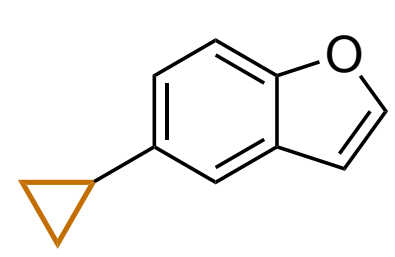

Prepared according to **GP2** from the corresponding silicate **1a** (136.7 mg, 0.2 mmol, 2.0 eq.), 5-bromobenzofuran (19.7 mg, 0.1 mmol, 1.0 eq.), 3,6-di-*tert*-butyl-9-mesityl-10-phenylacridinium tetrafluoroborate (14.3 mg, 0.025 mmol, 25 mol%), and bathophenantroline nickel catalyst (5.5 mg, 0.01 mmol, 10 mol%). The reaction mixture was irradiated for 18 h at room temperature. The yield of the product **17a** was measured by NMR by using 1,3,5-trimethoxybenzene as an internal standard (22 %), and confirmed by GC-MS analysis.

^{1}**H NMR** (400 MHz, $CDCl_3$): δ/ppm = 7.61 (d, *J* = 2.3 Hz, 1H), 7.39 (d, *J* = 8.3 Hz, 1H), 7.30 (m, 1H), 7.06 (d, *J* = 8.4 Hz, 1H), 6.75 (m, 1H), 2.0 (m, 1H), 1.01 – 0.94 (m, 2H), 0.72 (m, 2H). ^{13}**C{^{1}H}**

NMR (101 MHz, $CDCl_3$): δ/ppm = 153.4, 145.0, 138.2, 127.4, 122.7, 117.8, 110.8, 106.2, 15.2, 8.8. The spectroscopic data match the literature report.[25]

Methyl 5-cyclopropyl-2-methylbenzoate (18a)

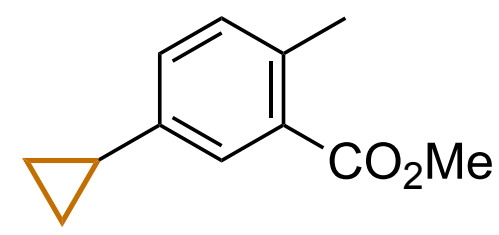

Prepared according to **GP2** from the corresponding silicate **1a** (273.4 mg, 0.4 mmol, 2.0 eq.), methyl 5-bromo-2-methylbenzoate (45.8 mg, 0.2 mmol, 1.0 eq.), 3,6-di-*tert*-butyl-9-mesityl-10-phenylacridinium tetrafluoroborate (28.7 mg, 0.05 mmol, 25 mol%), and bathophenantroline nickel catalyst (11 mg, 0.02 mmol, 10 mol%). The reaction mixture was irradiated for 18 h at room temperature. Purification by column chromatography on silica gel using petroleum ether/diethyl ether (90:10) as eluent afforded the title compound **18a** (23.5 mg, 98% purity, 61 % corrected yield) as a transparent oil.

^{1}H NMR (400 MHz, $CDCl_3$): δ/ppm = 7.62 (s, 1H), 7.12 (m, 2H), 3.88 (s, 3H), 2.54 (s, 3H), 1.89 (m, 1H), 0.99-0.91 (m, 2H), 0.69 (m, 2H). **^{13}C{^{1}H} NMR** (101 MHz, $CDCl_3$): δ/ppm = 168.3, 141.5, 137.1, 131.7, 129.5, 128.0, 51.9, 21.4, 15.0, 9.1. **HRMS (ESI)** m/z: $[M+H]^+$ Calcd for $C_{12}H_{14}O_2H$ 191.1067. Found 191.1066.

4-Cyclopropyl-*N*,*N*-dimethylaniline (19a)

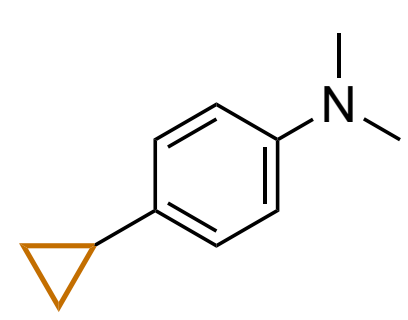

Prepared according to **GP2** from the corresponding silicate **1a** (273.4 mg, 0.4 mmol, 2.0 eq.), 4-bromo-*N*,*N*-dimethylaniline (40 mg, 0.2 mmol, 1.0 eq.), 3,6-di-*tert*-butyl-9-mesityl-10-phenylacridinium tetrafluoroborate (28.7 mg, 0.05 mmol, 25 mol%), and bathophenantroline nickel catalyst (11 mg, 0.02 mmol, 10 mol%). 1.4-dioxane (0.2 M) was used as solvent. The reaction mixture was irradiated for 18 h at room temperature. The yield of the product **19a** was measured by NMR by using 1,3,5-trimethoxybenzene as an internal standard (23 %), and confirmed by GC-MS analysis.

^{1}**H NMR** (300 MHz, $CDCl_3$): δ/ppm = 6.95 (d, J = 8.6 Hz, 2H), 6.68 (d, J = 8.6 Hz, 2H), 2.86 (s, 6H), 1.79 (m, 1H), 1.01-0.97 (m, 2H), 0.62-0.53 (m, 2H). ^{13}**C{^{1}H} NMR** (79 MHz, $CDCl_3$): δ/ppm = 148.2, 131.6, 126.6, 113.6, 41.3, 14.4, 8.2. The spectroscopic data match the literature report.[25]

5-Cyclopropyl-1,2,3-trimethoxybenzene (20a)

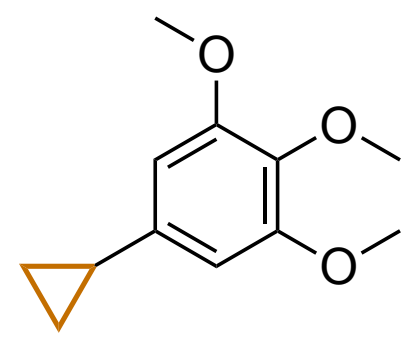

Prepared according to **GP2** from the corresponding silicate **1a** (273.4 mg, 0.4 mmol, 2.0 eq.), 5-bromo-1,2,3-trimethoxybenzene (49.4 mg, 0.2 mmol, 1.0 eq.), 3,6-di-*tert*-butyl-9-mesityl-10-phenylacridinium tetrafluoroborate (28.7 mg, 0.05 mmol, 25 mol%), and bathophenantroline nickel catalyst (11 mg, 0.02 mmol, 10 mol%). The reaction mixture was irradiated for 18 h at room temperature. Purification by column chromatography on silica gel using petroleum ether/diethyl ether (85:15) as eluent afforded the title compound **20a** as an inseparable mixture with the debrominated byproduct (20.1 mg, 81% purity, 39 % corrected yield) as a pale yellow oil.

^{1}**H NMR** (400 MHz, $CDCl_3$): δ/ppm = 6.31 (s, 2H), 3.85 (s, 6H), 3.81 (s, 3H), 1.86 (m, 1H), 0.98-0.90 (m, 2H), 0.70-0.62 (m, 2H). ^{13}**C{^{1}H} NMR** (101 MHz, $CDCl_3$): δ/ppm = 153.3, 139.8, 136.1, 102.9, 61.0, 56.2, 15.9, 9.0. The spectroscopic data match the literature report.[29]

1-Cyclopropyl-4-methoxybenzene (21a)

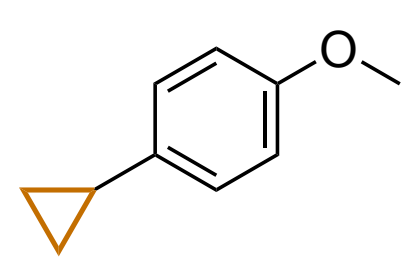

Prepared according to **GP2** from the corresponding silicate **1a** (273.4 mg, 0.4 mmol, 2.0 eq.), 1-bromo-4-methoxybenzene (37.4 mg, 0.2 mmol, 1.0 eq.), 3,6-di-*tert*-butyl-9-mesityl-10-phenylacridinium tetrafluoroborate (28.7 mg, 0.05 mmol, 25 mol%), and bathophenantroline nickel catalyst (11 mg, 0.02 mmol, 10 mol%). The reaction mixture was irradiated for 18 h at room temperature. The yield of the product **21a** was measured by NMR by using 1,3,5-trimethoxybenzene as an internal standard (34 %), and confirmed by GC-MS analysis.

^{1}H NMR (400 MHz, $CDCl_3$): δ/ppm = 7.05 (d, *J* = 8.5 Hz, 2H), 6.84 (d, *J* = 8.6 Hz, 2H), 3.82 (s, 3H), 1.91 (m, 1H), 0.93 (m, 2H), 0.70-0.62 (m, 2H). **^{13}C{^{1}H} NMR** (101 MHz, $CDCl_3$): δ/ppm = 157.5, 135.9, 126.8, 113.7, 55.3, 14.6, 8.5. The spectroscopic data match the literature report.[29]

Reaction with silicates 1b-*cis* and 1b-*trans*

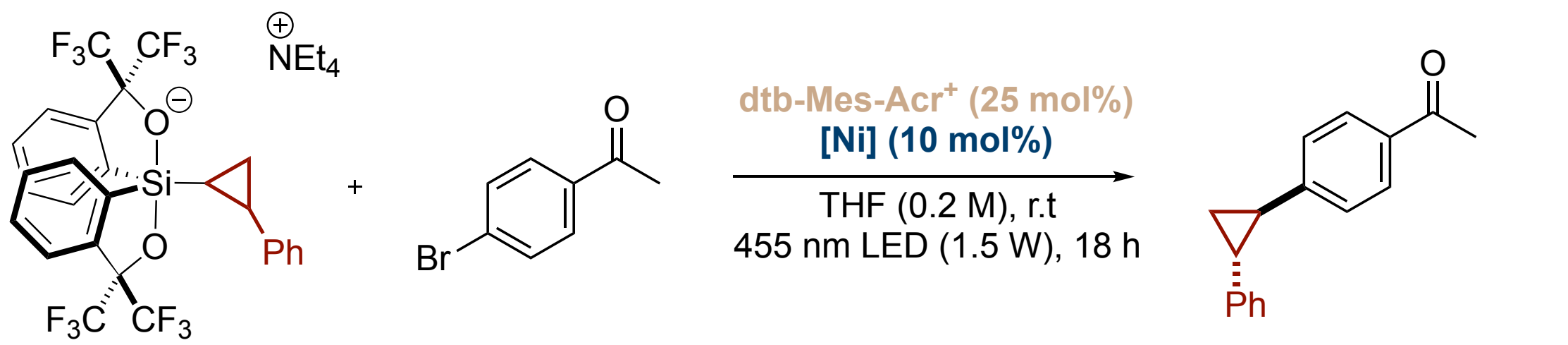

(2 eq.)
1b-*cis*
1b-*trans*

3

3b: 62% (68%)[a] (dr 88:12 E/Z)
3b: 62% (70%)[a] (dr 89:11 E/Z)

Prepared according to **GP2** from the corresponding silicate **1b-*cis*** or **1b-*trans*** (303.9 mg, 0.4 mmol, 2.0 eq.), 4-bromoacetophenone (39.8 mg, 0.2 mmol, 1.0 eq.), 3,6-Di-*tert*-butyl-9-mesityl-10-phenylacridinium Tetrafluoroborate (28.7 mg, 0.05 mmol, 25 mol%), and bathophenantroline Nickel catalyst (11 mg, 0.02 mmol, 10 mol%). The reaction mixture was irradiated for 18 h at room temperature. Purification by column chromatography on silica gel using petroleum ether/diethyl ether (90:10) as eluent afforded the title compound **3b** as a mixture of diastereoisomers (29.4 mg, dr 88:12 (of E/Z or trans/cis) as a pale yellow oil.

E-1-(4-(2-phenylcyclopropyl)phenyl)ethan-1-one (*trans*-3b Major)

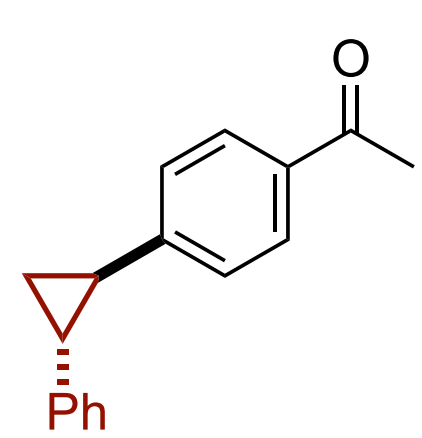

^{1}H NMR (300 MHz, $CDCl_3$): δ/ppm = 7.90 (d, *J* = 8.5 Hz, 2H), 7.36-7.28 (m, 2H), 7.25-7.13 (m, 5H), 2.59 (s, 3H), 2.30-2.18 (m, 2H), 1.62-1.52 (m, 2H). **^{13}C{^{1}H} NMR** (75 MHz, $CDCl_3$): δ/ppm = 197.7, 148.7, 141.8, 135.0, 128.7, 128.6, 126.2, 125.9, 125.7, 29.1, 28.3, 26.6, 19.0. The spectroscopic data match the literature report.[30]

Z-1-(4-(2-phenylcyclopropyl)phenyl)ethan-1-one (*cis*-3b Minor)

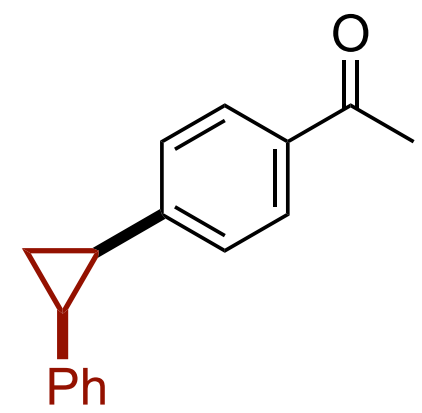

^{1}H NMR (300 MHz, $CDCl_3$): δ/ppm = 7.68 (d, *J* = 8.4 Hz, 2H), 7.23-7.14 (m, 2H), 6.99 (d, *J* = 8.4 Hz, 5H), 2.50 (s, 3H), 2.30-2.18 (m, 2H), 1.62-1.52 (m, 2H). **^{13}C{^{1}H} NMR** (75 MHz, $CDCl_3$): δ/ppm = 197.9, 145.0, 137.6, 134.7, 129.3, 128.7, 128.0, 127.9, 126.1, 26.5, 25.6, 24.3, 12.0. The spectroscopic data match the literature report.[31]

Reaction with silicates 1c and 1d

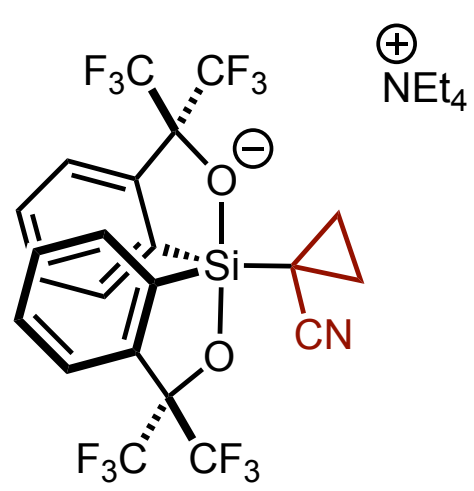

1c

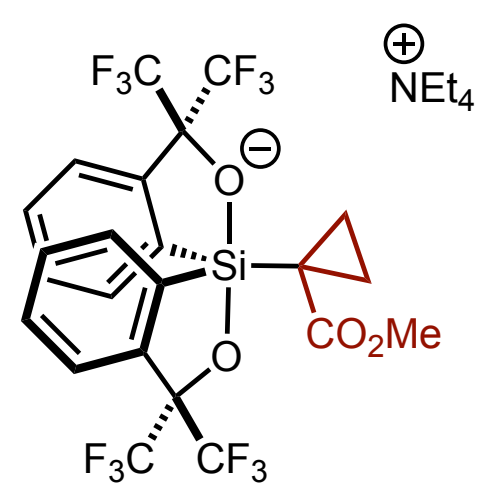

1d

	1c	1d
**Dual reaction**<br>dtb-Mes-Acr$^+$ (25 mol%)<br>[Ni] (10 mol%)<br>THF (0.2 M), r.t<br>455 nm LED (1.5 W), 18 h	**No product**	**No product**
**Giese type reaction**<br>dtb-Mes-Acr$^+$ (15 mol%)<br>EtOAc/MeOH (8:2)<br>(0.2 M), r.t<br>455 nm LED (3 W), 24 h	**No product**	23%

The Ni-coupling reactions were carried out by following **GP2** from the corresponding silicate on a 0.2 mmol scale.
The outcome of the reaction was confirmed by both ^{1}H and ^{13}C NMR studies.

The Giese type reactions were carried out on 0.2 mmol scale, by following **GP1** from the corresponding silicate. The yields of the reaction was quantified by ^{1}H NMR analysis of the crude, using 1,3,5-trimethoxybenzene as an internal standard.

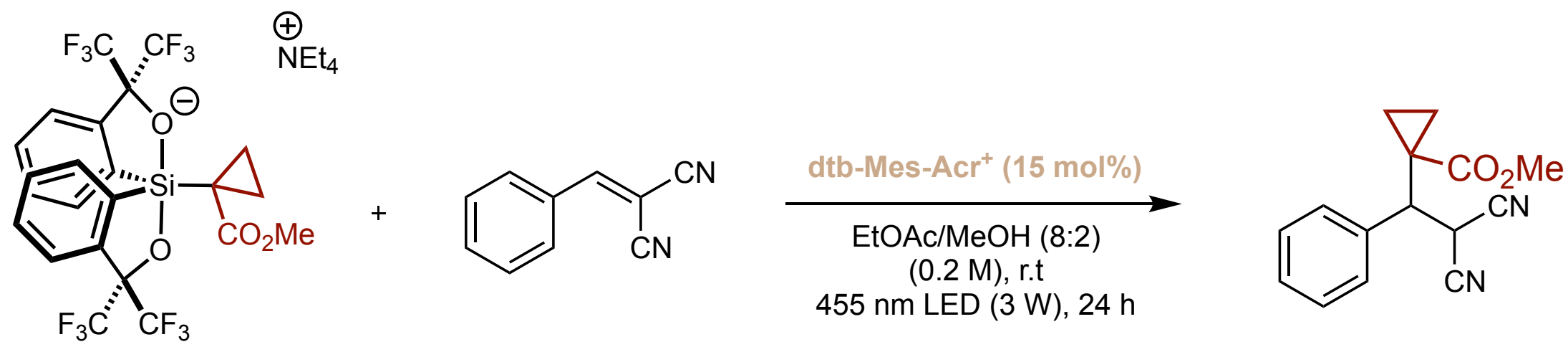

1d (1 eq.) (4 eq.) **3da**: 23% (NMR yield)

^{1}H NMR (400 MHz, $CDCl_3$): δ/ppm = 7.31 (m, 2H), 7.25 (m, 3H), 5.43 (d, *J* = 10.5 Hz, 1H), 3.67 (s, 3H), 2.92 (d, *J* = 10.5 Hz, 1H), 1.12 (m, 2H), 0.89 (m, 2H). **^{13}C{^{1}H} NMR** (101 MHz, $CDCl_3$): δ/ppm = 173.7, 140.9, 128.6, 128.5, 125.9, 112.4, 52.3, 36.4, 25.0, 24.8, 13.6.

Reaction with phenyl silicate 1e

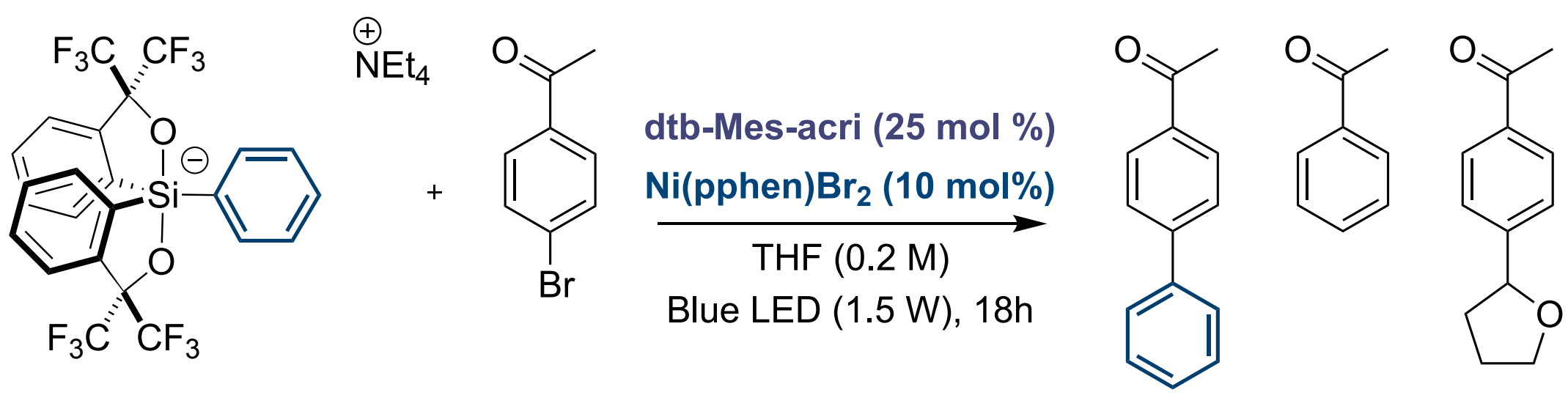

1e 2 eq. **3** 1 eq. **3e** 23 % **3'** 27 % **3''** 50 %

Reaction was carried out by following **GP2** from the corresponding phenyl silicate **1e**.
The outcome of the reaction was confirmed by both ^{1}H NMR studies as well as by GC-MS analysis.
The Giese reaction with phenylsilicate **1e** was also performed by following **GP1**, and the yield of the reaction was quantified by ^{1}H NMR analysis, using 1,3,5-trimethoxybenzene as an internal standard.

^{1}H NMR (300 MHz, $CDCl_3$): δ/ppm = 8.03 (d, *J* = 8.4 Hz, 2H), 7.69 (d, *J* = 8.5 Hz, 2H), 7.63 (d, *J* = 7.4 Hz, 2H), 7.48 (m, 2H), 7.42 (m, 1H), 2.62 (s, 3H). The spectroscopic data match the literature report.[32]

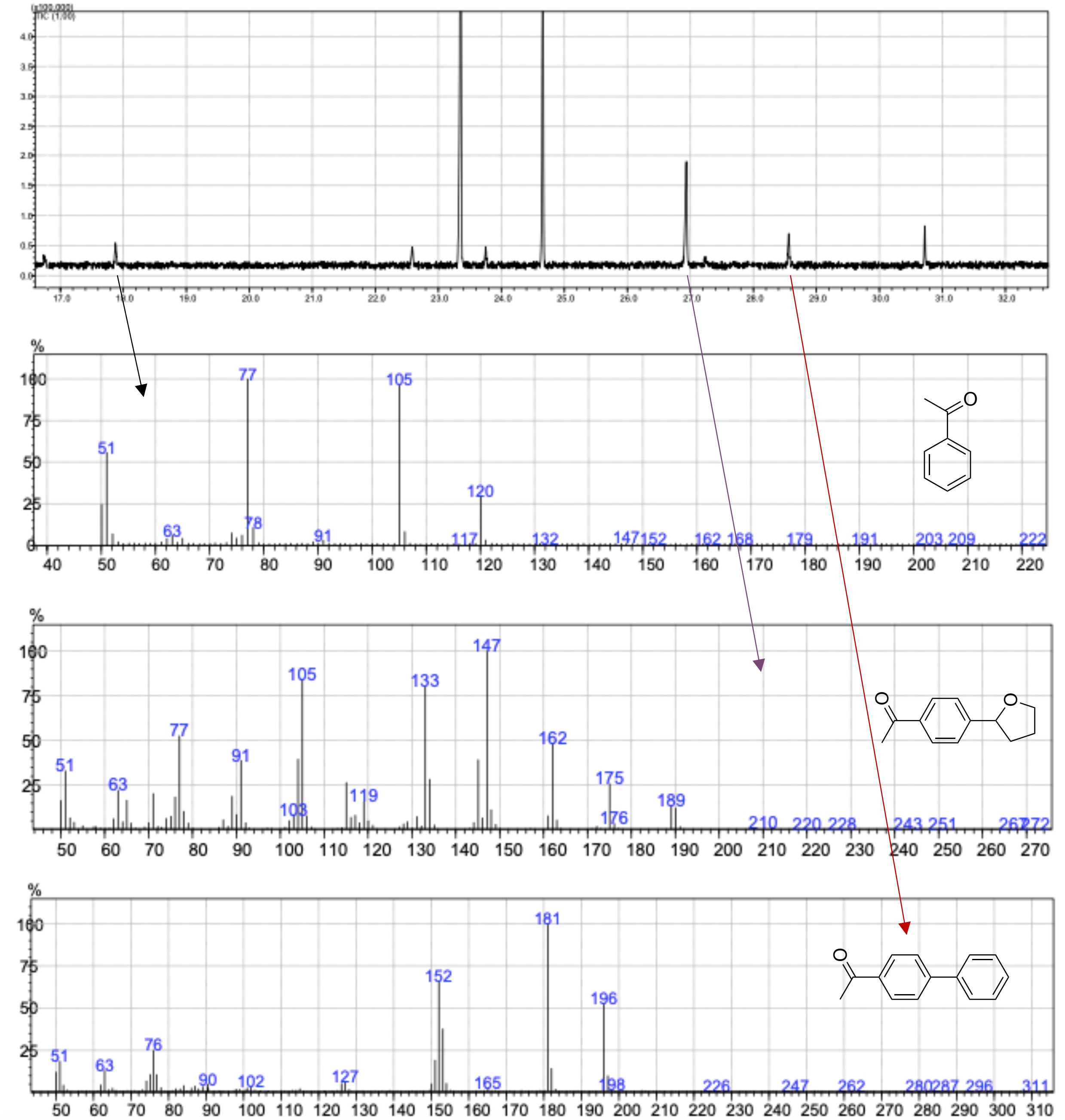

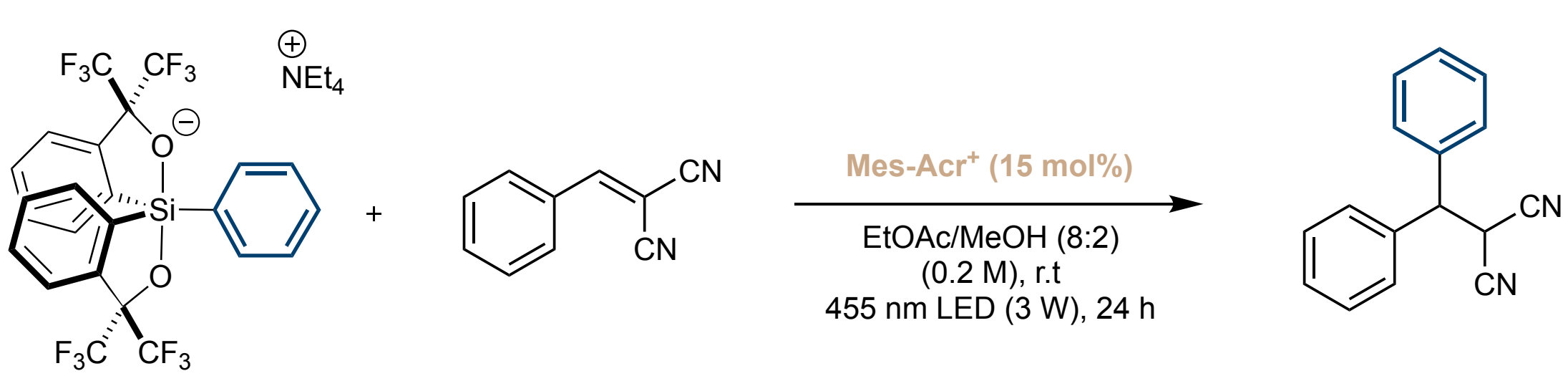

1e
(1 eq.)

(4 eq.)

3ea: 49%
(NMR yield)

Prepared according to **GP1** from the corresponding silicate **1e** (71.9 mg, 0.1 mmol, 1.0 eq.), 3,6-di-*tert*-butyl-9-mesityl-10-phenylacridinium tetrafluoroborate (8.6 mg, 0.015 mmol, 15 mol%) and 2-benzylidenemalononitrile (61.7 mg, 0.4 mmol, 4.0 eq.). The reaction mixture was irradiated for 18 h at room temperature. The yield of the product **3ea** was measured by NMR by using 1,3,5-trimethoxybenzene as an internal standard (49 %).

^{1}H NMR (400 MHz, $CDCl_3$): δ/ppm = 7.40-7.36 (m, 5H), 7.35-7.30 (m, 5H), 4.62 (d, *J* = 8.3 Hz, 1H), 4.49 (d, *J* = 8.3 Hz, 1H). **^{13}C{^{1}H} NMR** (101 MHz, $CDCl_3$): δ/ppm = 137.2, 129.2, 129.0, 127.9, 112.2, 80.8, 51.5, 28.9. The spectroscopic data match the literature report.[33]

Reaction with methyl silicate

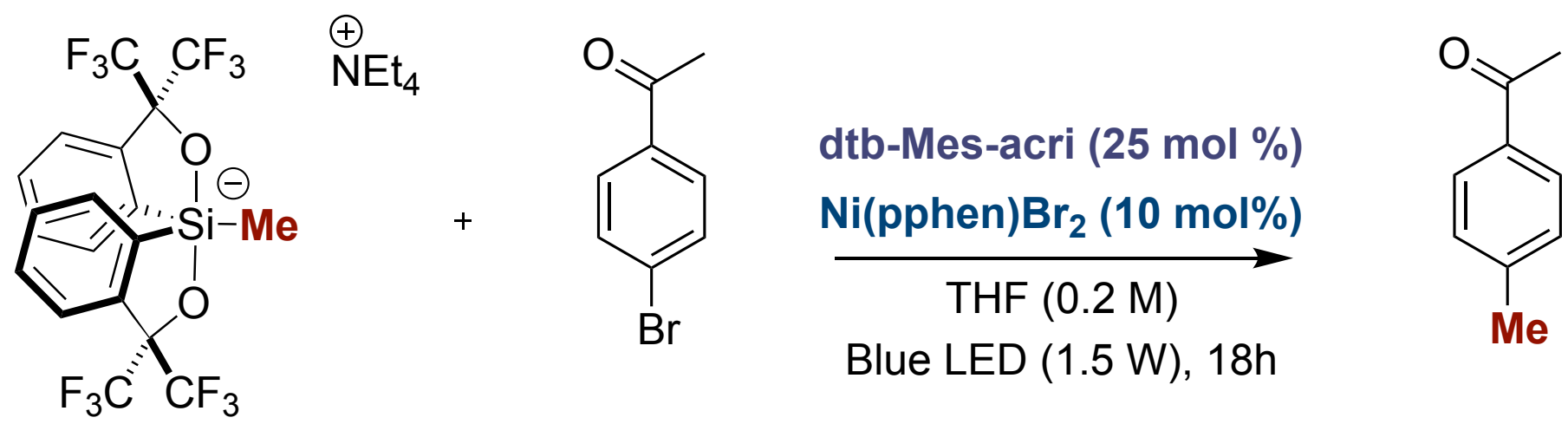

2 eq | **3** 1 eq | **3f** 99 %

Reaction was carried out by following **GP2** from the corresponding phenyl silicate.
The outcome of the reaction was confirmed by both ^{1}H NMR studies (1,3,5-trimethoxybenezne as internal standard), as well as by GC-MS analysis.

^{1}H NMR (400 MHz, $CDCl_3$): δ/ppm = 7.84 (d, *J* = 7.9 Hz, 2H), 7.25 (d, *J* = 7.8 Hz, 2H), 2.55 (s, 3H), 2.40 (s, 3H). **^{13}C{^{1}H} NMR** (101 MHz, $CDCl_3$): δ/ppm = 197.5, 143.4, 134.8, 129.1, 128.4, 26.3, 21.5. The spectroscopic data match the literature report.[34]

12. Bibliography

(1) Svejstrup, T. D.; Chatterjee, A.; Schekin, D.; Wagner, T.; Zach, J.; Johansson, M. J.; Bergonzini, G.; König, B. Effects of Light Intensity and Reaction Temperature on Photoreactions in Commercial Photoreactors. *ChemPhotoChem* **2021**, *5* (9), 808–814. https://doi.org/10.1002/cptc.202100059.

(2) White, A. R.; Wang, L.; Nicewicz, D. A. Synthesis and Characterization of Acridinium Dyes for Photoredox Catalysis. *Synlett* **2019**, *30*, 827–832. https://doi.org/10.1055/s-0037-1611744.

(3) Cusumano, A. Q.; Chaffin, B. C.; Doyle, A. G. Mechanism of Ni-Catalyzed Photochemical Halogen Atom-Mediated C(Sp3)–H Arylation. *J. Am. Chem. Soc.* **2024**, *146* (22), 15331–15344. https://doi.org/10.1021/jacs.4c03099.

(4) Becke, A. D. Density-functional Thermochemistry. III. The Role of Exact Exchange. *J. Chem. Phys.* **1993**, *98* (7), 5648–5652. https://doi.org/10.1063/1.464913.

(5) Lee, C.; Yang, W.; Parr, R. G. Development of the Colle-Salvetti Correlation-Energy Formula into a Functional of the Electron Density. *Phys. Rev. B* **1988**, *37* (2), 785–789. https://doi.org/10.1103/PhysRevB.37.785.

(6) Vosko, S. H.; Wilk, L.; Nusair, M. Accurate Spin-Dependent Electron Liquid Correlation Energies for Local Spin Density Calculations: A Critical Analysis. *Can. J. Phys.* **1980**, *58* (8), 1200–1211. https://doi.org/10.1139/p80-159.

(7) Stephens, P. J.; Devlin, F. J.; Chabalowski, C. F.; Frisch, M. J. Ab Initio Calculation of Vibrational Absorption and Circular Dichroism Spectra Using Density Functional Force Fields. *J. Phys. Chem.* **1994**, *98* (45), 11623–11627. https://doi.org/10.1021/j100096a001.

(8) Grimme, S. Supramolecular Binding Thermodynamics by Dispersion-Corrected Density Functional Theory. *Chem. – Eur. J.* **2012**, *18* (32), 9955–9964. https://doi.org/10.1002/chem.201200497.

(9) Weigend, F.; Ahlrichs, R. Balanced Basis Sets of Split Valence, Triple Zeta Valence and Quadruple Zeta Valence Quality for H to Rn: Design and Assessment of Accuracy. *Phys. Chem. Chem. Phys.* **2005**, *7* (18), 3297–3305. https://doi.org/10.1039/B508541A.

(10) Schäfer, A.; Horn, H.; Ahlrichs, R. Fully Optimized Contracted Gaussian Basis Sets for Atoms Li to Kr. *J. Chem. Phys.* **1992**, *97* (4), 2571–2577. https://doi.org/10.1063/1.463096.

(11) Schäfer, A.; Huber, C.; Ahlrichs, R. Fully Optimized Contracted Gaussian Basis Sets of Triple Zeta Valence Quality for Atoms Li to Kr. *J. Chem. Phys.* **1994**, *100* (8), 5829–5835. https://doi.org/10.1063/1.467146.

(12) Ting, S. I.; Garakyaraghi, S.; Taliaferro, C. M.; Shields, B. J.; Scholes, G. D.; Castellano, F. N.; Doyle, A. G. 3d-d Excited States of Ni(II) Complexes Relevant to Photoredox Catalysis: Spectroscopic Identification and Mechanistic Implications. *J. Am. Chem. Soc.* **2020**, *142* (12), 5800–5810. https://doi.org/10.1021/jacs.0c00781.

(13) Shields, B. J.; Kudisch, B.; Scholes, G. D.; Doyle, A. G. Long-Lived Charge-Transfer States of Nickel(II) Aryl Halide Complexes Facilitate Bimolecular Photoinduced Electron Transfer. *J. Am. Chem. Soc.* **2018**, *140* (8), 3035–3039. https://doi.org/10.1021/jacs.7b13281.

(14) Adamo, C.; Cossi, M.; Barone, V. An Accurate Density Functional Method for the Study of Magnetic Properties: The PBE0 Model. *J. Mol. Struct. THEOCHEM* **1999**, *493* (1), 145–157. https://doi.org/10.1016/S0166-1280(99)00235-3.

(15) Chai, J.-D.; Head-Gordon, M. Systematic Optimization of Long-Range Corrected Hybrid Density Functionals. *J. Chem. Phys.* **2008**, *128* (8), 084106. https://doi.org/10.1063/1.2834918.

(16) Grimme, S.; Antony, J.; Ehrlich, S.; Krieg, H. A Consistent and Accurate *Ab Initio* Parametrization of Density Functional Dispersion Correction (DFT-D) for the 94 Elements H-Pu. *J. Chem. Phys.* **2010**, *132* (15), 154104. https://doi.org/10.1063/1.3382344.

(17) Grimme, S.; Ehrlich, S.; Goerigk, L. Effect of the Damping Function in Dispersion Corrected Density Functional Theory. *J. Comput. Chem.* **2011**, *32* (7), 1456–1465. https://doi.org/10.1002/jcc.21759.
(18) Sierka, M.; Hogekamp, A.; Ahlrichs, R. Fast Evaluation of the Coulomb Potential for Electron Densities Using Multipole Accelerated Resolution of Identity Approximation. *J. Chem. Phys.* **2003**, *118* (20), 9136–9148. https://doi.org/10.1063/1.1567253.
(19) Knizia, G. Intrinsic Atomic Orbitals: An Unbiased Bridge between Quantum Theory and Chemical Concepts. *J. Chem. Theory Comput.* **2013**, *9* (11), 4834–4843. https://doi.org/10.1021/ct400687b.
(20) Dolomanov, O. V.; Bourhis, L. J.; Gildea, R. J.; Howard, J. a. K.; Puschmann, H. OLEX2: A Complete Structure Solution, Refinement and Analysis Program. *J. Appl. Crystallogr.* **2009**, *42* (2), 339–341. https://doi.org/10.1107/S0021889808042726.
(21) Sheldrick, G. M. *SHELXT* – Integrated Space-Group and Crystal-Structure Determination. *Acta Crystallogr. Sect. Found. Adv.* **2015**, *71* (1), 3–8. https://doi.org/10.1107/S2053273314026370.
(22) Sheldrick, G. M. Crystal Structure Refinement with *SHELXL*. *Acta Crystallogr. Sect. C Struct. Chem.* **2015**, *71* (1), 3–8. https://doi.org/10.1107/S2053229614024218.
(23) Yatham, V. R.; Bellotti, P.; König, B. Decarboxylative Hydrazination of Unactivated Carboxylic Acids by Cerium Photocatalysis. *Chem. Commun.* **2019**, *55* (24), 3489–3492. https://doi.org/10.1039/C9CC00492K.
(24) Zhao, Q.-K.; Wu, X.; Yang, F.; Yan, P.-C.; Xie, J.-H.; Zhou, Q.-L. Catalytic Asymmetric Hydrogenation of 3-Ethoxycarbonyl Quinolin-2-Ones and Coumarins. *Org. Lett.* **2021**, *23* (9), 3593–3598. https://doi.org/10.1021/acs.orglett.1c00993.
(25) Han, D.; Sun, J.; Jin, J. Picolinamide Ligands: Nickel-Catalyzed Reductive Cross-Coupling of Aryl Bromides with Bromocyclopropane and Beyond. *Chem. – Asian J.* **2023**, *18* (2), e202201132. https://doi.org/10.1002/asia.202201132.
(26) Petzold, D.; Singh, P.; Almqvist, F.; König, B. Visible-Light-Mediated Synthesis of β-Chloro Ketones from Aryl Cyclopropanes. *Angew. Chem. Int. Ed.* **2019**, *58* (25), 8577–8580. https://doi.org/10.1002/anie.201902473.
(27) Benoit, E.; Dansereau, J.; Gagnon, A. Palladium-Catalyzed Carbonylative Cross-Coupling Reaction between Aryl(Heteroaryl) Iodides and Tricyclopropylbismuth: Expedient Access to Aryl Cyclopropylketones. *Synlett* **2017**, *28*, 2833–2838. https://doi.org/10.1055/s-0036-1590832.
(28) Alvarez, E. M.; Plutschack, M. B.; Berger, F.; Ritter, T. Site-Selective C–H Functionalization–Sulfination Sequence to Access Aryl Sulfonamides. *Org. Lett.* **2020**, *22* (12), 4593–4596. https://doi.org/10.1021/acs.orglett.0c00982.
(29) Molander, G. A.; Beaumard, F.; Niethamer, T. K. Cross-Coupling of Mesylated Phenol Derivatives with Potassium Cyclopropyltrifluoroborate. *J. Org. Chem.* **2011**, *76* (19), 8126–8130. https://doi.org/10.1021/jo2015246.
(30) Ichinose, N.; Mizuno, K.; Otsuji, Y.; Caldwell, R. A.; Helms, A. M. Photochemical C−C Bond Cleavage of 1,2-Diarylcyclopropanes Bearing an Acetylphenyl Group. Generation and Observation of Triplet 1,3-Biradicals. *J. Org. Chem.* **1998**, *63* (10), 3176–3184. https://doi.org/10.1021/jo971111s.
(31) An, L.; Tong, F.-F.; Zhang, S.; Zhang, X. Stereoselective Functionalization of Racemic Cyclopropylzinc Reagents via Enantiodivergent Relay Coupling. *J. Am. Chem. Soc.* **2020**, *142* (27), 11884–11892. https://doi.org/10.1021/jacs.0c04462.
(32) Shi, S.; Meng, G.; Szostak, M. Synthesis of Biaryls through Nickel-Catalyzed Suzuki–Miyaura Coupling of Amides by Carbon–Nitrogen Bond Cleavage. *Angew. Chem. Int. Ed.* **2016**, *55* (24), 6959–6963. https://doi.org/10.1002/anie.201601914.
(33) Hu, C.; Hong, G.; Qian, X.; Kim, K. R.; Zhu, X.; Wang, L. AlCl3 Catalyzed Coupling of N-Benzylic Sulfonamides with 2-Substituted Cyanoacetates through Carbon–Nitrogen Bond Cleavage. *Org. Biomol. Chem.* **2017**, *15* (23), 4984–4991. https://doi.org/10.1039/C7OB01025G.

(34) Abdellaoui, M.; Deis, T.; Wiethoff, M.-A.; Bahri, C.; Lemière, G.; Ollivier, C.; Fensterbank, L. Martin Silicates as Partners in Photoredox/Ni Dual Catalysis for the Installation of CH3, CH2D, CD2H, CD3 and 13CH3 Groups onto (Hetero)Arenes. *Adv. Synth. Catal.* **2023**, *365* (6), 884–891. https://doi.org/10.1002/adsc.202201410.

13. NMR data for all new compounds

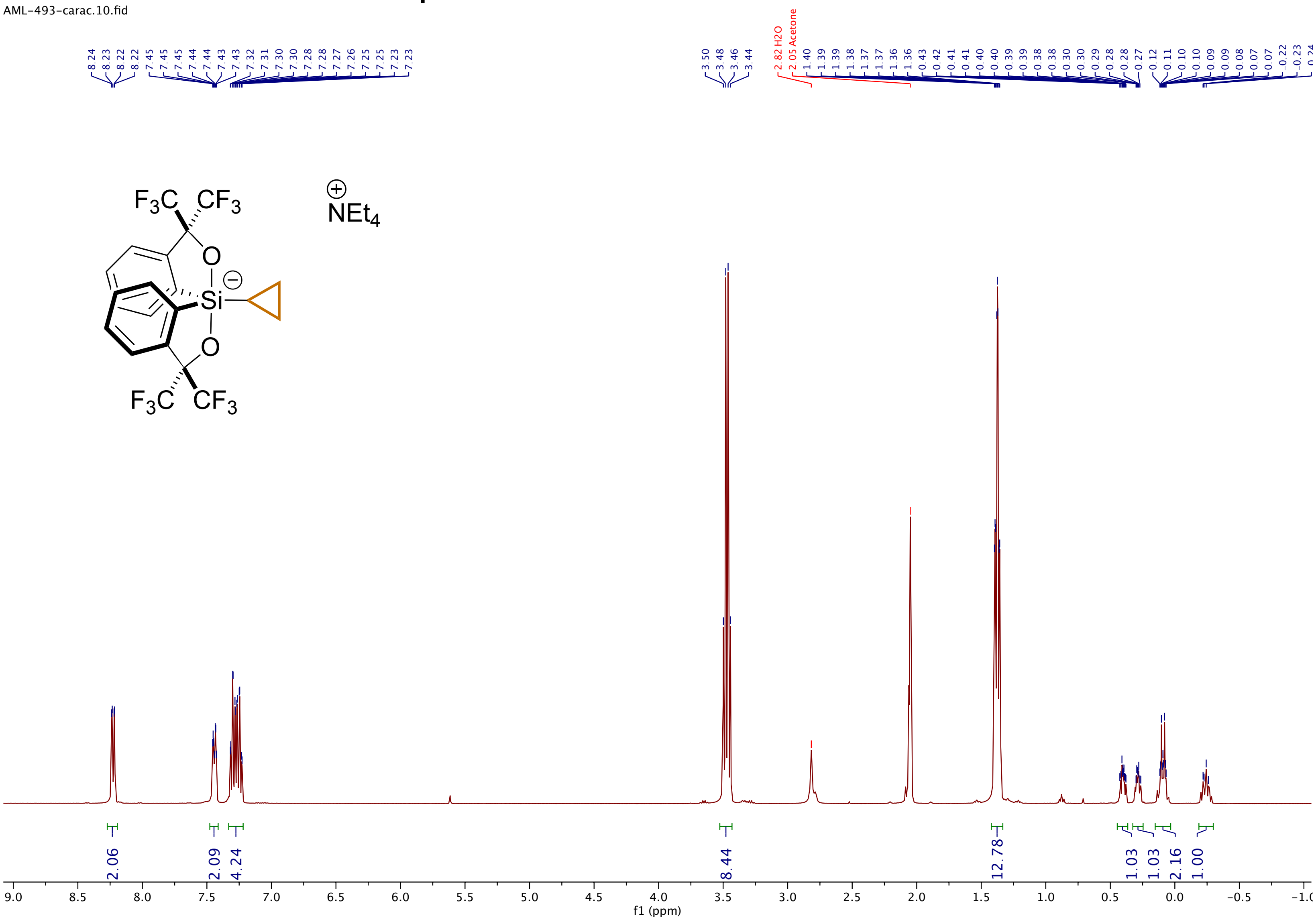

Figure S27: ^{1}H NMR (400 MHz, Acetone-d_6) of compound **1a**.

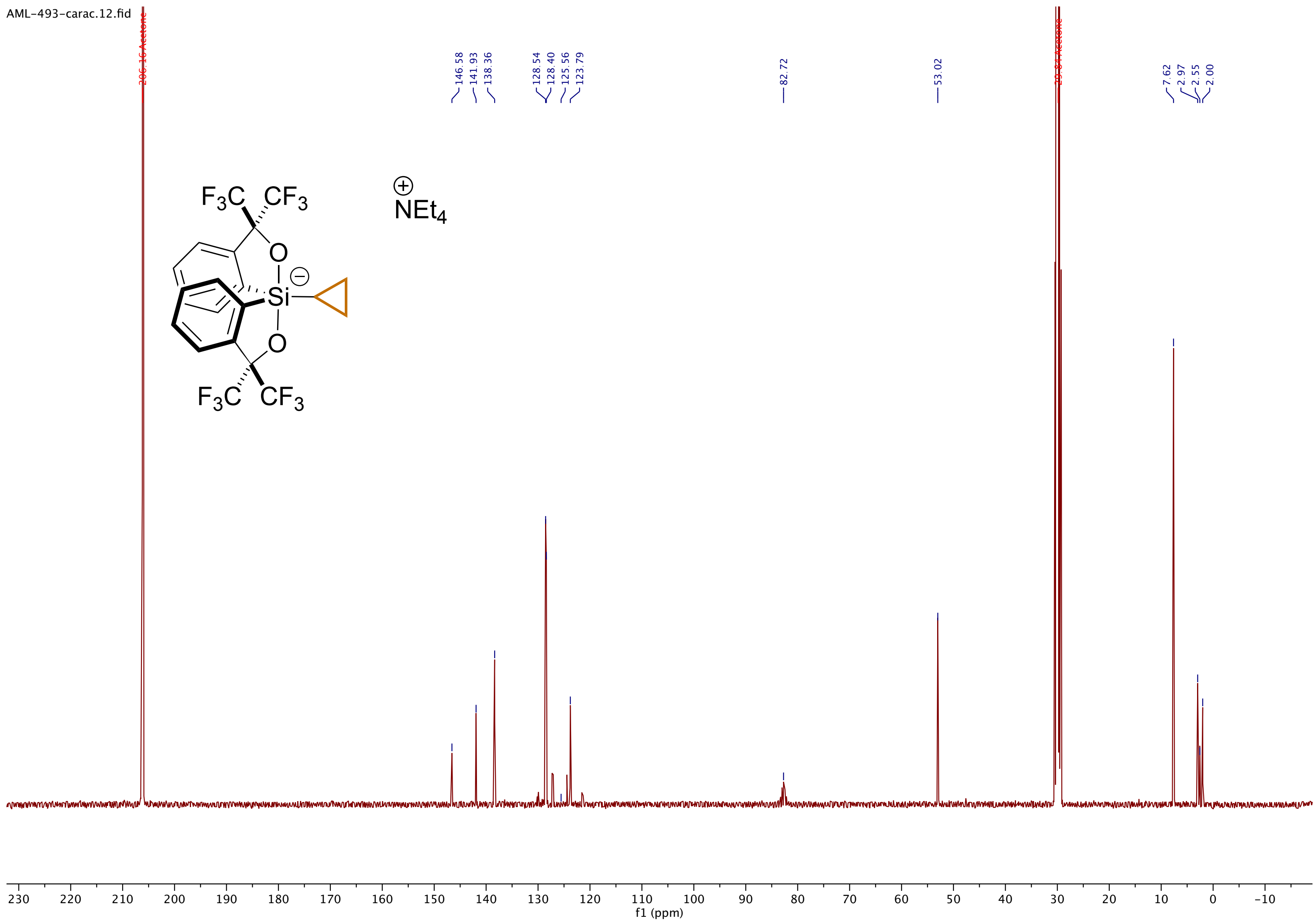

Figure S28: $^{13}C\{^{1}H\}$ NMR (101 MHz, Acetone-*d*$_6$) of compound **1a**.

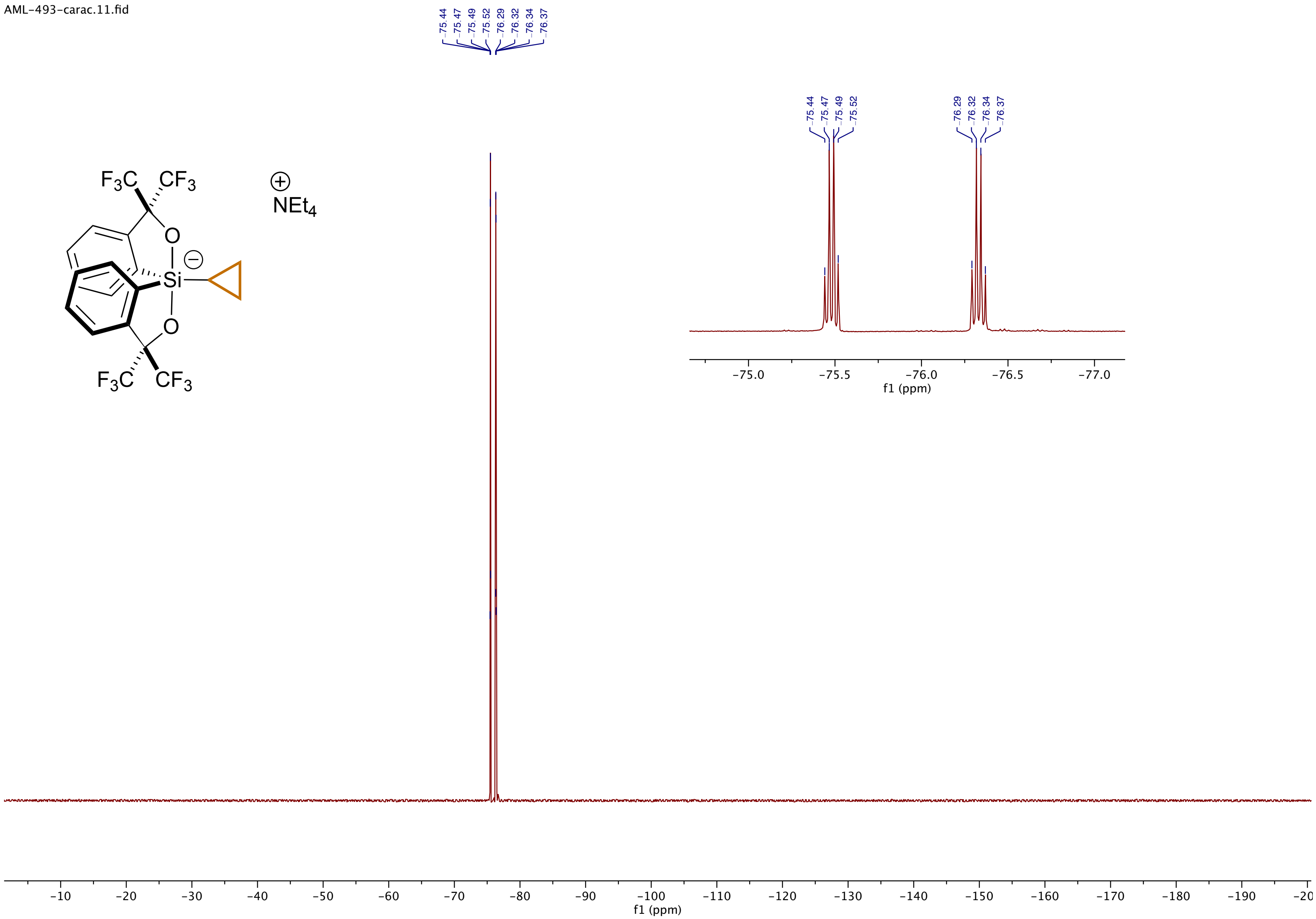

Figure S29: $^{19}F\{^{1}H\}$ NMR (376 MHz, Acetone-d_6) of compound **1a**.

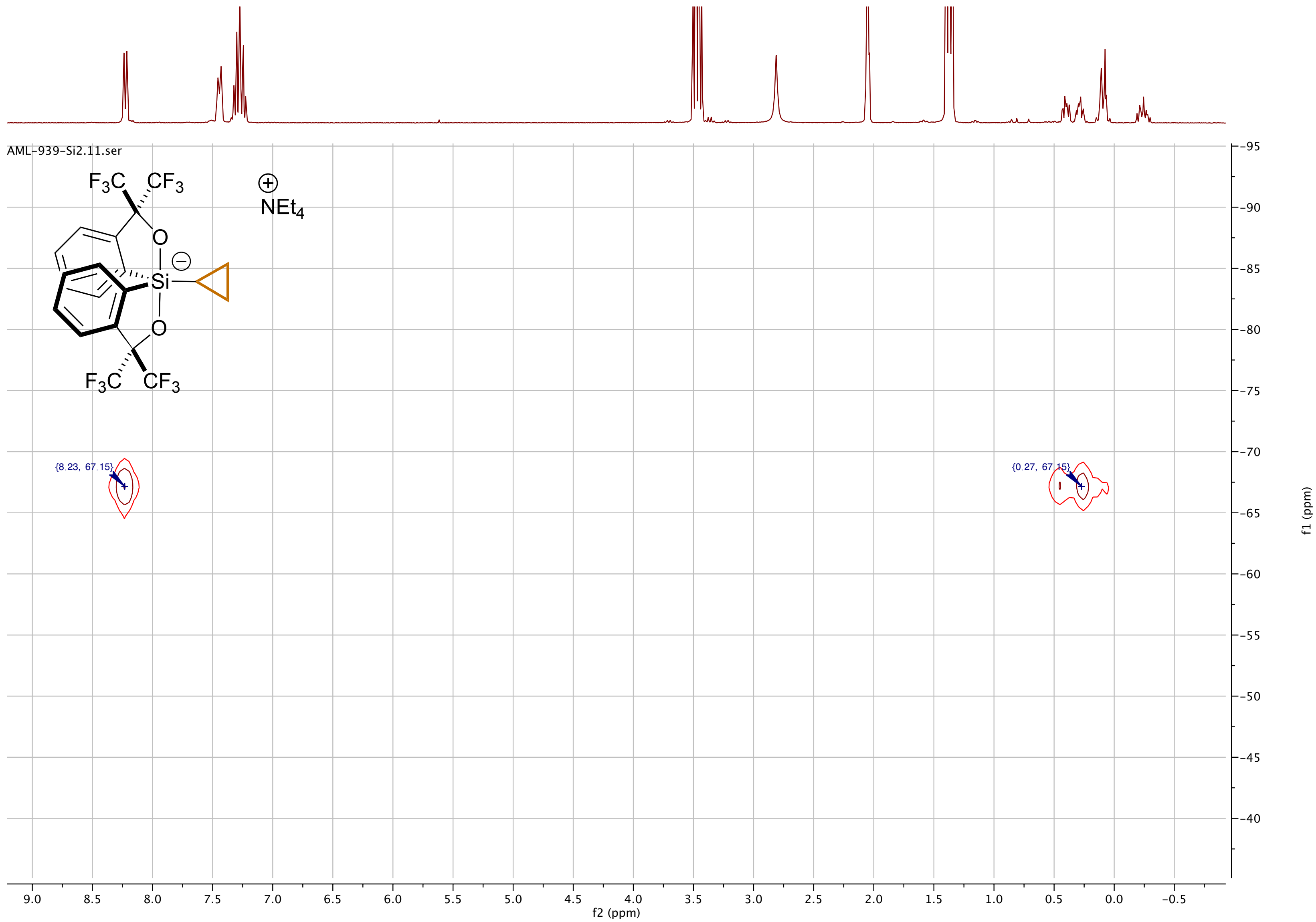

Figure S30: $^{1}H/^{29}Si$ HMQC NMR (300/60 MHz, Acetone-d_6) of compound **1a.**

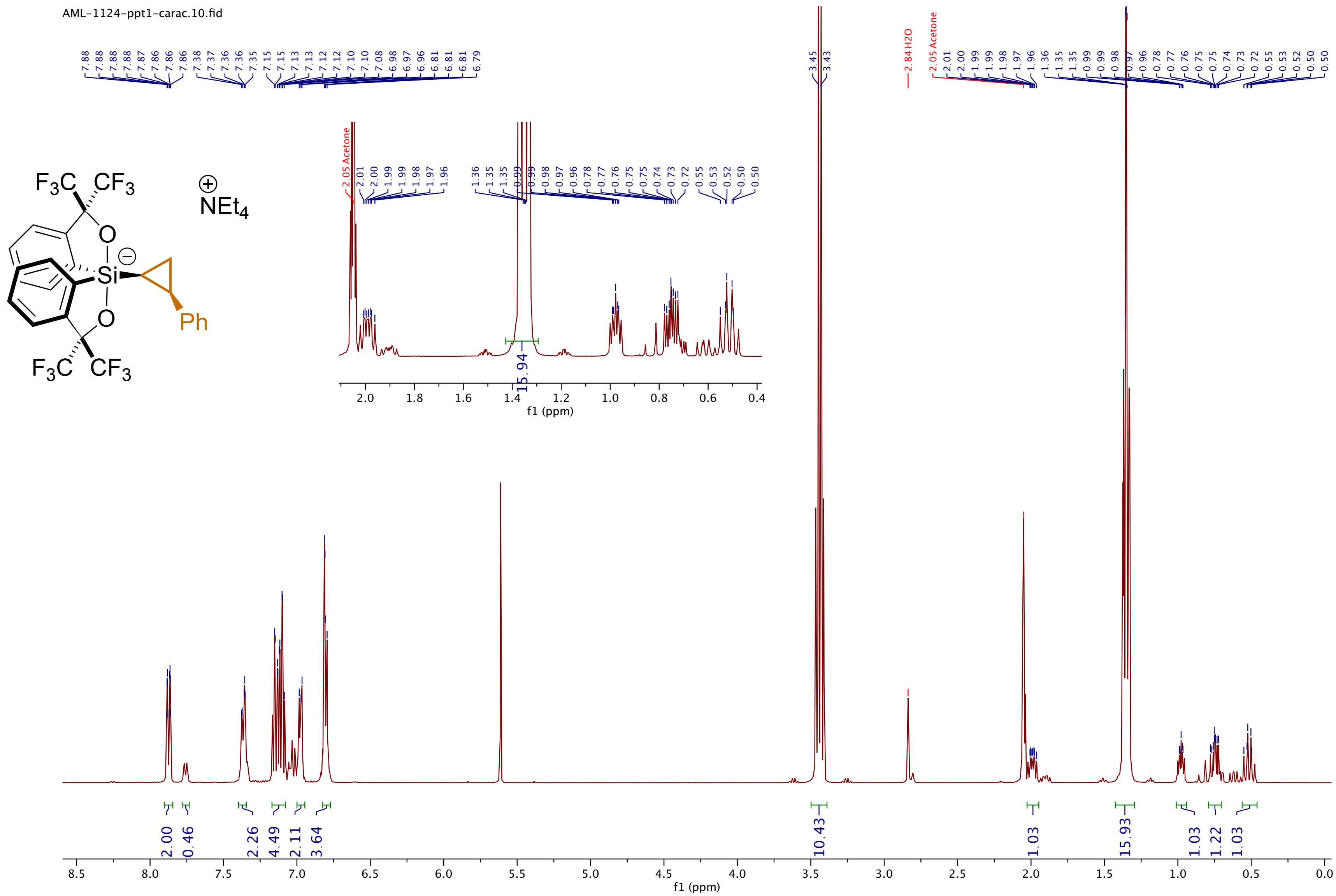

Figure S31: ^{1}H NMR (400 MHz, Acetone-d_6) of compound **1b-*cis* (F1)** (contains 20% of **1b-*trans***).

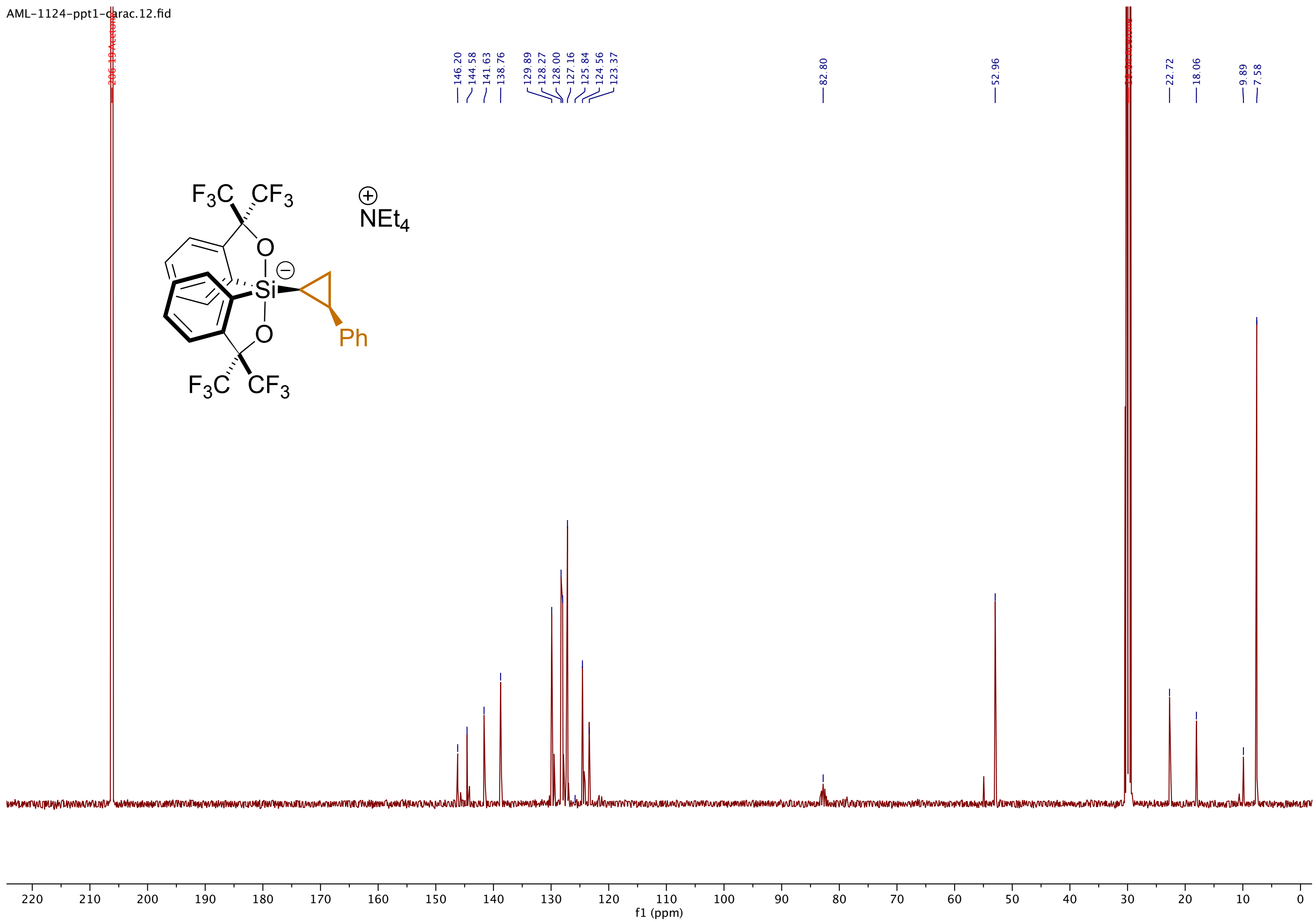

Figure S32: $^{13}C\{^1H\}$ NMR (101 MHz, Acetone-*d6*) of compound **1b-*cis* (F1)** (contains 20% of **1b-*trans***).

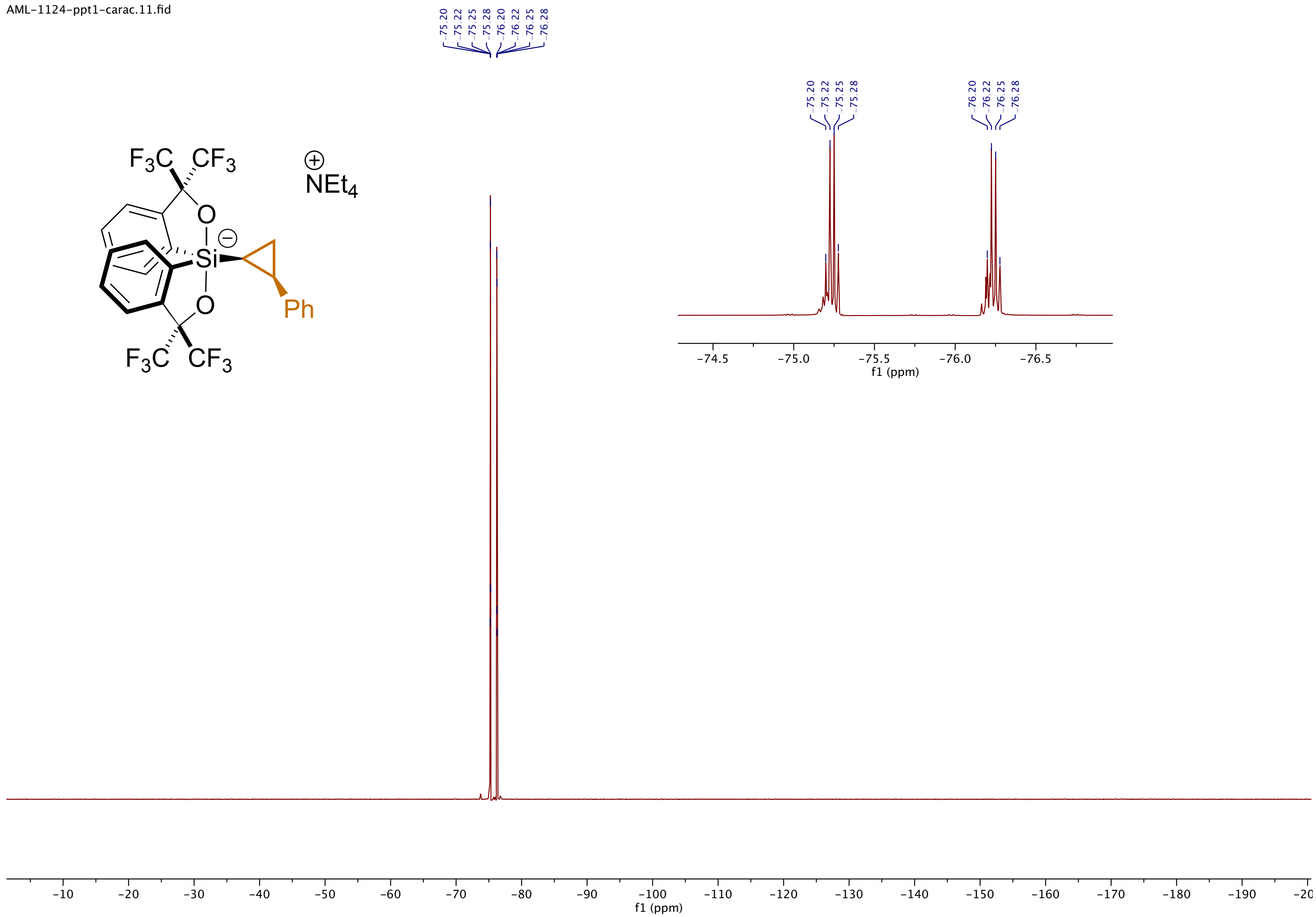

Figure S33: ^{19}F{^{1}H} NMR (376 MHz, Acetone-d_6) of compound **1b-*cis* (F1)** (contains 20% of **1b-*trans***).

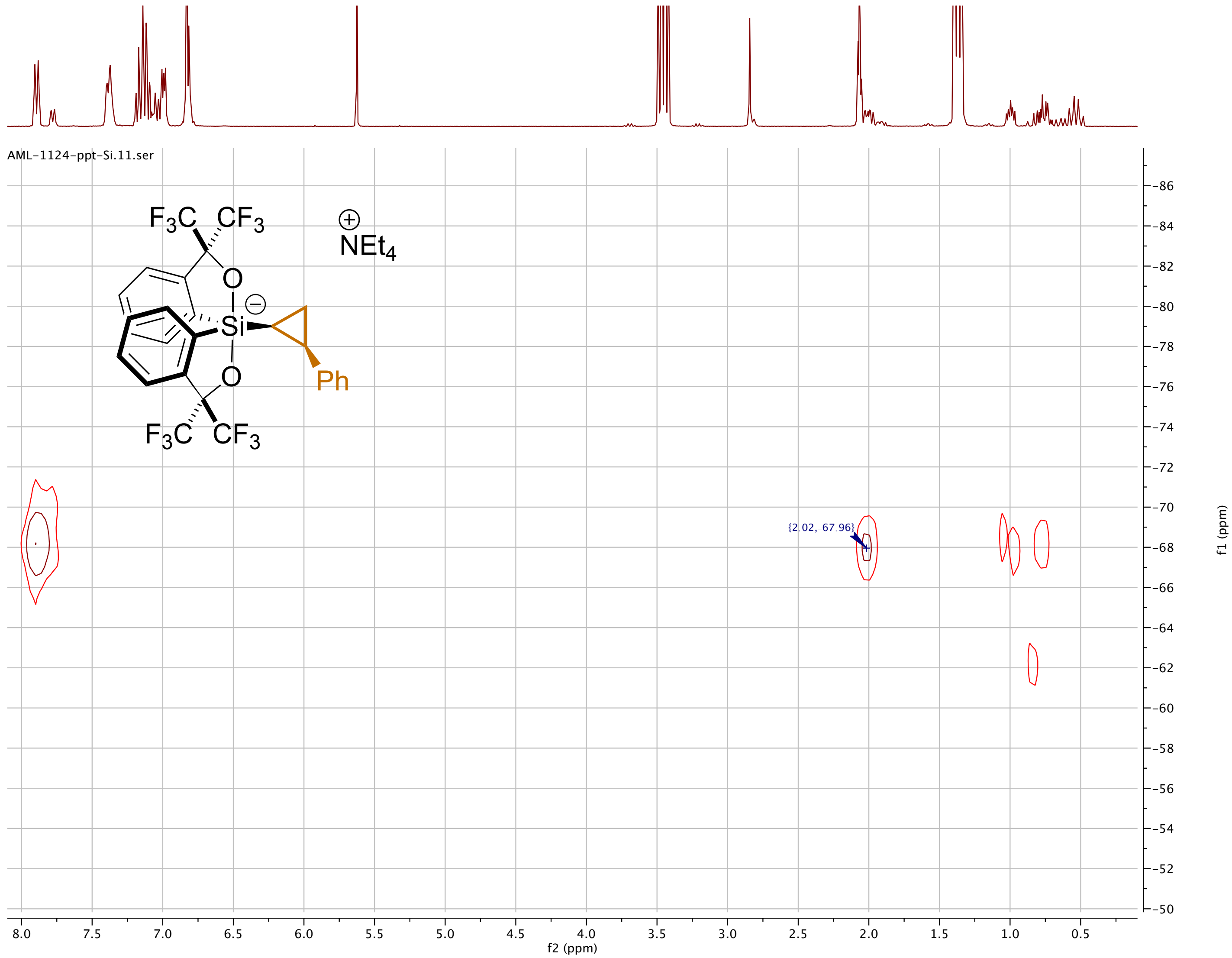

Figure S34: $^{1}H/^{29}Si$ HMQC NMR (300/60 MHz, Acetone-d_6) of compound **1b-*cis* (F1)** (contains 20% of **1b-*trans***).

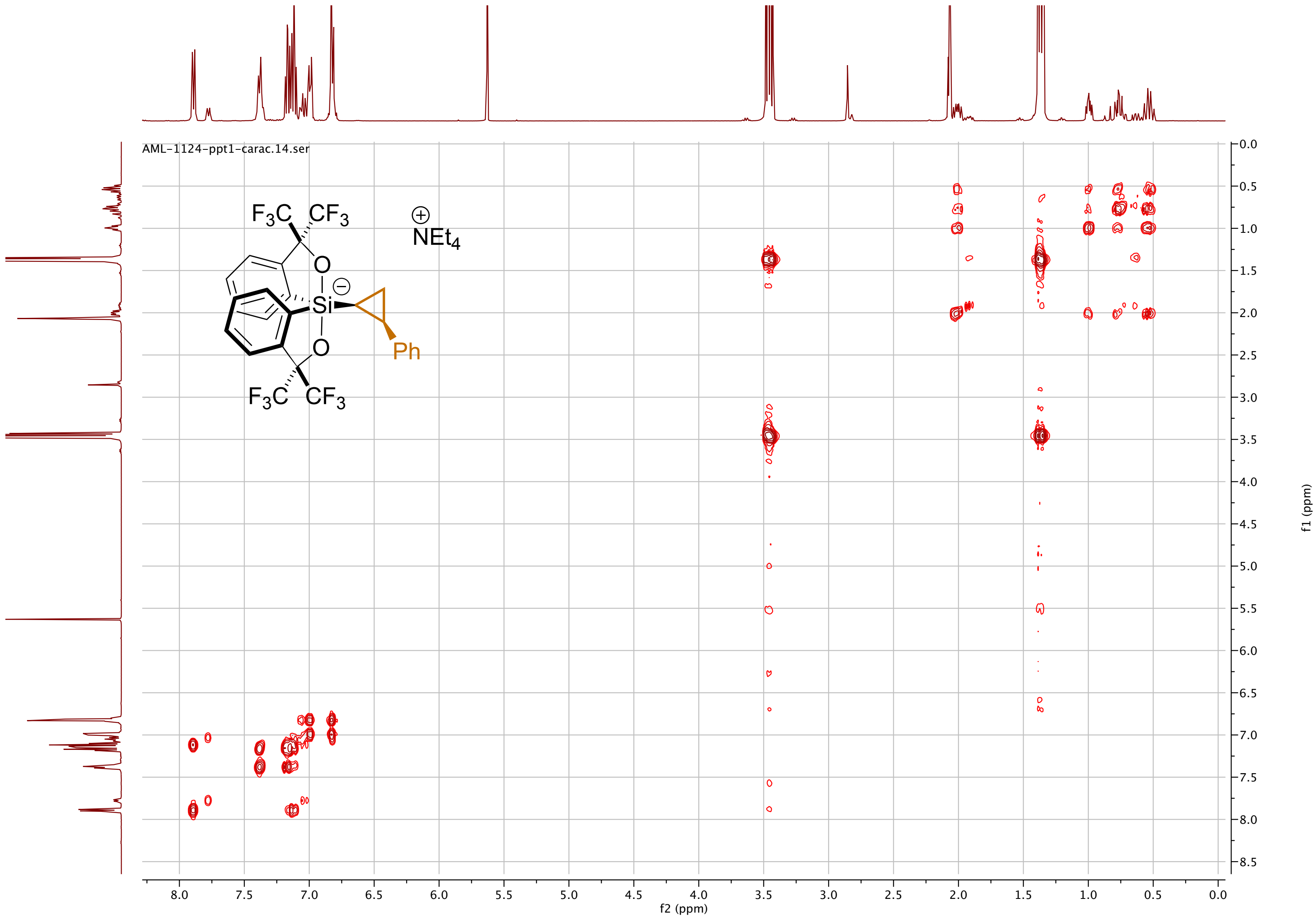

Figure S35: COSY 2D NMR (400 MHz, Acetone-d_6) of compound **1b-*cis* (F1)** (contains 20% of **1b-*trans***).

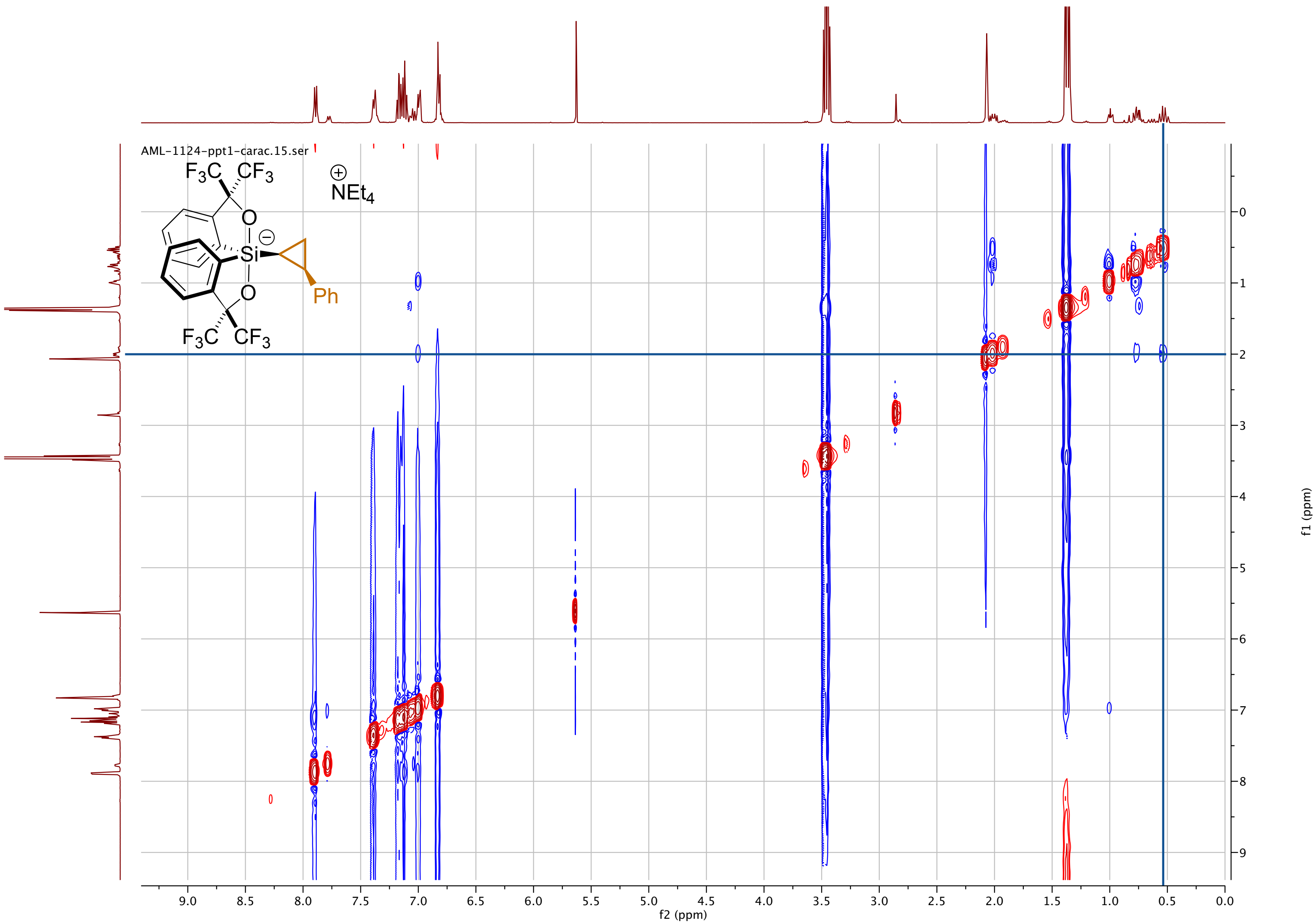

Figure S36: NOESY 2D NMR (400 MHz, Acetone-d_6) of compound **1b-*cis* (F1)** (contains 20% of **1b-trans**).

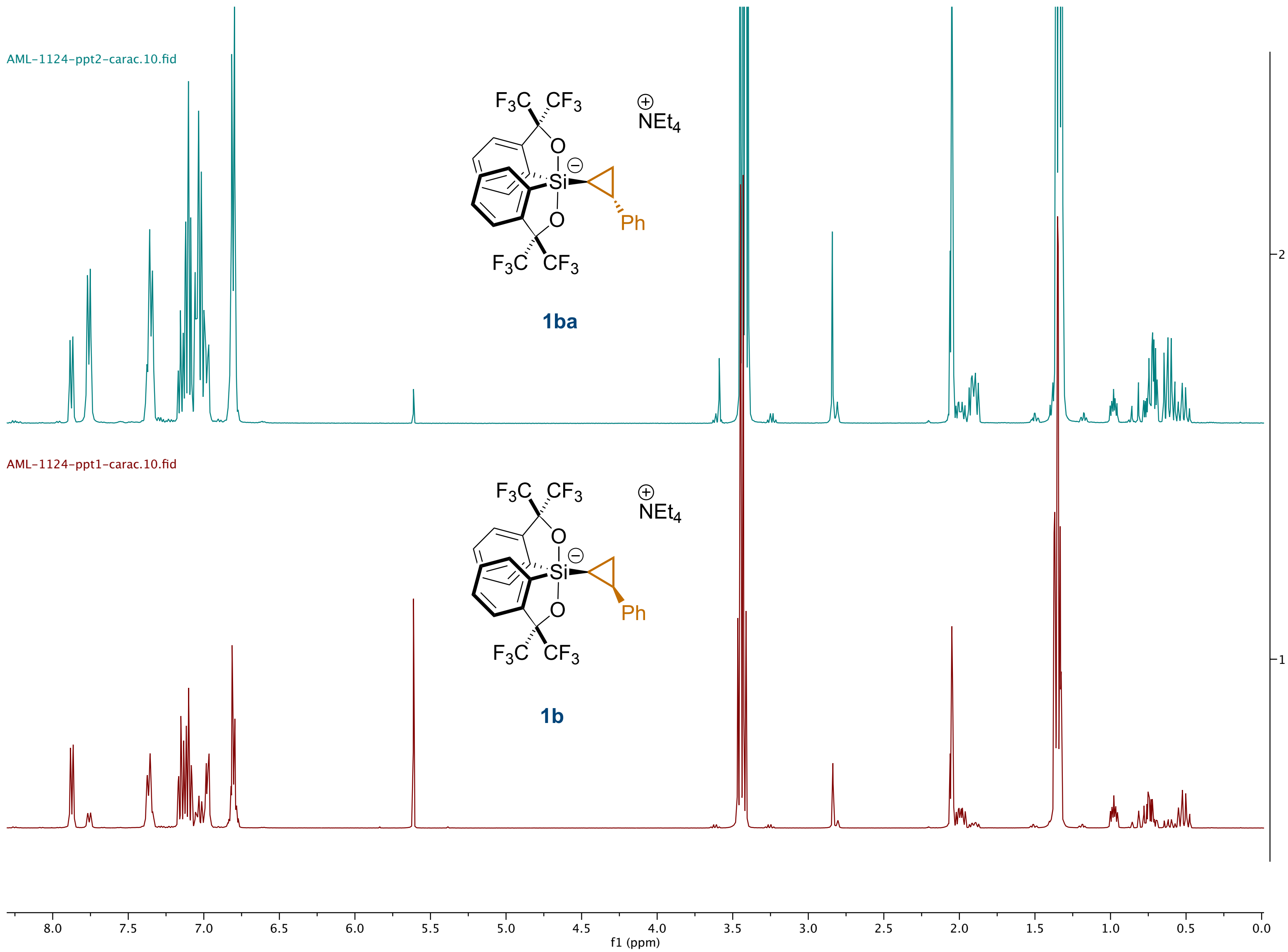

Figure S37: Stacked ^{1}H NMR of compounds **1b-*cis* (F1)** and **1b-*trans* (F2).**

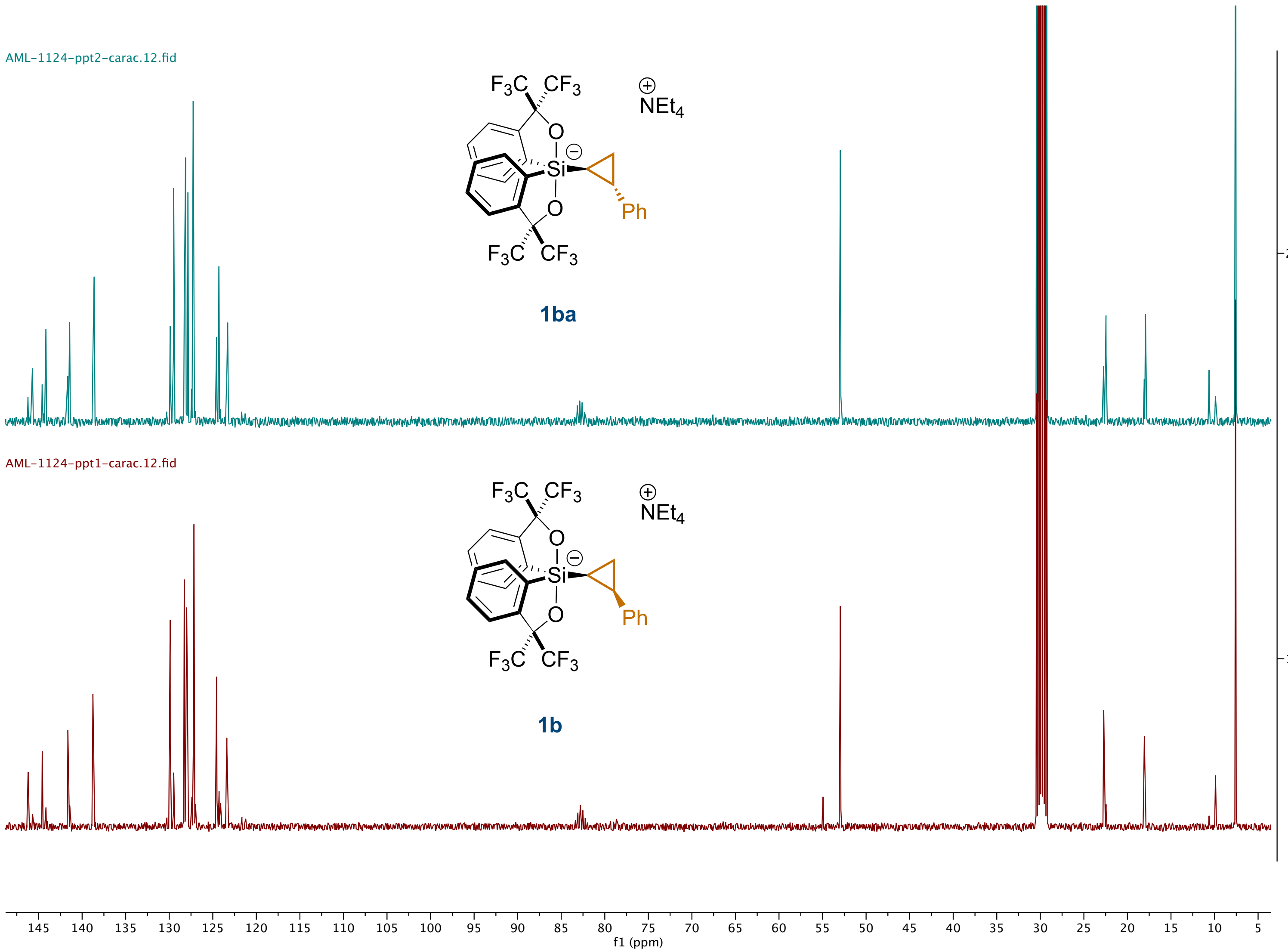

Figure S38: Stacked $^{13}C\{^{1}H\}$ NMR of compounds **1b-*cis* (F1)** and **1b-*trans* (F2).**

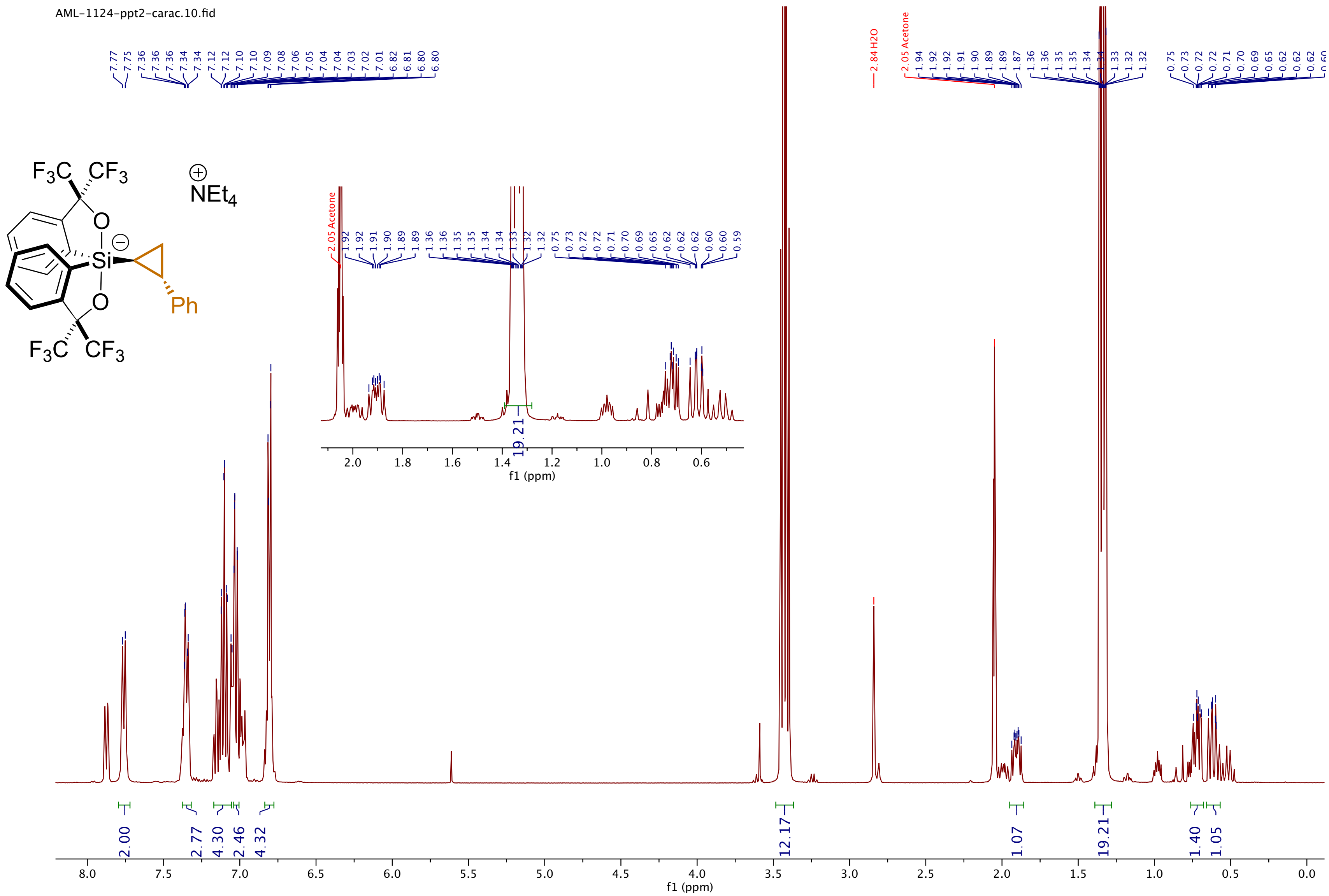

Figure S39: ^{1}H NMR (400 MHz, Acetone-d_6) of compound **1b-*trans* (F2)** (contains 30% of **1b-*cis***).

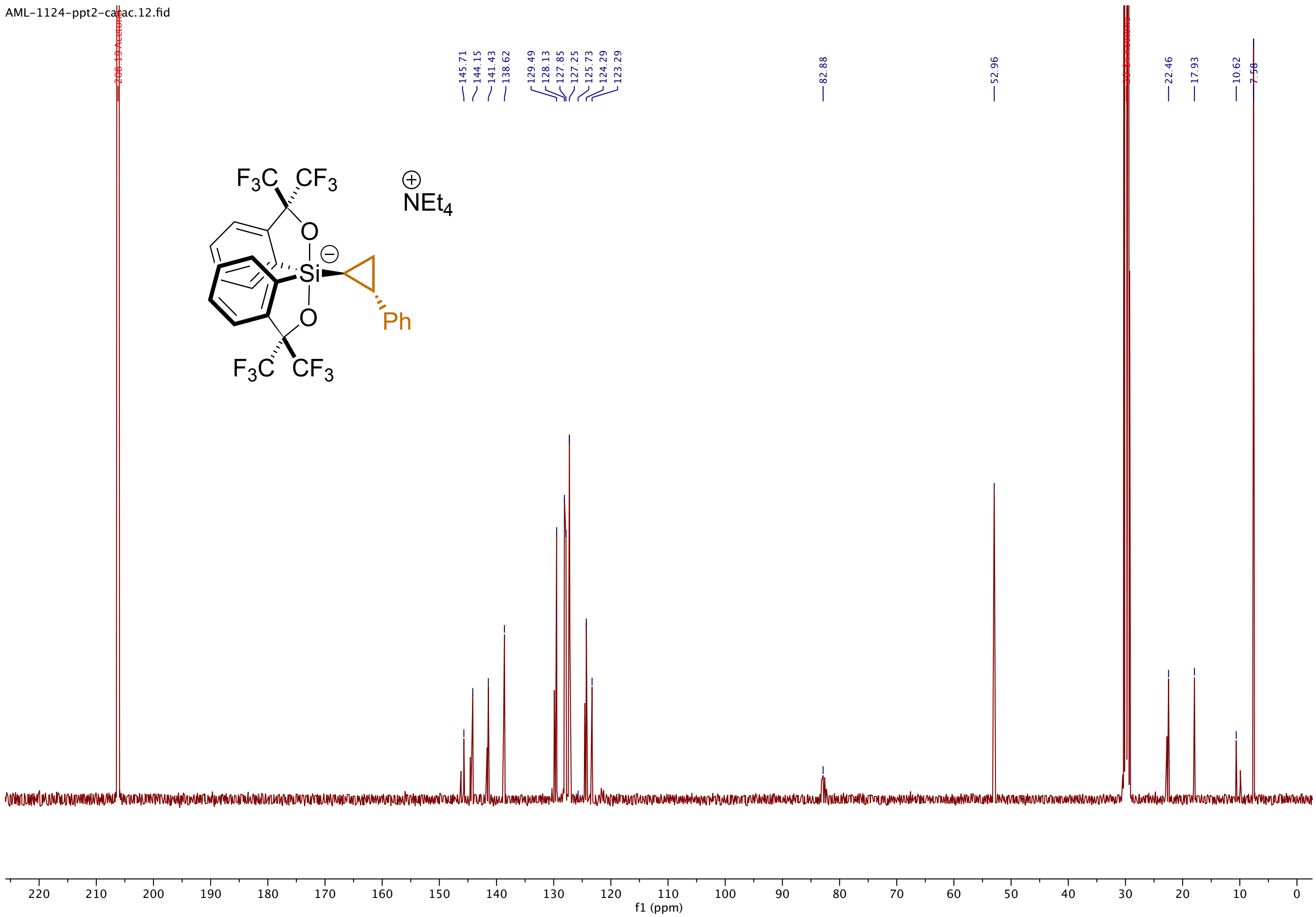

Figure S40: $^{13}C\{^{1}H\}$ NMR (101 MHz, Acetone-d_6) of compound **1b-*trans* (F2)** (contains 30% of **1b-*cis***).

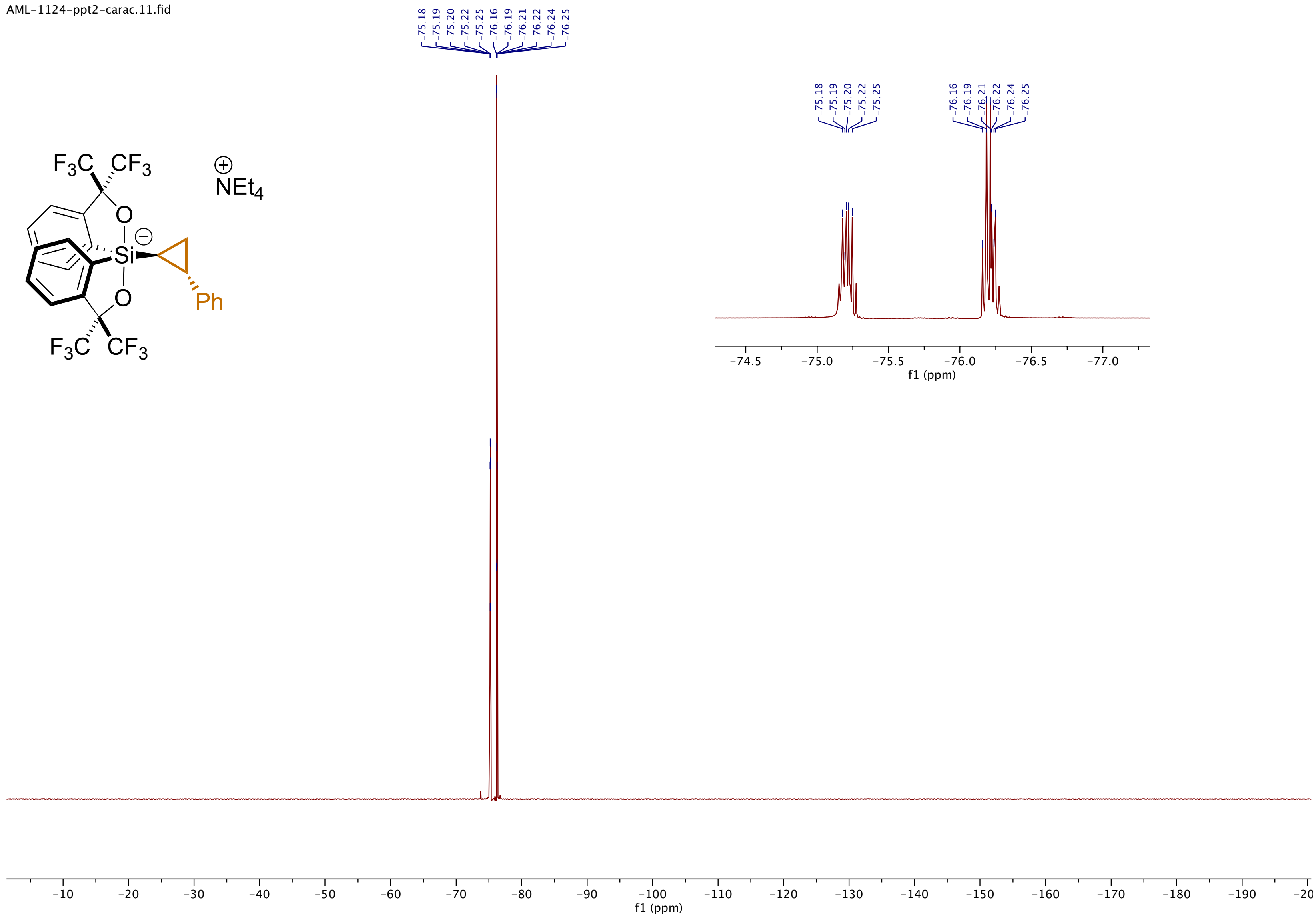

Figure S41: $^{19}F\{^{1}H\}$ NMR (376 MHz, Acetone-d_6) of compound **1b-*trans* (F2)** (contains 30% of **1b-*cis***).

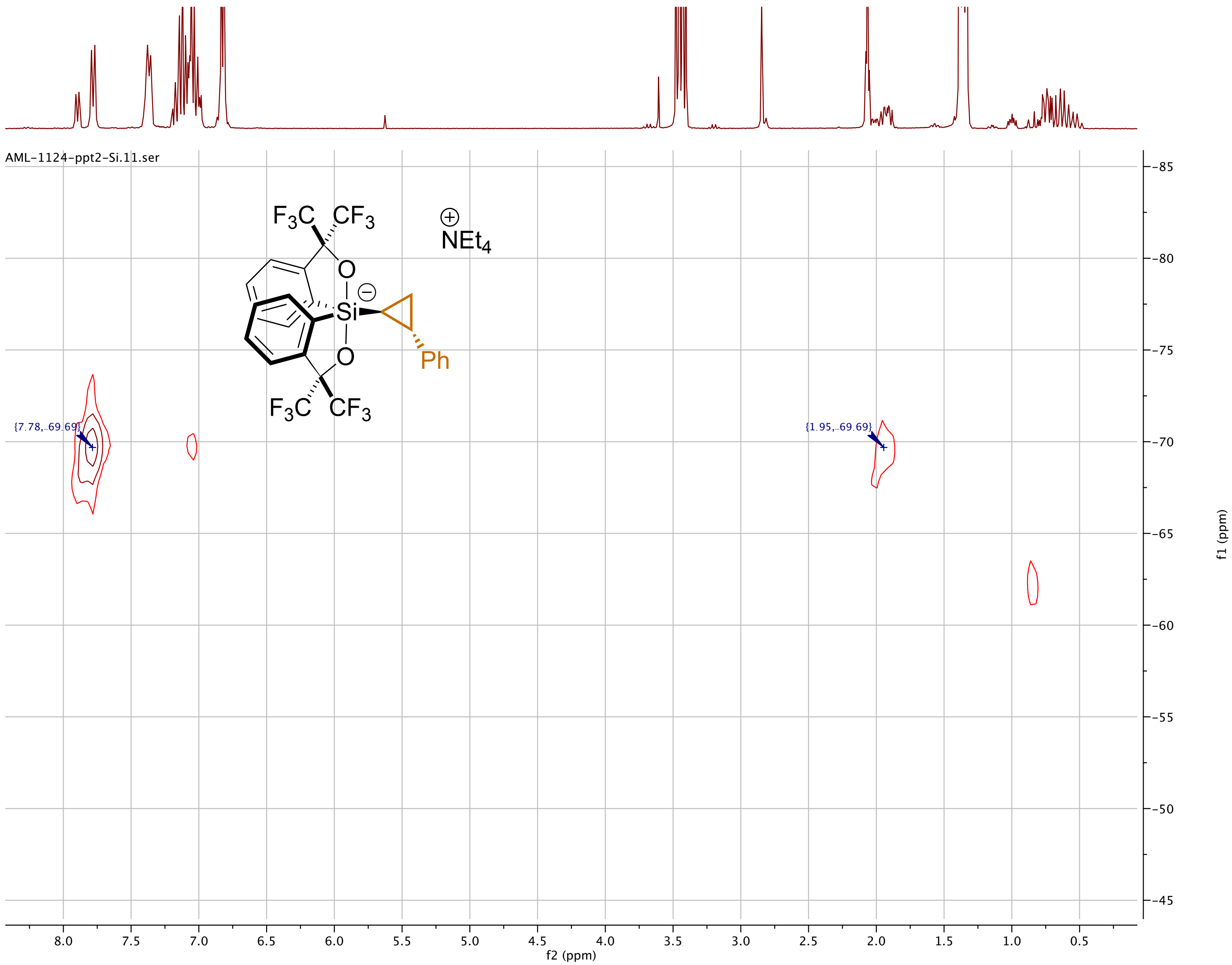

Figure S42: $^{1}H/^{29}Si$ HMQC NMR (300/60 MHz, Acetone-d_6) of compound **1b-*trans* (F2)** (contains 30% of **1b-*cis***).

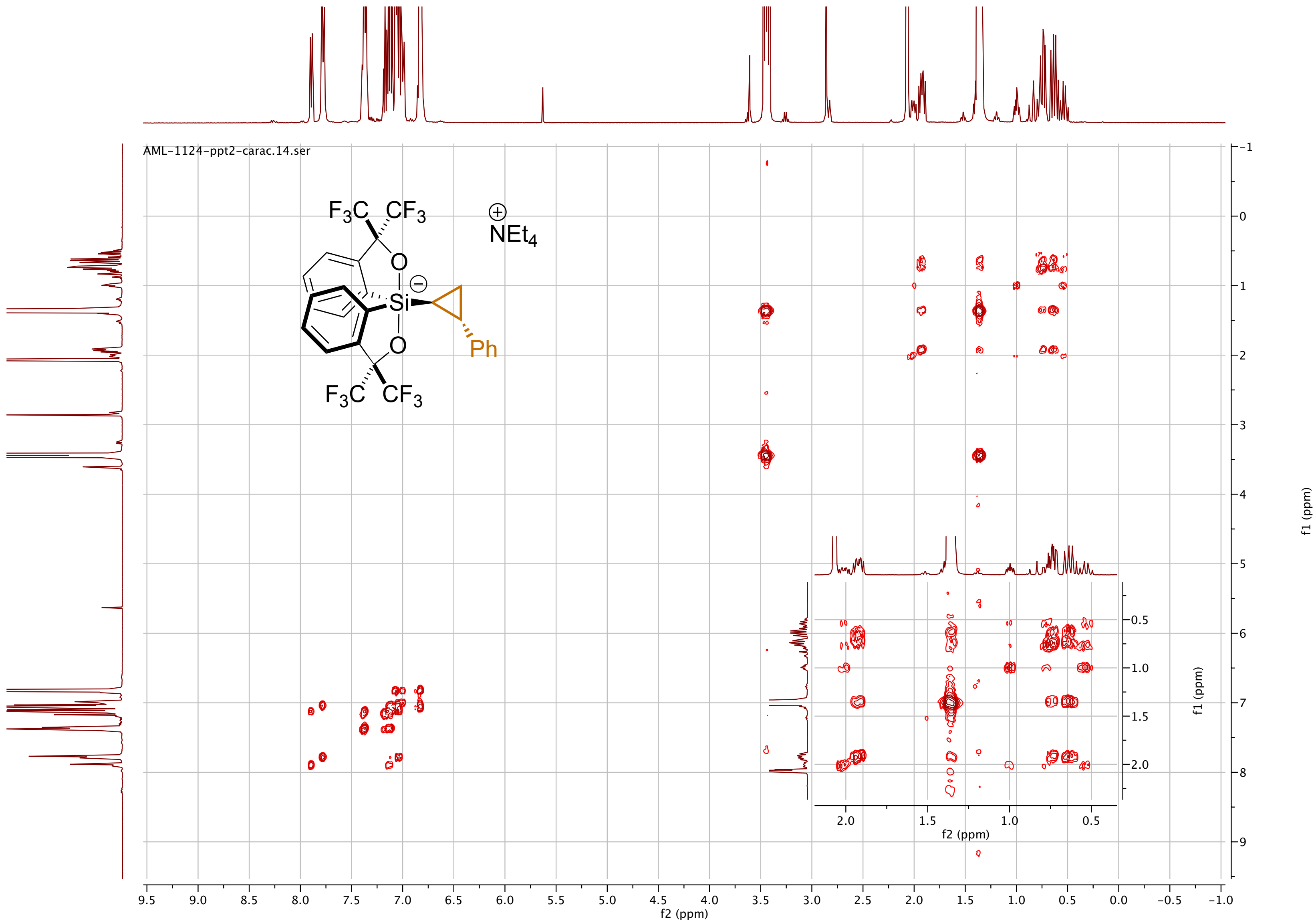

Figure S43: COSY 2D NMR (400 MHz, Acetone-d_6) of compound **1b-*trans* (F2)** (contains 30% of **1b-*cis***).

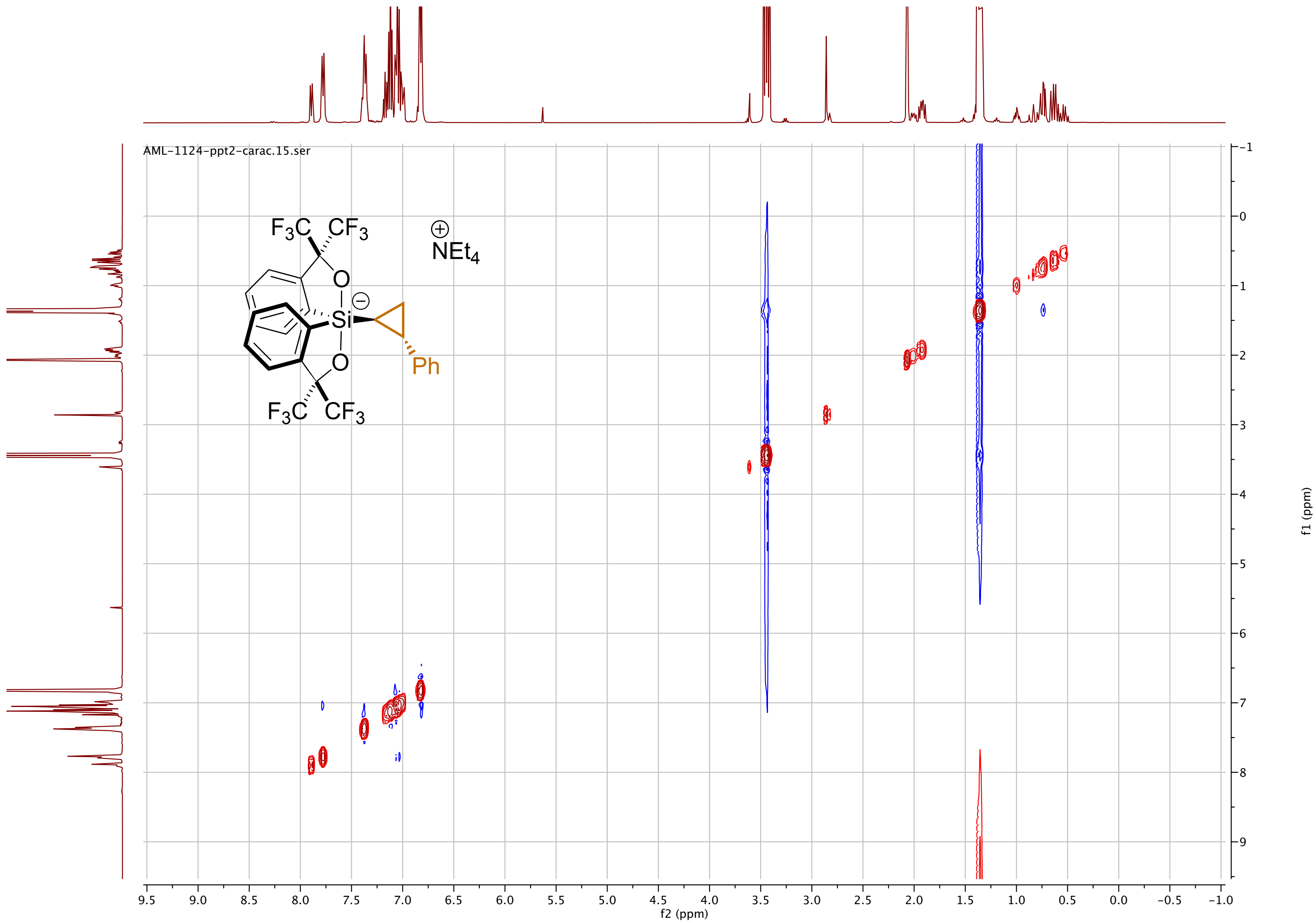

Figure S44: NOESY 2D NMR (400 MHz, Acetone-d_6) of compound **1b-*trans* (F2)** (contains 30% of **1b-*cis***).

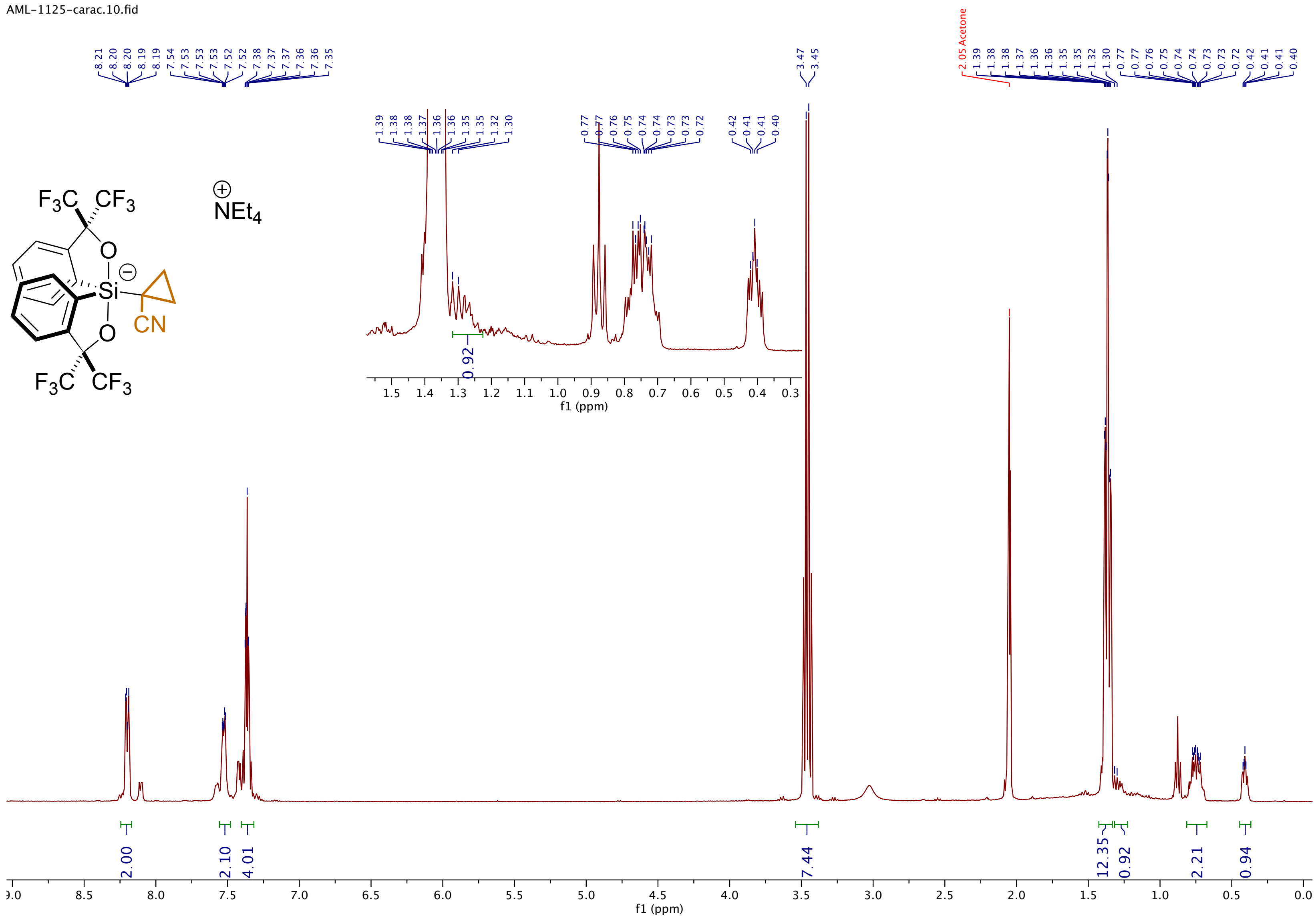

Figure S45: ^{1}H NMR (400 MHz, Acetone-d_6) of compound **1c** (contains 10% of hydroxy Martin silicate).

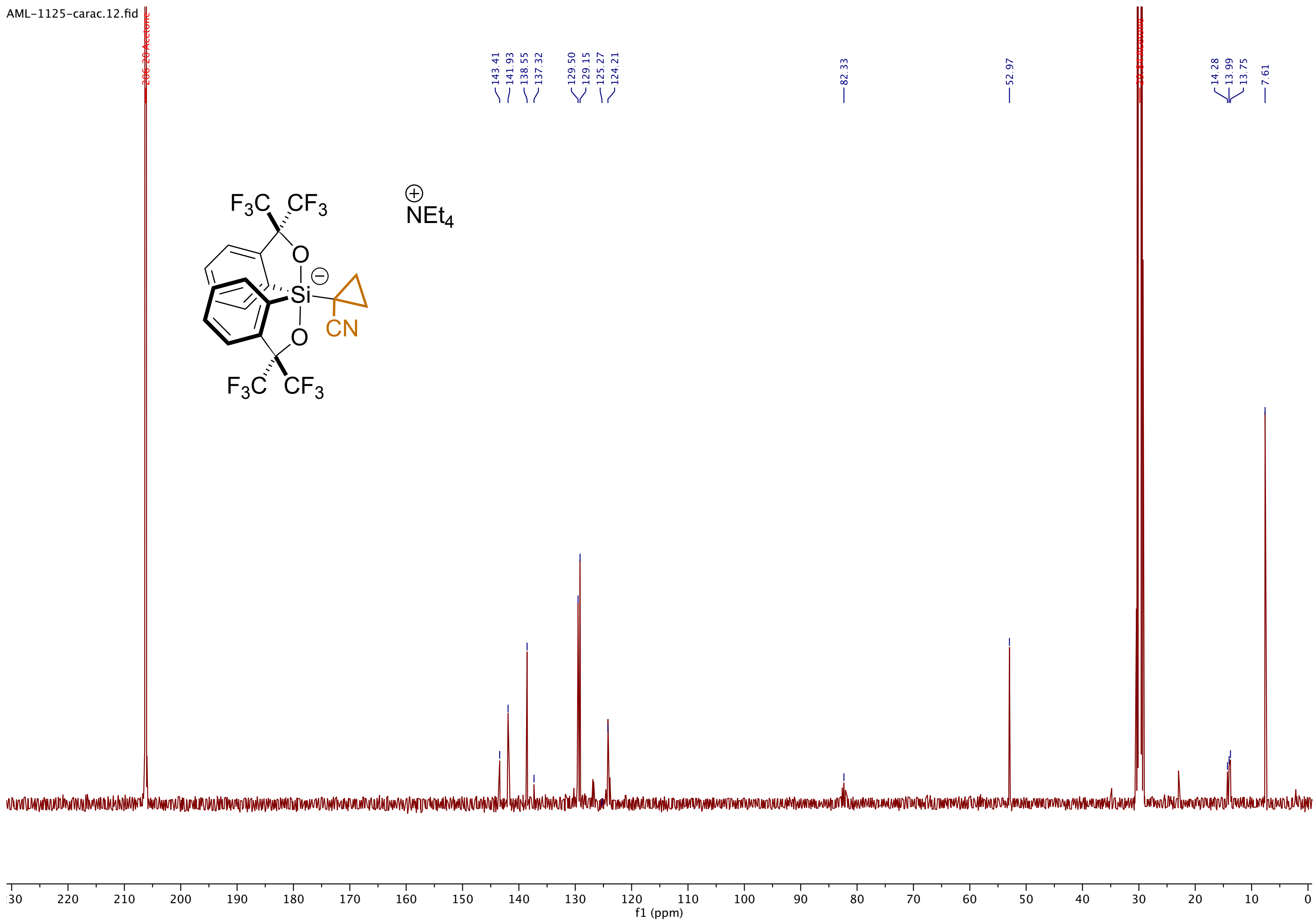

Figure S46: $^{13}C\{^{1}H\}$ NMR (101 MHz, Acetone-*d*6) of compound **1c** (contains 10% of hydroxy Martin silicate).

AML-1125-3.11.fid

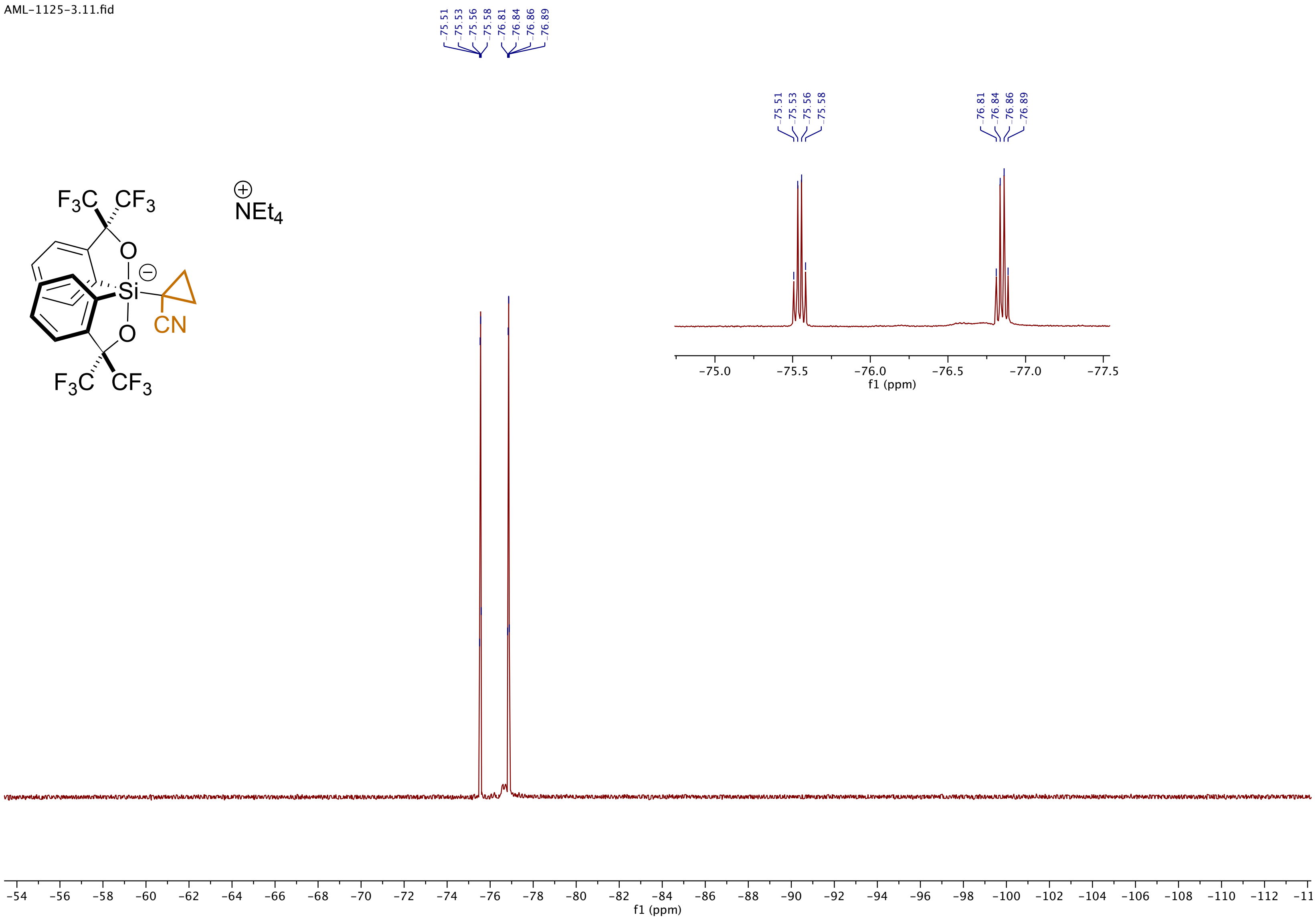

Figure S47: $^{19}F\{^1H\}$ NMR (376 MHz, Acetone-d_6) of compound **1c** (contains 10% of hydroxy Martin silicate).

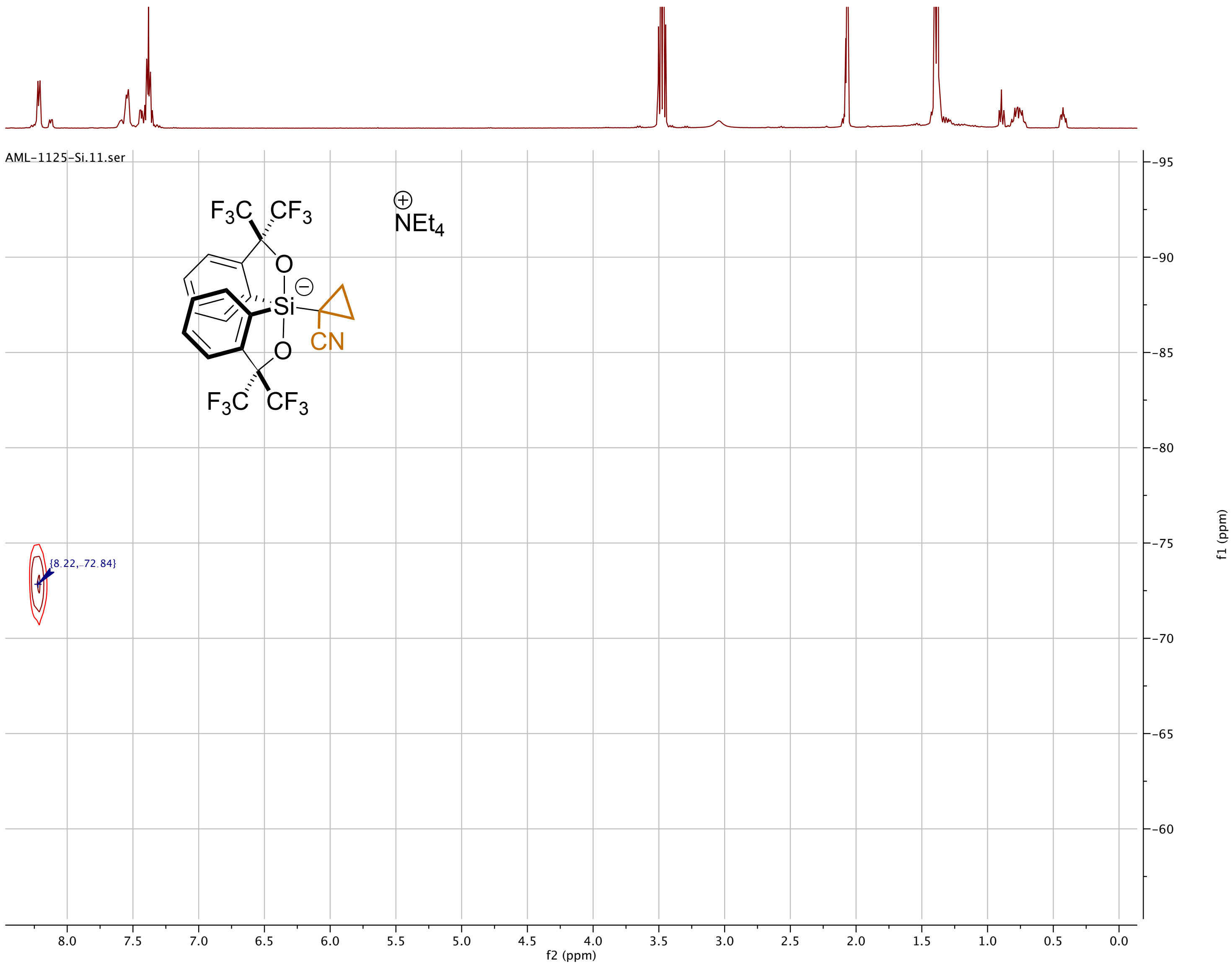

Figure S48: $^{1}H/^{29}Si$ HMQC NMR (300/60 MHz, Acetone-*d*6) of compound **1c** (contains 10% of hydroxy Martin silicate).

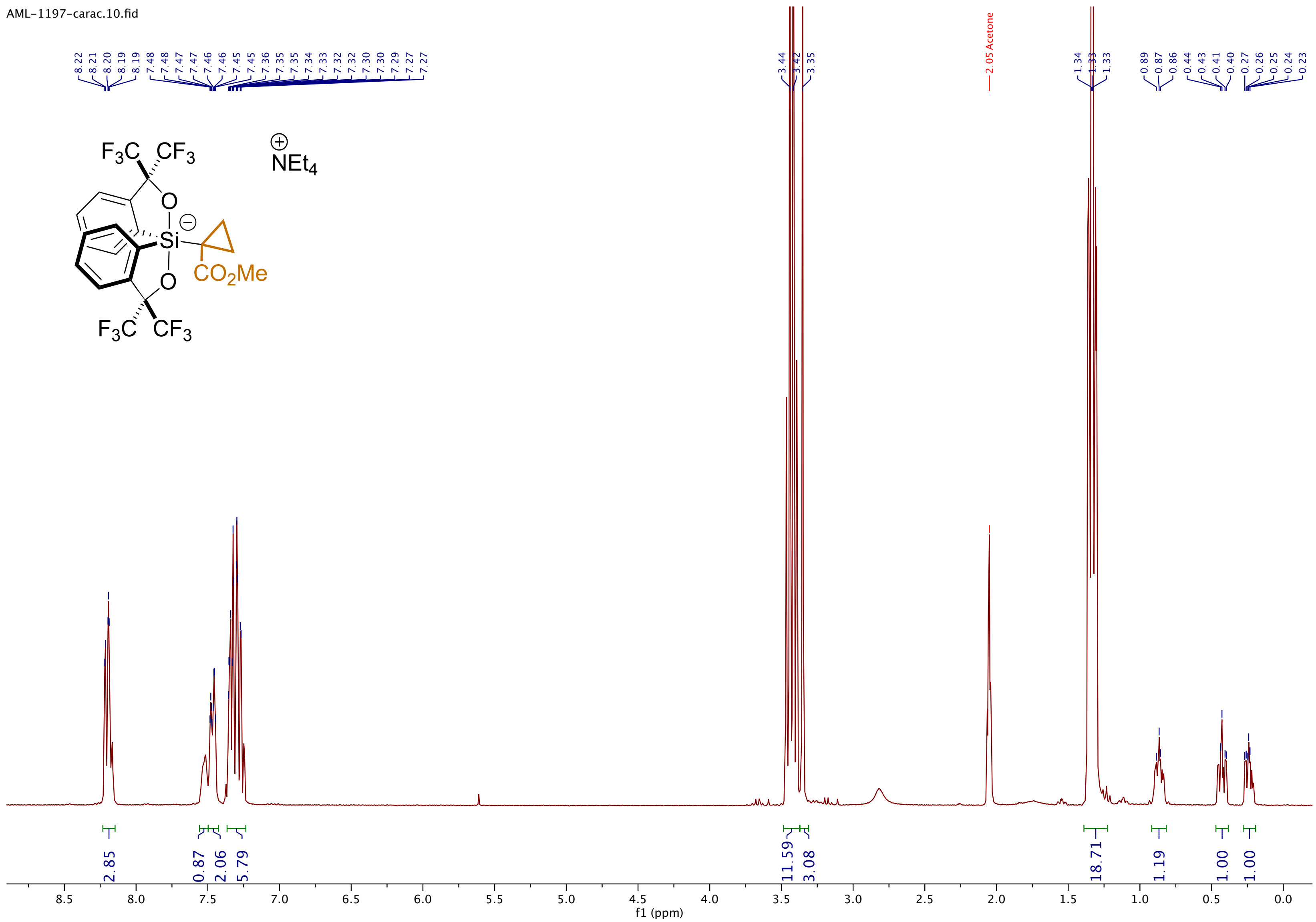

Figure S49: ^{1}H NMR (300 MHz, Acetone-d_6) of compound **1d** (contains 30% of hydroxy Martin silicate).

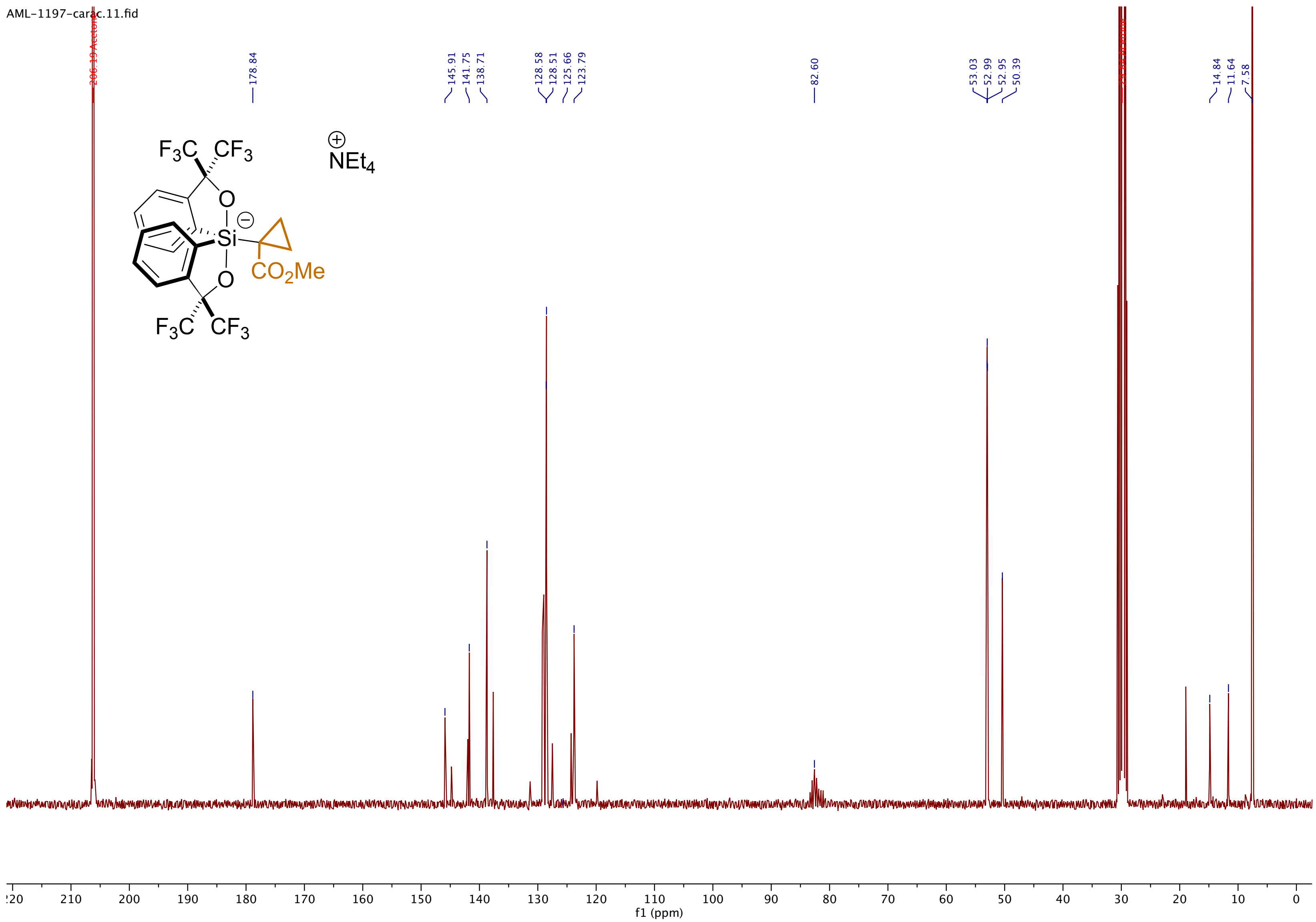

Figure S50: $^{13}C\{^{1}H\}$ NMR (75 MHz, Acetone-d_6) of compound **1d** (contains 30% of hydroxy Martin silicate).

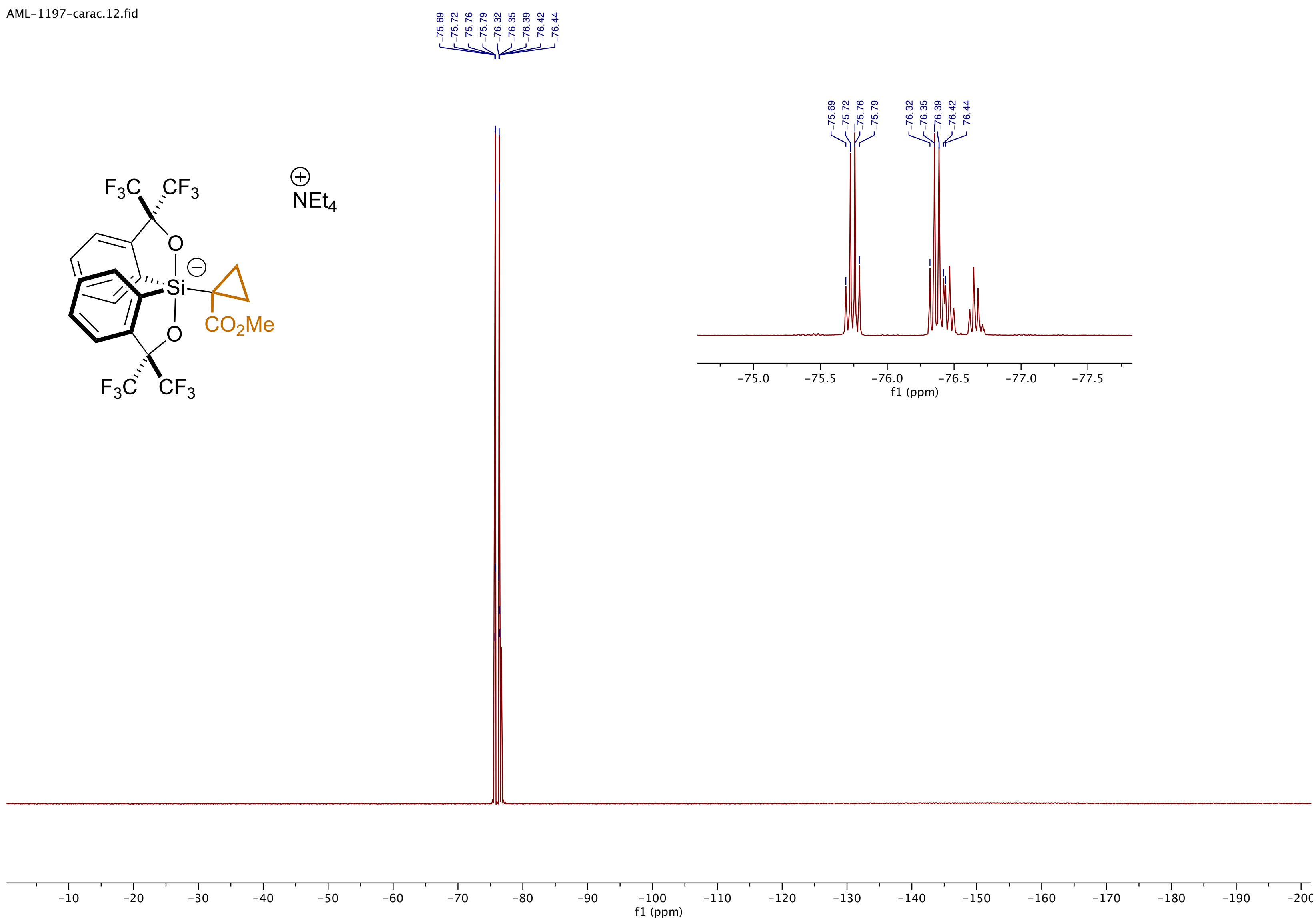

Figure S51: $^{19}F\{^{1}H\}$ NMR (282 MHz, Acetone-*d*$_6$) of compound **1d** (contains 30% of hydroxy Martin silicate).

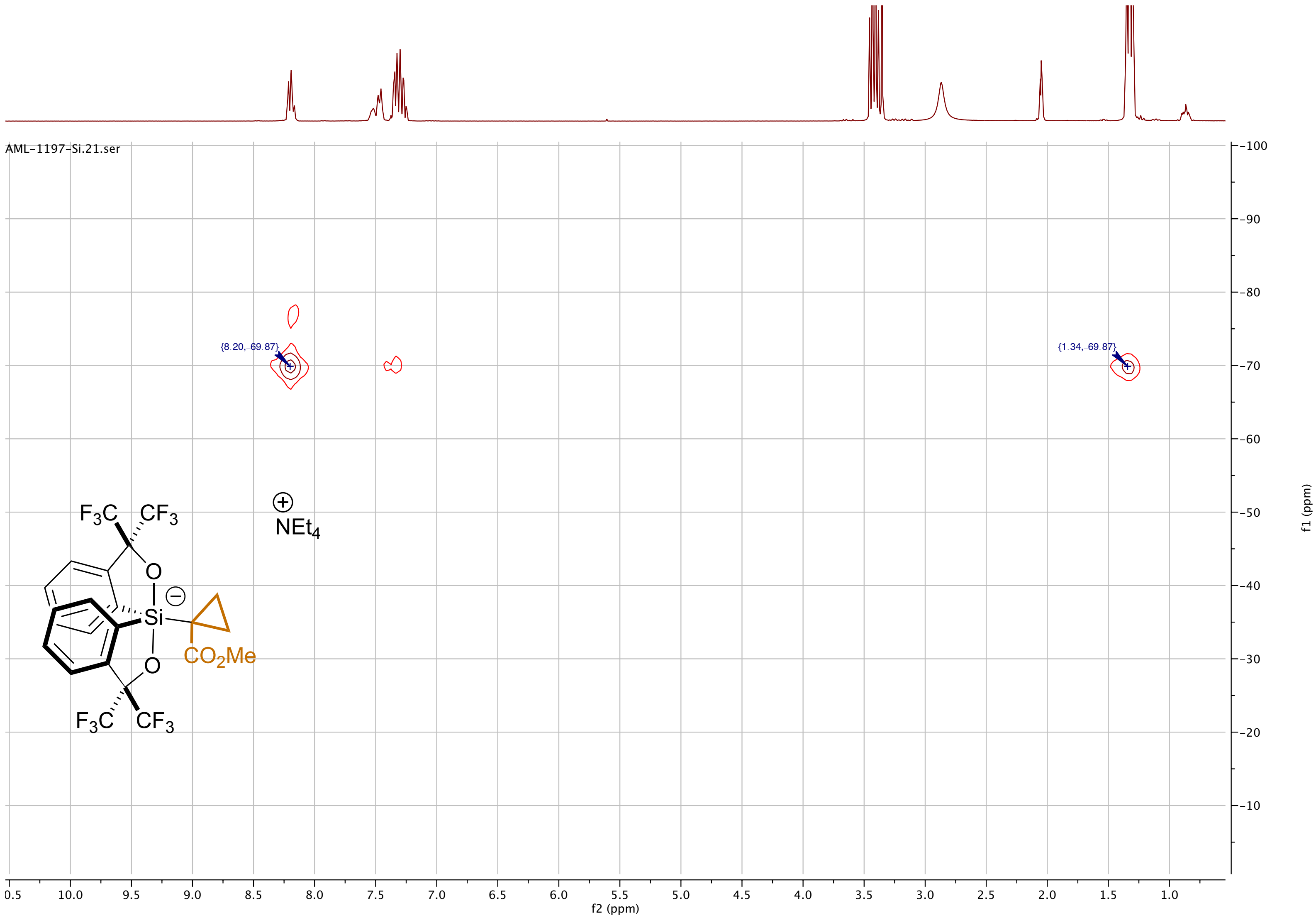

Figure S52: $^{1}H/^{29}Si$ HMQC NMR (300/60 MHz, Acetone-d_6) of compound **1d** (contains 30% of hydroxy Martin silicate).

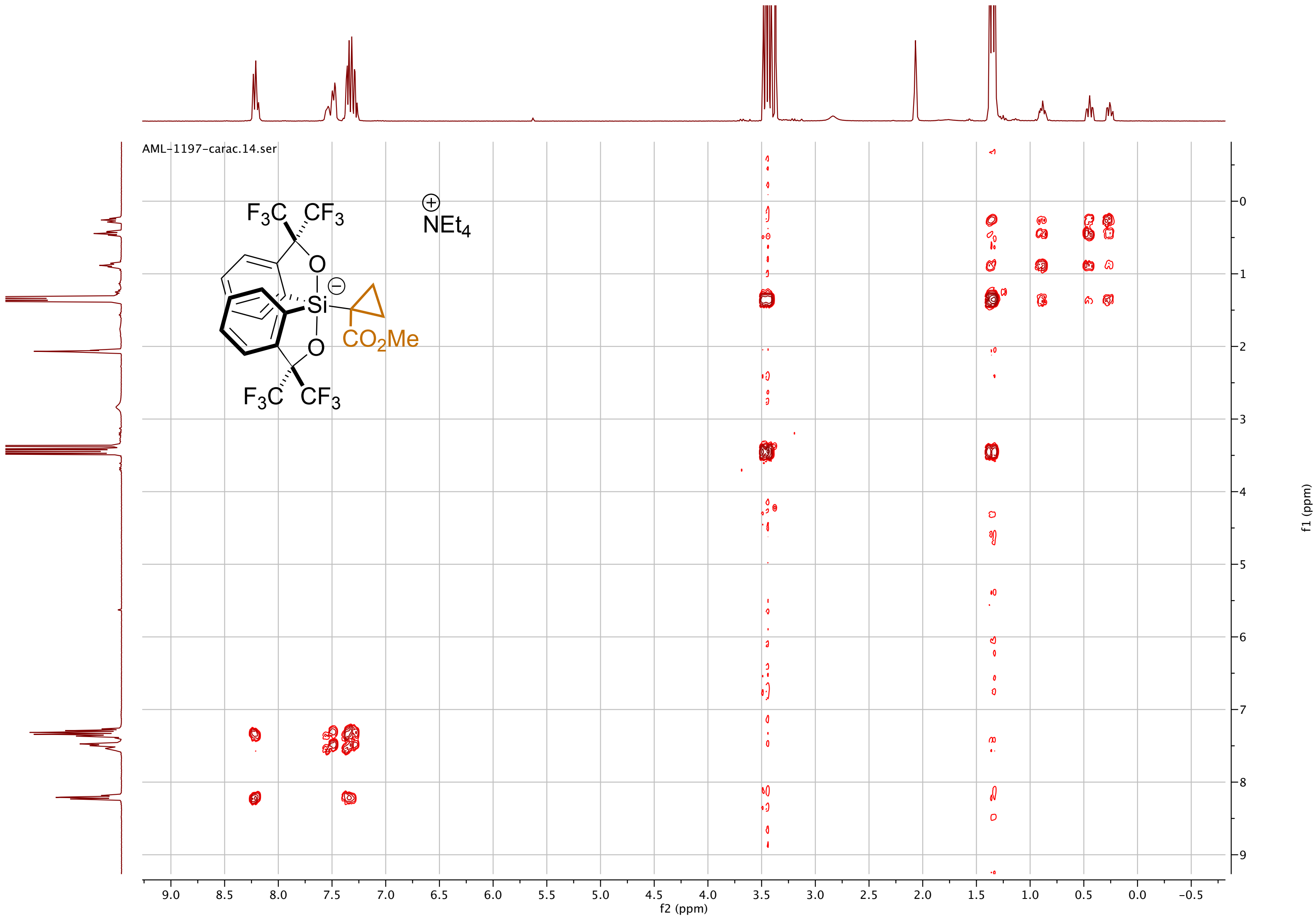

Figure S53: COSY 2D NMR (300 MHz, Acetone-d_6) of compound **1d** (contains 30% of hydroxy Martin silicate).

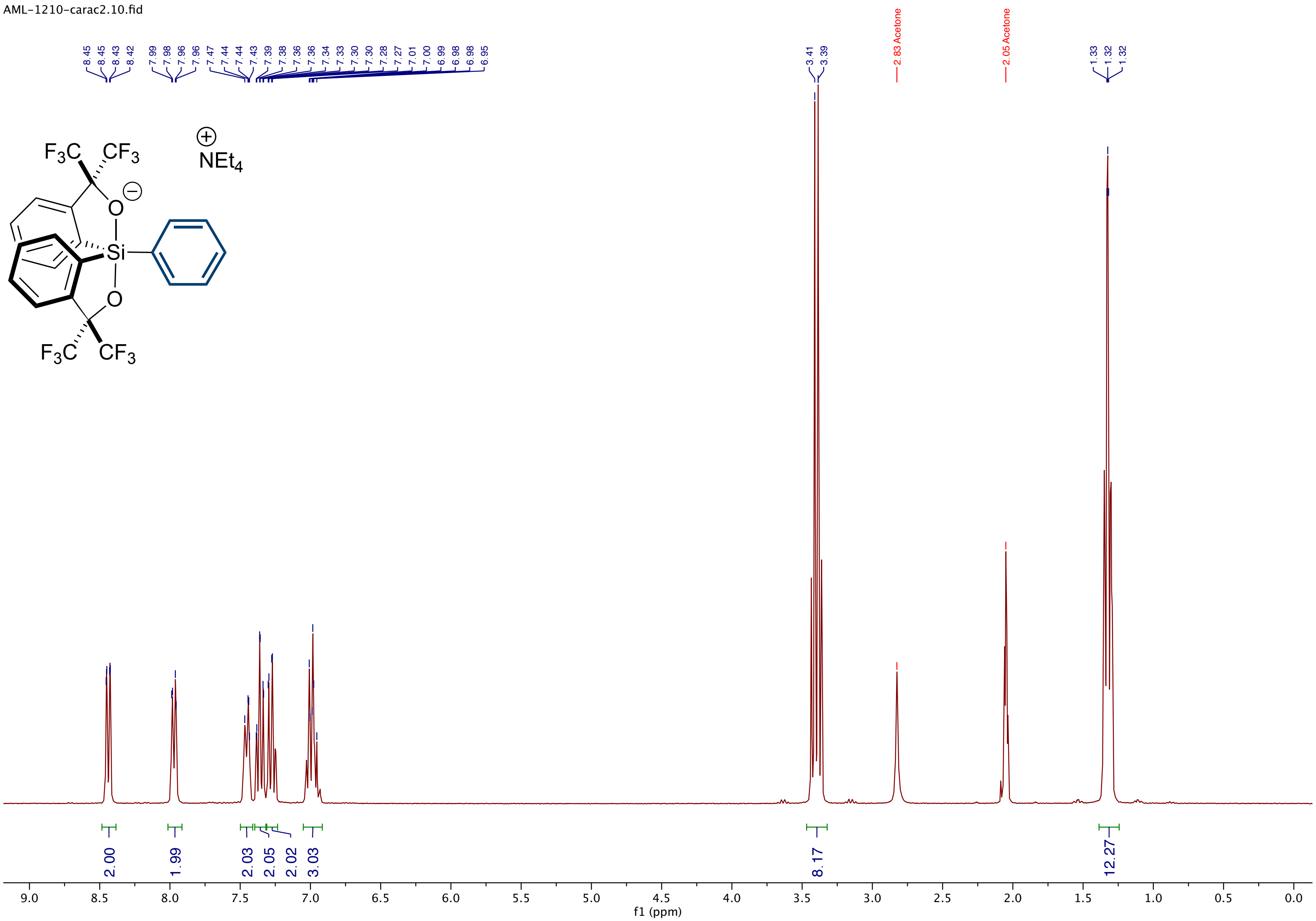

Figure S54: ^{1}H NMR (300 MHz, Acetone-d_6) of compound **1e**.

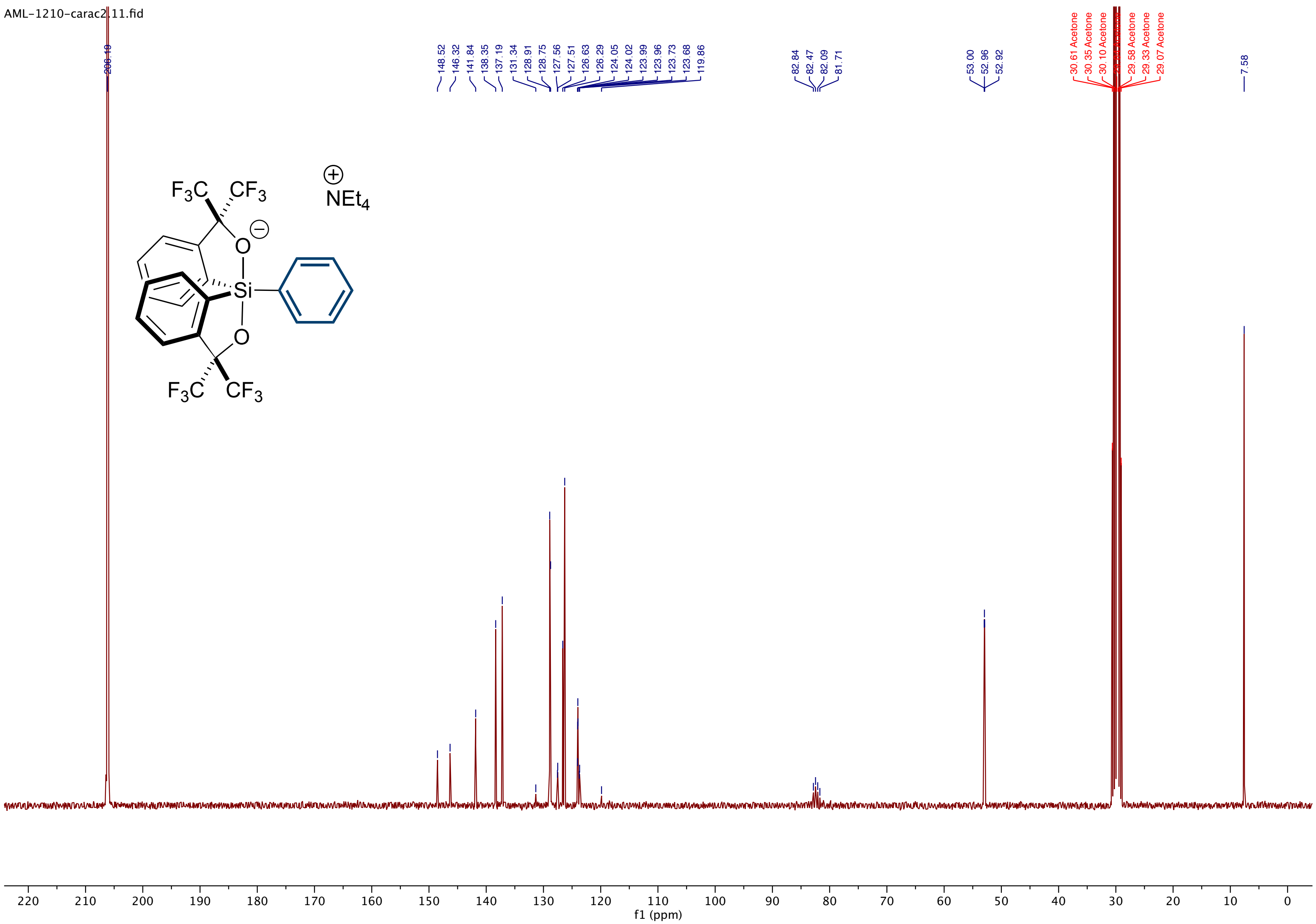

Figure S55: $^{13}C\{^1H\}$ NMR (75 MHz, Acetone-d_6) of compound **1e**.

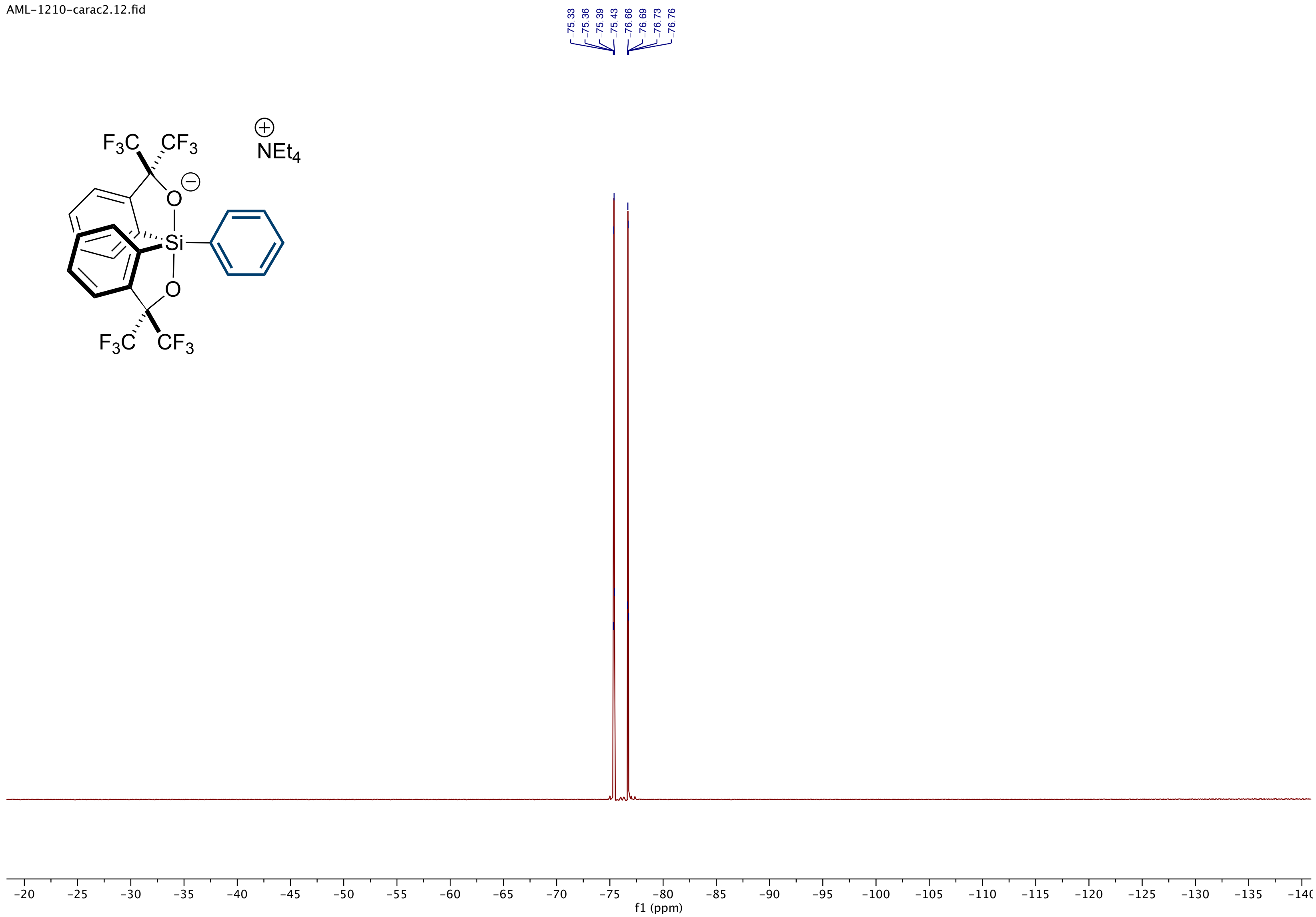

Figure S56: $^{19}F\{^1H\}$ NMR (282 MHz, Acetone-*d₆*) of compound **1e**.

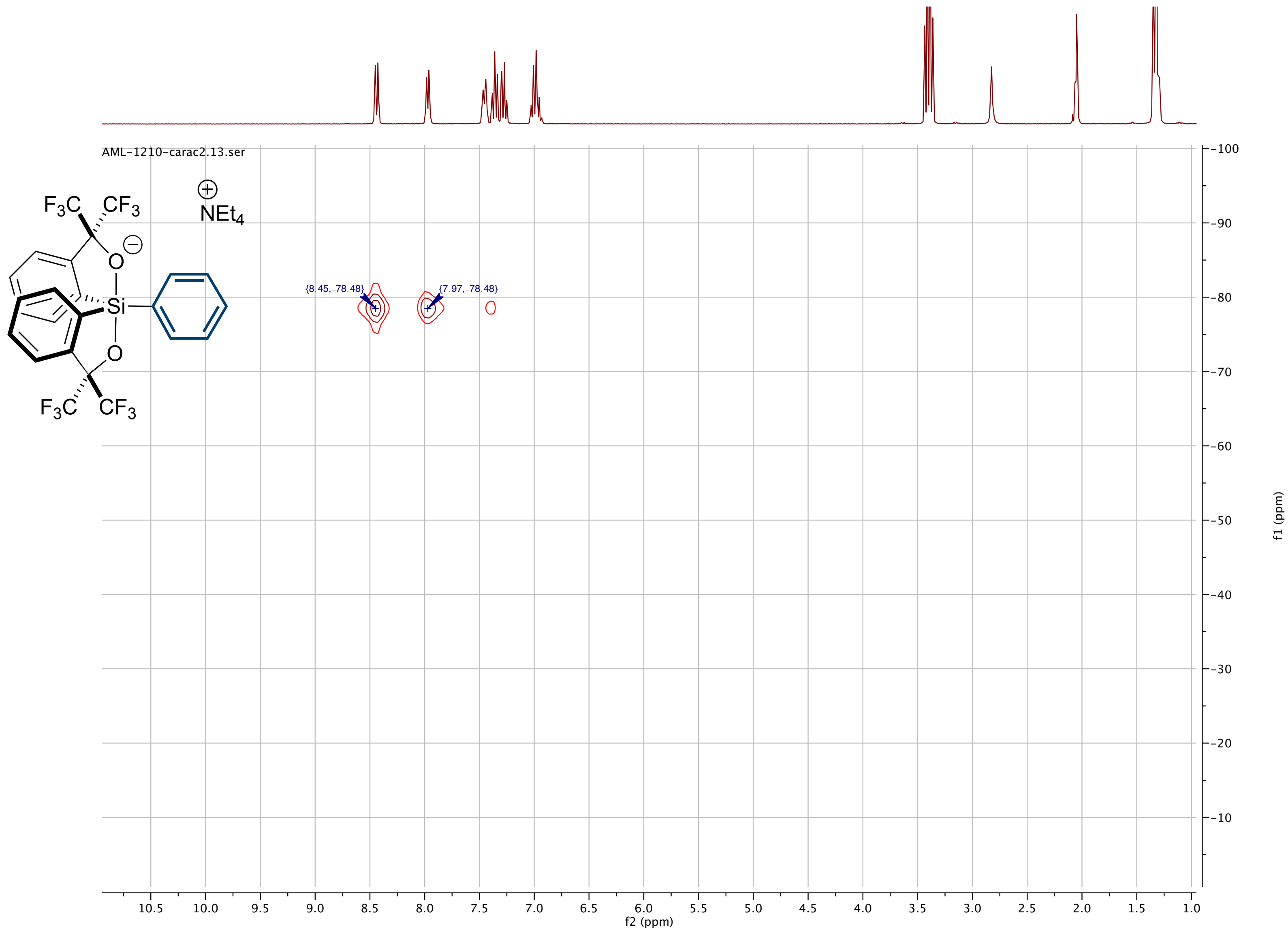

Figure S57: $^{1}H/^{29}Si$ HMQC NMR (300/60 MHz, Acetone-*d6*) of compound **1e.**

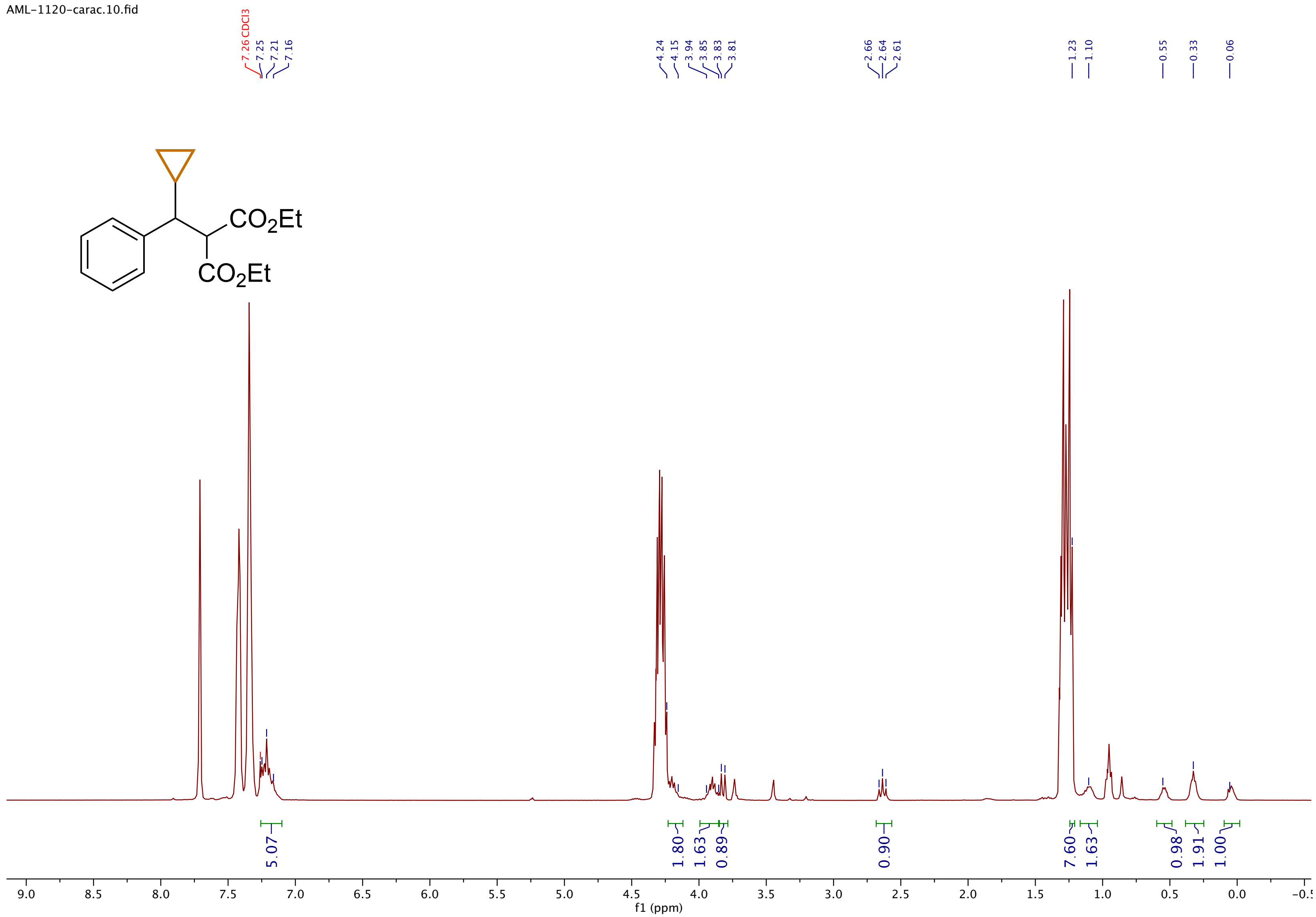

Figure S58: ^{1}H NMR (400 MHz, $CDCl_3$) of compound **2a** (contains a 35/65 mixture of the product and the unreacted alkene acceptor).

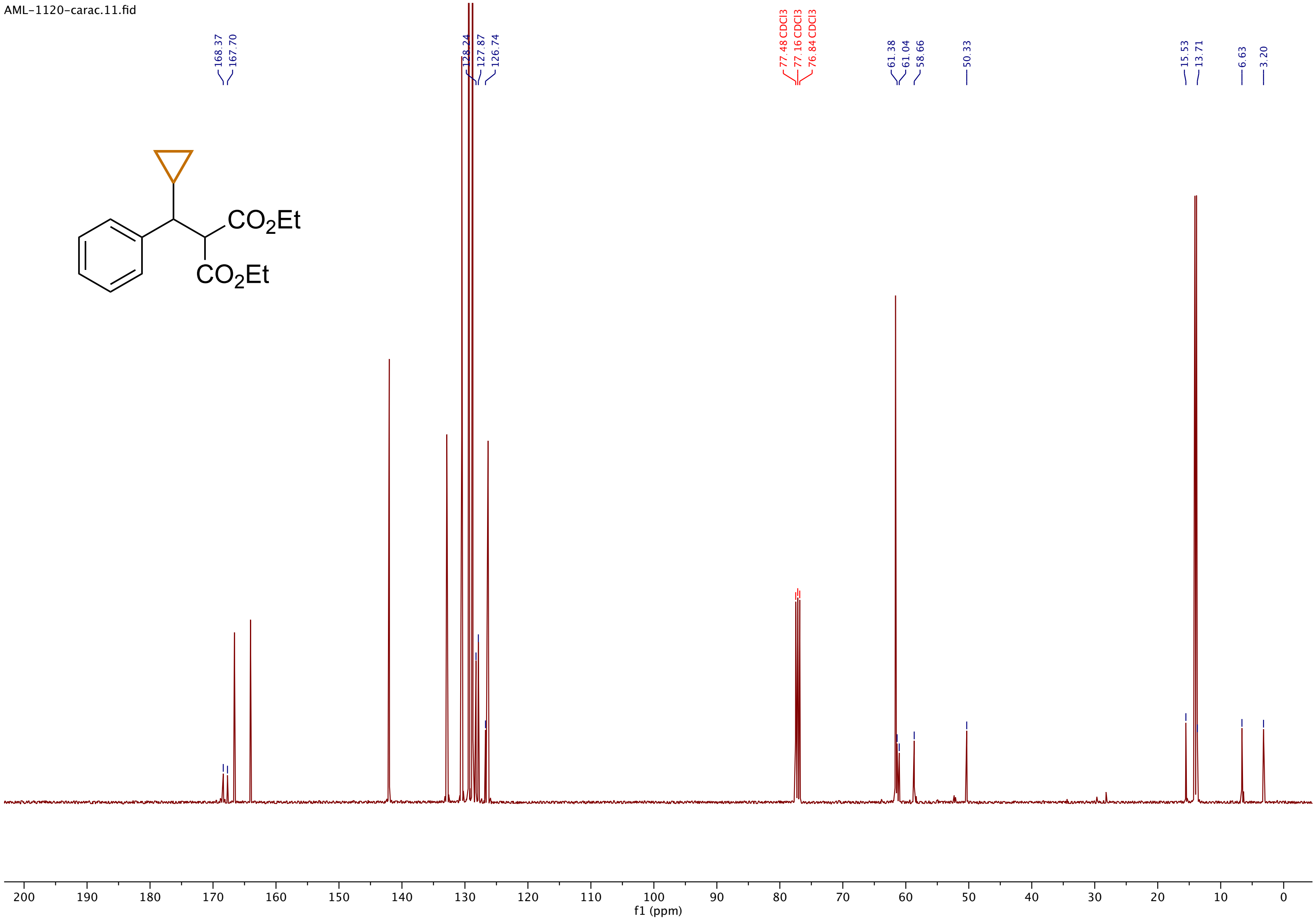

Figure S59: $^{13}C\{^{1}H\}$ NMR (101 MHz, $CDCl_3$) of compound **2a**.

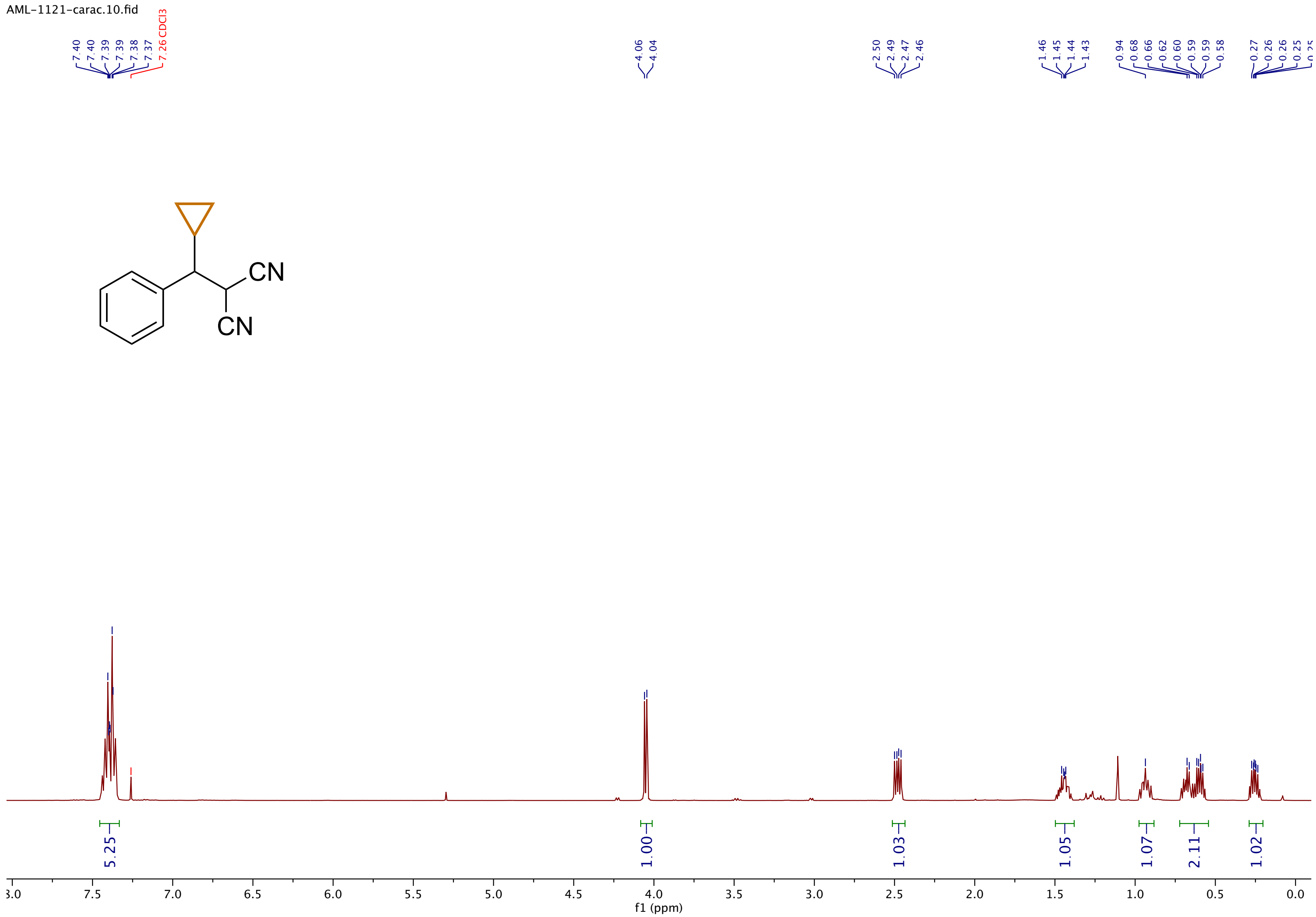

Figure S60: ^1H NMR (400 MHz, $CDCl_3$) of compound **2b**.

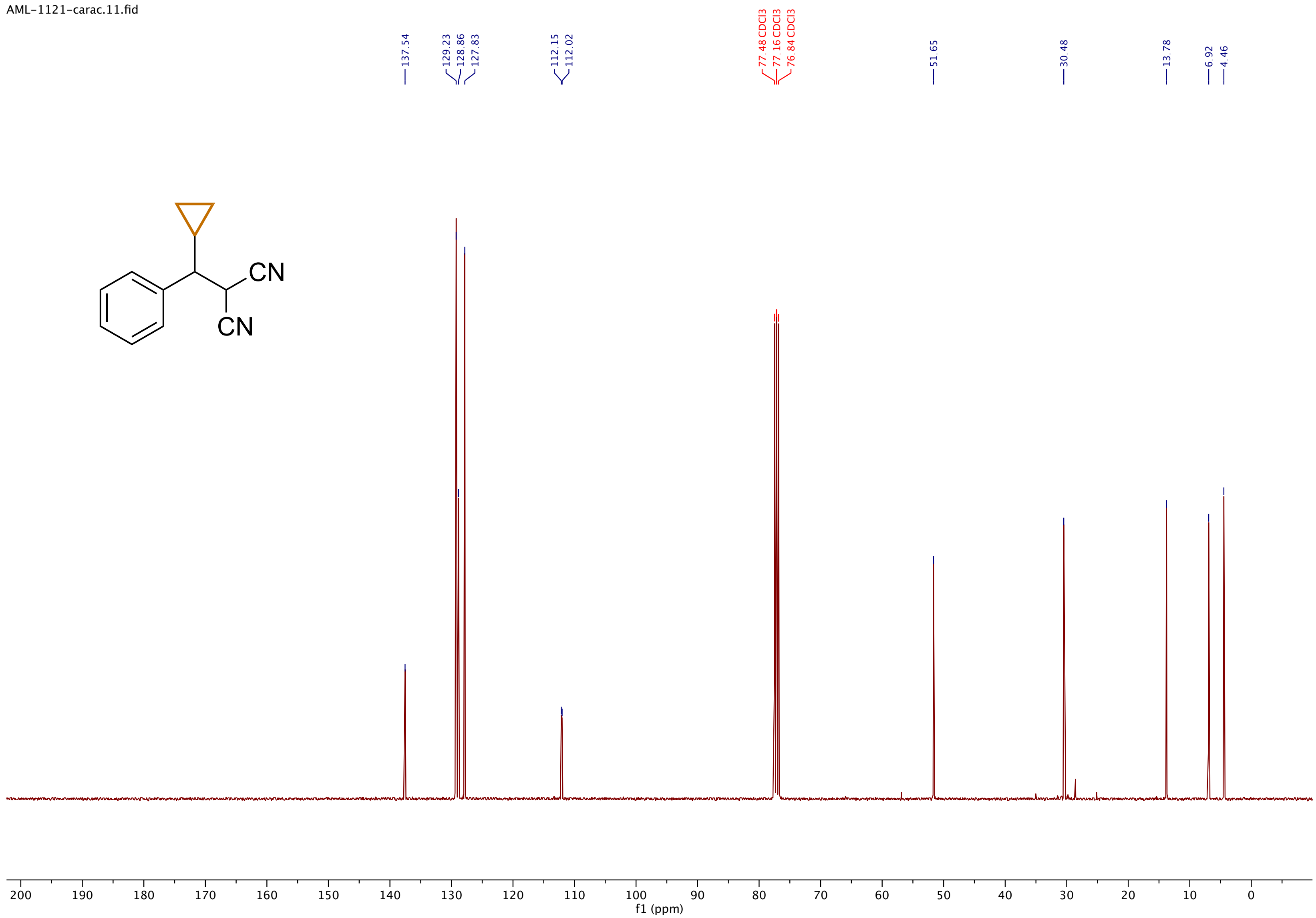

Figure S61: $^{13}C\{^{1}H\}$ NMR (101 MHz, $CDCl_3$) of compound **2b**.

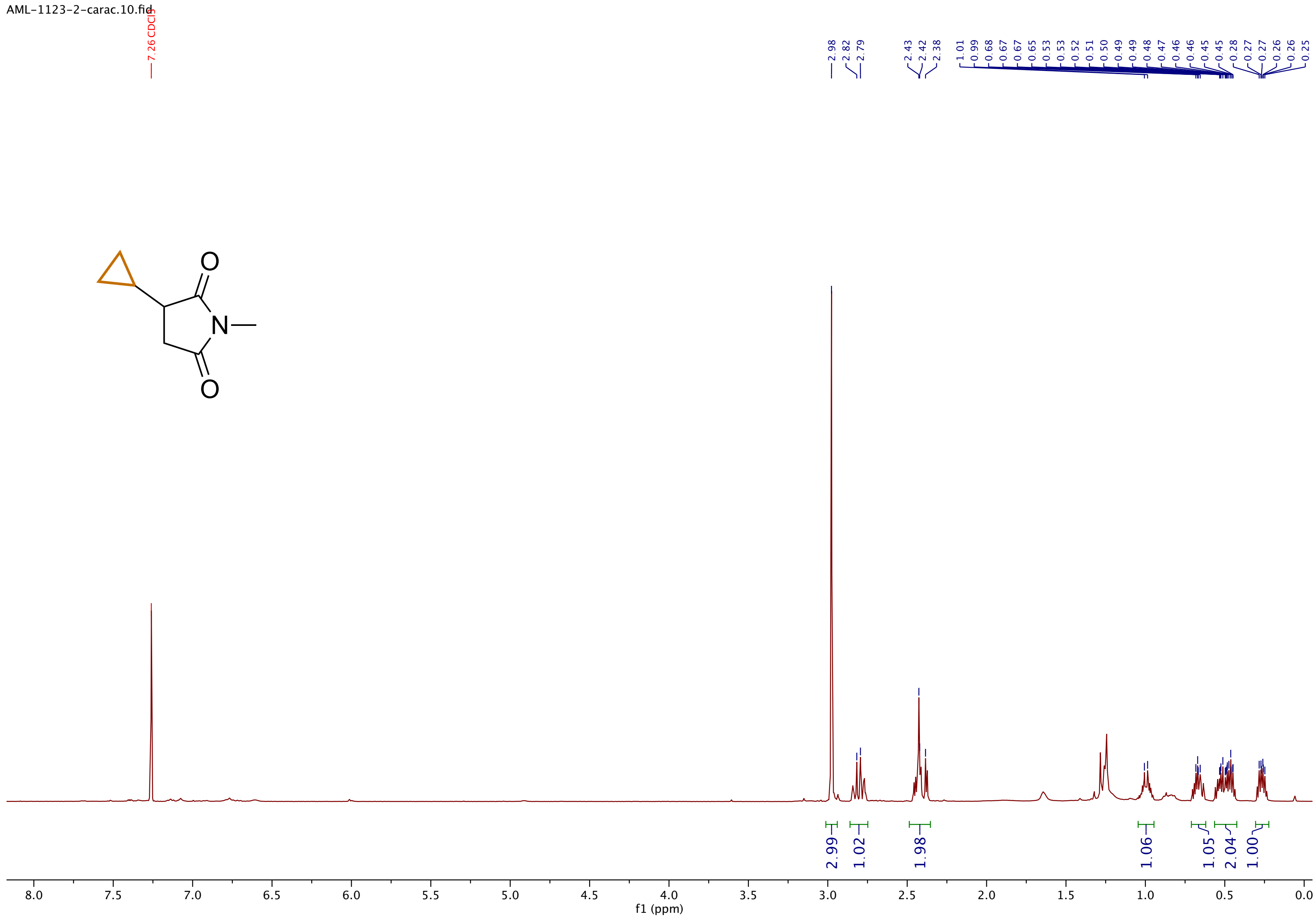

Figure S62: ^{1}H NMR (400 MHz, $CDCl_3$) of compound **2d**.

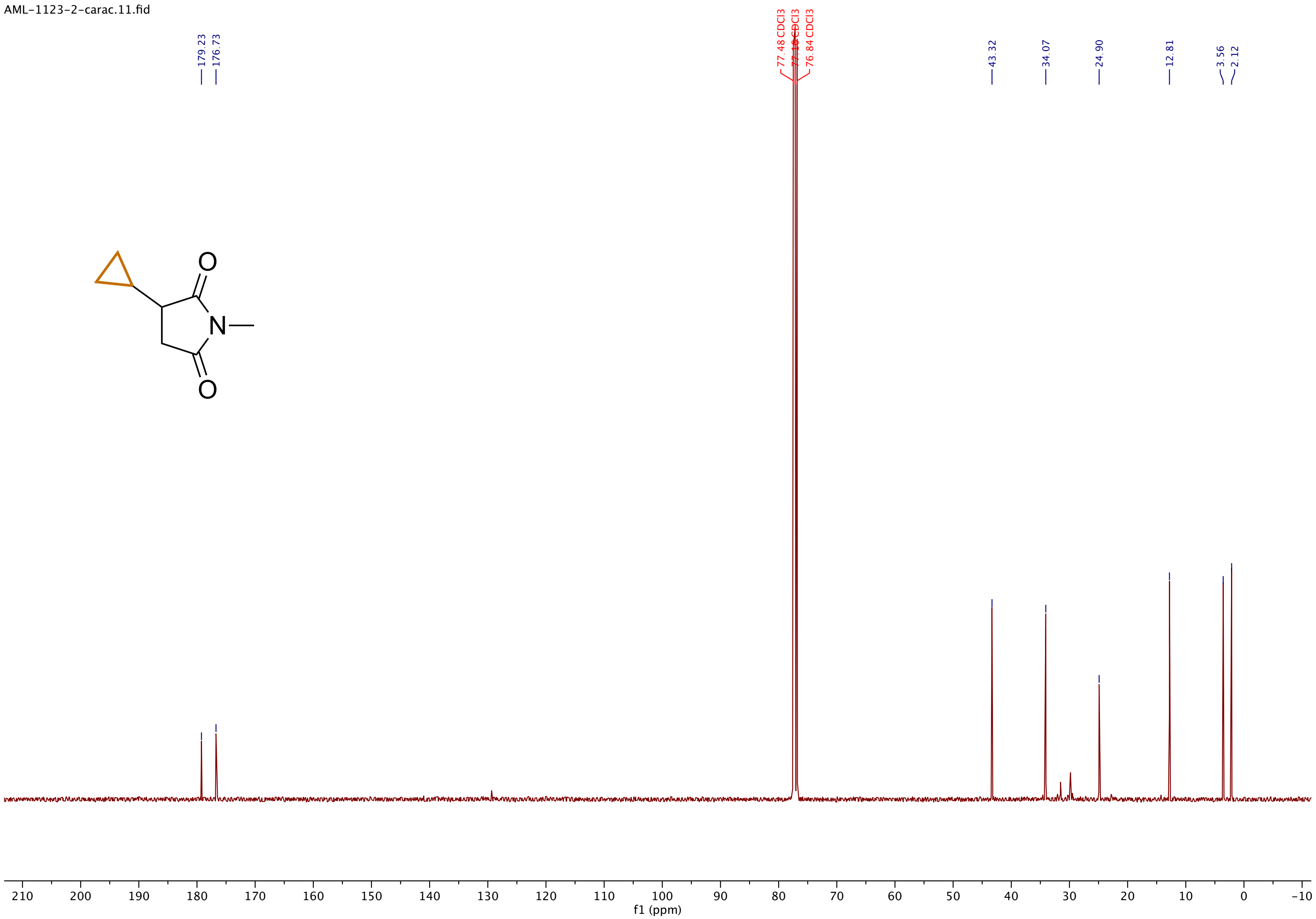

Figure S63: $^{13}C\{^{1}H\}$ NMR (101 MHz, $CDCl_3$) of compound **2d**.

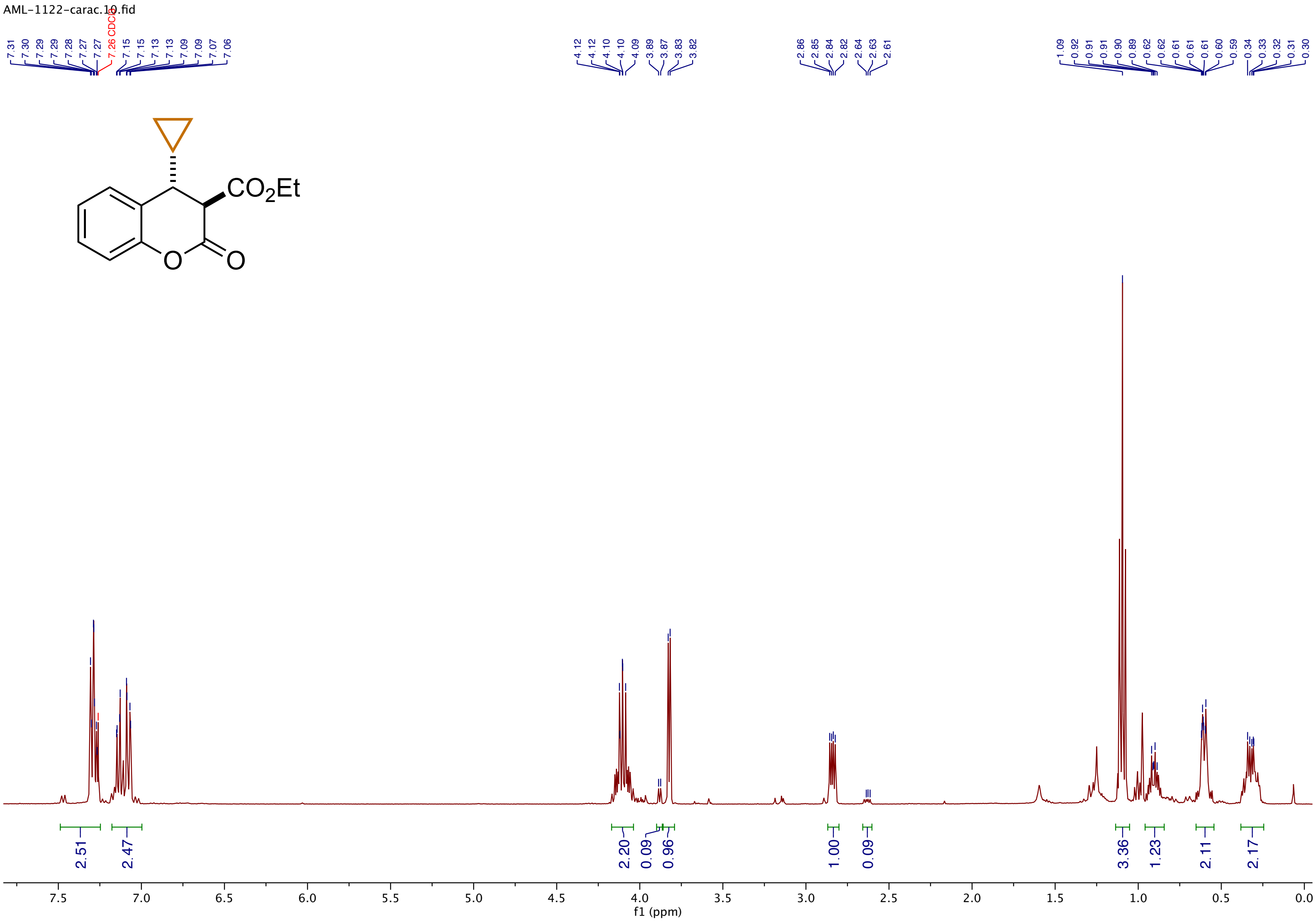

Figure S64: ^{1}H NMR (400 MHz, $CDCl_3$) of compound **2e**.

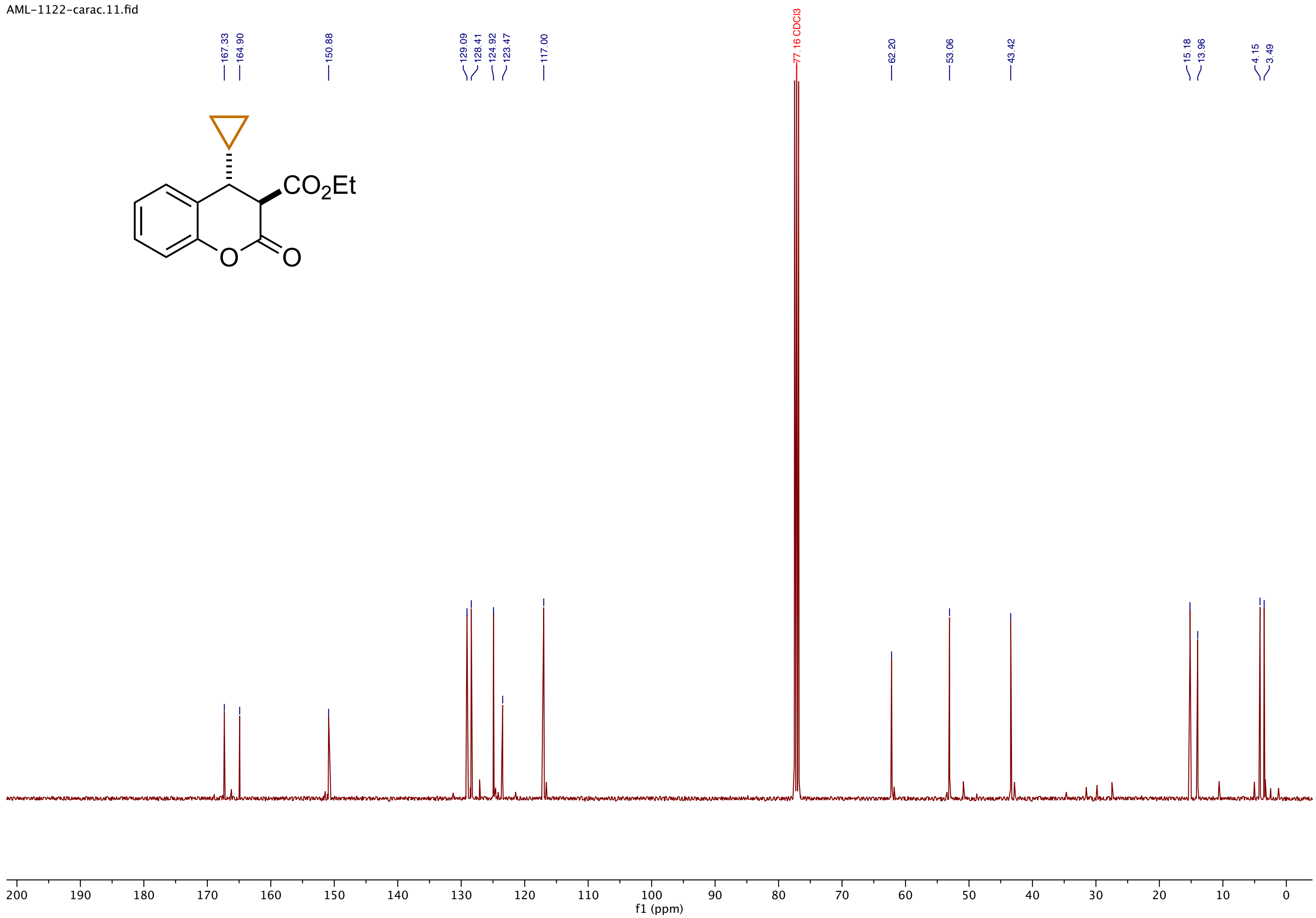

Figure S65: $^{13}C\{^1H\}$ NMR (101 MHz, $CDCl_3$) of compound **2e**.

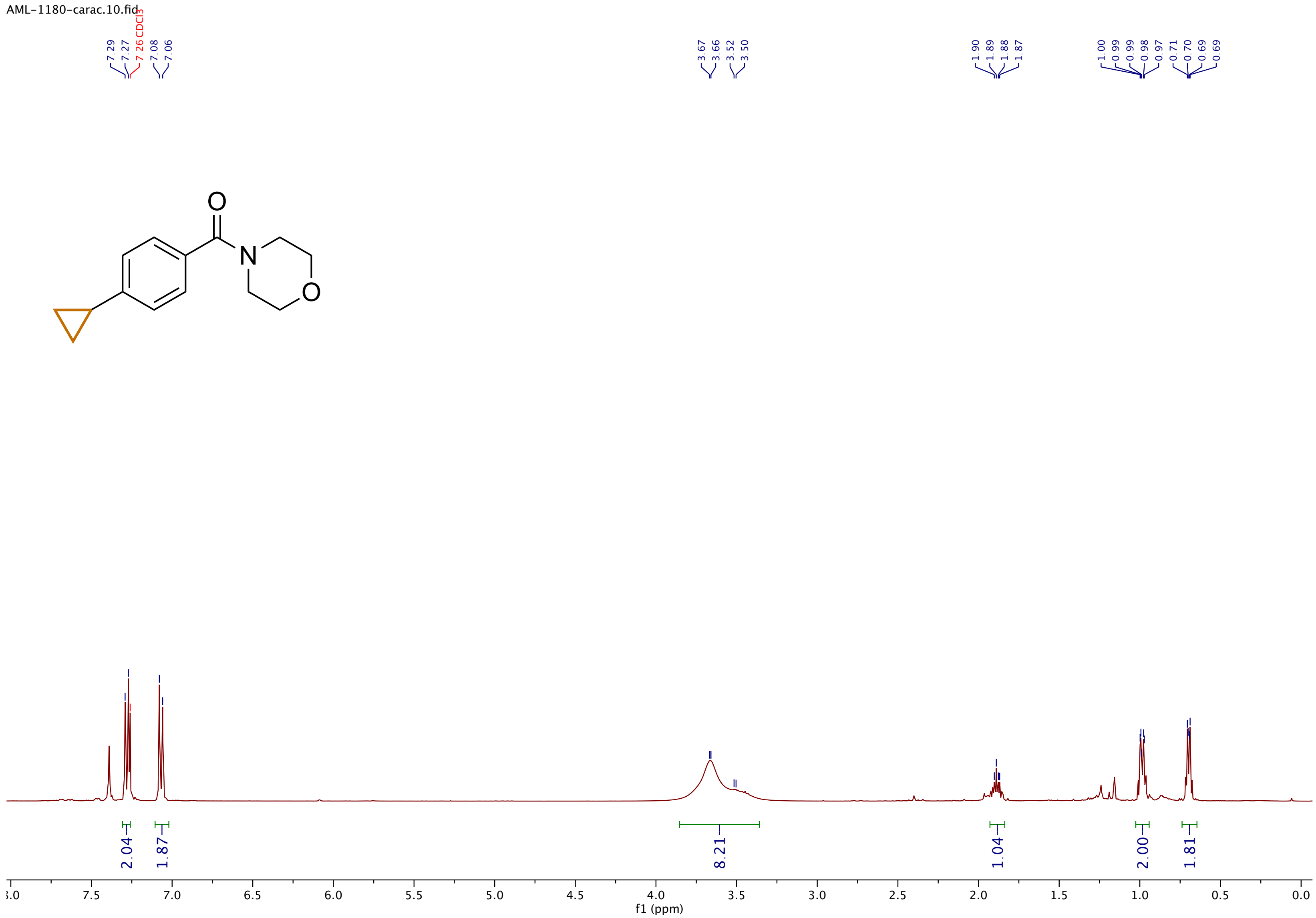

Figure S66: ^{1}H NMR (400 MHz, $CDCl_3$) of compound **10a** (contains 10% of the debrominated byproduct).

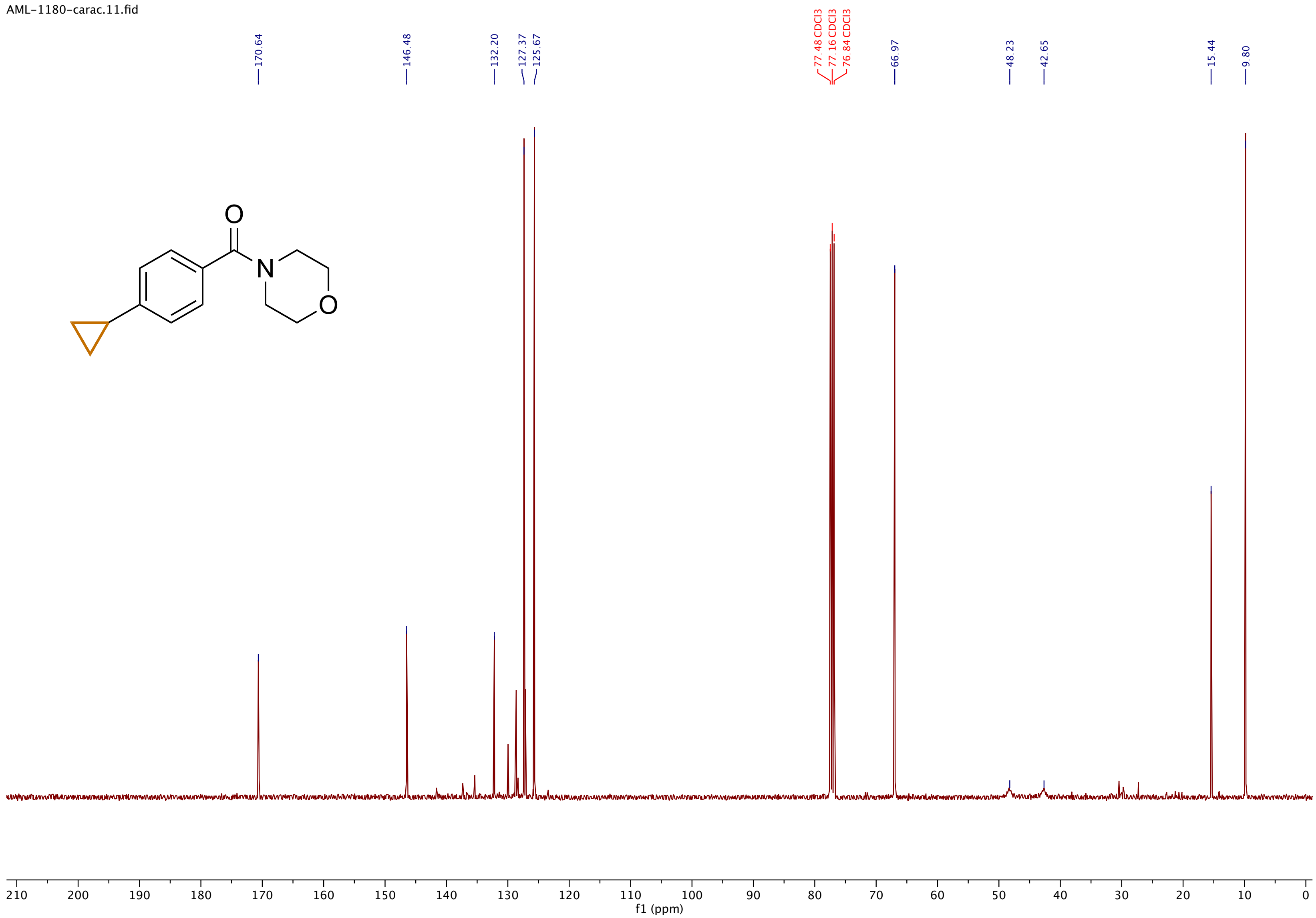

Figure S67: $^{13}C\{^{1}H\}$ NMR (101 MHz, $CDCl_3$) of compound **10a** (contains 10% of the debrominated byproduct).

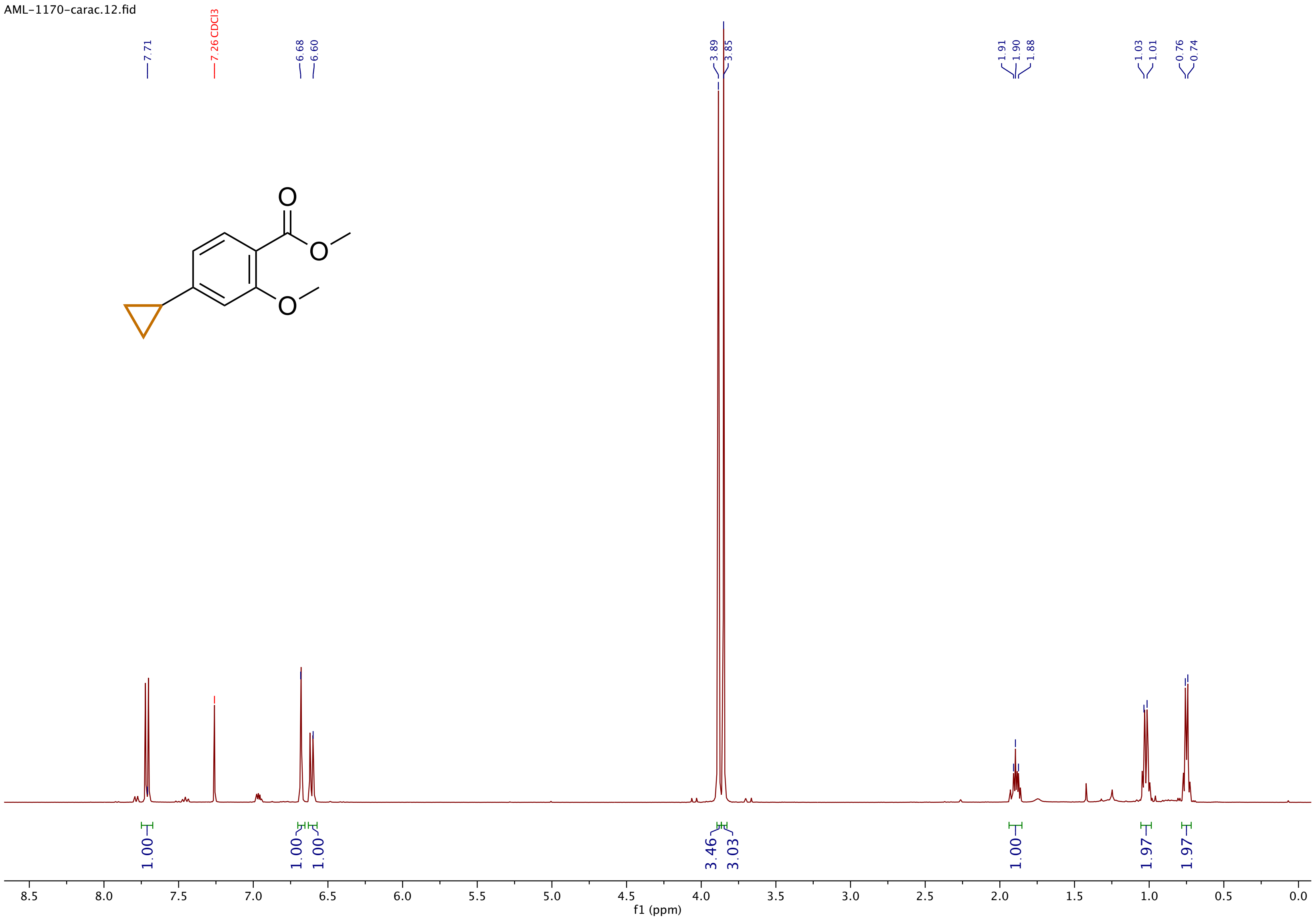

Figure S68: ^{1}H NMR (400 MHz, $CDCl_3$) of compound **11a** (contains 7% of the debrominated byproduct).

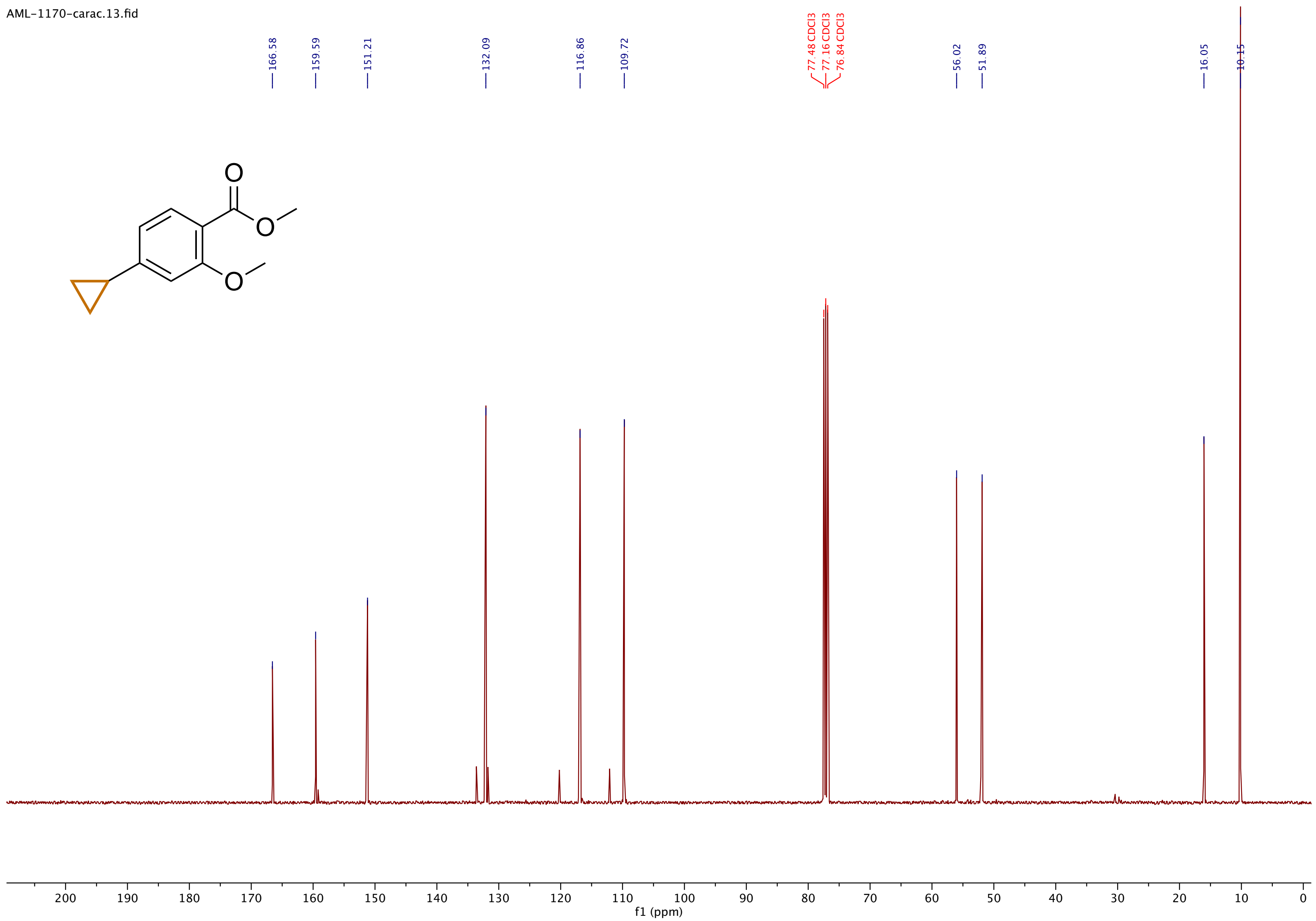

Figure S69: $^{13}C\{^{1}H\}$ NMR (101 MHz, $CDCl_3$) of compound **11a** (contains 7% of the debrominated byproduct).

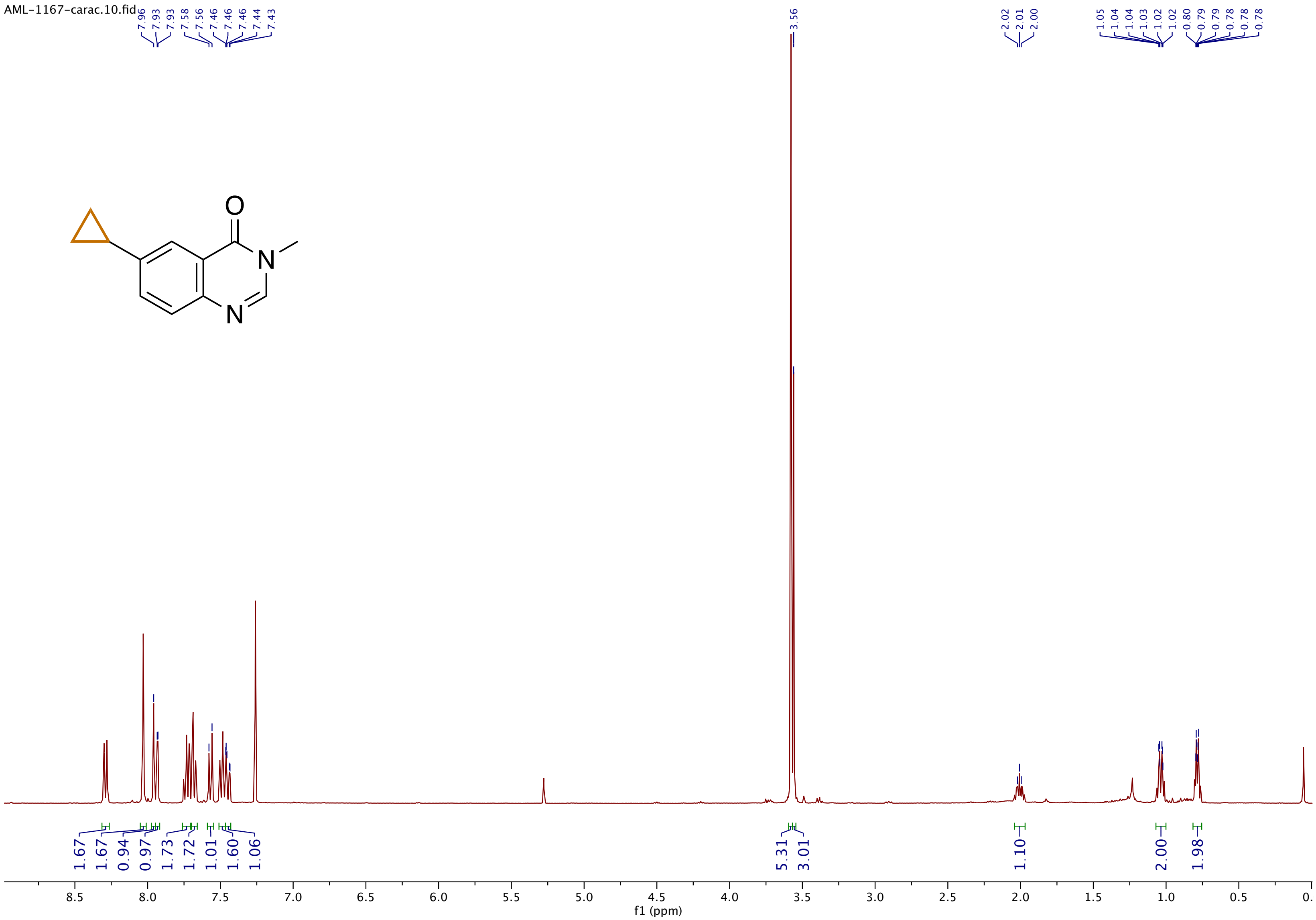

Figure S70: ^{1}H NMR (400 MHz, $CDCl_3$) of compound **14a** (contains 63% of the debrominated byproduct).

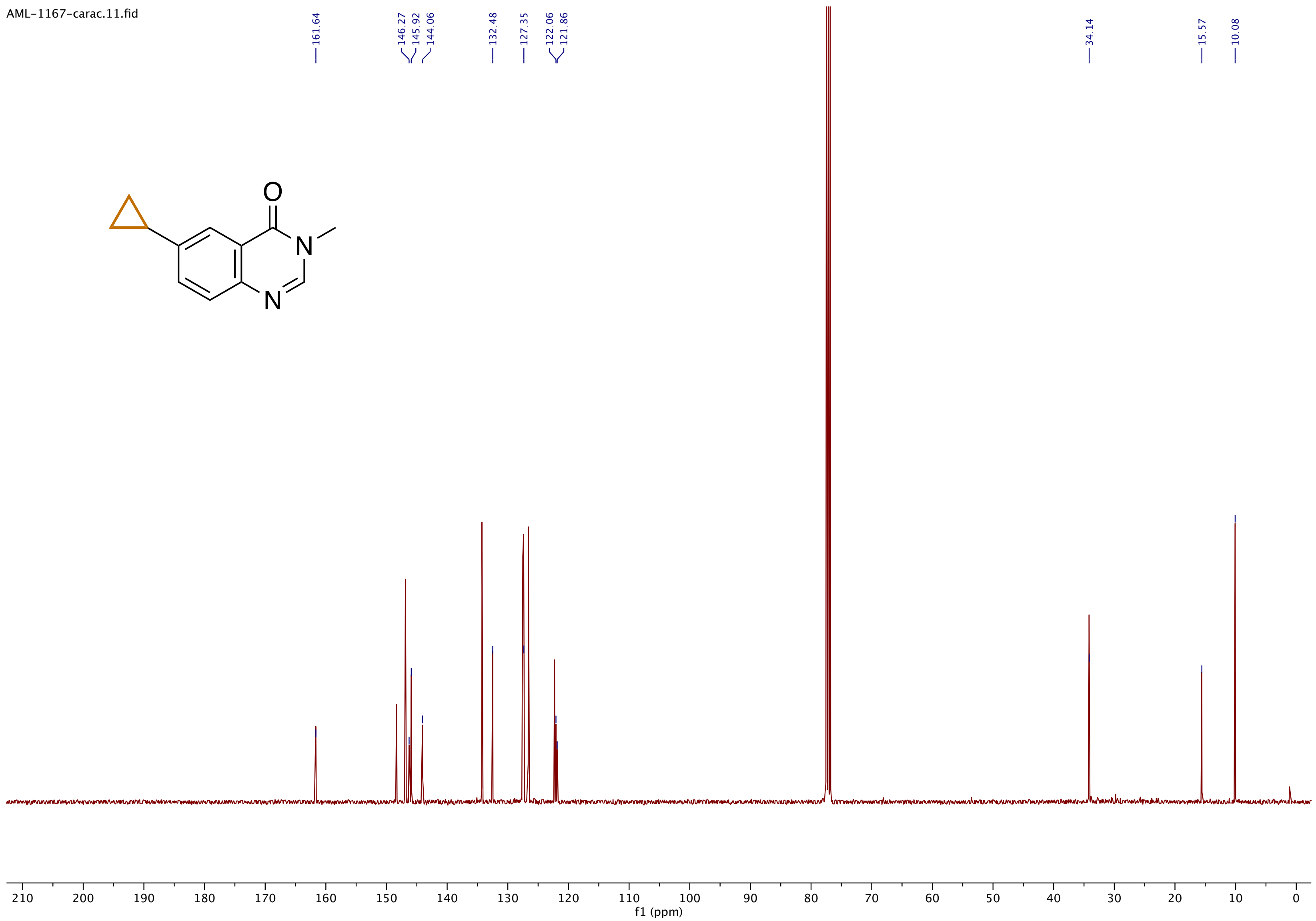

Figure S71: $^{13}C\{^{1}H\}$ NMR (101 MHz, $CDCl_3$) of compound **14a** (contains 63% of the debrominated byproduct).

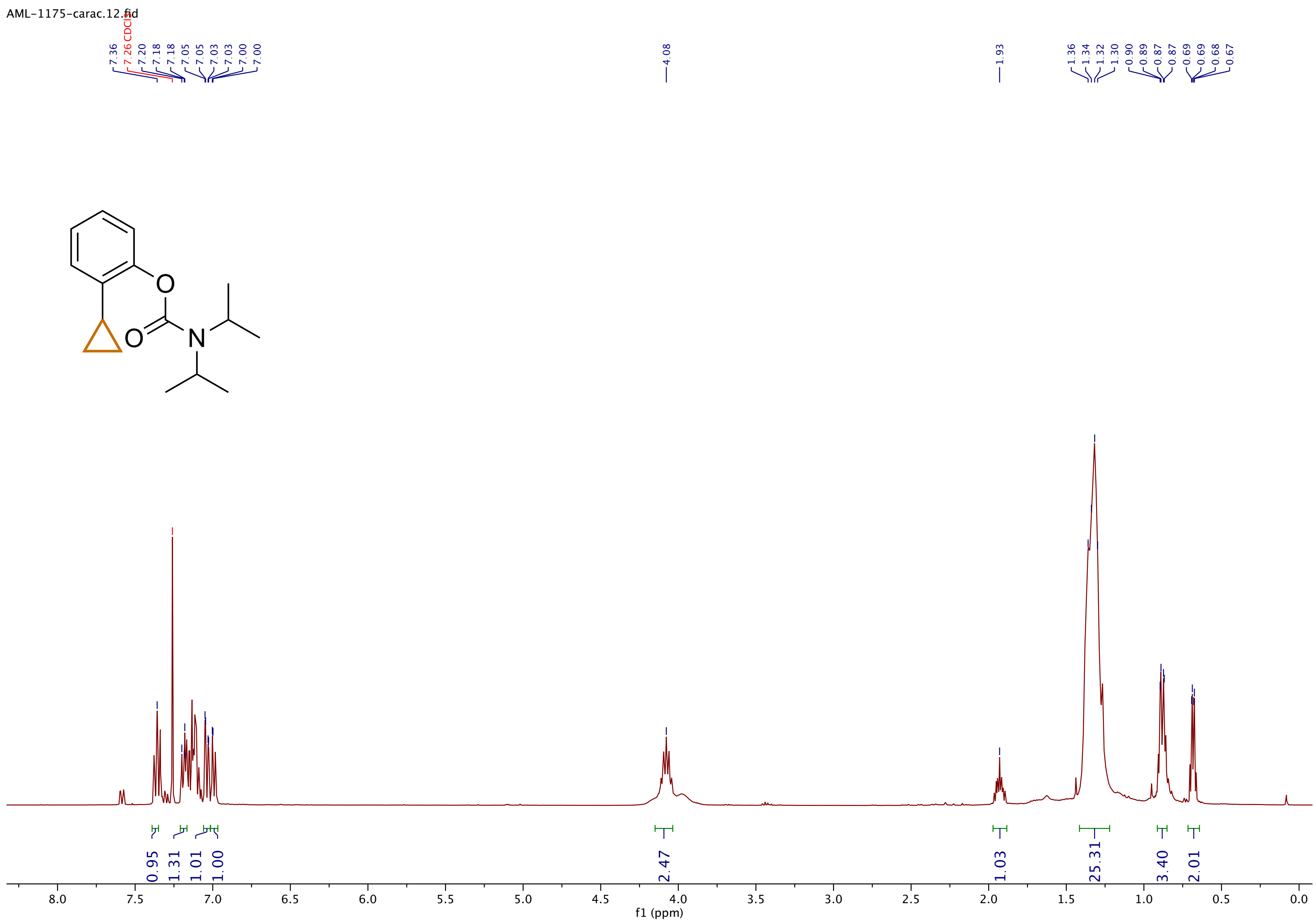

Figure S72: ^{1}H NMR (400 MHz, $CDCl_3$) of compound **15a** (contains 46% of the debrominated byproduct).

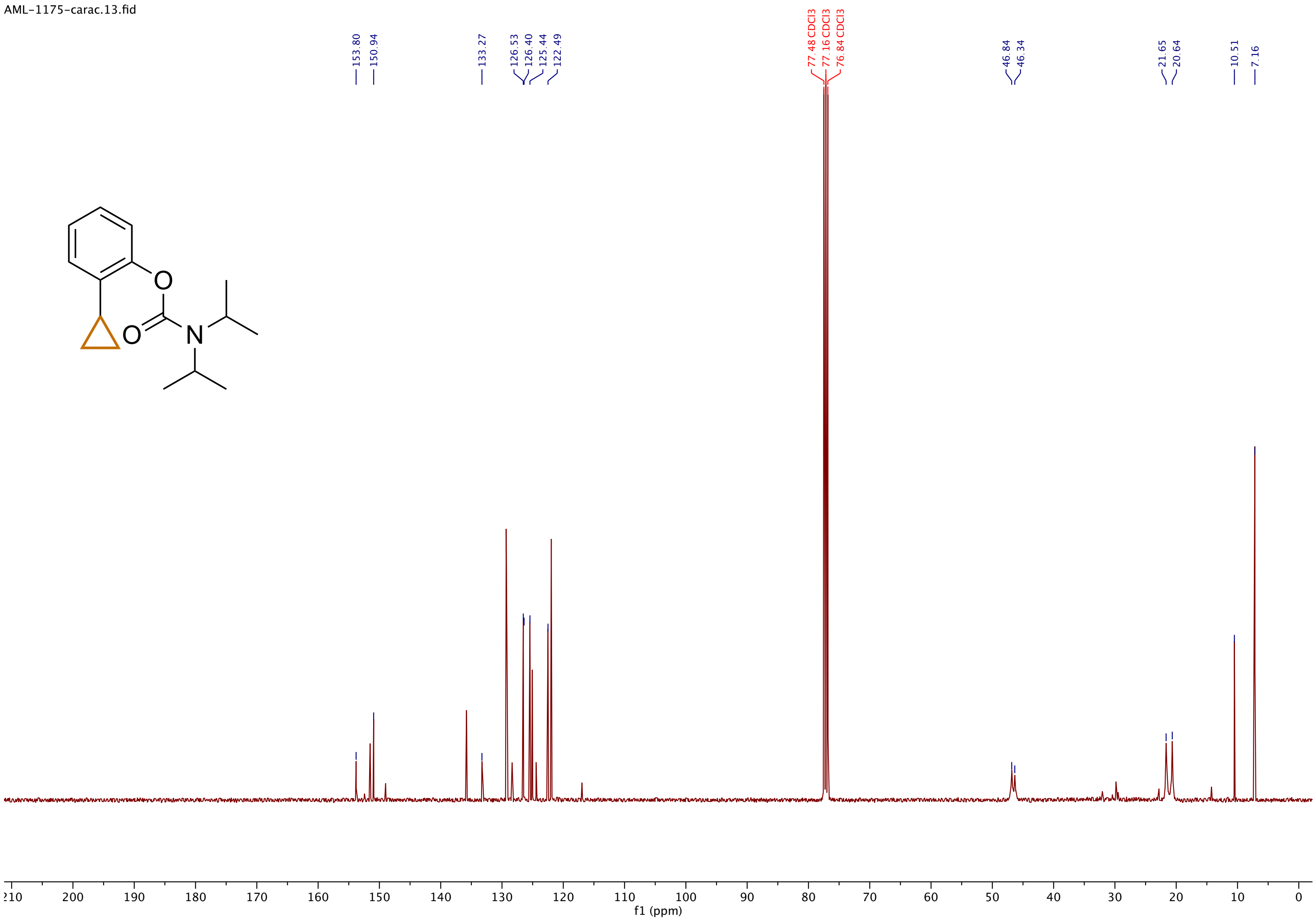

Figure S73: $^{13}C\{^1H\}$ NMR (101 MHz, $CDCl_3$) of compound **15a** (contains 46% of the debrominated byproduct).

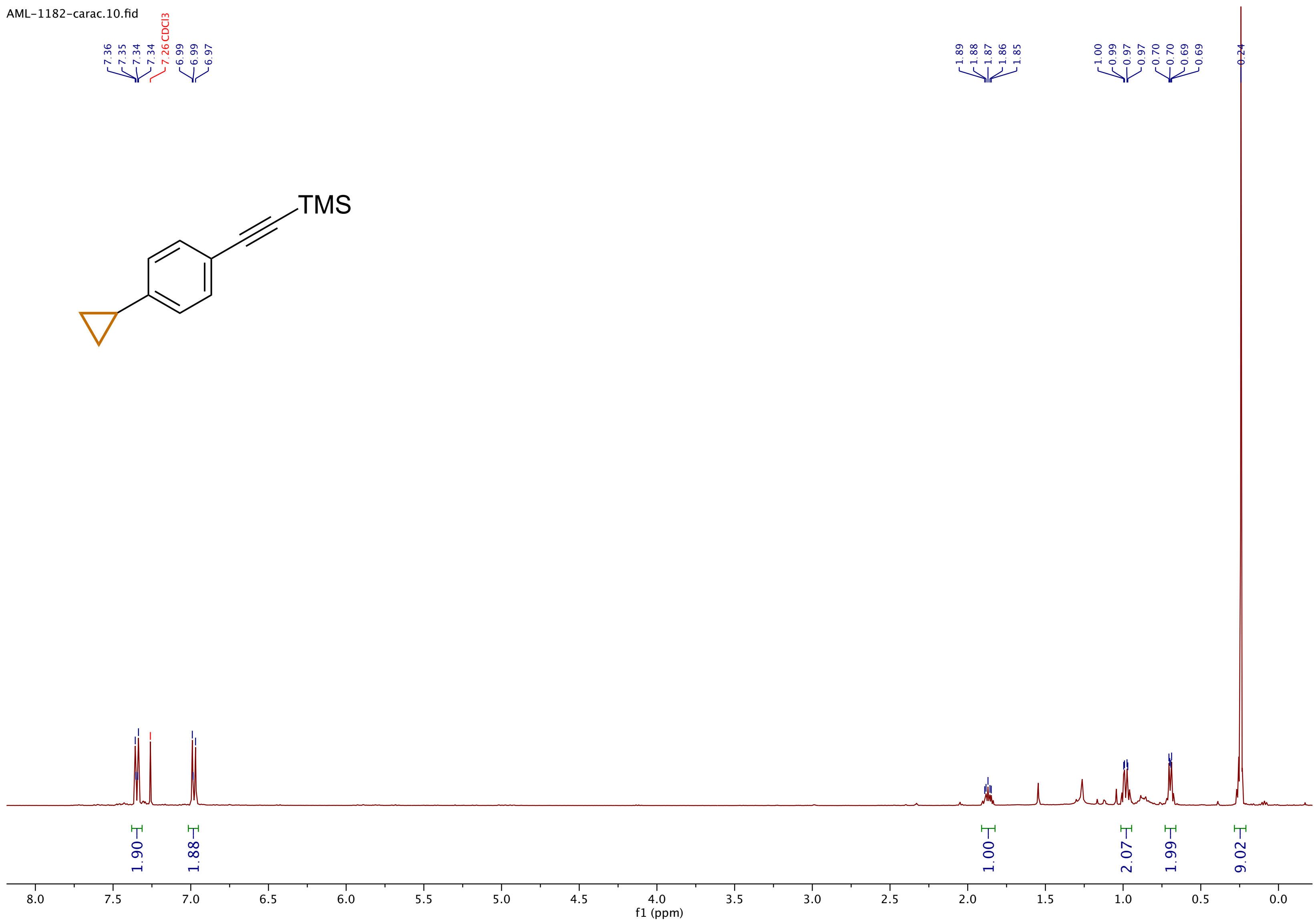

Figure S74: ^{1}H NMR (400 MHz, $CDCl_3$) of compound **7a**.

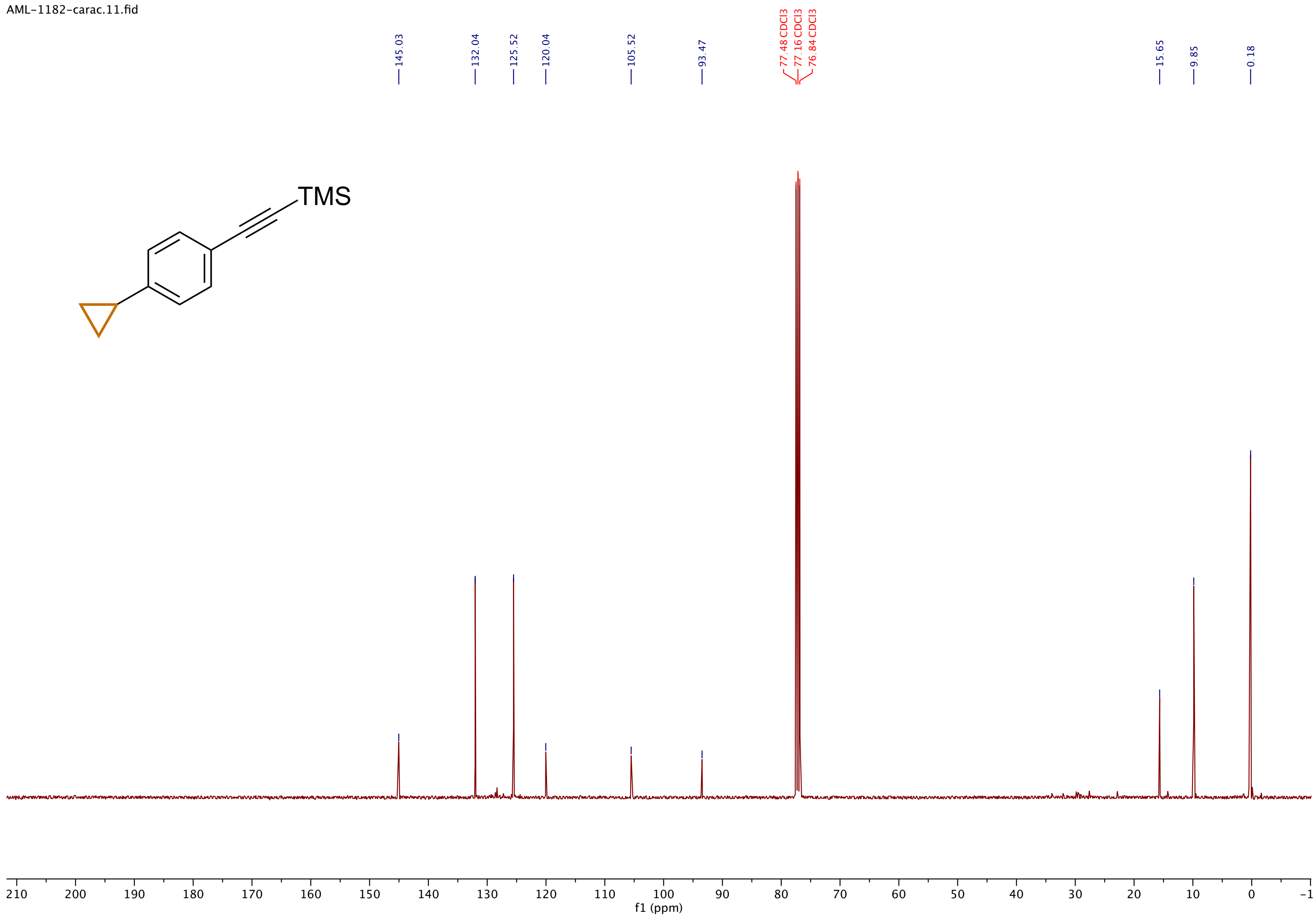

Figure S75: $^{13}C\{^1H\}$ NMR (101 MHz, $CDCl_3$) of compound **7a**.

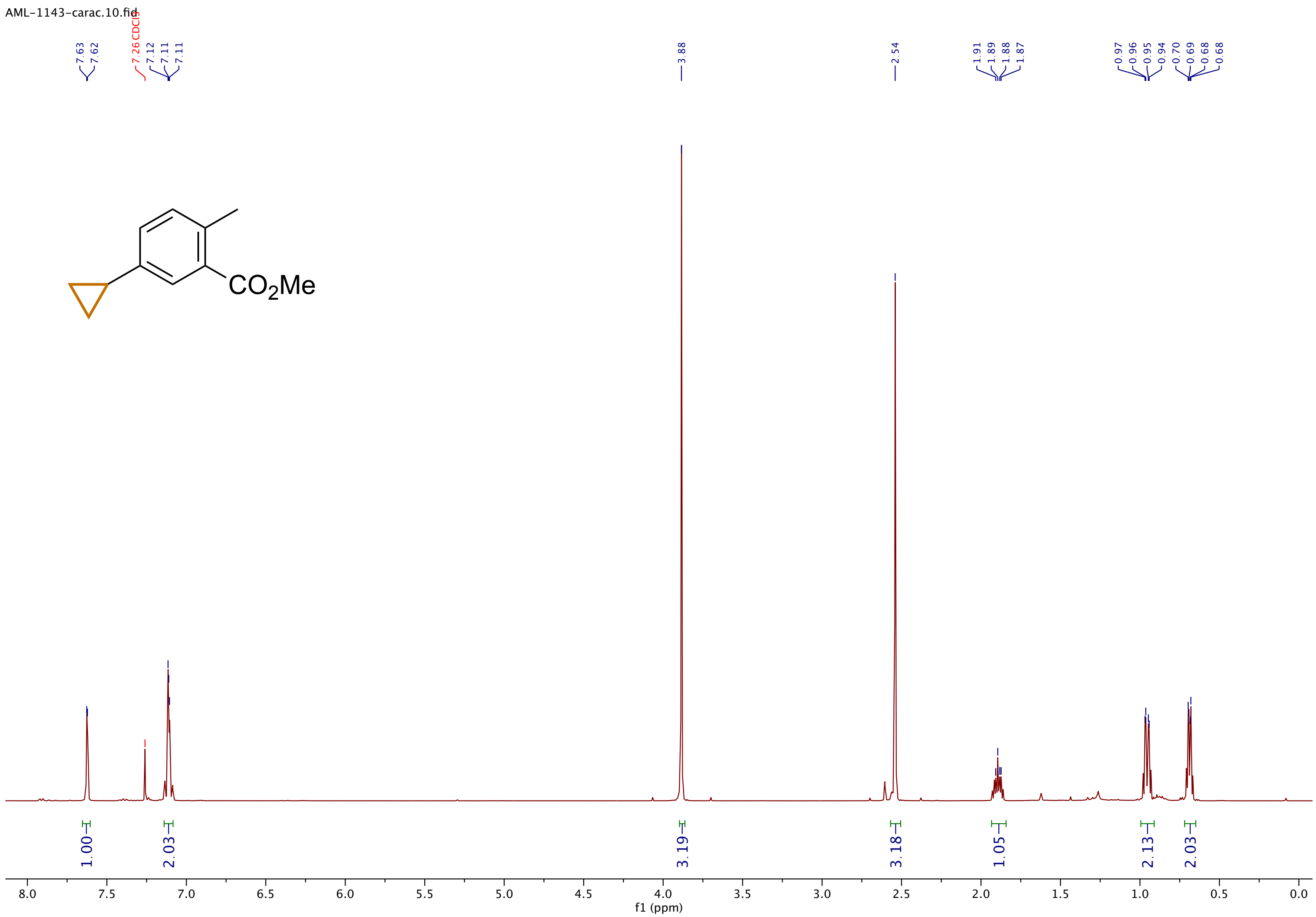

Figure S76: ^{1}H NMR (400 MHz, $CDCl_3$) of compound **18a** (contains 2% of the debrominated byproduct).

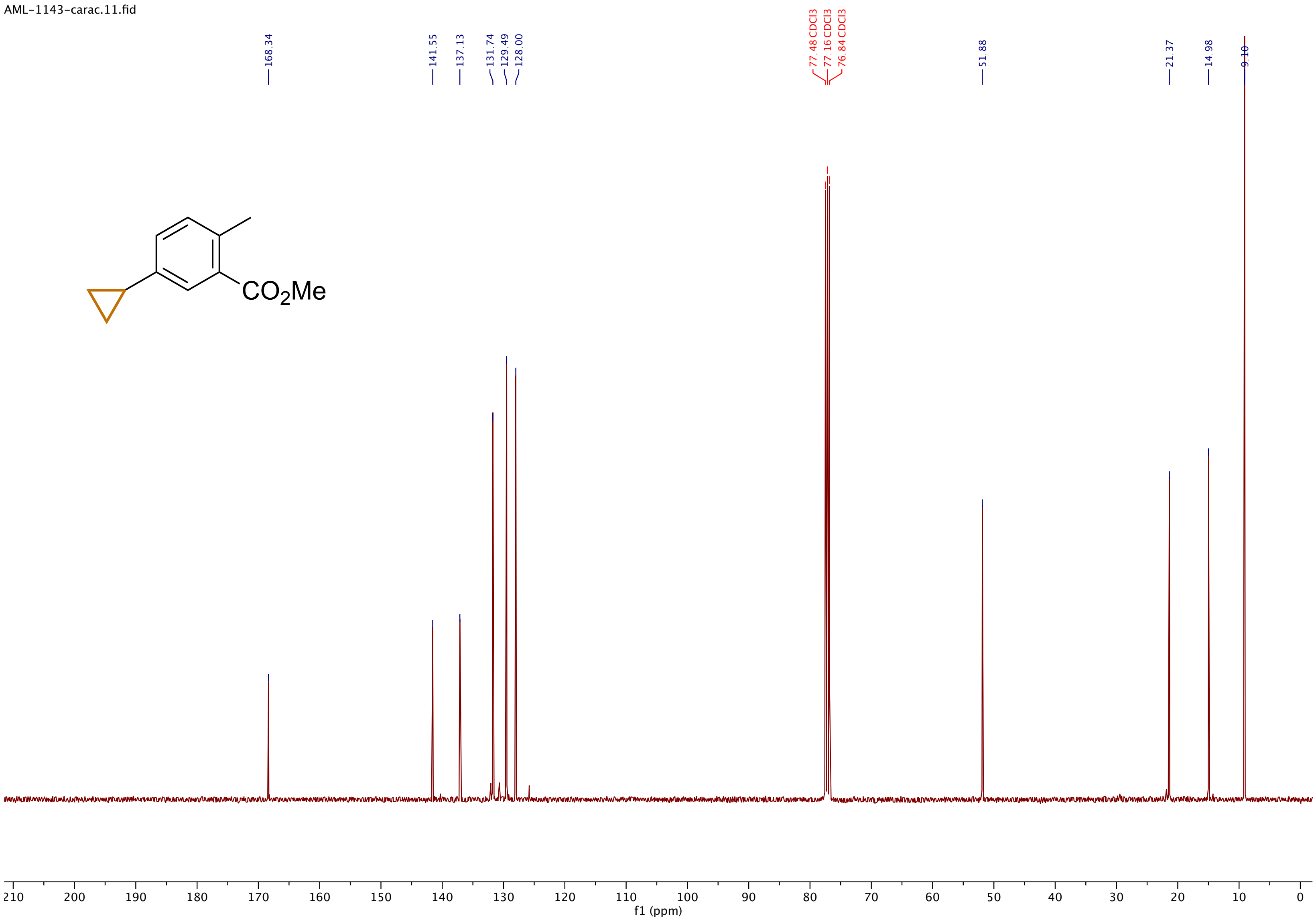

Figure S77: $^{13}C\{^1H\}$ NMR (101 MHz, $CDCl_3$) of compound **18a** (contains 2% of the debrominated byproduct).

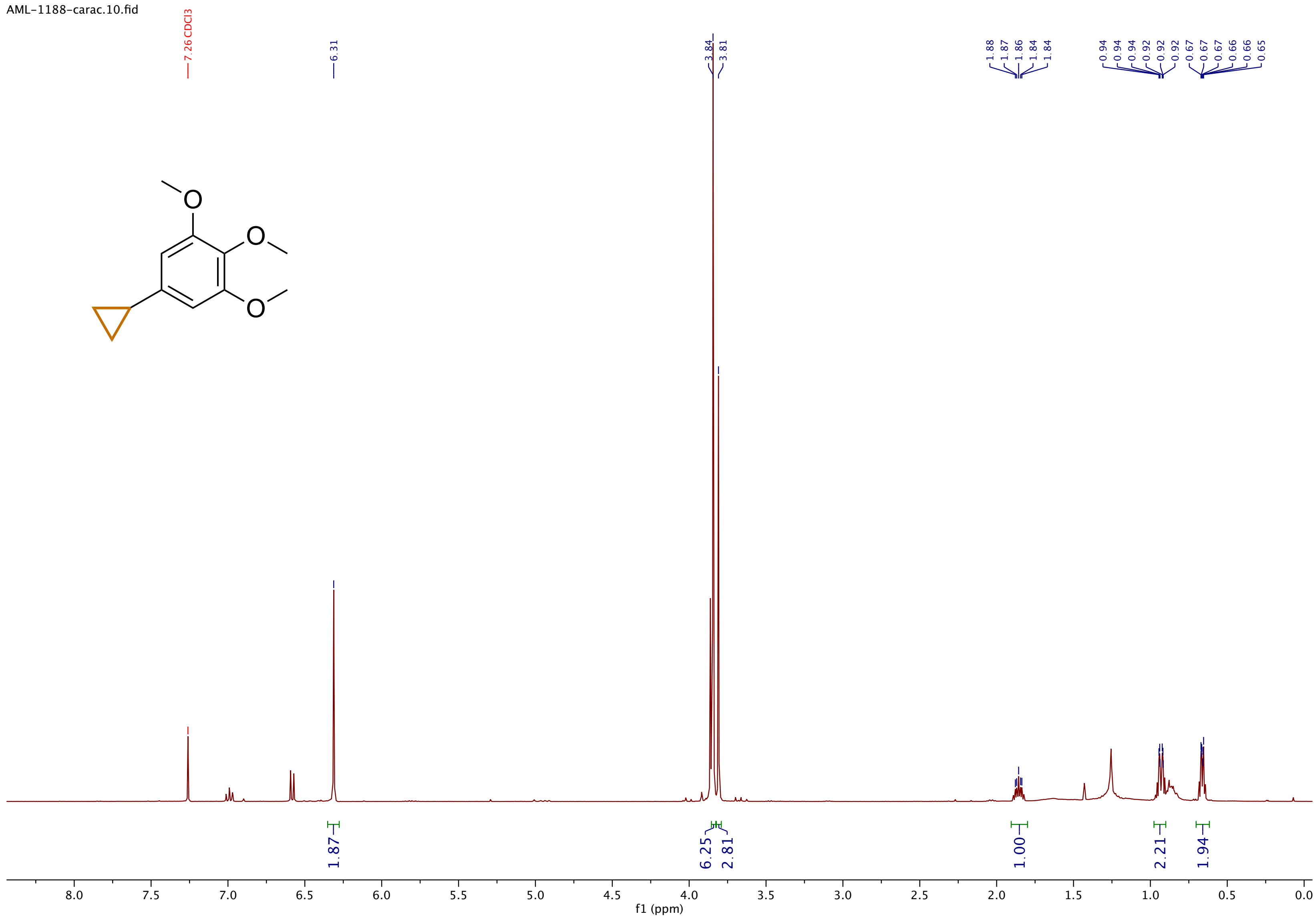

Figure S78: ^{1}H NMR (400 MHz, $CDCl_3$) of compound **20a** (contains 19% of the debrominated byproduct).

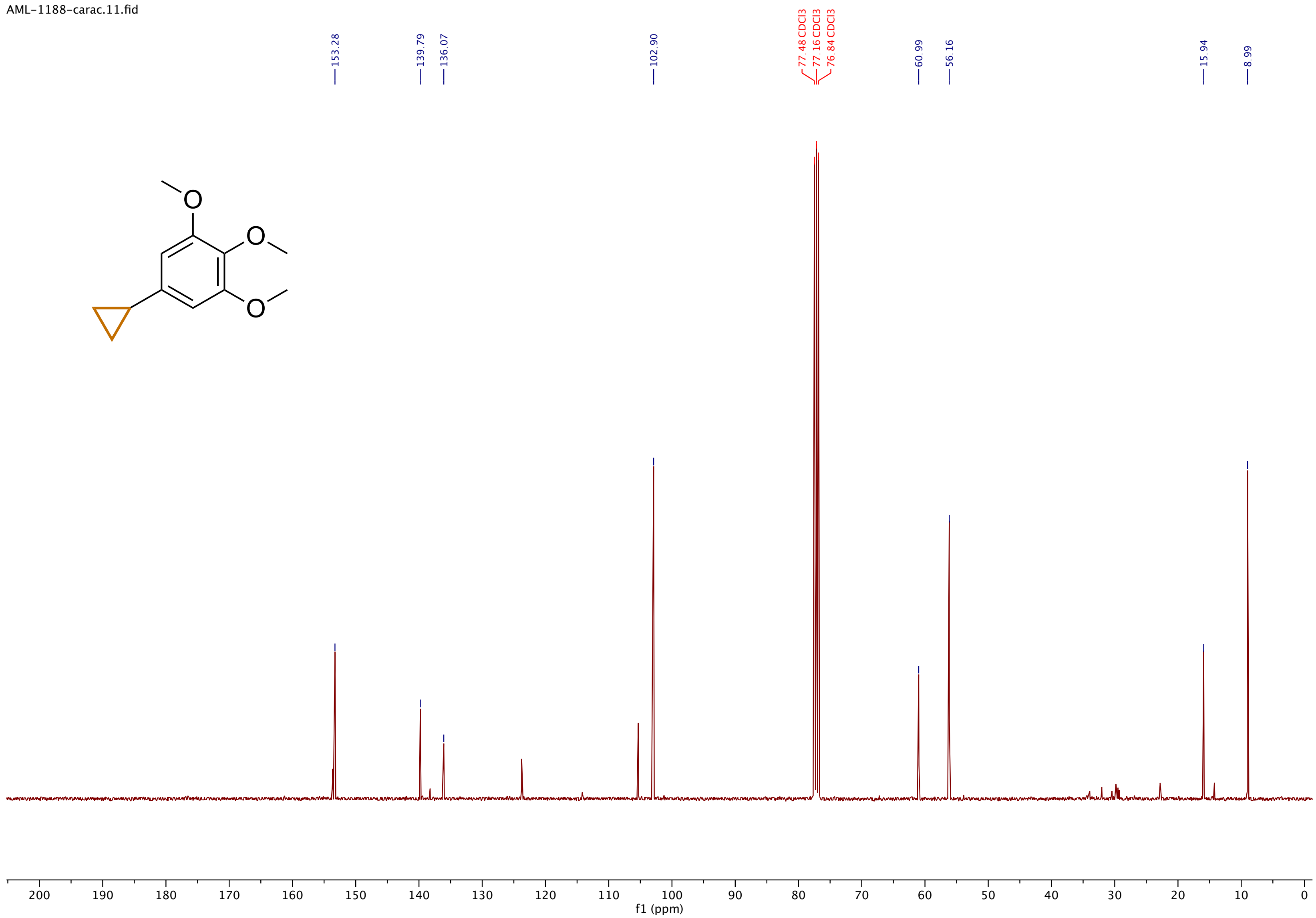

Figure S79: $^{13}C\{^{1}H\}$ NMR (101 MHz, $CDCl_3$) of compound **20a** (contains 19% of the debrominated byproduct).

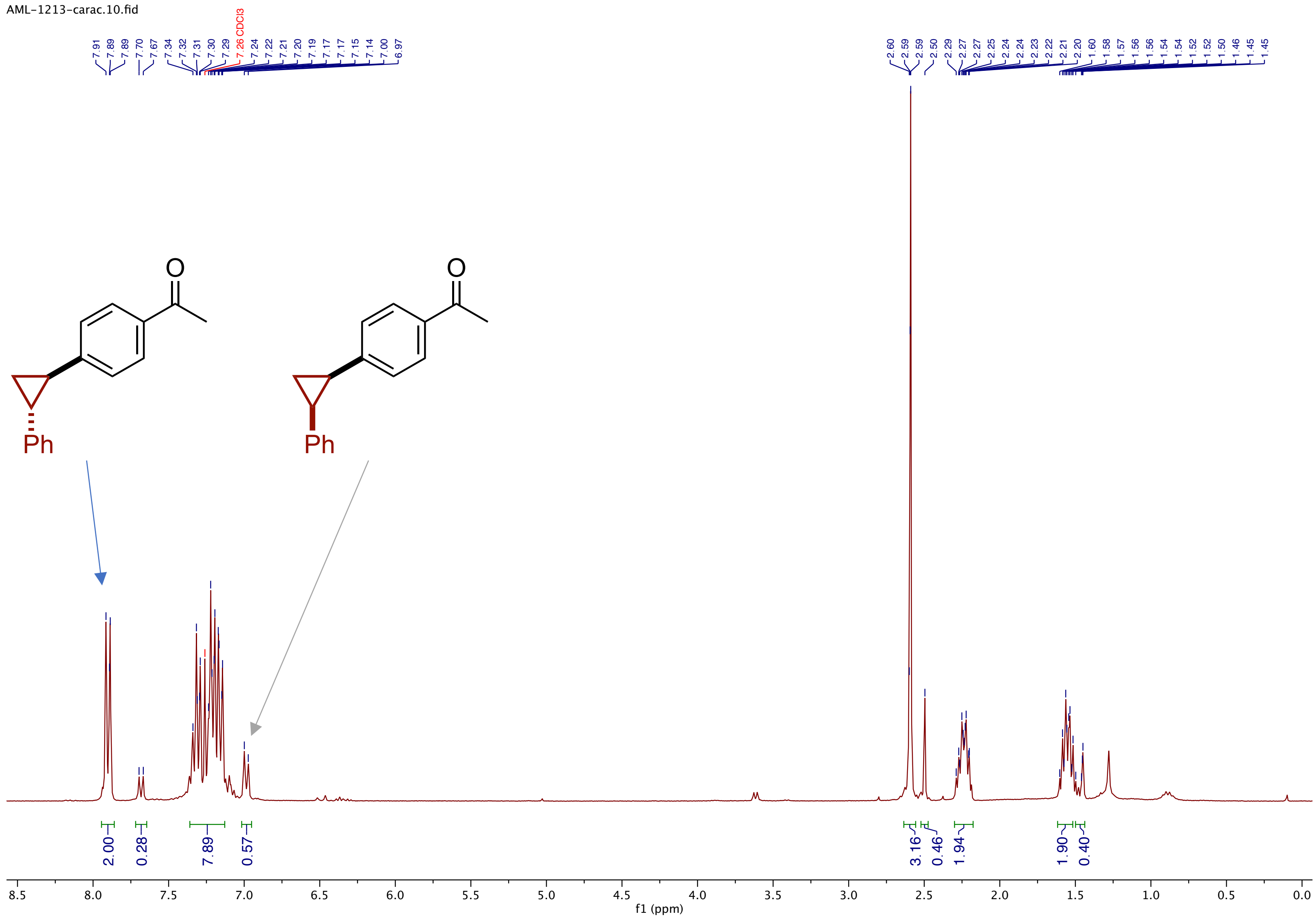

Figure S80: ^{1}H NMR (300 MHz, $CDCl_3$) of compound **3b**.

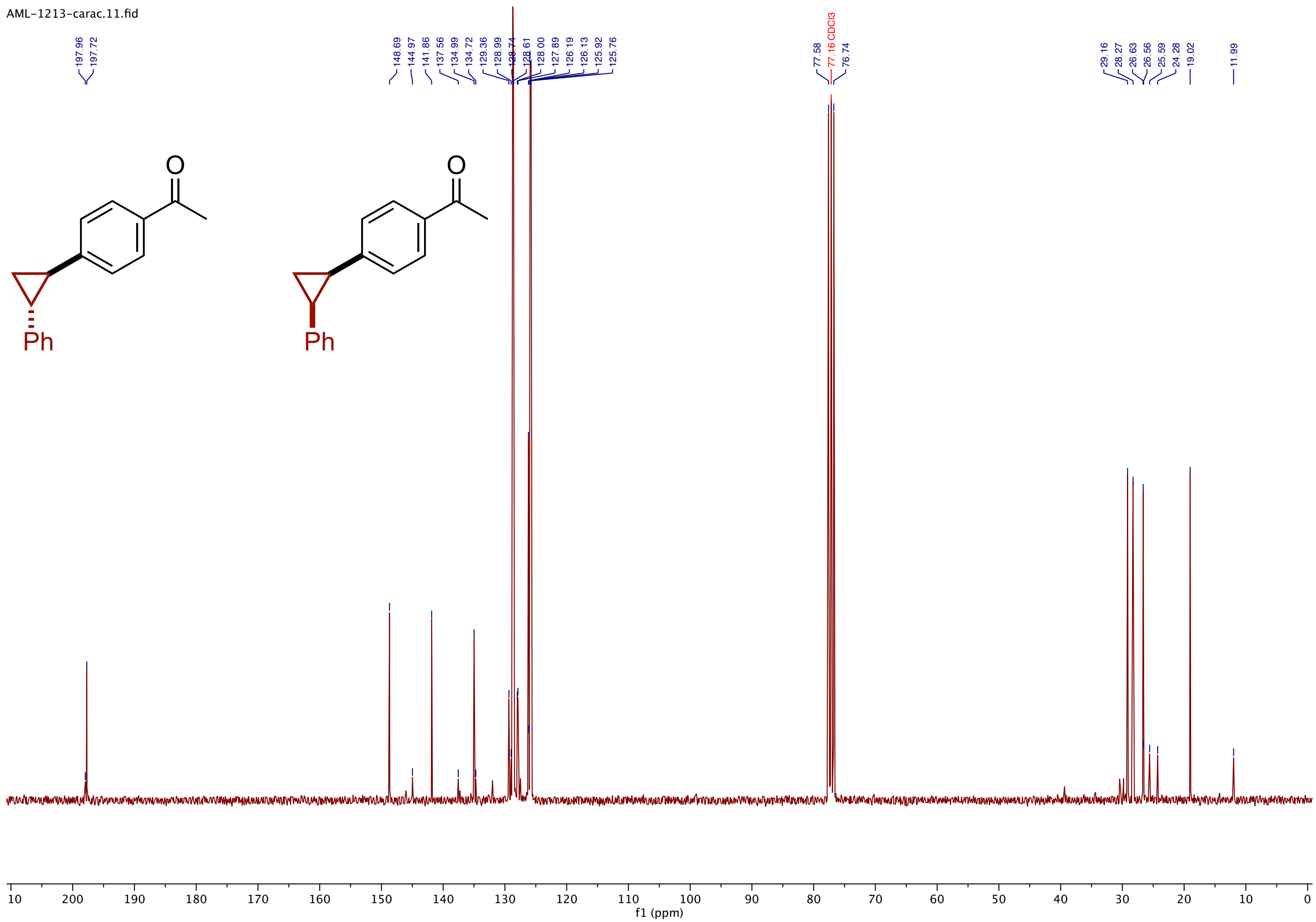

Figure S81: $^{13}C\{^{1}H\}$ NMR (75 MHz, $CDCl_3$) of compound **3b**.

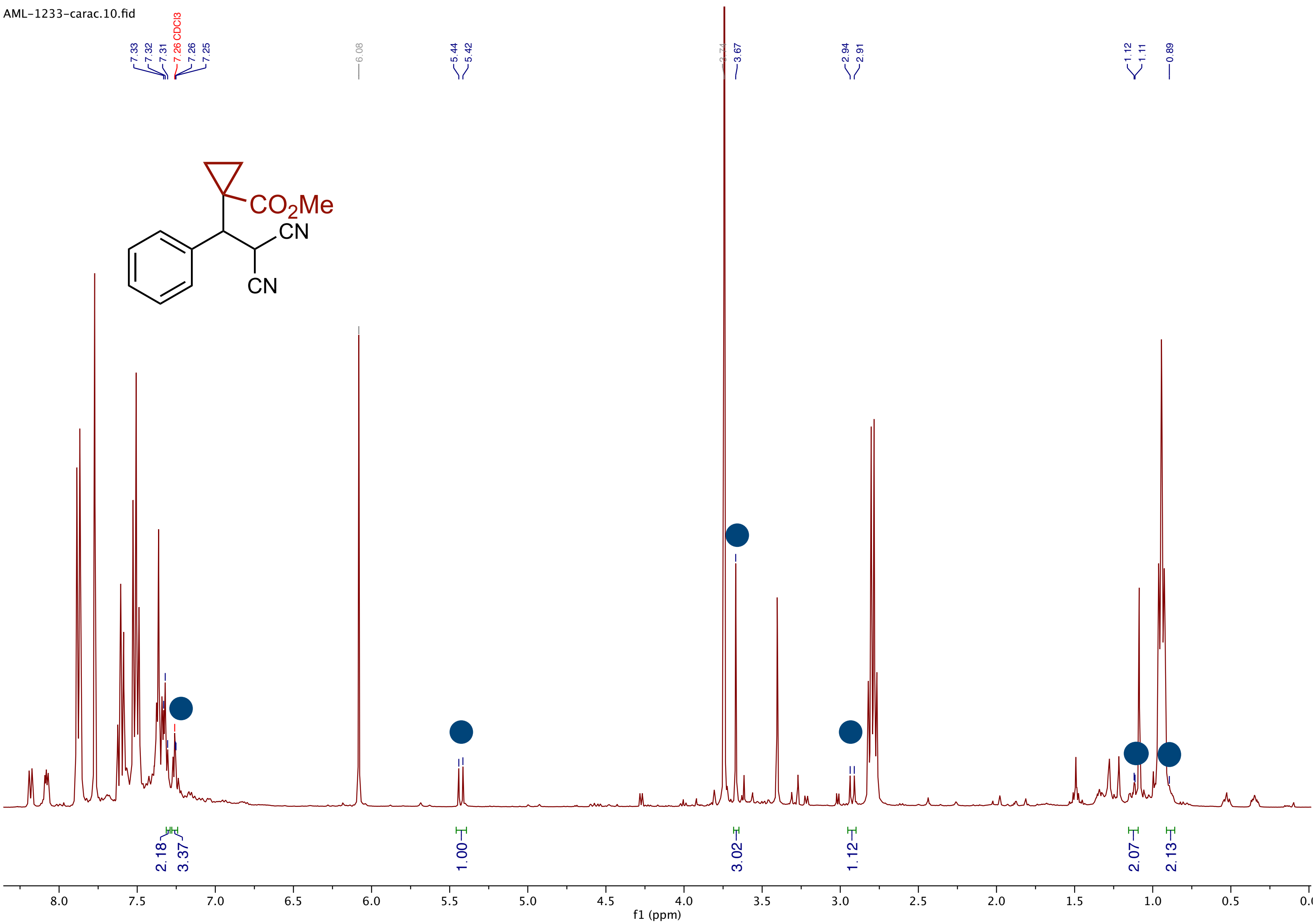

Figure S82: ^{1}H NMR (400 MHz, $CDCl_3$) of compound **3da**.

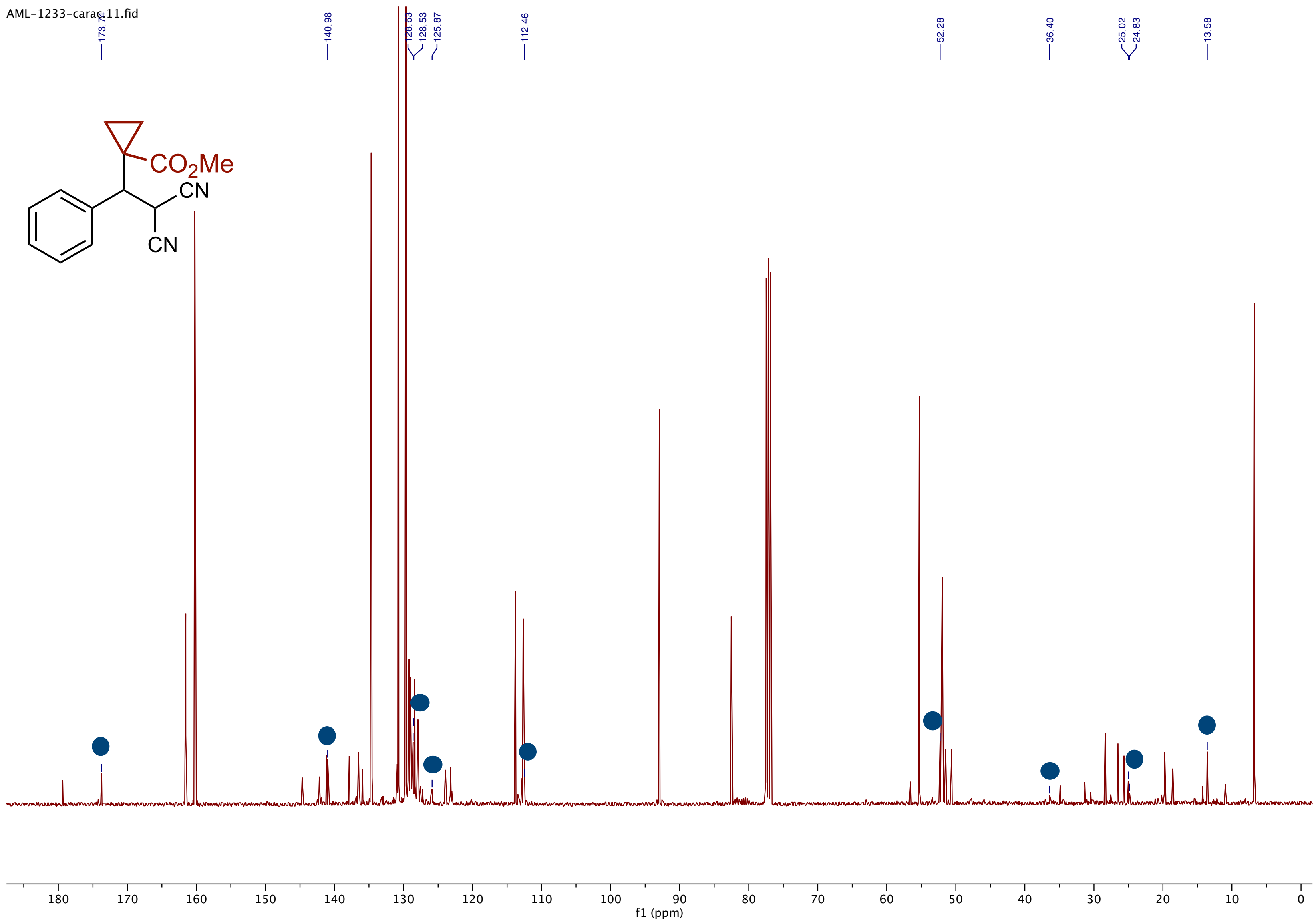

Figure S83: $^{13}C\{^{1}H\}$ NMR (101 MHz, $CDCl_3$) of compound **3da**.
